# Novel fungal diphenyl ether biosynthetic gene clusters encode a promiscuous oxidase for elevated antibacterial activities†

**DOI:** 10.1039/d4sc01435a

**Published:** 2024-07-29

**Authors:** Qingpei Liu, Shuaibiao Gao, Jin Fang, Yifu Gong, Yiling Zheng, Yao Xu, Dan Zhang, Jiayuan Wei, Liangxiu Liao, Ming Yao, Wenjing Wang, Xiaole Han, Fusheng Chen, István Molnár, Xiaolong Yang

**Affiliations:** a School of Pharmaceutical Sciences, South-Central Minzu University Wuhan 430074 P.R. China 2019001@mail.scuec.edu.cn; b VTT Technical Research Centre of Finland FI-02044 VTT Espoo Finland istvan.molnar@vtt.fi; c School of Chemistry and Materials Science, South-Central Minzu University Wuhan 430074 P.R. China; d School of Life Sciences, Guizhou Normal University Guiyang 550025 P.R. China; e College of Food Science and Technology, Huazhong Agricultural University Wuhan 430070 P.R. China

## Abstract

Diphenyl ethers (DPEs) are produced by filamentous fungi using polyketide synthases (PKSs) directly, or *via* Cu oxidase-catalyzed oxidative rearrangements of benzophenone intermediates. Here, we use heterologous expression to reveal a third route towards DPEs in *Preussia isomera* that relies on an oxidative multienzyme cascade to convert a PKS-generated, ester-linked didepside to depsidones and further to DPEs, and apply comparative genomics to identify conserved biosynthetic gene clusters for this pathway in multiple fungi. The distribution of DPE products is modulated by the expression chassis upon pathway reconstitution. Among the post-PKS enzymes, the DpeH tyrosinase shows considerable substrate promiscuity towards synthetic DPE analogues. By creating hybrid enzymes with a DpeH orthologue from *Aspergillus nidulans*, we identify the *C*-terminal region of DpeH to alter substrate recognition. Our work highlights an evolutionarily conserved way to produce DPEs, and provides enzymatic tools to generate DPE analogues with broad spectrum antibiotic activity against multidrug-resistant human pathogens.

## Abstract 2

This work describes a novel pathway for fungal diphenyl ether (DPE) biosynthesis, including a promiscuous hydroxylase whose substrate range is modulated by its *C*-terminus. DPEs may provide antibiotics against multidrug-resistant human pathogens.

## Graphical abstract

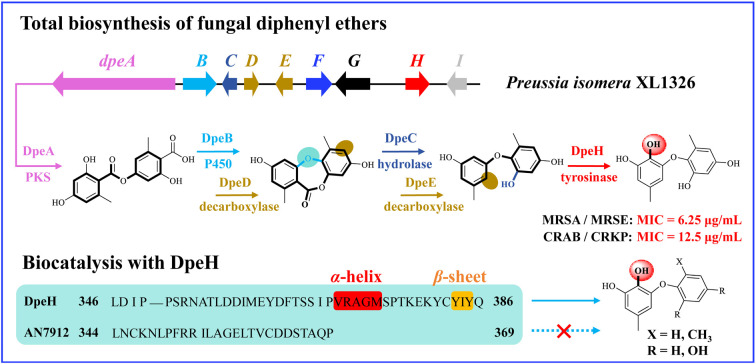

Naturally occurring didepsides, encompassing two hydroxybenzoic acid moieties connected by an ester linkage, are common lichen metabolites and have also been isolated from a limited number of fungi. Didepsides display antitumor, antimalarial, antibacterial, antifouling, and various human enzyme-inhibitory activities.^1^ The ester linkage may form by oxidative coupling or rearrangements after the release of orsellinic acid or its derivatives from polyketide synthase enzymes (PKSs).^1,2^ However, PKSs directly affording didepsides have also been reported recently, among which DrcA from *Aspergillus duricaulis*,^3^ MollE from *Ovatospora* sp.,^4^ and DepH from *A.* sp. SCSIO SX7S7 ^5^ condense acyl carrier protein-bound intermediates to form ester-linked didepsides (Fig. 1A). The enzymes encoded by gene clusters containing such PKSs may further elaborate the didepsides. Thus, cytochrome P450 monooxygenases (P450s) such as MollD and DepG install an ether bond to form depsidones (DEPs),^4,5^ while nonribosomal peptide synthetases (such as DrcB) or prenyltransferases (*e.g.*, MollF) form composite natural products (Fig. 1A).^3,4^

**Fig. 1:**
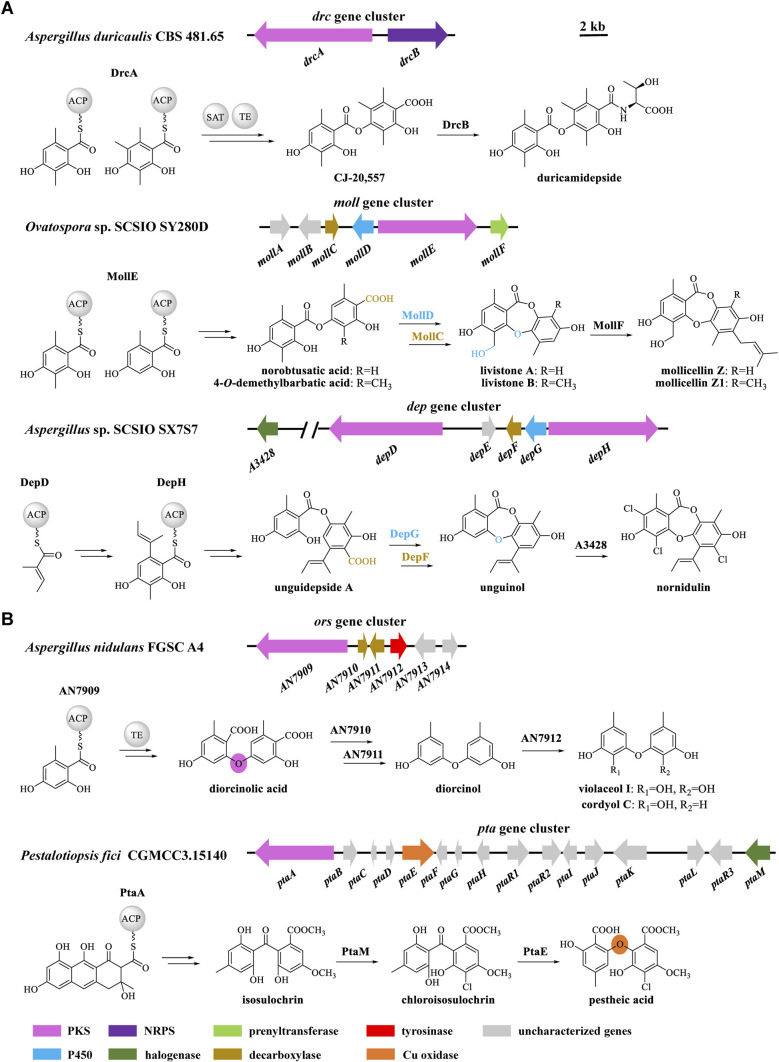
Biosynthetic gene clusters in fungi and models for the biosynthesis of (A) didepsides; and (B) DPEs.

Previously, we identified a novel PKS, Preu6 (renamed DpeA here) from *Preussia isomera* XL1326, which utilizes collaborating starter acyl transferase (SAT) and thioesterase (TE) domains to form didepside 1 (Fig. 2D).^6,7^ In addition to *dpeA*, the corresponding cluster contains eight genes, *dpeB*–*dpeI* (GenBank accession PP925597, Fig. 2A). We reconstituted this cluster and delineated the reaction order of the encoded enzymes using stepwise heterologous expression in *Saccharomyces cerevisiae* BJ5464-NpgA^8,9^ (Table S1 and Fig. S1†). The results revealed that DpeB (P450, 49% identity to DepG of *A.* sp. SCSIO SX7S7 ^5^) and DpeD (decarboxylase, 42% identity to DepF) transform didepside 1 to two DEPs (2 and 3), similar to the reactions catalyzed by their DepGF orthologues during the formation of unguinol (Fig. 1A).^5^ Next, DpeC (predicted α/β-hydrolase), DpeE (putative methyltransferase with a Methyltransf_23 conserved domain, pfam13489), DpeF (deduced methyltransferase with a Methyltransf_2 conserved domain, pfam00891), and DpeH (putative tyrosinase) convert DEP 3 to a series of DPEs, 4–8, among which DPE 8 is a new compound (Fig. 2B and D).

**Fig. 2:**
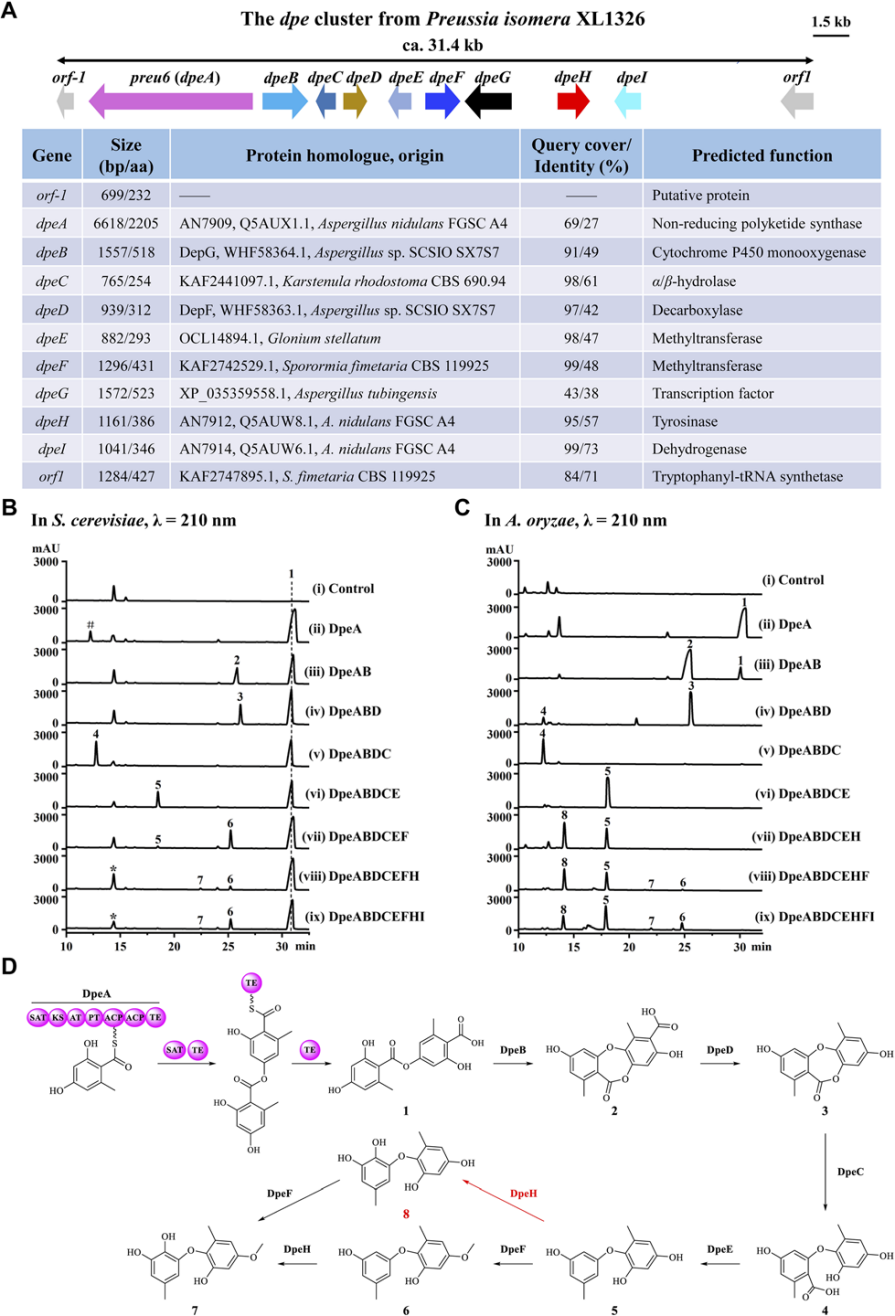
Characterization of the *dpe* gene cluster in *Preussia isomera*. (A) Schematic representation and annotation of the *dpe* cluster. (B and C) Product profiles of *S. cerevisiae* BJ5464-NpgA or *A. oryzae* NSAR1 transformants expressing the indicated gene combinations, respectively. *Control*, the corresponding empty vectors. *, a mixture of an endogenous yeast metabolite with trace amounts of 8 (detected by MS); #, orsellinic acid. (D) Biosynthetic model for DPEs 7 and 8.

Since the production of 7 was very inefficient in the yeast system, we could not detect any further transformations with DpeI, a putative dehydrogenase (73% identity to AN7914 of *A. nidulans* with an unknown function^10,11^). Thus, we introduced the *dpe* biosynthetic gene combinations defined in BJ5464-NpgA into *A. oryzae* NSAR1.^12^ The biosynthetic steps catalyzed by DpeABDCE to yield compounds 1–5 were identical in the two systems, although small amounts of 3 were also converted to 4 by endogenous enzymes in *A. oryzae* (Fig. 2C and S2†). DpeE, a predicted SAM-dependent methyltransferase (Fig. S3A†), acted as a decarboxylase in both chassis to convert 4 to 5, as precedented by other enzymes with an apparent methyltransferase fold.^13^ This decarboxylase activity was not dependent on the presence of SAM as verified by *in vitro* reconstitution of the recombinant DpeE enzyme (Fig. S3B and C†). Remarkably, *A. oryzae* NSAR1 preferred to transform 5 to 8 using DpeH (Fig. S4†), in contrast to the yeast BJ5464-NpgA that utilized DpeF to convert 5 to 6 (Fig. 2B). Despite the higher productivity of the *A. oryzae* chassis, DpeI remained apparently nonfunctional, nor did purified, recombinant DpeI catalyze any conversions of 1–8*in vitro* (Fig. S5†). Thus, the function of DpeI, if any, requires further investigation. Meanwhile, 8 was shown to be transformed to 7 using *in vivo* biocatalytic conversions with DpeF-producing *S. cerevisiae* or *A. oryzae* strains (Fig. S6†).

Taken together, the *dpe* cluster relies on the transformation of a PKS-generated, ester-linked didepside to DEPs, and further to various DPEs in a multistep, multi-enzyme, oxidative reaction cascade. This is different from the *ors* cluster of *A. nidulans* FGSC A4 where PKS AN7909 alone affords a DPE;^10,14^ or from the *pta* cluster of *Pestalotiopsis fici* where the Cu oxidase PtaE catalyzes an oxidative rearrangement to generate the DPE product (Fig. 1B).^15^

To date, approximately 170 DPEs have been isolated from 46 fungal species (Table S2†). Upon alignment of the 31 available genome sequences of DPE producers (Table S2†) to the known clusters *ors*, *pta*, and *dpe*, we identified putative DPE clusters in 20 species (Table S3†), although the existence of divergent DPE clusters cannot be excluded in the remaining species either. The identified clusters could be classified into three categories. Twelve DPE clusters belong to **Type I** (*ors*-like clusters; Fig. 3A), featuring PKSs with high similarity to AN7909. These are predicted to produce diorcinolic acid-like DPEs utilizing these PKSs alone.^10,14^ Correspondingly, the DPE compounds isolated from these 12 species are overwhelmingly diorcinol derivatives (Table S4†). Three DPE clusters belong to **Type II** (*pta*-like clusters; DPE products similar to pestheic acid; Fig. 3B and Table S5†), in which PtaE-like Cu oxidases are predicted to form the ether linkage.^15^ Finally, five DPE clusters belong to **Type III** (*dpe*-like clusters; Fig. 3C), featuring conserved PKSs, P450s, and DpeD-like decarboxylases that presumably generate DEPs en route to DPEs (Table S6†). Three of the five **Type III** clusters (those from *Corynespora*, *Boeremia*, and *Aspergillus* spp.) lack DpeC orthologues that would hydrolyze the ester bond of DEPs to form DPEs. We hypothesize that endogenous hydrolases encoded elsewhere in the genomes of these species perform this function, just as seen in *A. oryzae* NSAR1 (Fig. 2C and S2†). Interestingly, all three cluster types are present in genus *Aspergillus*, potentially enriching the variety of DPEs in these fungi.

**Fig. 3:**
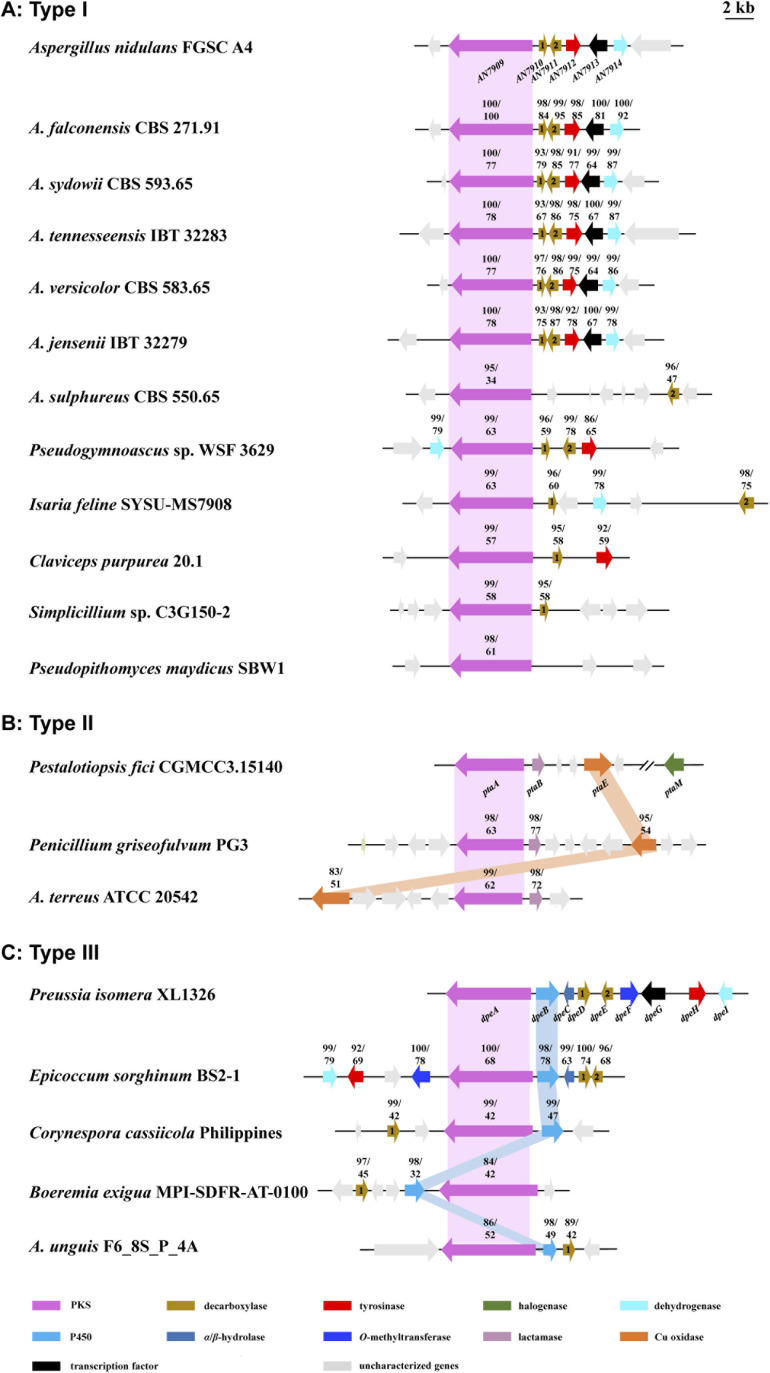
Distribution of the proposed DPE biosynthetic gene clusters in fungi. (A) *ors*-like gene clusters (**Type I**), in which the PKSs alone may produce DPEs. (B) *pta*-like gene clusters (**Type II**), in which conserved PKSs and PtaE-like Cu oxidases are predicted to be involved in the formation of DPEs. (C) *dpe*-like gene clusters (**Type III**), in which conserved PKSs and P450s may produce DEPs that are further converted to DPEs. Arrows with identical colors indicate orthologous genes. The numbers above the genes indicate the percent identities of the encoded proteins to their orthologues in the corresponding model cluster.

Next, we tested the activities of compounds 1–8 against eight antibiotic-resistant bacteria. DEP 3 was active against Gram-positive bacteria, while DPEs 7 and 8 with the C-2′ hydroxy functionality installed by DpeH displayed broad-spectrum antibacterial activities. Remarkably, the novel DPE 8 (yield: 259.8 ± 15.9 mg L^−1^ in *A. oryzae*; Fig. S7†) exhibited potent activities against multidrug-resistant *Staphylococcus epidermidis*, methicillin-resistant *Sta. aureus* (MIC = 6.25 μg mL^−1^), and carbapenems-resistant *Acinetobacter baumannii* and *Klebsiella pneumoniae* (MIC = 12.5 μg mL^−1^; Table S7†).

Then, we turned to AN7912, an orthologue (57% identity) of DpeH in the *ors* cluster. AN7912 hydroxylates diorcinol (Fig. 1B)^11^ and accepts the simplified substrate 9 (Fig. 4C), but this enzyme could not convert 5 to 8 (Fig. 4B), or 6 to 7 (Fig. S8†). In contrast, DpeH converts 9, 5, and 6 to their hydroxylated derivatives (9a/b, 8, and 7, respectively; Fig. 4B and S8†). The AlphaFold2-predicted^16^ structures of AN7912 and DpeH differed both at their *N*-termini (M^1^–K^59^ in DpeH; M^1^–R^58^ in AN7912) and *C*-termini (L^346^–Q^386^ in DpeH; L^344^–P^369^ in AN7912; Fig. 4A and S9†). Replacing either or both terminal regions of AN7912 with those of DpeH showed that hybrid M2 (AN7912[M^1^–L^343^] + DpeH[L^346^–Q^386^]) could transform compounds 5 to 8, and 6 to 7 while chimera M1 (DpeH[M^1^–K^59^] + AN7912[E^59^–P^369^]) could not, revealing that the *C*-terminal region modulates substrate recognition. Correspondingly, hybrid M4 (DpeH[M^1^–L^345^] + AN7912[L^344^–P^369^]), and truncated enzyme M5 (DpeH[M^1^–L^345^]) lost the ability to convert 5 to 8, or 6 to 7 (Fig. 4B and S8†). Importantly, all tested chimeras (M1–M4) and truncated enzymes (M5–M6) retained their activities towards simplified substrate 9.

**Fig. 4:**
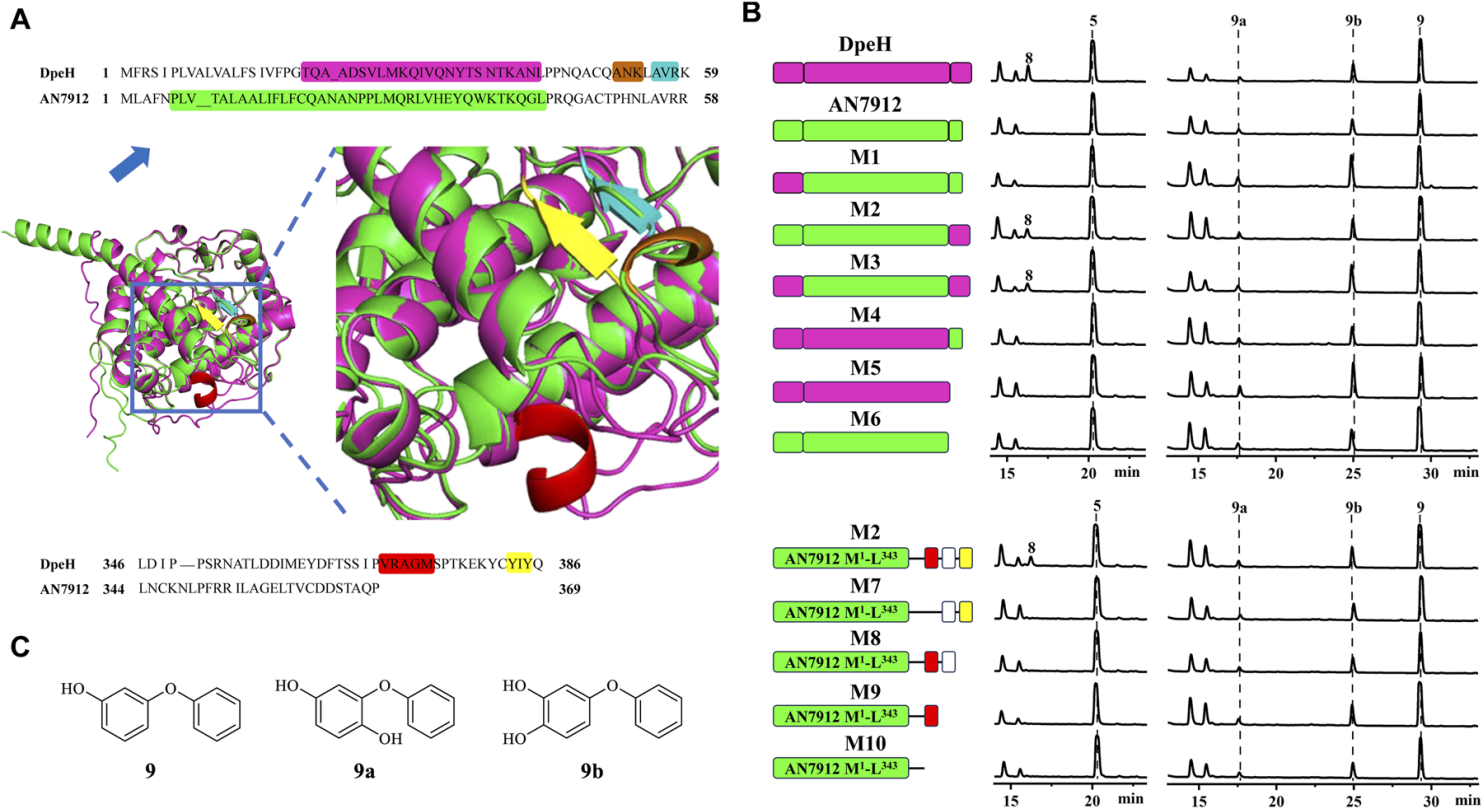
Structural elements of DpeH that modulate substrate recognition. (A) Protein structural differences between DpeH (*magenta*) and AN7912 (*green*) predicted by AlphaFold2. *Brown* or *cyan* highlights, *α*-helix or *β*-sheet, respectively, in the *N*-terminal region of DpeH (*top*); *red* or *yellow* highlights, *α*-helix or *β*-sheet, respectively, in the *C*-terminal region of DpeH (*bottom*). (B) Product profiles (210 nm) of the *A. oryzae* NSAR1 strains expressing the indicated enzymes and challenged with substrates 5 or 9, respectively. In mutant enzymes M1–M10, AN7912-derived regions are shown in *green*. In mutants M1–M5, segments from DpeH are in *magenta*. In mutants M7–M10 (derived from M2), *missing boxes* indicate deletions; *red boxes* show the *α*-helix, *white boxes* represent the linker, and *yellow boxes* indicate the *β*-sheet of DpeH. (C) Structures of compounds 9, 9a and 9b.

The *C*-terminal region of DpeH possesses a short *α*-helix (V^370^–M^374^), a linker (S^375^–C^382^), and a short *β*-sheet (Y^383^–Y^385^) that are all missing from AN7912 (Fig. 4A). Deleting the *α*-helix from chimera M2 (*i.e.*, mutant M7: M2-Δ[V^368^–M^372^]) or progressively truncating the M2 hybrid enzyme (*i.e.*, mutants M8–M10) revealed that all these structural elements are necessary to produce 8 from 5, and 7 from 6, but their presence or absence does not affect the hydroxylation of substrate 9 (Fig. 4B and S8†).

Finally, we tested the substrate promiscuities of DpeH and AN7912 with various synthetic DPE analogues using biotransformation in the *A. oryzae* chassis. DPE 11 was partially converted to 11c by an unknown endogenous enzyme of *A. oryzae* (Fig. 5 and S10†). AN7912 showed a narrow substrate spectrum, affording only minute amounts of 17a from 17, but rejecting all other substrates (Fig. 5B). In contrast, DpeH turned out to be a promiscuous enzyme accepting all tested DPEs to generate novel “unnatural” products (Fig. 5A and D). The regiospecificity of the reaction was strict, with hydroxylation at C-2′ in all cases. With DPE 17, double hydroxylation at C-3 and C-2′ was also observed, as may have been expected considering the symmetrical structure of this substrate (Fig. 5A). Importantly, mutant M2 (AN7912[M^1^–L^343^] + DpeH[L^346^–Q^386^]) was just as promiscuous as DpeH, and afforded the same biotransformation products (Fig. S11†), confirming the essential role of the *C*-terminal region of DpeH in determining substrate specificity.

**Fig. 5:**
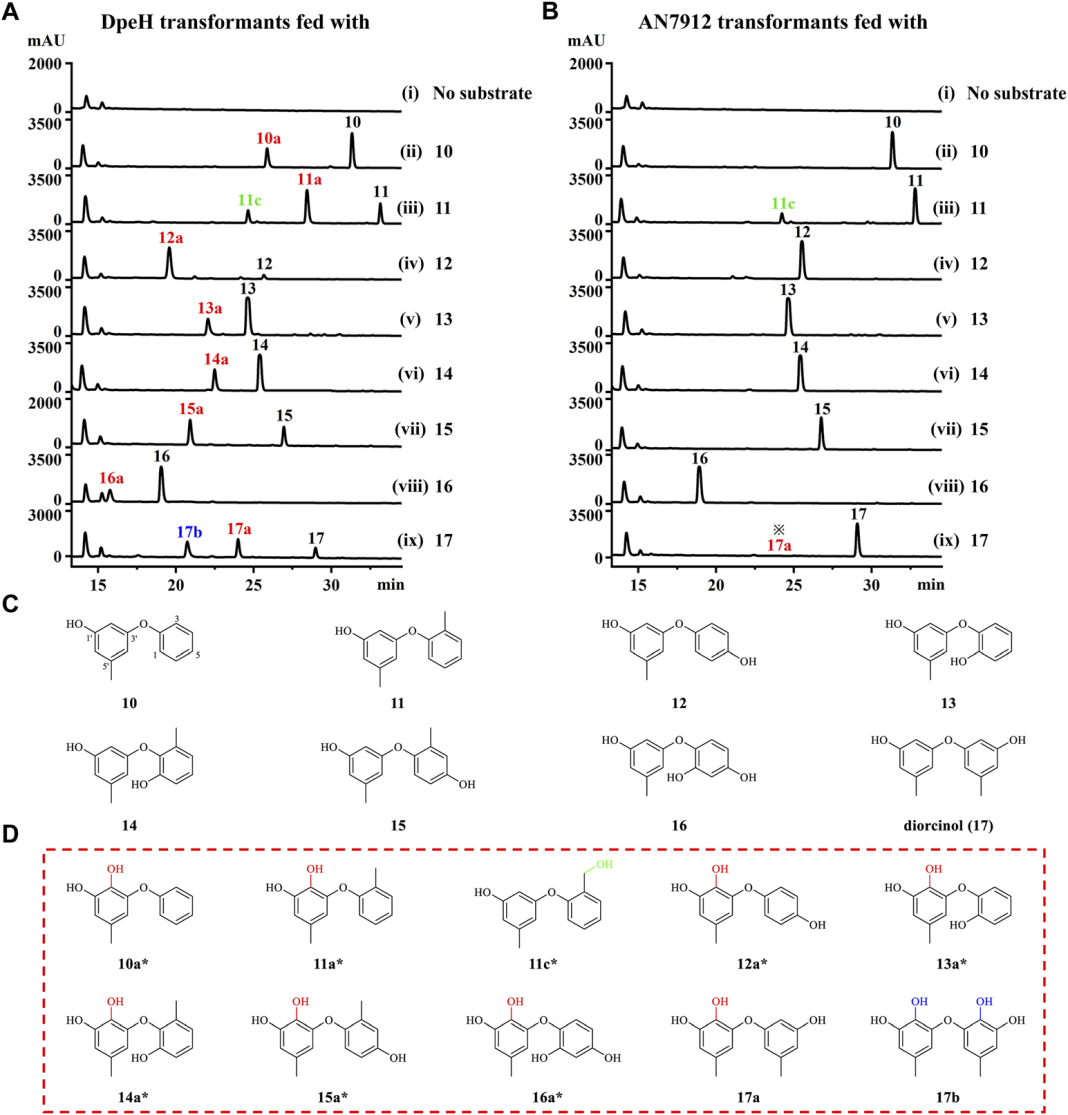
Substrate promiscuity of DpeH and AN7912. (A and B) Product profiles (210 nm) of the *A. oryzae* NSAR1 strains expressing DpeH or AN7912, and fed with substrates 10–17, respectively. ※, 17a was detectable only with MS. (C and D) Structures of substrates 10–17, DpeH products 10a–17a and 17b, and host-derived product 11c. *, New compounds.

## Conclusions

In summary, we characterized the *dpe* biosynthetic gene cluster of *Preussia isomera via* heterologous expression in *S. cerevisiae* and *A. oryzae* chassis. Most predicted DPE biosynthetic clusters in fungi seem to rely on a conserved PKS to directly form DPE analogues from orsellinic acid-type nascent, PKS-bound monomers. Rarer DPE clusters afford their products by enlisting a PtaE-like Cu oxidase to catalyze the oxidative rearrangement of polyketide-derived benzophenones. In contrast, *dpe*-type clusters utilize a conserved PKS to produce analogues of the ester-linked didepside lecanoric acid 1. These are then converted to DPEs by a multienzyme cascade. With the *dpe* cluster, oxidative conversion of 1 to DEP 2 is catalyzed by DpeB, followed by decarboxylation to 3 by DpeD, oxidative ring opening to 4 by DpeC, and another decarboxylation to 5 by DpeE. DPE 5 may then be converted to the novel broad-spectrum antibiotic 8 by DpeH, or methylated by DpeF to yield 6 which is then hydroxylated by DpeH to yield another broad-spectrum antibiotic, DPE 7. Interestingly, the preference to produce 8 or 6/7 seems to be determined by the chassis, with DPE 8 dominating in *Aspergillus*, while DPEs 6 and 7 preferred in *Saccharomyces*. DpeH is a promiscuous enzyme that recognizes a variety of DPE analogues, and faithfully transforms them to their C-2′ hydroxylated derivatives. AN7912, the DpeH orthologue encoded in the *ors* cluster of *A. nidulans* is a much less promiscuous catalyst than DpeH. However, transplanting a short *C*-terminal region of DpeH (41 amino acids) broadens the substrate specificity of AN7912, and allows the resulting chimera to accept DPEs 5 and 10–17 as its substrates. Our work not only clarifies a novel pathway towards DPEs in filamentous fungi, but also provides a useful tool for synthetic biology to produce novel “unnatural” DPE analogues and highlights protein engineering strategies to broaden the substrate specificity of DpeH orthologues. Meanwhile, DPEs 7 and 8 may further be evaluated as potent broad-spectrum antibiotics against multidrug-resistant Gram-positive and Gram-negative pathogens on the WHO global priority list.

## Author contributions

Conceptualization, project administration, supervision, and resources, Q. L., I. M., and X. Y.; methodology, investigation, and data curation, Q. L., S. G., J. F., Y. G., Y. Z., Y. X., D. Z., and J. W.; formal analysis, Q. L., S. G., J. F., Y. G., L. L., M. Y., W. W., X. H., and F. C.; writing – original draft, Q. L., S. G., and J. F.; writing – review & editing, Q. L., I. M., and X. Y.; funding acquisition, Q. L., X. Y., W. W., and I. M.

## Conflicts of interest

I. M. has disclosed financial interests in TEVA Pharmaceuticals Hungary which are unrelated to the subject of the research presented here. All other authors declare no competing financial interests.

## Supplementary Material

SC-015-D4SC01435A-s001

## Data Availability

Materials and methods, additional tables and figures, and spectroscopic data are available in the ESI.† The *dpe* gene cluster of *P. isomera* XL1326 has been deposited in GenBank under accession code PP925597. The genome sequences used for comparative genomics were obtained from the NCBI GenBank or the Joint Genome Institute (JGI) MycoCosm databases (Table S2†).
