# Supplementary material for: Novel fungal diphenyl ether biosynthetic gene clusters encode a promiscuous oxidase for elevated antibacterial activities
Source: Chem Sci. 2024 Jul 29;15(35):14248–53. doi: 10.1039/d4sc01435a (PMC11320064; doi:10.1039/d4sc01435a)
Supplement: SC-015-D4SC01435A-s001 [file SC-015-D4SC01435A-s001.pdf]

Supplementary Information (SI) for Chemical Science.
This journal is © The Royal Society of Chemistry 2024

Supporting Information

for

Novel Fungal Diphenyl Ether Biosynthetic Gene Clusters Encode a Promiscuous Oxidase for Elevated Antibacterial Activities

Qingpei Liu[1,#], Shuaibiao Gao[1,#], Jin Fang[1,#], Yifu Gong[1], Yiling Zheng[1], Yao Xu[1], Dan Zhang[1], Jiayuan Wei[1], Liangxiu Liao[1], Ming Yao[1], Wenjing Wang[1], Xiaole Han[3], Fusheng Chen[4,5], István Molnár[2,*], and Xiaolong Yang[1,*]

[1] School of Pharmaceutical Sciences, South-Central Minzu University, Wuhan 430074, P.R. China

[2] VTT Technical Research Centre of Finland, FI-02044 VTT, Espoo, Finland

[3] School of Chemistry and Materials Science, South-Central Minzu University, Wuhan 430074, P.R. China

[4] School of Life Sciences, Guizhou Normal University, Guiyang 550025, P.R. China

[5] College of Food Science and Technology, Huazhong Agricultural University, Wuhan 430070, P.R. China

[#] These authors contributed equally to this work.

* Corresponding authors: 2019001@mail.scuec.edu.cn; istvan.molnar@vtt.fi

Contents

1 SI Materials and Methods 1

1.1 Molecular biology, microbiology and bioinformatics 1

1.1.1 Construction of *Saccharomyces cerevisiae* expression vectors 1

1.1.2 Construction of *Aspergillus oryzae* expression vectors 3

1.1.3 Total biosynthesis of the depsidones and diphenyl ethers 7

1.1.4 Quantitative analysis of the formation of compounds **1-8** 8

1.1.5 Protein expression, purification, and *in vitro* biochemical assay 9

1.1.6 Comparative genome analysis 9

1.2 Isolation and characterization of the DEPs and DPEs 10

1.2.1 General methods 10

1.2.2 Compound **2** 10

1.2.3 Compound **3** 11

1.2.4 Compound **4** 11

1.2.5 Compound **5** 12

1.2.6 Compound **6** 12

1.2.7 Compound **7** 12

1.2.8 Compound **8** 13

1.3 Antibacterial assay 13

1.4 Chemical synthesis of the DPEs 14

1.4.1 Preparation of 3-(methoxymethoxy)-5-methylphenol 14

1.4.2 General procedure for the synthesis of **10**, **11**, **12** and **17** 14

1.4.3 General procedure for the synthesis of **13**, **14**, **15**, **16** and **26** 18

1.5 Biotransformation and characterization of the DPE products 24

1.5.1 Feeding experiments 24

1.5.2 Compounds **9a**/**b** 24

1.5.3 Compound **10a** 25

1.5.4 Compounds **11a**/**c** 25

1.5.5 Compound **12a** 26

1.5.6 Compound **13a** 26

1.5.7 Compound **14a** 27

1.5.8 Compound **15a** 27

1.5.9 Compound **16a** 27

1.5.10 Compounds **17a**/**b** 28

2 SI Tables 29

Table S1. Plasmids used in this study 29

Table S2. DPE-producing fungi reported in the literature, and genome sequence assemblies used for comparative genomics 32

Table S3. Putative DPE gene clusters identified in 20 species of fungi 34

Table S4. DPE compounds produced by species with a **Type I** biosynthetic gene cluster 35

Table S5. DPE compounds produced by species with a **Type II** biosynthetic gene cluster 36

Table S6. DPE compounds produced by species with a **Type III** biosynthetic gene cluster 37

Table S7. Antibacterial activities of compounds **1-8** (MIC, μg/mL)[a] 39

Table S8. Primers used in this study 40

Table S9. ^{1}H NMR and ^{13}C NMR data 43

Table S9.1. Compounds **7** and **8** (in methanol-d_4) 43

Table S9.2. Compounds **9**, **9a** and **9b** (in DMSO-d_6) 44

Table S9.3. Compounds **10** and **10a** (in methanol-d_4) 45

Table S9.4. Compounds **11**, **11a** and **11c** (in methanol-d_4) 46

Table S9.5. Compounds **12** and **12a** (in methanol-d_4) 47

Table S9.6. Compounds **13** and **13a** (in methanol-d_4) 48

Table S9.7. Compounds **14** and **14a** (in DMSO-d_6) 49

Table S9.8. Compounds **15** and **15a** (in methanol-d_4) 50

Table S9.9. Compounds **16** and **16a** (in methanol-d_4) 51

Table S9.10. Compounds **17** and **17a** (in methanol-d_4) 52

3 SI Figures 53

Figure S1. Stepwise reconstitution of the *dpe* cluster by heterologous expression in *S. cerevisiae* BJ5464-NpgA 53

Figure S2. Transformation of compound **3** to **4** by *A. oryzae* NSAR1 54

Figure S3. Confirmation of the function of DpeE 55

Figure S4. A. oryzae NSAR1 preferentially uses DpeH to transform **5** to **8** 56

Figure S5. Assays with purified, recombinant DpeI.. 57

Figure S6. Biotransformation of compound **8** to **7** with DpeF-producing *S. cerevisiae* or *A. oryzae* strains 58

Figure S7. Quantitative analysis of the production of compounds **1**-**8** in the *S. cerevisiae* or *A. oryzae* systems 59

Figure S8. Product profiles (210 nm) of the *A. oryzae* NSAR1 strains expressing the indicated enzymes and challenged with substrate **6** 60

Figure S9. Amino acid alignment of DpeH and AN7912 61

Figure S10. Product profiles (reversed-phase HPLC traces, 210 nm) of the *A. oryzae* NSAR1 strain fed with substrates **10**-**17** 62

Figure S11. Product profiles (210 nm) of the *A. oryzae* NSAR1 strains expressing M2, and fed with substrates **10**-**17** 63

Figure S12. Product profiles (210 nm) of **9a**, **9b** and synthesized **26**, respectively 64

Figure S13. Chemical structures and key HMBC (→) correlations of the DPEs 65

Figure S14. Standard curves of Compounds **1**-**8** (A-H) 66

Figure S15. UV-VIS spectra of the DEPs and DPEs.. 67

Figure S16. HRESIMS spectra of the DEPs and DPEs 72

Figure S16.1. (-)-HRESIMS spectrum of **2** 72

Figure S16.2. (+)-HRESIMS spectrum of **3** 72

Figure S16.3. (+)-HRESIMS spectrum of **4** 73

Figure S16.4. (+)-HRESIMS spectrum of **5** 73

Figure S16.5. (+)-HRESIMS spectrum of **6** 74

Figure S16.6. (-)-HRESIMS spectrum of **7** 74

Figure S16.7. (+)-HRESIMS spectrum of **8** 75

Figure S16.8. (-)-HRESIMS spectrum of **9a** 76

Figure S16.9. (-)-HRESIMS spectrum of **9b** 76

Figure S16.10. (-)-HRESIMS spectrum of **10** 77

Figure S16.11. (-)-HRESIMS spectrum of **10a** 77

Figure S16.12. (+)-HRESIMS spectrum of **11** 78

Figure S16.13. (-)-HRESIMS spectrum of **11a** 78

Figure S16.14. (-)-HRESIMS spectrum of **11c** 79

Figure S16.15. (-)-HRESIMS spectrum of **12** 80

Figure S16.16. (-)-HRESIMS spectrum of **12a** 80

Figure S16.17. (-)-HRESIMS spectrum of **13** 81

Figure S16.18. (-)-HRESIMS spectrum of **13a** 81

Figure S16.19. (-)-HRESIMS spectrum of **14** 82

Figure S16.20. (-)-HRESIMS spectrum of **14a** 82

Figure S16.21. (-)-HRESIMS spectrum of **15** 83

Figure S16.22. (-)-HRESIMS spectrum of **15a** 83

Figure S16.23. (-)-HRESIMS spectrum of **16** 84

Figure S16.24. (-)-HRESIMS spectrum of **16a** 84

Figure S16.25. (-)-HRESIMS spectrum of **17** 85

Figure S16.26. (-)-HRESIMS spectrum of **17a** 85

Figure S16.27. (-)-HRESIMS spectrum of **17b** 86

Figure S16.28. (+)-HRESIMS spectrum of **18** 87

Figure S16.29. (+)-HRESIMS spectrum of **19** 87

Figure S16.30. (+)-HRESIMS spectrum of **20** 88

Figure S16.31. (+)-HRESIMS spectrum of **21** 88

Figure S16.32. (+)-HRESIMS spectrum of **22** 89

Figure S16.33. (+)-HRESIMS spectrum of **23** 89

Figure S16.34. (+)-HRESIMS spectrum of **24** 90

Figure S16.35. (+)-HRESIMS spectrum of **25** 90

Figure S16.36. (-)-HRESIMS spectrum of **26** 91

Figure S16.37. (+)-HRESIMS spectrum of **27** 91

Figure S17. NMR spectra of the DEPs and DPEs 92

Figure S17.1. ^{1}H NMR spectrum of **2** in methanol-d_4 92

Figure S17.2. ^{13}C NMR spectrum of **2** in methanol-d_4 93

Figure S17.3. ^{1}H NMR spectrum of **3** in methanol-d_4 94

Figure S17.4. ^{13}C NMR spectrum of **3** in methanol-d_4 95

Figure S17.5. ^{1}H NMR spectrum of **4** in methanol-d_4 96
Figure S17.6. ^{13}C NMR spectrum of **4** in methanol-d_4 97
Figure S17.7. ^{1}H NMR spectrum of **5** in acetone-d_6 98
Figure S17.8. ^{13}C NMR spectrum of **5** in acetone-d_6 99
Figure S17.9. ^{1}H NMR spectrum of **6** in methanol-d_4 100
Figure S17.10. ^{13}C NMR spectrum of **6** in methanol-d_4 101
Figure S17.11. HMBC spectrum of **6** in methanol-d_4 102
Figure S17.12. ^{1}H NMR spectrum of **7** in methanol-d_4 103
Figure S17.13. ^{13}C NMR spectrum of **7** in methanol-d_4 104
Figure S17.14. ^{1}H-^{1}H COSY spectrum of **7** in methanol-d_4 105
Figure S17.15. HSQC spectrum of **7** in methanol-d_4 106
Figure S17.16. HMBC spectrum of **7** in methanol-d_4 107
Figure S17.17. ^{1}H NMR spectrum of **8** in methanol-d_4 108
Figure S17.18. ^{13}C NMR spectrum of **8** in methanol-d_4 109
Figure S17.19. ^{1}H-^{1}H COSY spectrum of **8** in methanol-d_4 110
Figure S17.20. HSQC spectrum of **8** in methanol-d_4 111
Figure S17.21. HMBC spectrum of **8** in methanol-d_4 112
Figure S17.22. ^{1}H NMR spectrum of **9a** in DMSO-d_6 113
Figure S17.23. ^{13}C NMR spectrum of **9a** in DMSO-d_6 114
Figure S17.24. ^{1}H-^{1}H COSY spectrum of **9a** in DMSO-d_6 115
Figure S17.25. HSQC spectrum of **9a** in DMSO-d_6 116
Figure S17.26. HMBC spectrum of **9a** in DMSO-d_6 117
Figure S17.27. ^{1}H NMR spectrum of **9b** in DMSO-d_6 118
Figure S17.28. ^{13}C NMR spectrum of **9b** in DMSO-d_6 119
Figure S17.29. ^{1}H-^{1}H COSY spectrum of **9b** in DMSO-d_6 120
Figure S17.30. HSQC spectrum of **9b** in DMSO-d_6 121
Figure S17.31. HMBC spectrum of **9b** in DMSO-d_6 122
Figure S17.32. ^{1}H NMR spectrum of **10** in methanol-d_4 123
Figure S17.33. ^{13}C NMR spectrum of **10** in methanol-d_4 124
Figure S17.34. HSQC spectrum of **10** in methanol-d_4 125

Figure S17.35. HMBC spectrum of **10** in methanol-d_4 126

Figure S17.36. ^{1}H NMR spectrum of **10a** in methanol-d_4 127

Figure S17.37. ^{13}C NMR spectrum of **10a** in methanol-d_4 128

Figure S17.38. HSQC spectrum of **10a** in methanol-d_4 129

Figure S17.39. HMBC spectrum of **10a** in methanol-d_4 130

Figure S17.40. ^{1}H NMR spectrum of **11** in methanol-d_4 131

Figure S17.41. ^{13}C NMR spectrum of **11** in methanol-d_4 132

Figure S17.42. HSQC spectrum of **11** in methanol-d_4 133

Figure S17.43. HMBC spectrum of **11** in methanol-d_4 134

Figure S17.44. ^{1}H NMR spectrum of **11a** in methanol-d_4 135

Figure S17.45. ^{13}C NMR spectrum of **11a** in methanol-d_4 136

Figure S17.46. ^{1}H-^{1}H COSY spectrum of **11a** in methanol-d_4 137

Figure S17.47. HSQC spectrum of **11a** in methanol-d_4 138

Figure S17.48. HMBC spectrum of **11a** in methanol-d_4 139

Figure S17.49. ^{1}H NMR spectrum of **11c** in methanol-d_4 140

Figure S17.50. ^{13}C NMR spectrum of **11c** in methanol-d_4 141

Figure S17.51. DEPT 135 spectrum of **11c** in methanol-d_4 142

Figure S17.52. ^{1}H-^{1}H COSY spectrum of **11c** in methanol-d_4 143

Figure S17.53. HSQC spectrum of **11c** in methanol-d_4 144

Figure S17.54. HMBC spectrum of **11c** in methanol-d_4 145

Figure S17.55. ^{1}H NMR spectrum of **12** in methanol-d_4 146

Figure S17.56. ^{13}C NMR spectrum of **12** in methanol-d_4 147

Figure S17.57. HSQC spectrum of **12** in methanol-d_4 148

Figure S17.58. HMBC spectrum of **12** in methanol-d_4 149

Figure S17.59. ^{1}H NMR spectrum of **12a** in methanol-d_4 150

Figure S17.60. ^{13}C NMR spectrum of **12a** in methanol-d_4 151

Figure S17.61. HSQC spectrum of **12a** in methanol-d_4 152

Figure S17.62. HMBC spectrum of **12a** in methanol-d_4 153

Figure S17.63. ^{1}H NMR spectrum of **13** in methanol-d_4 154

Figure S17.64. ^{13}C NMR spectrum of **13** in methanol-d_4 155

Figure S17.65. HSQC spectrum of **13** in methanol-d_4 156
Figure S17.66. HMBC spectrum of **13** in methanol-d_4 157
Figure S17.67. ^{1}H NMR spectrum of **13a** in methanol-d_4 158
Figure S17.68. ^{13}C NMR spectrum of **13a** in methanol-d_4 159
Figure S17.69. HSQC spectrum of **13a** in methanol-d_4 160
Figure S17.70. HMBC spectrum of **13a** in methanol-d_4 161
Figure S17.71. ^{1}H NMR spectrum of **14** in DMSO-d_6 162
Figure S17.72. ^{13}C NMR spectrum of **14** in DMSO-d_6 163
Figure S17.73. HSQC spectrum of **14** in DMSO-d_6 164
Figure S17.74. HMBC spectrum of **14** in DMSO-d_6 165
Figure S17.75. ^{1}H NMR spectrum of **14a** in DMSO-d_6 166
Figure S17.76. ^{13}C NMR spectrum of **14a** in DMSO-d_6 167
Figure S17.77. HSQC spectrum of **14a** in DMSO-d_6 168
Figure S17.78. HMBC spectrum of **14a** in DMSO-d_6 169
Figure S17.79. ^{1}H NMR spectrum of **15** in methanol-d_4 170
Figure S17.80. ^{13}C NMR spectrum of **15** in methanol-d_4 171
Figure S17.81. HSQC spectrum of **15** in methanol-d_4 172
Figure S17.82. HMBC spectrum of **15** in methanol-d_4 173
Figure S17.83. ^{1}H NMR spectrum of **15a** in methanol-d_4 174
Figure S17.84. ^{13}C NMR spectrum of **15a** in methanol-d_4 175
Figure S17.85. HSQC spectrum of **15a** in methanol-d_4 176
Figure S17.86. HMBC spectrum of **15a** in methanol-d_4 177
Figure S17.87. ^{1}H NMR spectrum of **16** in methanol-d_4 178
Figure S17.88. ^{13}C NMR spectrum of **16** in methanol-d_4 179
Figure S17.89. HSQC spectrum of **16** in methanol-d_4 180
Figure S17.90. HMBC spectrum of **16** in methanol-d_4 181
Figure S17.91. ^{1}H NMR spectrum of **16a** in methanol-d_4 182
Figure S17.92. ^{13}C NMR spectrum of **16a** in methanol-d_4 183
Figure S17.93. HSQC spectrum of **16a** in methanol-d_4 184
Figure S17.94. HMBC spectrum of **16a** in methanol-d_4 185

Figure S17.95. ^{1}H NMR spectrum of **17** in methanol-d_4 186

Figure S17.96. ^{13}C NMR spectrum of **17** in methanol-d_4 187

Figure S17.97. HSQC spectrum of **17** in methanol-d_4 188

Figure S17.98. HMBC spectrum of **17** in methanol-d_4 189

Figure S17.99. ^{1}H NMR spectrum of **17a** in methanol-d_4 190

Figure S17.100. ^{13}C NMR spectrum of **17a** in methanol-d_4 191

Figure S17.101. HSQC spectrum of **17a** in methanol-d_4 192

Figure S17.102. HMBC spectrum of **17a** in methanol-d_4 193

Figure S17.103. ^{1}H NMR spectrum of **17b** in methanol-d_4 194

Figure S17.104. ^{1}H NMR spectrum of **18** in methanol-d_4 195

Figure S17.105. ^{13}C NMR spectrum of **18** in methanol-d_4 196

Figure S17.106. ^{1}H NMR spectrum of **19** in methanol-d_4 197

Figure S17.107. ^{13}C NMR spectrum of **19** in methanol-d_4 198

Figure S17.108. ^{1}H NMR spectrum of **20** in methanol-d_4 199

Figure S17.109. ^{13}C NMR spectrum of **20** in methanol-d_4 200

Figure S17.110. ^{1}H NMR spectrum of **21** in methanol-d_4 201

Figure S17.111. ^{13}C NMR spectrum of **21** in methanol-d_4 202

Figure S17.112. ^{1}H NMR spectrum of **22** in methanol-d_4 203

Figure S17.113. ^{13}C NMR spectrum of **22** in methanol-d_4 204

Figure S17.114. ^{1}H NMR spectrum of **23** in methanol-d_4 205

Figure S17.115. ^{13}C NMR spectrum of **23** in methanol-d_4 206

Figure S17.116. ^{1}H NMR spectrum of **24** in methanol-d_4 207

Figure S17.117. ^{13}C NMR spectrum of **24** in methanol-d_4 208

Figure S17.118. ^{1}H NMR spectrum of **25** in methanol-d_4 209

Figure S17.119. ^{13}C NMR spectrum of **25** in methanol-d_4 210

Figure S17.120. ^{1}H NMR spectrum of **26** in DMSO-d_6 211

Figure S17.121. ^{13}C NMR spectrum of **26** in DMSO-d_6 212

Figure S17.122. ^{1}H NMR spectrum of **27** in DMSO-d_6 213

Figure S17.123. ^{13}C NMR spectrum of **27** in DMSO-d_6 214

References 215

1 SI Materials and Methods

1.1 Molecular biology, microbiology and bioinformatics

1.1.1 Construction of *Saccharomyces cerevisiae* expression vectors

The primers used in this study are listed in **Table S8**. The plasmids are summarized in **Table S1**.

The YEpADH2p-FLAG-URA plasmid harboring the encoding gene for Preu6 (DpeA), YEpPreu6, was constructed as previously described.[1-2] YEpADH2p-FLAG-TRP plasmids carrying the encoding genes for DpeB, DpeC, DpeD, DpeE, DpeF, DpeH, and DpeI, respectively, were constructed as follows.[1] The sequences of the open reading frames of these genes were confirmed first by transcriptome sequencing of *Preussia isomera* XL1326 grown under DPE-producing conditions. Next, using cDNA of *Preussia isomera* XL1326 as the template, a 1,617-bp fragment (DpeB-06F), a 825-bp fragment (DpeC-06F), a 999-bp fragment (DpeD-06F), a 942-bp fragment (DpeE-06F), a 1,356-bp fragment (DpeF-06F), a 1,221-bp fragment (DpeH-06F), and a 1,101-bp fragment (DpeI-06F) were amplified with primer pairs 06F-DpeB-F/06F-DpeB-R, 06F-DpeC-F/06F-DpeC-R, 06F-DpeD-F/06F-DpeD-R, 06F-DpeE-F/06F-DpeE-R, 06F-DpeF-F/06F-DpeF-R, 06F-DpeH-F/06F-DpeH-R, and 06F-DpeI-F/06F-DpeI-R, respectively. Then, DpeB-06F, or DpeC-06F, or DpeD-06F, or DpeE-06F, or DpeF-06F, or DpeH-06F, or DpeI-06F, which contain appropriate overlapping segments with the expression vector, were merged with the *Nde*I/*Pme*I fragment of YEpADH2p-FLAG-TRP (6,144-bp) using the SE Seamless Cloning and Assembly Kit (ZOMANBIO, Beijing, China) to yield plasmid YEpTRP(DpeB), YEpTRP(DpeC), YEpTRP(DpeD), YEpTRP(DpeE), YEpTRP(DpeF), YEpTRP(DpeH), or YEpTRP(DpeI), respectively.

YEpADH2p-FLAG-LEU plasmids carrying *dpeB* or *dpeD* with *Xho*I site mutations, respectively, were constructed as follows.[1] Using YEpTRP(DpeB) as the template, a 1,197-bp fragment (DpeB-ΔXhoI-1), and a 448-bp fragment (DpeB-ΔXhoI-2) were amplified with primer pairs 06F-DpeB-F/DpeB-ΔXhoI-R, and DpeB-ΔXhoI-F/06F-DpeB-R, respectively. Using YEpTRP(DpeD) as the template, a 486-bp fragment (DpeD-ΔXhoI-1), and a 540-bp fragment (DpeD-ΔXhoI-2) were amplified with primer pairs 06F-DpeD-F/DpeD-ΔXhoI-R, and DpeD-ΔXhoI-F/06F-DpeD-R, respectively. DpeB-ΔXhoI-1 and DpeB-ΔXhoI-2, or DpeD-ΔXhoI-1 and DpeD-ΔXhoI-2, which contain appropriate overlapping segments, were merged with the *Nde*I/*Pme*I fragment of YEpADH2p-FLAG-LEU (7,573-bp) using the SE Seamless Cloning and Assembly Kit to yield plasmid YEpLEU(DpeB-ΔXhoI) or YEpLEU(DpeD-ΔXhoI), respectively. Then, the YEpADH2p-FLAG-LEU plasmid that contains the encoding genes for both DpeB and DpeD, YEpLEU(DpeB+D), was constructed by ligating the 1,889-

bp *Bgl*II-*Sal*I restriction fragment of YEpLEU(DpeD-ΔXhoI) into the *Bam*HI-*Xho*I-digested YEpLEU(DpeB-ΔXhoI) (8,973-bp).

For construction of YEpLEU(DpeB+D+C), the YEpADH2p-FLAG-LEU plasmid containing the encoding genes for DpeB, DpeD and DpeC, YEpLEU(DpeC) was constructed first by ligating the 770-bp *Nde*I-*Pme*I restriction fragment of YEpTRP(DpeC) into the *Nde*I-*Pme*I-digested YEpADH2p-FLAG-LEU (7,573-bp). Then, the 1,715-bp *Bgl*II-*Sal*I restriction fragment of YEpLEU(DpeC) was ligated into the *Bam*HI-*Xho*I-digested YEpLEU(DpeB+D) (10,700-bp) to make the construct YEpLEU(DpeB+D+C).

For construction of YEpLEU(DpeB+D+C+E), the YEpADH2p-FLAG-LEU plasmid carrying the encoding genes for DpeB, DpeD, DpeC and DpeE, YEpLEU[(DpeB+D+C)-ΔBamHI] and YEpLEU(DpeE-ΔBglII) had to be constructed first. Using YEpLEU(DpeB+D+C) as the template, a 1,524-bp fragment [(DpeB+D+C)-ΔBamHI-1], and a 426-bp fragment [(DpeB+D+C)-ΔBamHI-2] were amplified with primer pairs DpeBDC-BlpI-F/DpeBDC-ΔBamHI-R, and DpeBDC-ΔBamHI-F/DpeBDC-XhoI-R, respectively. Next, (DpeB+D+C)-ΔBamHI-1 and (DpeB+D+C)-ΔBamHI-2, which contain appropriate overlapping segments, were merged with the *Blp*I/*Xho*I fragment of YEpLEU(DpeB+D+C) (10,559-bp) using the SE Seamless Cloning and Assembly Kit to yield plasmid YEpLEU[(DpeB+D+C)-ΔBamHI]. Using YEpTRP(DpeE) as the template, a 852-bp fragment (DpeE-ΔBglII-1), and a 117-bp fragment (DpeE-ΔBglII-2) were amplified with primer pairs 06F-DpeE-F/DpeE-ΔBglII-R, and DpeE-ΔBglII-F/06F-DpeE-R, respectively. Next, DpeE-ΔBglII-1 and DpeE-ΔBglII-2, which contain appropriate overlapping segments, were merged with the *Nde*I/*Pme*I fragment of YEpADH2p-FLAG-LEU (7,573-bp) using the SE Seamless Cloning and Assembly Kit to yield plasmid YEpLEU(DpeE-ΔBglII). And then, the 1,832-bp *Bgl*II-*Sal*I restriction fragment of YEpLEU(DpeE-ΔBglII) was ligated into the *Bam*HI-*Xho*I-digested YEpLEU[(DpeB+D+C)-ΔBamHI] (12,253-bp) to make the construct YEpLEU(DpeB+D+C+E).

For construction of YEpLEU(DpeB+D+C+E+F), the YEpADH2p-FLAG-LEU plasmid carrying the encoding genes for DpeB, DpeD, DpeC, DpeE and DpeF, YEpLEU[(DpeB+D+C+E)-ΔBamHI] and YEpLEU(DpeF-ΔBglII,SalI) had to be constructed first. Using YEpLEU(DpeB+D+C+E) as the template, a 700-bp fragment [(DpeB+D+C+E)-ΔBamHI] was amplified with primer pairs DpeBDCE-PstI-F/DpeBDC-XhoI-R. Next, (DpeB+D+C+E)-ΔBamHI, which contains appropriate overlapping segments, were merged with the *Pst*I/*Xho*I fragment of YEpLEU(DpeB+D+C+E) (13,455-bp) using the SE Seamless Cloning and Assembly Kit to yield plasmid YEpLEU[(DpeB+D+C+E)-ΔBamHI]. Using

YEpTRP(DpeF) as the template, a 225-bp fragment (DpeF-ΔBglII,SalI-1), a 556-bp fragment (DpeF-ΔBglII,SalI-2), a 505-bp fragment (DpeF-ΔBglII,SalI-3), and a 145-bp fragment (DpeF-ΔBglII,SalI-4) were amplified with primer pairs 06F-DpeF-F/DpeF-ΔBglII-1R, DpeF-ΔBglII-1F/DpeF-ΔSalI-R, DpeF-ΔSalI-F/DpeF-ΔBglII-2R and DpeF-ΔBglII-2F/06F-DpeF-R, respectively. Next, DpeF-ΔBglII,SalI-1, DpeF-ΔBglII,SalI-2, DpeF-ΔBglII,SalI-3 and DpeF-ΔBglII,SalI-4, which contain appropriate overlapping segments, were merged with the *Nde*I/*Pme*I fragment of YEpADH2p-FLAG-LEU (7,573-bp) using the SE Seamless Cloning and Assembly Kit to yield plasmid YEpLEU(DpeF-ΔBglII,SalI). Then, the 2,246-bp *Bgl*II-*Sal*I restriction fragment of YEpLEU(DpeF-ΔBglII,SalI) was ligated into the *Bam*HI-*Xho*I-digested YEpLEU[(DpeB+D+C+E)-ΔBamHI] (13,923-bp) to make the construct YEpLEU(DpeB+D+C+E+F).

For construction of YEpLEU(DpeB+D+C+E+F+H), the YEpADH2p-FLAG-LEU plasmid carrying the encoding genes for DpeB, DpeD, DpeC, DpeE, DpeF and DpeH, YEpLEU(DpeH-ΔBglII) had to be constructed first. Using YEpTRP(DpeH) as the template, a 231-bp fragment (DpeH-ΔBglII-1), and a 1,017-bp fragment (DpeH-ΔBglII-2) were amplified with primer pairs 06F-DpeH-F/DpeH-ΔBglII-R and DpeH-ΔBglII-F/06F-DpeH-R, respectively. Next, DpeH-ΔBglII-1 and DpeH-ΔBglII-2, which contain appropriate overlapping segments, were merged with the *Nde*I/*Pme*I fragment of YEpADH2p-FLAG-LEU (7,573-bp) using the SE Seamless Cloning and Assembly Kit to yield plasmid YEpLEU(DpeH-ΔBglII). Then, the 2,111-bp *Bgl*II-*Sal*I restriction fragment of YEpLEU(DpeH-ΔBglII) was ligated into the *Bam*HI-*Xho*I-digested YEpLEU(DpeB+D+C+E+F) (16,007-bp) to make the construct YEpLEU(DpeB+D+C+E+F+H).

All newly constructed plasmids were confirmed by DNA sequencing.

1.1.2 Construction of *Aspergillus oryzae* expression vectors

The primers used in this study are listed in **Table S8**. The plasmids are summarized in **Table S1**.

The pUSA plasmid harboring the encoding gene for DpeA, pUSA(DpeA), was constructed as follows.[3] Using YEpPreu6 as the template, a 1,269-bp fragment (DpeA-pUSA-1), and a 1,950-bp fragment (DpeA-pUSA-3) were amplified with primer pairs pUSA-DpeA-1F/pUSA-DpeA-1R and pUSA-DpeA-2F/pUSA-DpeA-2R, respectively. Next, a 3,551-bp fragment, DpeA-pUSA-2, was generated by digesting YEpPreu6 with *Bgl*II/*Sex*AI. DpeA-pUSA-1, DpeA-pUSA-2 and DpeA-pUSA-3, which contain appropriate overlapping segments, were merged with the *Kpn*I fragment of pUSA (7,133-bp) using the SE Seamless Cloning and Assembly Kit to yield plasmid pUSA(DpeA).

pTAex3 plasmids carrying the encoding genes for DpeB, DpeC, DpeD, DpeE, DpeF, DpeH, and DpeI, respectively, were constructed as follows.[3] Using YEpTRP(DpeB), YEpTRP(DpeC), YEpTRP(DpeD), YEpTRP(DpeE), YEpTRP(DpeF), YEpTRP(DpeH), or YEpTRP(DpeI), respectively, as the template, a 1,597-bp fragment (DpeB-pTAex3), a 805-bp fragment (DpeC-pTAex3), a 979-bp fragment (DpeD-pTAex3), a 922-bp fragment (DpeE-pTAex3), a 1,336-bp fragment (DpeF-pTAex3), a 1,201-bp fragment (DpeH-pTAex3), and a 1,081-bp fragment (DpeI-pTAex3) were amplified with primer pairs pTAex3-DpeB-F/pTAex3-DpeB-R, pTAex3-DpeC-F/pTAex3-DpeC-R, pTAex3-DpeD-F/pTAex3-DpeD-R, pTAex3-DpeE-F/pTAex3-DpeE-R, pTAex3-DpeF-F/pTAex3-DpeF-R, pTAex3-DpeH-F/pTAex3-DpeH-R, and pTAex3-DpeI-F/pTAex3-DpeI-R, respectively. Then, DpeB-pTAex3, or DpeC-pTAex3, or DpeD-pTAex3, or DpeE-pTAex3, or DpeF-pTAex3, or DpeH-pTAex3, or DpeI-pTAex3, which contain appropriate overlapping segments, were merged with the *Kpn*I fragment of pTAex3 (7,595-bp) using the SE Seamless Cloning and Assembly Kit to yield plasmid pTAex3(DpeB), pTAex3(DpeC), pTAex3(DpeD), pTAex3(DpeE), pTAex3(DpeF), pTAex3(DpeH), or pTAex3(DpeI), respectively.

The pTAex3 plasmid containing the encoding genes for both DpeE and DpeF, was constructed as follows. Using pTAex3(DpeF) as the template, a 2,208-bp fragment (PamyB-DpeF-TamyB) was amplified with primer pairs PamyB-DpeF-TamyB-F/PamyB-DpeF-TamyB-R, and ligated into pJET1.2 (Thermo Scientific, Vilnius, Lithuania) to make the construct pJET1.2(DpeF). Then, the 2,199-bp *Bst*EII restriction fragment of pJET1.2(DpeF) was ligated into the *Bst*EII-digested pTAex3(DpeE) (8,481-bp) to make the construct pTAex3(DpeE+F).

The pUSA plasmid containing the encoding genes for both DpeA and DpeC, was constructed as follows. Using pUSA(DpeA) as the template, a 436-bp fragment (DpeA+C-1), and a 1,793-bp fragment (DpeA+C-3) were amplified with primer pairs pUSA-DpeA-NdeI-F/pUSA-DpeA-TAA-R and pUSA-DpeA-sC-F/pUSA-DpeA-FspAI-R, respectively. Next, a 1,731-bp fragment, DpeA+C-2, was amplified with primers PamyB-DpeC-TamyB-F/PamyB-DpeC-TamyB-R using pTAex3(DpeC) as the template. DpeA+C-1, DpeA+C-2, and DpeA+C-3, which contain appropriate overlapping segments, were merged with pJET1.2 using the SE Seamless Cloning and Assembly Kit to yield plasmid pJET1.2(DpeC). Then, the 3,761-bp *Nde*I-*Fsp*AI restriction fragment of pJET1.2(DpeC) was ligated into the *Nde*I-*Fsp*AI-digested pUSA(DpeA) (11,657-bp) to make the construct pUSA(DpeA+C).

The pAdeA plasmid carrying the encoding gene for DpeB was constructed as follows.[3] Using pTAex3(DpeB) as the template, a 2,513-bp fragment (PamyB-DpeB-TamyB) was amplified with primer pairs PamyB-DpeB-TamyB-F/PamyB-DpeB-TamyB-R, and then was merged with the *Xba*I fragment of pAdeA (4,892-bp) using the SE Seamless Cloning and Assembly Kit to yield plasmid pAdeA(DpeB).

The pAdeA plasmid containing the encoding genes for both DpeB and DpeD, was constructed as follows. Using pTAex3(DpeD) as the template, an 1,853-bp fragment (PamyB-DpeD-TamyB) was amplified with primer pairs PamyB-DpeD-TamyB-F/PamyB-DpeD-TamyB-R, and ligated into pJET1.2 to make the construct pJET1.2(DpeD). Then, the 1,843-bp *Spe*I restriction fragment of pJET1.2(DpeD) was ligated into the *Spe*I-digested pAdeA(DpeB) (7,346-bp) to make the construct pAdeA(DpeB+D).

pUNA plasmids carrying the encoding genes for DpeC and DpeH, respectively, were constructed as follows.[3] Using pTAex3(DpeC) or pTAex3(DpeH), as the template, an 826-bp fragment (DpeC-pUNA), and a 1,222-bp fragment (DpeH-pUNA) were amplified with primer pairs pUNA-DpeC-F/pUNA-DpeC-R, and pUNA-DpeH-F/pUNA-DpeH-R, respectively. Then, DpeC-pUNA or DpeH-pUNA, which contain appropriate overlapping segments, were merged with the *Sma*I fragment of pUNA (9,090-bp) using the SE Seamless Cloning and Assembly Kit to yield plasmid pUNA(DpeC) or pUNA(DpeH), respectively.

The pUNA plasmid containing the encoding genes for both DpeH and DpeI, was constructed as follows. Using pTAex3(DpeI) as the template, a 1,957-bp fragment (PamyB-DpeI-TamyB) was amplified with primer pairs PamyB-DpeI-TamyB-F/PamyB-DpeI-TamyB-R, and ligated into pJET1.2 to make the construct pJET1.2(DpeI). Then, the 1,943-bp *Xba*I restriction fragment of pJET1.2(DpeI) was ligated into the *Xba*I-digested pUNA(DpeH) (10,251-bp) to make the construct pUNA(DpeH+I).

The pTAex3 plasmid carrying the encoding gene for AN7912 was constructed as follows. Using the synthesized *AN7912* cDNA fragment as the template, a 1,169-bp fragment (AN7912-pTAex3) was amplified with primer pairs pTAex3-AN7912-F/pTAex3-AN7912-R, and then was merged with the *Kpn*I fragment of pTAex3 (7,595-bp) using the SE Seamless Cloning and Assembly Kit to yield plasmid pTAex3(AN7912).

The pTAex3 plasmid carrying chimeric M1 (DpeH[M^1-K^{59}]+AN7912[E^{59}-P^{369}]) was constructed by replacing the gene segment encoding M^1-R^{58} of AN7912 with that encoding M^1-K^{59} of DpeH. A 227-bp fragment, DpeH(M^1-K^{59}), was amplified with primers pTAex3-DpeH-F/DpeHK59-R using pTAex3(DpeH) as the template. Next, a 966-bp fragment, AN7912(E^{59}-P^{369}), was amplified with primers

AN7912E59-F/pTAex3-AN7912-R using pTAex3(AN7912) as the template. DpeH(M^1-K^{59}) and AN7912(E^{59}-P^{369}), which contain appropriate overlapping segments, were merged with the *Kpn*I fragment of pTAex3(7,595-bp) using the SE Seamless Cloning and Assembly Kit to yield plasmid pTAex3(M1).

The pTAex3 plasmid carrying chimeric M2 (AN7912[M^1-L^{343}]+DpeH[L^{346}-Q^{386}]) was constructed by replacing the gene segment encoding L^{344}-P^{369} of AN7912 with that encoding L^{346}-Q^{386} of DpeH. A 1,058-bp fragment, AN7912(M^1-L^{343}), was amplified with primers pTAex3-AN7912-F/AN7912L343-R using pTAex3(AN7912) as the template. Next, a 176-bp fragment, DpeH(L^{346}-Q^{386}), was amplified with primers DpeHL346-F/pTAex3-DpeH-R using pTAex3(DpeH) as the template. AN7912(M^1-L^{343}) and DpeH(L^{346}-Q^{386}), which contain appropriate overlapping segments, were merged with the *Kpn*I fragment of pTAex3(7,595-bp) using the SE Seamless Cloning and Assembly Kit to yield plasmid pTAex3(M2).

The pTAex3 plasmid carrying chimeric M3 (DpeH[M^1-K^{59}]+AN7912[E^{59}-L^{343}]+DpeH[L^{346}-Q^{386}]) was constructed by replacing the gene segments encoding M^1-R^{58} and L^{344}-P^{369} of AN7912 with the corresponding ones encoding M^1-K^{59} or L^{346}-Q^{386} of DpeH, respectively. A 1,052-bp fragment, [DpeH(M^1-K^{59})+AN7912(E^{59}-L^{343})], was amplified with primers pTAex3-DpeH-F/AN7912L343-R using pTAex3(M1) as the template. Then, [DpeH(M^1-K^{59})+AN7912(E^{59}-L^{343})] and DpeH(L^{346}-Q^{386}), which contain appropriate overlapping segments, were merged with the *Kpn*I fragment of pTAex3(7,595-bp) using the SE Seamless Cloning and Assembly Kit to yield plasmid pTAex3(M3).

The pTAex3 plasmid carrying chimeric M4 (DpeH[M^1-L^{345}]+AN7912[L^{344}-P^{369}]) was constructed by replacing the gene segment encoding L^{346}-Q^{386} of DpeH with that encoding L^{344}-P^{369} of AN7912. A 1,055-bp fragment, DpeH(M^1-L^{345}), was amplified with primers pTAex3-DpeH-F/DpeHL345-R using pTAex3(DpeH) as the template. Next, a 141-bp fragment, AN7912(L^{344}-P^{369}), was amplified with primers AN7912L344-F/pTAex3-AN7912-R using pTAex3(AN7912) as the template. DpeH(M^1-L^{345}) and AN7912(L^{344}-P^{369}), which contain appropriate overlapping segments, were merged with the *Kpn*I fragment of pTAex3(7,595-bp) using the SE Seamless Cloning and Assembly Kit to yield plasmid pTAex3(M4).

pTAex3 plasmids carrying the encoding gene segments for M5 (DpeH[M^1-L^{345}]) and M6 (AN7912[M^1-L^{343}]), respectively, were constructed as follows. Using pTAex3(DpeH) or pTAex3(AN7912), as the template, a 1,088-bp fragment [DpeH(M^1-L^{345})-2], and a 1,091-bp fragment [AN7912(M^1-L^{343})-2] were amplified with primer pairs pTAex3-DpeH-F/DpeHL345-R2, and pTAex3-

AN7912-F/AN7912L343-R2, respectively. Then, DpeH(M^1-L^{345})-2 or AN7912(M^1-L^{343})-2, which contain appropriate overlapping segments, were merged with the *Kpn*I fragment of pTAex3(7,595-bp) using the SE Seamless Cloning and Assembly Kit to yield plasmid pTAex3(M5) or pTAex3(M6), respectively.

The pTAex3 plasmid carrying chimeric M7 (M2-Δ[V^{368}-M^{372}]) was constructed by deleting the gene segment encoding V^{368}-M^{372} of M2. Using pTAex3(DpeH) as the template, a 102-bp fragment DpeH(L^{346}-P^{369}), and an 89-bp fragment DpeH(S^{375}-Q^{386}), were amplified with primer pairs DpeHL346-F/DpeHP369-R, DpeHS375-F/pTAex3-DpeH-R, respectively. AN7912(M^1-L^{343}), DpeH(L^{346}-P^{369}), DpeH(S^{375}-Q^{386}), which contain appropriate overlapping segments, were merged with the *Kpn*I fragment of pTAex3(7,595-bp) using the SE Seamless Cloning and Assembly Kit to yield plasmid pTAex3(M7).

pTAex3 plasmids carrying chimeric M8 (M2-Δ[Y^{381}-Q^{384}]), M9 (M2-Δ[P^{374}-Q^{384}]), and M10 (M2-Δ[V^{368}-Q^{384}]) were constructed by deleting the gene segments encoding Y^{381}-Q^{384}, P^{374}-Q^{384}, and V^{368}-Q^{384} of M2, respectively. Using pTAex3(M2) as the template, a 1,202-bp fragment [M2-Δ(Y^{381}-Q^{384})], a 1,181-bp fragment [M2-Δ(P^{374}-Q^{384})], and a 1,163-bp fragment [M2-Δ(V^{368}-Q^{384})] were amplified with primer pairs pTAex3-AN7912-F/pTAex3-DpeH-R2, pTAex3-AN7912-F/pTAex3-DpeH-R3, and pTAex3-AN7912-F/pTAex3-DpeH-R4, respectively. Then, [M2-Δ(Y^{381}-Q^{384})], [M2-Δ(P^{374}-Q^{384})] or [M2-Δ(V^{368}-Q^{384})], which contain appropriate overlapping segments, were merged with the *Kpn*I fragment of pTAex3(7,595-bp) using the SE Seamless Cloning and Assembly Kit to yield plasmid pTAex3(M8), pTAex3(M9) or pTAex3(M10), respectively.

All newly constructed plasmids were confirmed by DNA sequencing.

1.1.3 Total biosynthesis of the depsidones and diphenyl ethers

1.1.3.1 Total biosynthesis in *S. cerevisiae*

For total biosynthesis, the appropriate expression plasmids were transformed into cells of *S. cerevisiae* BJ5464-NpgA (*MATα*, *ura3-52*, *his3-Δ200*, *leu2-Δ1*, *trp1*, *pep4*::*HIS3*, *prb1-Δ1.6R*, *can1*, *GAL*) using the LiAc-PEG4000 procedure,[4,5] respectively, and the transformants were selected on the appropriate synthetic drop-out (SD) media plates.[2] Three to five independent *S. cerevisiae* transformants were tested for the production of depsidones (DEPs) or/and diphenyl ethers (DPEs), and fermentations with representative isolates were repeated at least three times. Recombinant yeast cells were grown in 150 mL Erlenmeyer flasks containing 25 mL of the appropriate SD medium at 30ºC with shaking at 200 rpm until the OD_{600nm} reached 0.6. Then, an equal volume of YPD medium (1% yeast extract, 2% peptone,

and 1% dextrose) was added to the culture, and the fermentation was continued under the same conditions for an additional 5 days. Fermentations were scaled up for the isolation of DEPs or DPEs to two to ten liters, depending on yield.

1.1.3.2 Total biosynthesis in *A. oryzae*

The appropriate expression plasmids were transformed into *A. oryzae* NSAR1 (*niaD*$^-$, *sC*$^-$, Δ*argB*, *adeA*$^-$) by the protoplast-polyethylene glycol method, respectively, and the transformants were selected on the appropriate nutrient deficient media plates.[3,6] For each transformation, at least twelve independent *A. oryzae* transformants were tested for the production of DEPs or/and DPEs, and fermentations with representative isolates were repeated at least three times. The selected transformants were grown in 500 mL Erlenmeyer flasks containing 150 mL of DPY medium (2% dextrin, 1% polypeptone, 2.5% yeast extract, 0.5% KH_2PO_4, and 0.05% $MgSO_4 \cdot 7H_2O$) at 30ºC with shaking at 160 rpm for 4 days. The equivalent Czapek-Dox (CD) medium with hipolypepton (10 g/L) and starch (20 g/L) was then added to induce expression under the *α*-amylase promoter, and the cultures were shaken for further 4 days. Fermentations were scaled up for the isolation of the DEPs or the DPEs to two to ten liters, depending on yield.

1.1.4 Quantitative analysis of the formation of compounds 1-8

1.1.4.1 Standard curve

For the standard curves, compounds **1**-**8** were accurately weighed and dissolved in 1 mL of methanol to obtain stock solutions. For compounds **1**, **2**, **4**-**6**, and **8**, the concentrations of the stock solutions were 0.5 mg/mL, 1.0 mg/mL, 1.5 mg/mL, 2.0 mg/mL, and 2.5 mg/mL; for compounds **3** and **7**, the concentrations of the stock solutions were 1.0 mg/mL, 2.0 mg/mL, 3.0 mg/mL, 4.0 mg/mL, and 5.0 mg/mL. All standard solutions were stored in a refrigerator at 4ºC until analysis. Every stock solution was analyzed by UHPLC to record the peak area (DAD, 210 nm) following the method described in **1.2.1**, with the injection volume set to 2 μL. Three technical replicates for each stock solution were performed.[2]

1.1.4.2 Quantitative analysis

For quantitative experiments, five independent *S. cerevisiae* or *A. oryzae* transformants were cultivated twice (n=10), and the peak area of the corresponding DEPs and the DPEs were measured. Fermentation and extraction processes followed the protocols in **1.1.3.1** and **1.2.1**, respectively. The

extracts were reconstituted in methanol and analyzed by UHPLC to record the peak area (DAD, 210 nm) following the method described in **1.2.1**, and the injection volume was set to 2 μL.

1.1.5 Protein expression, purification, and *in vitro* biochemical assay

1.1.5.1 Protein expression and purification

The genes encoding DpeE and DpeI were amplified with primer pairs 28a-DpeE-F/28a-DpeE-R and 28a-DpeI-F/28a-DpeI-R using cDNA of *Preussia isomera* XL1326 as template, and inserted into the multiple cloning site of the pET-28a vector (ZOMANBIO, **Table S1**) as *Nde*I-*Xho*I fragments, 28a-DpeE and 28a-DpeI, respectively (**Table S8**). The resulting plasmids were transformed into *Escherichia coli* BL21-CodonPlus (DE3)-RIPL (ZOMANBIO), respectively. The expression and purification of the proteins followed the previously described methods.[2]

1.1.5.2 *In vitro* biochemical assays

The DpeE reaction mixture (100 μL) contained 10 μM enzyme and 500 μM compound **4**, or additional 1 mM *S*-adenosyl methionine (SAM), respectively, in buffer C (50 mM Tris, 50 mM NaCl, 5% glycerol, pH 7.5).[2,7] The reaction mixture was incubated at 28 ºC for 2h. Then, the reaction was quenched by addition of an equal volume of methanol, and the reaction products were analyzed by UHPLC (**Fig. S3**).

The DpeI reaction mixture (100 μL) contained 10 μM enzyme and 200 μM of any of compounds **1**-**8**, respectively, in buffer C.[2] The reaction mixture was incubated at 25 ºC for 6h. The following steps were the same as DpeE assays (**Fig. S5**).

1.1.6 Comparative genome analysis

To explore the distribution of the DPE biosynthetic gene clusters in fungi, 46 species described to produce DPEs were selected through literature review (**Table S2**). The genome sequences of the selected fungi, if available, were obtained from the National Center for Biotechnology Information (NCBI) or the Joint Genome Institute (JGI) MycoCosm databases. These genome sequences were submitted to antiSMASH 7.0 (https://fungismash.secondarymetabolites.org/#!/start) to identify and characterize putative DPE-related biosynthetic gene clusters. Proteins homologous to the known enzymes of voucher DPE biosynthetic gene clusters (i.e., the *ors*, *pta*, or *dpe* clusters), were analyzed using FGENESH (http://linux1.softberry.com/berry.phtml) combined with BLASTP (https://blast.ncbi.nlm.nih.gov/Blast.cgi) (>80% sequence coverage and >30% amino acid sequence identity).

1.2 Isolation and characterization of the DEPs and DPEs

1.2.1 General methods

At the end of the fermentations, *S. cerevisiae* BJ5464-NpgA cultures were adjusted to pH 5.0 and extracted with equal volumes of ethyl acetate; *A. oryzae* NSAR1 cultures (300 mL/flask) were extracted with 200 mL ethyl acetate with shaking at 200 rpm for 0.5 h. Both *S. cerevisiae* BJ5464-NpgA and *A. oryzae* NSAR1 cultures were extracted three times each. The crude extracts were pooled, dried *in vacuo*, and reconstituted in methanol. Samples were routinely analyzed on a Thermo Scientific UltiMate 3000 UHPLC system, fitted with a Sunniest PFP & C18 column (250 × 4.6 mm, 5.0 μm, ChromaNik Technologies). The mobile phases consisted of water (A) and methanol (B, with 0.1% acetic acid), and the flow rate was kept at 1.0 mL/min. The system was run with the following gradient program: from 30% B to 100% B for 40 min, then kept at 100% B for 10 min. The column was balanced with 30% B for 4 min before each sample injection. Absorbance was monitored with an UltiMate 3000 DAD Detector at 210, 254, 270 and 300 nm wavelength.

For isolation of the DEPs and DPEs, the crude extracts were first subjected to ODS column chromatography eluted with a gradient of methanol/water. The resulting fractions containing the targets were subsequently purified by semi-preparative HPLC on an Agilent 1260 Infinity II system. UHPLC-HRESIMS spectra were acquired on an Thermo Scientific UltiMate 3000 UHPLC coupled with an Thermo Scientific Q Exactive instrument operated in positive and negative ion mode using full scanning of 150-1000 *m/z*; capillary voltage of 3.8 kV; capillary temperature of 325 ºC; sheath gas flow velocity of 40 arb; auxiliary gas velocity of 20 arb; auxiliary gas heating temperature of 350 ºC. ^{1}H, ^{13}C, and 2D NMR, including proton-proton correlation spectroscopy (^{1}H-^{1}H COSY), heteronuclear single quantum coherence (HSQC), and heteronuclear multiple bond correlation (HMBC) spectra were recorded on a Bruker Avance III^{TM} HD 500 or 600 MHz spectrometer in CD_3OD, DMSO, or acetone as the solvent. Chemical shift values (δ) are given in parts per million (ppm), and the coupling constants (J values) are in Hz. Chemical shifts were referenced to the residual solvent peaks of methanol-d_4, DMSO-d_6 or acetone-d_6. Unless otherwise stated, chemicals and solvents were of reagent grade and used as obtained from commercial sources.

1.2.2 Compound 2

The crude extract (0.83 g) of a 2 L fermentation broth of *S. cerevisiae* BJ5464-NpgA carrying plasmids YEpPreu6 + YEpTRP(DpeB) was applied to an ODS column, eluting with a gradient of

methanol/water (20:80, 40:60, 60:40) to yield fractions A-C. After UHPLC-MS analysis, fraction C was further purified by semi-preparative HPLC, eluting with MeOH/H_2O (0-16 min, 70:30, v/v) to yield compound **2** (49.7 mg, t_R=8.0 min).

2: Corynesidone D. Grey amorphous powder; maximum UV absorption wavelength 316 nm (**Fig. S15**); ^{1}H NMR (500 MHz, methanol-d_4): δ 6.60 (s, 1H), 6.58 (br d, 2H), 2.66 (s, 3H), 2.40 (s, 3H); ^{13}C NMR (125 MHz, methnol-d_4): δ 173.6, 164.6, 164.2, 164.0, 160.6, 149.8, 146.9, 143.3, 134.5, 117.1, 113.6, 113.2, 107.3, 105.9, 21.4, 15.0 (**Fig. S17.1, 17.2**); HRESI-MS: *m/z* 315.05255 [M-H]$^-$ (calcd for $C_{16}H_{11}O_7$, 315.0505) (**Fig. S16.1**).[8,9]

1.2.3 Compound 3

The crude extract (1.33 g) of a 3 L fermentation broth of *S. cerevisiae* BJ5464-NpgA carrying plasmids YEpPreu6 + YEpLEU(DpeB) + YEpTRP(DpeD) was applied to an ODS column, eluting with a gradient of methanol/water (20:80, 40:60, 60:40) to yield fractions A-C. After UHPLC-MS analysis, fraction C was further purified by semi-preparative HPLC, eluting with MeOH/H_2O (from 30% to 90% MeOH for 25 min, from 90% to 30% MeOH for 1 min, then kept at 30% MeOH for 4 min) to yield compound **3** (75.6 mg, t_R=20.1 min).

3: Corynesidone A. White solid; maximum UV absorption wavelength 268 nm (**Fig. S15**); ^{1}H NMR (600 MHz, methanol-d_4): δ 6.56 (br s, 1H), 6.53 (d, J = 2.3 Hz, 1H), 6.46 (d, J = 2.8 Hz, 1H), 6.44 (d, J = 2.8 Hz, 1H), 2.39 (s, 3H), 2.36 (s, 3H); ^{13}C NMR (150 MHz, methanol-d_4): δ 165.3, 165.0, 163.6, 155.8, 146.6, 146.1, 143.3, 132.5, 116.6, 114.8, 113.3, 105.8, 105.7, 21.3, 16.0 (**Fig. S17.3, 17.4**); HRESI-MS: *m/z* 273.07559 [M+H]$^+$ (calcd for $C_{15}H_{13}O_5$, 273.0763) (**Fig. S16.2**).[8,10]

1.2.4 Compound 4

The crude extract (0.73 g) of a 3 L fermentation broth of *S. cerevisiae* BJ5464-NpgA carrying plasmids YEpPreu6 + YEpLEU(DpeB+D) + YEpTRP(DpeC) was applied to an ODS column, eluting with a gradient of methanol/water (20:80, 25:75, 30:70, 35:65, 40:60, 45:55) to yield fractions A-F. After UHPLC-MS analysis, fraction B was further purified by semi-preparative HPLC, eluting with MeOH/H_2O (0-20 min, 42:58, v/v) to yield compound **4** (59.3 mg, t_R=12.1 min).

4: Corynether A. Brown solid; maximum UV absorption wavelength 284 nm (**Fig. S15**); ^{1}H NMR (500 MHz, methanol-d_4): δ 6.31 (d, J = 2.1 Hz, 1H), 6.25 (d, J = 2.8 Hz, 1H), 6.20 (d, J = 2.8 Hz, 1H), 5.87 (d, J = 2.1 Hz, 1H), 2.34 (s, 3H), 2.05 (s, 3H); ^{13}C NMR (125 MHz, methanol-d_4): δ 173.8, 161.5, 159.3,

157.1, 152.4, 140.5, 135.6, 134.5, 117.5, 112.4, 110.1, 103.4, 100.5, 21.0, 17.1 (**Fig. S17.5, 17.6**); HRESI-MS: *m/z* 291.08615 $[M+H]^+$ (calcd for $C_{15}H_{15}O_6$, 291.0869) (**Fig. S16.3**).[8,9]

1.2.5 Compound 5

The crude extract (1.33 g) of a 3 L fermentation broth of *S. cerevisiae* BJ5464-NpgA carrying plasmids YEpPreu6 + YEpLEU(DpeB+D+C) + YEpTRP(DpeE) was applied to an ODS column, eluting with a gradient of methanol/water (20:80, 25:75, 30:70, 35:65, 40:60, 45:55) to yield fractions A-F. After UHPLC-MS analysis, fraction D was further purified by semi-preparative HPLC, eluting with $MeOH/H_2O$ (0-20 min, 50:50, v/v) to yield compound **5** (22.5 mg, t_R=14.2 min).

5: Diaryl ether. Pale brown viscous oil; maximum UV absorption wavelength 281 nm (**Fig. S15**); ^1H NMR (500 MHz, acetone-d_6): δ 8.24 (br s, 1H), 8.20 (br s, 1H), 8.01 (br s, 1H), 6.35 (d, J = 2.9 Hz, 1H), 6.28 (m, 1H), 6.25 (dd, J = 2.9, 0.6 Hz, 1H), 6.13 (m, 1H), 6.04 (t, J = 2.1 Hz, 1H), 2.17 (s, 3H), 1.98 (s, 3H); ^{13}C NMR (125 MHz, acetone-d_6): δ 159.7, 158.4, 154.9, 150.6, 139.8, 133.2, 132.3, 109.2, 108.1, 106.8, 101.4, 99.1, 20.7, 15.5 (**Fig. S17.7, 17.8**); HRESI-MS: *m/z* 247.09642 $[M+H]^+$ (calcd for $C_{14}H_{15}O_4$, 247.0970) (**Fig. S16.4**).[10]

1.2.6 Compound 6

The crude extract (2.58 g) of a 4 L fermentation broth of *S. cerevisiae* BJ5464-NpgA carrying plasmids YEpPreu6 + YEpLEU(DpeB+D+C+E) + YEpTRP(DpeF) was applied to an ODS column, eluting with a gradient of methanol/water (20:80, 25:75, 30:70, 35:65, 40:60, 45:55) to yield fractions A-F. After UHPLC-MS analysis, fraction F was further purified by semi-preparative HPLC, eluting with $MeOH/H_2O$ (0-20 min, 70:30, v/v) to yield compound **6** (14.4 mg, t_R=6.1 min).

6: Cyperine. Colorless oil; maximum UV absorption wavelength 279 nm (**Fig. S15**); ^1H NMR (600 MHz, methanol-d_4): δ 6.35 (d, J = 2.9 Hz, 1H), 6.31 (d, J = 2.9 Hz, 1H), 6.23 (br s, 1H), 6.14 (br s, 1H), 6.03 (t, J = 2.0 Hz, 1H), 3.74 (s, 3H), 2.18 (s, 3H), 2.03 (s, 3H); ^{13}C NMR (150 MHz, methanol-d_4): δ 161.1, 159.6, 158.7, 151.9, 141.4, 135.8, 134.1, 110.4, 108.2, 107.9, 101.4, 100.4, 56.0, 21.8, 16.7 (**Fig. S17.9-17.11**); HRESI-MS: *m/z* 261.11206 $[M+H]^+$ (calcd for $C_{15}H_{17}O_4$, 261.1127) (**Fig. S16.5**).[11,12]

1.2.7 Compound 7

The crude extract (1.17 g) of a 5 L fermentation broth of *S. cerevisiae* BJ5464-NpgA carrying plasmids YEpPreu6 + YEpLEU(DpeB+D+C+E+F) + YEpTRP(DpeH) was applied to an ODS column, eluting with a gradient of methanol/water (20:80, 25:75, 30:70, 35:65, 40:60, 45:55) to yield fractions

A-F. After UHPLC-MS analysis, fraction F was further purified by semi-preparative HPLC, eluting with MeOH/H_2O (0-24 min, 55:45, v/v) to yield compound **7** (5.5 mg, t_R=15.4 min).

7: 5-methoxy-3,5'-dimethyl-2,3'-oxybiphenyl-1,1',2'-triol. Pale brown oil; maximum UV absorption wavelength 281 nm (**Fig. S15**); ^{1}H NMR (600 MHz, methanol-d_4): δ 6.36 (d, J = 2.9 Hz, 1H), 6.31 (d, J = 2.9 Hz, 1H), 6.28 (d, J = 1.2 Hz, 1H), 5.74 (d, J = 1.2 Hz, 1H), 3.74 (s, 3H), 2.05 (s, 3H), 2.03 (s, 3H); ^{13}C NMR (150 MHz, methanol-d_4): δ 158.7, 151.9, 148.1, 147.2, 136.1, 134.1, 132.7, 129.8, 111.0, 108.0, 106.8, 101.4, 56.0, 21.3, 16.6 (**Table S9.1**; **Fig. S17.12-17.16**); HRESI-MS: m/z 275.09393 [M-H]$^-$ (calcd for $C_{15}H_{15}O_5$, 275.0919) (**Fig. S16.6**).

1.2.8 Compound 8

The spores of *A. oryzae* NSAR1 co-transformed with plasmids pUSA(DpeA+C) + pAdeA(DpeB+D) + pTAex3(DpeE+F) + pUNA(DpeH+I) were inoculated into 100 g rice and 80 mL MEB (2% malt extract, 2% sucrose, and 1% peptone) mixed media, and fermented for 20 days. The crude extract (1.51 g) was applied to an ODS column, eluting with a gradient of methanol/water (20:80, 25:75, 30:70, 35:65, 40:60, 45:55, v/v) to yield fractions A-F. After UHPLC-MS analysis, fraction C was further purified by semi-preparative HPLC, eluting with MeOH/H_2O (0-20 min, 45:55, v/v) to yield compound **8** (105.5 mg, t_R=11.5 min).

8: 3,5'-dimethyl-2,3'-oxybiphenyl-1,5,1',2'-tetraol. Brown oil; maximum UV absorption wavelength 281 nm (**Fig. S15**); ^{1}H NMR (500 MHz, methanol-d_4): δ 6.28 (d, J = 2.0 Hz, 1H), 6.26 (d, J = 2.8 Hz, 1H), 6.19 (d, J = 2.8 Hz, 1H), 5.77 (d, J = 1.9 Hz, 1H), 2.05 (s, 3H), 2.01 (s, 3H); ^{13}C NMR (125 MHz, methanol-d_4): δ 156.0, 151.8, 148.2, 147.2, 135.3, 134.0, 132.7, 129.8, 111.0, 109.5, 106.8, 102.6, 21.4, 16.4 (**Table S9.1**; **Fig. S17.17-17.21**); HRESI-MS: m/z 263.09137 [M+H]$^+$ (calcd for $C_{14}H_{15}O_5$, 263.0919) (**Fig. S16.7**).

1.3 Antibacterial assay

All isolated DEPs and DPEs were evaluated for their antibacterial activities against eight antibiotic-resistant bacterial species, including carbapenems-resistant *Acinetobacter baumannii*, carbapenems-resistant *Escherichia coli*, carbapenems-resistant *Klebsiella pneumoniae*, carbapenems-resistant *Pseudomonas aeruginosa*, methicillin-resistant *Staphylococcus aureus*, multidrug-resistant *Enterococcus faecalis*, multidrug-resistant *Enterococcus faecium*, and multidrug-resistant *Staphylococcus epidermidis*.[2] Antibacterial activities against these strains were quantified in 96-well

plates by the modified broth dilution test as described previously.[13] Medium containing 1% DMSO and ciprofloxacin were used as the negative and positive control, respectively. The minimum inhibitory concentration (MIC) was defined as the lowest test concentration that completely inhibited the growth of the test organisms.

1.4 Chemical synthesis of the DPEs

1.4.1 Preparation of 3-(methoxymethoxy)-5-methylphenol

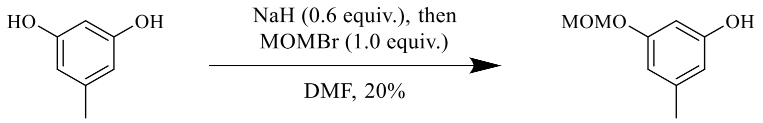

orcinol **3-(methoxymethoxy)-5-methylphenol**

NaH (60% in oil, 0.240 g, 6.0 mmol) was added to a 50 mL flask containing orcinol (CAS: 504-15-4, 1.241 g, 10.0 mmol) in DMF (10 mL) under nitrogen at 0 ºC. After stirring for 30 min, MOMBr (0.8 mL, 10.0 mmol) was added to the mixture, and continued stirring at room temperature for 3 h. The reaction mixture was quenched with H_2O, and the aqueous layer was extracted three times with EtOAc. The combined organic layer was washed with brine, dried with anhydrous Na_2SO_4, and then evaporated. The residue was purified by flash column chromatography (silica gel, PE:EtOAc [10:1 to 8:1, v:v]) to yield **3-(methoxymethoxy)-5-methylphenol** as a yellow oil (336.6 mg, 20%).[14] The reaction was repeated at the same scale to obtain enough compound for the following steps.

1.4.2 General procedure for the synthesis of 10, 11, 12 and 17

1.4.2.1 Preparation of the aryl halides

1-iodo-4-(methoxymethoxy)benzene

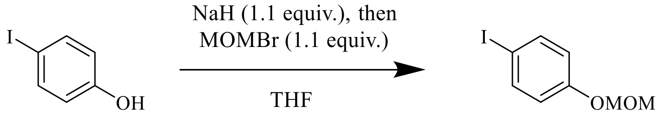

4-iodophenol **1-iodo-4-(methoxymethoxy)benzene**

NaH (60% in oil, 2.000 g, 50.0 mmol) was added to a 100 mL flask containing 4-iodophenol (CAS: 540-38-5, 10.000 g, 45.5 mmol) in THF (50 mL) under nitrogen at 0 ºC. After stirring for 30 min, MOMBr (4.2 mL, 50.0 mmol) was added to the mixture, and continued stirring at room temperature for 2 h. The reaction was quenched with NaOH (1 M, 100 mL) and extracted three times with EtOAc. The

combined organic mixture was washed with $NaHCO_3$ solution, brine, and dried over anhydrous Na_2SO_4, and then evaporated. The crude products were used without further purification.

1-bromo-3-(methoxymethoxy)-5-methylbenzene

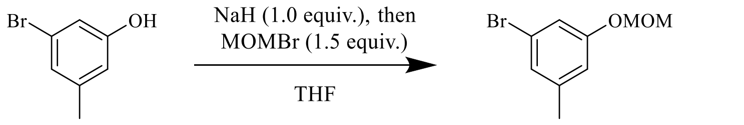

3-bromo-5-methylphenol **1-bromo-3-(methoxymethoxy)-5-methylbenzene**

NaH (60% in oil, 0.400 g, 10.0 mmol) was added to a 25 mL flask containing 3-bromo-5-methylphenol (CAS: 74204-00-5, 1.870 g, 10.0 mmol) in THF (10 mL) under nitrogen at 0 ºC. After stirring for 30 min, MOMBr (1.2 mL, 15.0 mmol) was added to the mixture, and continued stirring at room temperature for 2 h. The reaction was quenched with H_2O and the following steps were the same as those to prepare 1-iodo-4-(methoxymethoxy)benzene.

1.4.2.2 Ullmann-Ma reaction

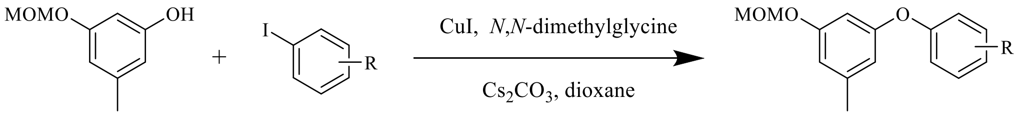

3-(methoxymethoxy)-5-methylphenol

18 : R = H
19 : R = *o*-CH_3
20 : R = *p*-OMOM

(1'-(methoxymethoxy)-5'-methylphenoxy)benzene (18)

Iodobenzene (CAS: 591-50-4, 0.612 g, 3.0 mmol), CuI (38.1 mg, 0.2 mmol), *N*,*N*-dimethylglycine hydrochloride (83.7 mg, 0.6 mmol), and Cs_2CO_3 (1.303 g, 4.0 mmol) were added sequentially to a 15 mL sealed tube containing 3-(methoxymethoxy)-5-methylphenol (0.504 g, 3.00 mmol) in dioxane (4 mL) under nitrogen. The mixture was heated to 90 ºC and stirred for 22 h. The reaction was monitored by TLC. After cooling to room temperature, the reaction was quenched with H_2O, extracted three times with EtOAc, washed with brine, dried over anhydrous Na_2SO_4, and then evaporated. The residue was purified by flash column chromatography (silica gel, PE:EtOAc [70:1, v:v]) to give a colorless oil **18** (209.6 mg, 29%).[15]

^{1}H NMR (600 MHz, methanol-d_4): δ 7.32 (t, J = 7.6 Hz, 2H), 7.09 (t, J = 7.4 Hz, 1H), 6.96 (d, J = 8.3 Hz, 2H), 6.60 (s, 1H), 6.44 (s, 1H), 6.40 (s, 1H), 5.10 (s, 2H), 3.41 (s, 3H), 2.24 (s, 3H); ^{13}C NMR (150 MHz, methanol-d_4): δ 160.0, 159.8, 158.6, 142.0, 131.0, 131.0, 124.6, 120.2, 120.2, 113.8, 112.9, 105.3, 95.6, 56.3, 21.8 (**Fig. S17.104, 17.105**). HRESI-MS: m/z 245.11746 $[M+H]^+$ (calcd for $C_{15}H_{17}O_3$, 245.1178) (**Fig. S16.28**).

3-methyl-2-(1'-(methoxymethoxy)-5'-methylphenoxy)benzene (19)

The synthesis of **19** followed the synthetic route of compound **18**, but with utilizing 1-iodo-2-methylbenzene (CAS: 615-37-2, 0.654 g, 3.0 mmol) instead of iodobenzene. The residue was purified by flash column chromatography (silica gel, PE:EtOAc [85:1, v:v]) to give a colorless oil **19** (150.4 mg, 19%).

^{1}H NMR (600 MHz, methanol-d_4): δ 7.25 (d, J = 7.5 Hz, 1H), 7.16 (t, J = 7.7 Hz, 1H), 7.06 (t, J = 7.4 Hz, 1H), 6.87 (d, J = 8.0 Hz, 1H), 6.54 (s, 1H), 6.32 (s, 1H), 6.28 (s, 1H), 5.09 (s, 2H), 3.40 (s, 3H), 2.22 (s, 3H), 2.17 (s, 3H); ^{13}C NMR (150 MHz, methanol-d_4): δ 160.5, 160.0, 155.6, 141.9, 132.6, 131.3, 128.4, 125.5, 121.3, 112.1, 112.0, 103.7, 95.5, 56.3, 21.8, 16.4 (**Fig. S17.106, 17.107**). HRESI-MS: *m*/*z* 259.13303 [M+H]$^+$ (calcd for $C_{16}H_{19}O_3$, 259.1334) (**Fig. S16.29**).

5-methoxymethoxy-2-(1'-(methoxymethoxy)-5'-methylphenoxy)benzene (20)

The synthesis of **20** followed the synthetic route of compound **18**, but with utilizing 1-iodo-4-(methoxymethoxy)benzene (0.792 g, 3.0 mmol) instead of iodobenzene. The residue was purified by flash column chromatography (silica gel, PE:EtOAc [70:1, v:v]) to give a pale yellow oil **20** (105.3 mg, 12%).

^{1}H NMR (600 MHz, methanol-d_4): δ 7.02 (d, J = 8.8 Hz, 2H), 6.92 (d, J = 8.8 Hz, 2H), 6.55 (s, 1H), 6.39 (s, 1H), 6.35 (s, 1H), 5.14 (br s, 2H), 5.09 (br s, 2H), 3.45 (br s, 3H), 3.41 (br s, 3H), 2.23 (s, 3H); ^{13}C NMR (150 MHz, methanol-d_4): δ 160.8, 159.9, 155.1, 152.6, 141.9, 121.8, 121.8, 118.8, 118.8, 112.8, 112.4, 104.4, 96.1, 95.6, 56.3, 56.3, 21.8 (**Fig. S17.108, 17.109**). HRESI-MS: *m*/*z* 305.13828 [M+H]$^+$ (calcd for $C_{17}H_{21}O_5$, 305.1389) (**Fig. S16.30**).

5,5'-oxybis(1-(methoxymethoxy)-3-methylbenzene) (25)

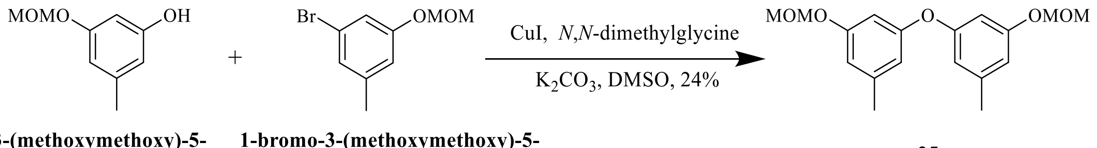

3-(methoxymethoxy)-5-methylphenol **1-bromo-3-(methoxymethoxy)-5-methylbenzene** **25**

The synthesis of **25** followed the synthetic route of compound **18**, but with utilizing 1-bromo-3-(methoxymethoxy)-5-methylbenzene (0.575 g, 2.5 mmol) instead of iodobenzene, and the amount of 3-(methoxymethoxy)-5-methylphenol changing to 2.5 mmol. The residue was purified by flash column chromatography (silica gel, PE:EtOAc [90:1, v:v]) to give a pale yellow oil **25** (192.5 mg, 24%).

^{1}H NMR (500 MHz, methanol-d_4): δ 6.62 (br s, 2H), 6.45 (t, J = 2.0 Hz, 2H), 6.43 (br s, 2H), 5.12 (s, 4H), 3.43 (s, 6H), 2.27 (s, 6H); ^{13}C NMR (125 MHz, methanol-d_4): δ 159.9, 159.9, 159.5, 159.5, 142.0,

142.0, 114.0, 114.0, 113.1, 113.1, 105.5, 105.5, 95.6, 95.6, 56.3, 56.3, 21.8, 21.8 (**Fig. S17.118, 17.119**). HRESI-MS: *m*/*z* 319.15396 $[M+H]^+$ (calcd for $C_{18}H_{23}O_5$, 319.1545) (**Fig. S16.35**).

1.4.2.3 Preparation of compounds 10, 11, 12, and 17

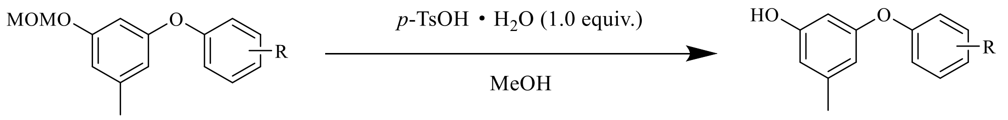

10 : R = H
11 : R = *o*-CH_3
12 : R = *p*-OH

5'-methyl-2,3'-oxybiphenyl-1'-ol (10)

p-Toluenesulfonic acid monohydrate (1.0 equiv.) was added to a 25 mL flask containing compound **18** (0.210 g, 0.9 mmol) in methanol (5 mL) under nitrogen. The reaction mixture was heated to 50 ºC and stirred for 10 h. After concentration under reduced pressure, the residue was purified by flash column chromatography (silica gel, PE:EtOAc [10:1, v:v]) to yield a colorless oil **10** (152.1 mg, 88%).

^1H NMR (600 MHz, methanol-d_4): δ 7.31 (t, J = 7.3 Hz, 2H), 7.07 (t, J = 7.3 Hz, 1H), 6.95 (d, J = 8.0 Hz, 2H), 6.36 (s, 1H), 6.26 (s, 1H), 6.19 (s, 1H), 2.21 (s, 3H); ^{13}C NMR (150 MHz, methanol-d_4): δ 159.9, 159.8, 158.8, 141.9, 130.9, 130.9, 124.4, 120.1, 120.1, 112.2, 111.7, 104.2, 21.7 (**Table S9.3**; **Fig. S17.32-17.35**). HRESI-MS: *m*/*z* 199.07663 $[M-H]^-$ (calcd for $C_{13}H_{11}O_2$, 199.0759) (**Fig. S16.10**).

3,5'-dimethyl-2,3'-oxybiphenyl-1'-ol (11)

The synthesis of **11** followed the synthetic route of **10**, utilizing compound **19** (0.150 g, 0.6 mmol) instead of **18**. The residue was purified by flash column chromatography (silica gel, PE:EtOAc [10:1, v:v]) to yield a colorless oil **11** (97.0 mg, 78%).

^1H NMR (600 MHz, methanol-d_4): δ 7.24 (d, J = 7.5 Hz, 1H), 7.16 (t, J = 7.7 Hz, 1H), 7.05 (t, J = 7.4 Hz, 1H), 6.87 (d, J = 8.0 Hz, 1H), 6.30 (s, 1H), 6.15 (s, 1H), 6.06 (s, 1H), 2.19 (s, 3H), 2.17 (s, 3H); ^{13}C NMR (150 MHz, methanol-d_4): δ 160.6, 159.8, 155.8, 141.8, 132.5, 131.3, 128.4, 125.4, 121.4, 111.3, 110.0, 102.5, 21.7, 16.4 (**Table S9.4**; **Fig. S17.40-17.43**). HRESI-MS: *m*/*z* 215.10657 $[M+H]^+$ (calcd for $C_{14}H_{15}O_2$, 215.1072) (**Fig. S16.12**).

5'-methyl-2,3'-oxybiphenyl-5,1'-diol (12)

The synthesis of **12** followed the synthetic route of **10**, utilizing compound **20** (0.105 g, 0.3 mmol) instead of **18**. The residue was purified by flash column chromatography (silica gel, PE:EtOAc [7:1, v:v]) to yield a pale yellow oil **12** (71.0 mg, 95%).

^{1}H NMR (600 MHz, methanol-d_4): δ 6.84 (d, J = 8.5 Hz, 2H), 6.77 (d, J = 8.6 Hz, 2H), 6.28 (s, 1H), 6.19 (s, 1H), 6.11 (s, 1H), 2.19 (s, 3H); ^{13}C NMR (150 MHz, methanol-d_4): δ 161.5, 159.6, 155.0, 150.6, 141.6, 122.3, 122.3, 117.2, 117.2, 111.2, 110.3, 102.8, 21.7 (**Table S9.5**; **Fig. S17.55-17.58**). HRESI-MS: *m*/*z* 215.07176 [M-H]$^-$ (calcd for $C_{13}H_{11}O_3$, 215.0708) (**Fig. S16.15**).

diorcinol (17)[16]

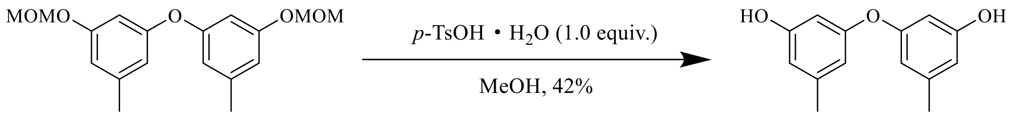

25 **17**

The synthesis of **17** followed the synthetic route of **10**, utilizing compound **25** (0.193 g, 0.6 mmol) instead of **18**. The residue was purified by flash column chromatography (silica gel, PE:EtOAc [8:1, v:v]) to yield a pale yellow oil **17** (58.7 mg, 42%).

^{1}H NMR (600 MHz, methanol-d_4): δ 6.36 (br s, 2H), 6.27 (br s, 2H), 6.20 (t, J = 2.1 Hz, 2H), 2.22 (s, 6H); ^{13}C NMR (150 MHz, methanol-d_4): δ 159.8, 159.8, 159.7, 159.7, 141.8, 141.8, 112.1, 112.1, 111.9, 111.9, 104.4, 104.4, 21.7, 21.7 (**Table S9.10**; **Fig. S17.95-17.98**). HRESI-MS: *m*/*z* 229.08743 [M-H]$^-$ (calcd for $C_{14}H_{13}O_3$, 229.0865) (**Fig. S16.25**).

1.4.3 General procedure for the synthesis of 13, 14, 15, 16 and 26

1.4.3.1 Preparation of the aryl halides

1-iodo-2-methoxybenzene

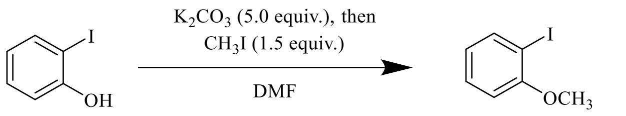

2-iodophenol **1-iodo-2-methoxybenzene**

K_2CO_3 (3.455 g, 25.0 mmol) was added to a 50 mL flask containing 2-iodophenol (CAS: 533-58-4, 1.100 g, 5.0 mmol) in dry DMF (15 mL). After stirring for 10 min, CH_3I (1.065 g, 7.5 mmol) was added to the mixture, and continued stirring at 50 ºC for 3 h. The reaction was monitored by TLC. After cooling to room temperature, the reaction was quenched with H_2O, extracted three times with EtOAc, washed with brine, dried over anhydrous Na_2SO_4, and then evaporated.[17] The crude products were used without further purification.

1-bromo-3-methoxy-5-methylbenzene

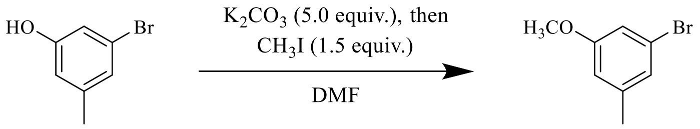

3-bromo-5-methylphenol **1-bromo-3-methoxy-5-methylbenzene**

K_2CO_3 (6.911 g, 50.0 mmol) was added to a 50 mL flask containing 3-bromo-5-methylphenol (1.870 g, 10.0 mmol) in dry DMF (20 mL). After stirring for 10 min, CH_3I (2.129 g, 15.0 mmol) was added to the mixture, and continued stirring at 50 ºC for 3 h. The following steps were the same as those to prepare 1-iodo-2-methoxybenzene.

1-iodo-4-methoxy-2-methylbenzene

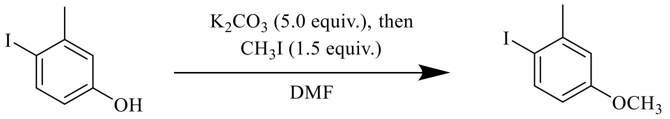

4-iodo-3-methylphenol **1-iodo-4-methoxy-2-methylbenzene**

K_2CO_3 (4.429 g, 32.0 mmol) was added to a 50 mL flask containing 4-iodo-3-methylphenol (CAS: 133921-27-4, 1.500 g, 6.4 mmol) in dry DMF (25 mL). After stirring for 10 min, CH_3I (1.365 g, 9.6 mmol) was added to the mixture, and continued stirring at 50 ºC for 3 h. The following steps were the same as those to prepare 1-iodo-2-methoxybenzene.

1.4.3.2 Ullmann-Ma reaction

1-methoxy-2-(1'-(methoxymethoxy)-5'-methylphenoxy)benzene (21)

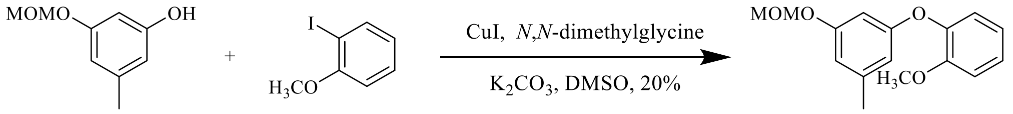

3-(methoxymethoxy)-5-methylphenol **1-iodo-2-methoxybenzene** **21**

1-iodo-2-methoxybenzene (0.702 g, 3.0 mmol), CuI (0.114 g, 0.6 mmol), *N,N*-dimethylglycine hydrochloride (0.167 g, 1.2 mmol), and K_2CO_3 (0.553 g, 4.0 mmol) were added sequentially to a 15 mL sealed tube containing 3-(methoxymethoxy)-5-methylphenol (0.504 g, 3.0 mmol) in DMSO (4 mL) under nitrogen. The mixture was heated to 120 ºC and stirred for 36 h. The reaction was monitored by TLC. After cooling to room temperature, the reaction was quenched with H_2O, extracted three times with EtOAc, washed with brine, dried over anhydrous Na_2SO_4, and then evaporated. The residue was purified by flash column chromatography (silica gel, PE:EtOAc) to give a pale yellow oil **21** (0.166 g, 20%).[15,18]

^{1}H NMR (600 MHz, methanol-*d*$_4$): *δ* 7.17 (t, *J* = 7.7 Hz, 1H), 7.10 (d, *J* = 8.1 Hz, 1H), 6.98 (d, *J* = 7.9 Hz, 1H), 6.95 (t, *J* = 7.7 Hz, 1H), 6.52 (s, 1H), 6.31 (s, 1H), 6.29 (s, 1H), 5.09 (s, 2H), 3.78 (s, 3H), 3.41 (s, 3H), 2.23 (s, 3H); ^{13}C NMR (150 MHz, methanol-*d*$_4$): *δ* 160.9, 159.9, 153.4, 145.9, 141.6, 126.7, 123.1, 122.4, 114.6, 111.9, 111.6, 103.3, 95.6, 56.5, 56.3, 21.8 (**Fig. S17.110, 17.111**). HRESI-MS: *m*/*z* 275.12778 $[M+H]^+$ (calcd for $C_{16}H_{19}O_4$, 275.1283) (**Fig. S16.31**).

1-methoxy-2-(1'-(methoxymethoxy)-5'-methylphenoxy)-3-methylbenzene (22)

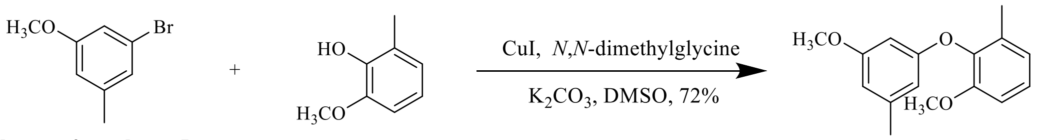

1-bromo-3-methoxy-5-methylbenzene **2-methoxy-6-methylphenol** **22**

The synthesis of **22** followed the synthetic route of compound **21** with utilizing 1-bromo-3-methoxy-5-methylbenzene (0.500 g, 2.5 mmol) instead of 1-iodo-2-methoxybenzene, and utilizing 2-methoxy-6-methylphenol (CAS: 2896-67-5, 0.345 g, 2.5 mmol) instead of 3-(methoxymethoxy)-5-methylphenol. The residue was purified by flash column chromatography (silica gel, PE:EtOAc) to give a yellow oil **22** (0.465 g, 72%).

^{1}H NMR (500 MHz, methanol-*d*$_4$): *δ* 7.10 (t, *J* = 8.0 Hz, 1H), 6.92 (d, *J* = 8.2 Hz, 1H), 6.86 (d, *J* = 7.6 Hz, 1H), 6.35 (br s, 1H), 6.12 (br s, 1H), 6.09 (t, *J* = 2.1 Hz, 1H), 3.72 (s, 3H), 3.68 (s, 3H), 2.20 (s, 3H), 2.11 (s, 3H); ^{13}C NMR (125 MHz, methanol-*d*$_4$): *δ* 162.4, 160.8, 154.1, 142.9, 141.4, 133.9, 126.8, 124.1, 111.8, 108.8, 108.8, 99.3, 56.4, 55.7, 21.9, 16.2 (**Fig. S17.112, 17.113**). HRESI-MS: *m*/*z* 259.13281 $[M+H]^+$ (calcd for $C_{16}H_{19}O_3$, 259.1334) (**Fig. S16.32**).

5-methoxy-2-(1'-(methoxymethoxy)-5'-methylphenoxy)-3-methylbenzene (23)

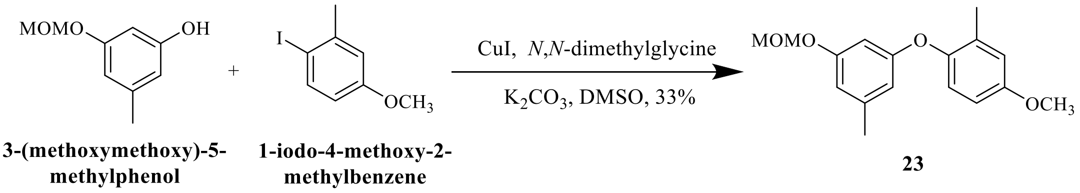

3-(methoxymethoxy)-5-methylphenol **1-iodo-4-methoxy-2-methylbenzene** **23**

The synthesis of **23** followed the synthetic route of compound **21**, utilizing 1-iodo-4-methoxy-2-methylbenzene (0.620 g, 2.5 mmol) instead of 1-iodo-2-methoxybenzene, and the amount of 3-(methoxymethoxy)-5-methylphenol changing to 2.5 mmol. The residue was purified by flash column chromatography (silica gel, PE:EtOAc) to give a pale yellow oil **23** (0.241 g, 33%).

^{1}H NMR (600 MHz, methanol-*d*$_4$): *δ* 6.85 (d, *J* = 8.7 Hz, 1H), 6.83 (br s, 1H), 6.75 (dd, *J* = 8.8, 2.9 Hz, 1H), 6.50 (s, 1H), 6.28 (s, 1H), 6.24 (s, 1H), 5.08 (s, 2H), 3.78 (s, 3H), 3.41 (s, 3H), 2.22 (s, 3H),

2.12 (s, 3H); ^{13}C NMR (150 MHz, methanol-d_4): δ 161.3, 160.0, 158.1, 148.7, 141.8, 132.9, 123.1, 117.6, 113.4, 111.5, 111.2, 102.8, 95.6, 56.3, 56.1, 21.8, 16.6 (**Fig. S17.114, 17.115**). HRESI-MS: *m*/*z* 289.14337 [M+H]$^+$ (calcd for $C_{17}H_{21}O_4$, 289.1440) (**Fig. S16.33**).

1,5-dimethoxy-2-(1'-(methoxymethoxy)-5'-methylphenoxy)benzene (24)

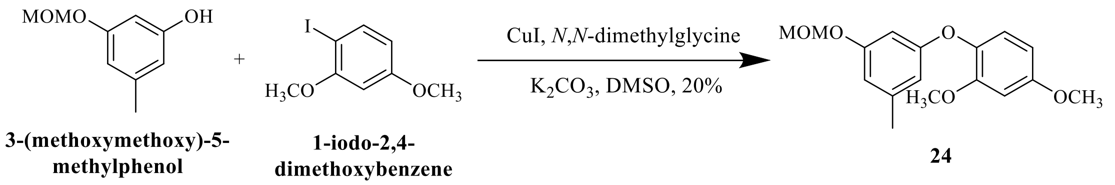

The synthesis of **24** followed the synthetic route of compound **23**, utilizing 1-iodo-2,4-dimethoxybenzene (CAS: 20469-63-0, 0.660 g, 2.5 mmol) instead of 1-iodo-4-methoxy-2-methylbenzene. The residue was purified by flash column chromatography (silica gel, PE:EtOAc) to give a pale yellow oil **24** (0.150 g, 20%).

^{1}H NMR (600 MHz, methanol-d_4): δ 6.92 (d, J = 8.6 Hz, 1H), 6.66 (d, J = 1.7 Hz, 1H), 6.51 (dd, J = 8.6, 1.7 Hz, 1H), 6.48 (s, 1H), 6.27 (s, 1H), 6.24 (s, 1H), 5.08 (s, 2H), 3.80 (s, 3H), 3.75 (s, 3H), 3.41 (s, 3H), 2.21 (s, 3H); ^{13}C NMR (150 MHz, methanol-d_4): δ 161.6, 159.8, 159.3, 154.2, 141.4, 139.1, 123.9, 111.4, 111.0, 105.9, 102.6, 101.8, 95.6, 56.5, 56.3, 56.2, 21.8 (**Fig. S17.116, 17.117**). HRESI-MS: *m*/*z* 305.13828 [M+H]$^+$ (calcd for $C_{17}H_{21}O_5$, 305.1389) (**Fig. S16.34**).

1',6'-dimethoxy-3'-phenoxybenzene (27)

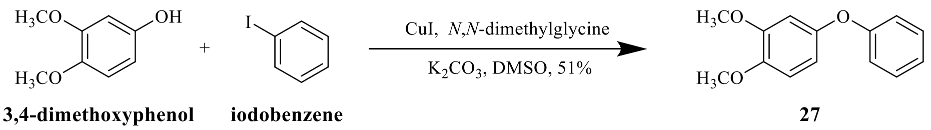

The synthesis of **27** followed the synthetic route of compound **21**, utilizing iodobenzene (0.408 g, 2.0 mmol) instead of 1-iodo-2-methoxybenzene, and utilizing 3,4-dimethoxyphenol (CAS: 2033-89-8, 0.308 g, 2.0 mmol) instead of 3-(methoxymethoxy)-5-methylphenol. The residue was purified by flash column chromatography (silica gel, PE:EtOAc) to give a pale yellow oil **27** (0.233 g, 51%).

^{1}H NMR (600 MHz, DMSO-d_6): δ7.34 (tt, J = 7.4, 2.5 Hz, 2H), 7.06 (tt, J = 7.5, 1.0 Hz, 1H), 6.96-6.91 (m, 3H), 6.74 (d, J = 2.7 Hz, 1H), 6.52 (dd, J = 8.7, 2.8 Hz, 1H), 3.74 (s, 3H), 3.72 (s, 3H).; ^{13}C NMR (150 MHz, DMSO-d_6): δ 157.9, 149.8, 149.6, 145.3, 129.9, 129.9, 122.6, 117.3, 117.3, 112.5, 110.6, 104.8, 55.9, 55.6 (**Fig. S17.122, 17.123**). HRESI-MS: *m*/*z* 231.10162 [M+H]$^+$ (calcd for $C_{14}H_{15}O_3$, 231.1021) (**Fig. S16.37**).

1.4.3.3 Preparation of compounds 13, 14, 15, 16 and 26

5'-methyl-2,3'-oxybiphenyl-1,1'-diol (13)

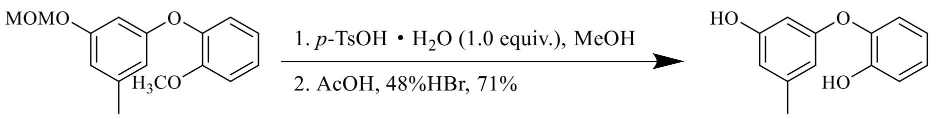

21 **13**

p-Toluenesulfonic acid monohydrate (1.0 equiv.) was added to a 25 mL flask containing compound **21** (0.166 g, 0.6 mmol) in methanol (5 mL) under nitrogen. The reaction mixture was heated to 50 ºC and stirred for 10 h, and then was concentrated under reduced pressure. Next, glacial acetic acid (3 mL) and 48% HBr (2 mL) were added to the concentrated mixture and refluxed at 120 ºC for 5 h. The reaction solution was then poured into an equal volume of water, and the pH was adjusted to 6-7 with saturated $NaHCO_3$ solution. The mixture was extracted three times with EtOAc, washed with brine, dried over anhydrous Na_2SO_4, and then evaporated. The residue was purified by flash column chromatography (silica gel, PE:EtOAc) to yield a fawn oil **13** (92.7 mg, 71%).

^{1}H NMR (500 MHz, methanol-*d*$_4$): δ 6.99 (ddd, *J* = 8.0, 7.3, 1.6 Hz, 1H), 6.92 (dd, *J* = 8.0, 1.6 Hz, 1H), 6.88 (dd, *J* = 8.0, 1.6 Hz, 1H), 6.80 (ddd, *J* = 8.0, 7.3, 1.6 Hz, 1H), 6.31 (m, 1H), 6.24 (m, 1H), 6.16 (t, *J* = 2.1 Hz, 1H), 2.20 (s, 3H); ^{13}C NMR (125 MHz, methanol-*d*$_4$): δ 160.6, 159.6, 150.4, 145.1, 141.6, 126.1, 122.3, 121.2, 118.2, 111.4, 110.3, 102.7, 21.7 (**Table S9.6**; **Fig. S17.63-17.66**). HRESI-MS: *m/z* 215.07178 [M-H]$^-$ (calcd for $C_{13}H_{11}O_3$, 215.0708) (**Fig. S16.17**).

3,5'-dimethyl-2,3'-oxybiphenyl-1,1'-diol (14)

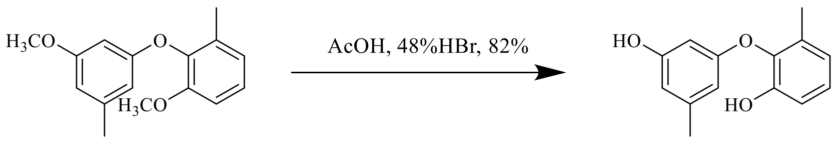

22 **14**

Glacial acetic acid (6 mL) and 48% HBr (4 mL) were added to a 50 mL flask containing compound **22** (0.453 g, 1.8 mmol) and refluxed at 120 ºC for 5 h. The remaining steps followed the synthetic route of **13.** The residue was purified by flash column chromatography (silica gel, PE:EtOAc) to give a brown solid **14** (330.7mg, 82%).

^{1}H NMR (600 MHz, DMSO-*d*$_6$): δ 6.94 (t, *J* = 7.8 Hz, 1H), 6.78 (d, *J* = 8.0 Hz, 1H), 6.70 (d, *J* = 7.4 Hz, 1H), 6.18 (br s, 1H), 6.05 (br s, 1H), 5.91 (t, *J* = 1.9 Hz, 1H), 2.13 (s, 3H), 2.02 (s, 3H); ^{13}C NMR (150 MHz, DMSO-*d*$_6$): δ 158.8, 158.3, 150.2, 139.9, 139.5, 132.0, 125.4, 121.1, 114.7, 109.2, 106.3, 98.9, 21.3, 15.9 (**Table S9.7**; **Fig. S17.71-17.74**). HRESI-MS: *m/z* 229.08760 [M-H]$^-$ (calcd for $C_{14}H_{13}O_3$, 229.0865) (**Fig. S16.19**).

3,5'-dimethyl-2,3'-oxybiphenyl-5,1'-diol (15)

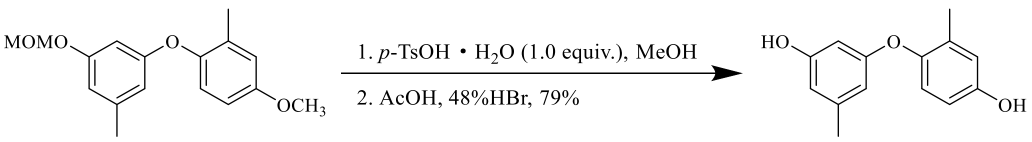

23 **15**

The synthesis of **15** followed the synthetic route of **13**, utilizing compound **23** (0.120 g, 0.4 mmol) instead of **21**. The residue was purified by flash column chromatography (silica gel, PE:EtOAc) to yield a pale yellow oil **15** (75.5 mg, 79%).

^{1}H NMR (600 MHz, methanol-*d*$_4$): δ 6.76 (d, *J* = 8.6 Hz, 1H), 6.68 (br s, 1H), 6.61 (br d, 1H), 6.24 (s, 1H), 6.11 (s, 1H), 6.01 (s, 1H), 2.18 (s, 3H), 2.07 (s, 3H); ^{13}C NMR (150 MHz, methanol-*d*$_4$): δ 161.7, 159.6, 155.3, 147.8, 141.6, 132.9, 123.3, 118.7, 114.7, 110.6, 109.0, 101.4, 21.8, 16.5 (**Table S9.8**; **Fig. S17.79-17.82**). HRESI-MS: *m/z* 229.08762 [M-H]$^-$ (calcd for $C_{14}H_{13}O_3$, 229.0865) (**Fig. S16.21**).

5'-methyl-2,3'-oxybiphenyl-1,5,1'-triol (16)

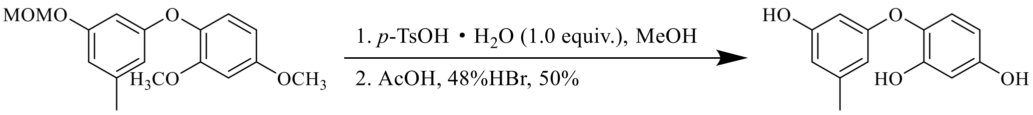

24 **16**

The synthesis of **16** followed the synthetic route of **13**, utilizing compound **24** (0.143 g, 0.5 mmol) instead of **21**. The residue was purified by flash column chromatography (silica gel, PE:EtOAc) to yield a fawn oil **16** (54.8 mg, 50%).

^{1}H NMR (600 MHz, methanol-*d*$_4$): δ 6.73 (d, *J* = 8.6 Hz, 1H), 6.41 (d, *J* = 2.3 Hz, 1H), 6.26 (dd, *J* = 8.6, 2.2 Hz, 1H), 6.25 (s, 1H), 6.19 (s, 1H), 6.11 (s, 1H), 2.19 (s, 3H); ^{13}C NMR (150 MHz, methanol-*d*$_4$): δ 161.7, 159.5, 156.2, 151.3, 141.3, 137.4, 123.7, 110.7, 109.4, 107.7, 105.2, 101.8, 21.8 (**Table S9.9**; **Fig. S17.87-17.90**). HRESI-MS: *m/z* 231.06688 [M-H]$^-$ (calcd for $C_{13}H_{11}O_4$, 231.0657) (**Fig. S16.23**).

2,3'-oxybiphenyl-1',6'-diol (26)

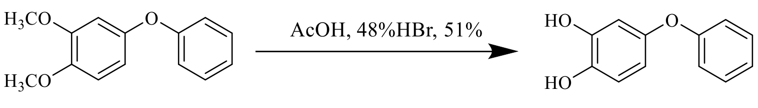

27 **26**

Glacial acetic acid (4 mL) and 48% HBr (3 mL) were added to a 50 mL flask containing compound **27** (0.233 g, 1.0 mmol) and the remaining steps followed the synthetic route of **14**. The residue was purified·by flash column chromatography (silica gel, PE:EtOAc) to give a yellow oil **26** (104.1 mg, 51%).

^{1}H NMR (600 MHz, DMSO-d_6): δ 9.16 (s, 1H), 8.80 (s, 1H), 7.31 (tt, J = 7.5, 2.2 Hz, 2H), 7.03 (tt, J = 7.4, 1.0 Hz, 1H), 6.89 (m, 2H), 6.72 (d, J = 8.5 Hz, 1H), 6.43 (d, J = 2.8 Hz, 1H), 6.31 (dd, J = 8.5, 2.8 Hz, 1H); ^{13}C NMR (150 MHz, DMSO-d_6): δ 158.2, 148.2, 146.2, 141.9, 129.8, 129.8, 122.3, 117.2, 117.2, 115.9, 110.0, 107.6 (**Fig. S17.120, 17.121**). HRESI-MS: m/z 201.05592 [M-H]$^-$ (calcd for $C_{12}H_9O_3$, 201.0552) (**Fig. S16.36**).

1.5 Biotransformation and characterization of the DPE products

1.5.1 Feeding experiments

For biotransformation experiments in yeast, YEpTRP(DpeF) was transformed into *S. cerevisiae* BJ5464-NpgA. Transformation and transformants selection processes followed the protocols in **1.1.3.1**. Twelve independent transformants were tested for the biotransformation of compound **8**, and fermentations with representative isolates were repeated at least three times for product identification. The selected transformants were grown in 100 mL Erlenmeyer flasks containing 10 mL of the appropriate SD medium at 30ºC with shaking at 200 rpm until the OD_{600nm} reached 0.6. An equivalent volume of YPD medium and 0.5 mg compound **8** (dissolved in DMSO) were then added to each flask, and the cultures were shaken at 200 rpm for further 2 days at 30 ºC. Extraction and structure characterization processes followed the protocols in **1.2.1**.

For biotransformation experiments in *Aspergillus*, pTAex3(DpeF), pTAex3(DpeH) or pTAex3(AN7912) were transformed into *A. oryzae* NSAR1, respectively. Transformation and transformants selection processes followed the protocols in **1.1.3.2**. Twelve independent transformants each were tested for the biotransformation of the DPE substrates, and fermentations with representative isolates were repeated at least three times for product identification. The selected transformants were grown in 300 mL Erlenmeyer flasks containing 75 mL of DPY medium at 30ºC with shaking at 160 rpm for 4 days. An equivalent volume of CD medium and 5 mg DPE substrate (dissolved in DMSO) were then added to each flask, and the cultures were shaken at 160 rpm for further 12 hours at 30 ºC. Extraction and structure characterization processes followed the protocols in **1.2.1**.

1.5.2 Compounds 9a/b

80 mg of 3'-Phenoxyphenol (**9**, CAS: 713-68-8, purchased from MACKLIN®) was fed to the *dpeH* gene-harboring *A. oryzae* transformant. After the fermentation, the crude extract (0.16 g) was chromatographed on Sephadex LH-20 (MeOH) followed by semi-preparative HPLC, eluting with

MeOH/H_2O (MeOH from 40% to 80% for 30 min, from 80% to 40% for 1 min, then kept at 40% for 4 min) to yield compounds **9a** (1.0 mg, t_R=14.2 min) and **9b** (5.6 mg, t_R=21.8 min).

9a: 2,3'-oxybiphenyl-1',4'-diol. Dark brown solid; maximum UV absorption wavelength 293 nm (**Fig. S15**); ^{1}H NMR (500 MHz, DMSO-d_6): δ 7.30 (tt, J = 7.5, 2.2 Hz, 2H), 7.01 (t, J = 7.4 Hz, 1H), 6.85 (d, J = 8.7 Hz, 2H), 6.76 (d, J = 8.7 Hz, 1H), 6.44 (dd, J = 8.7, 2.8 Hz, 1H), 6.33 (d, J = 2.8 Hz, 1H); ^{13}C NMR (125 MHz, DMSO-d_6): δ 157.8, 150.3, 142.8, 141.5, 129.6, 129.6, 122.0, 117.6, 116.5, 116.5, 111.6, 108.5 (**Table S9.2**; **Fig. S17.22-17.26**); HRESI-MS: m/z 201.05589 [M-H]$^-$ (calcd for $C_{12}H_9O_3$, 201.0552) (**Fig. S16.8**).

9b: 2,3'-oxybiphenyl-1',6'-diol. Yellow oil; maximum UV absorption wavelength 288 nm (**Fig. S15**); ^{1}H NMR (500 MHz, DMSO-d_6): δ7.31 (tt, J = 7.7, 2.2 Hz, 2H), 7.03 (t, J = 7.4 Hz, 1H), 6.89 (d, J = 8.1 Hz, 2H), 6.71 (d, J = 8.5 Hz, 1H), 6.42 (d, J = 2.6 Hz, 1H), 6.30 (dd, J = 8.4, 2.6 Hz, 1H); ^{13}C NMR (125 MHz, DMSO-d_6): δ 158.3, 148.2, 146.3, 142.0, 129.8, 129.8, 122.2, 117.2, 117.2, 116.0, 109.9, 107.7 (**Table S9.2**; **Fig. S17.27-17.31**); HRESI-MS: m/z 201.05595 [M-H]$^-$ (calcd for $C_{12}H_9O_3$, 201.0552) (**Fig. S16.9**).

1.5.3 Compound 10a

30 mg of compound **10** was fed to the *dpeH* gene-harboring *A. oryzae* transformant. After the fermentation, the crude extract was directly purified by semi-preparative HPLC, eluting with MeOH/H_2O (0-26 min, 50:50, v/v) to yield compound **10a** (1.5 mg, t_R=13.8 min).

10a: 5'-methyl-2,3'-oxybiphenyl-1',2'-diol. Black solid; maximum UV absorption wavelength 273 nm (**Fig. S15**); ^{1}H NMR (500 MHz, methanol-d_4): δ 7.28 (m, 2H), 7.01 (t, J = 7.4 Hz, 1H), 6.91 (d, J = 8.7 Hz, 1H), 6.91 (d, J = 8.5 Hz, 2H), 6.46 (d, J = 1.4 Hz, 1H), 6.20 (d, J = 1.2 Hz, 1H), 2.14 (s, 3H); ^{13}C NMR (125 MHz, methanol-d_4): δ 159.7, 148.0, 145.4, 136.2, 130.6, 130.6, 130.2, 123.4, 118.4, 118.4, 113.4, 113.3, 21.1 (**Table S9.3**; **Fig. S17.36-17.39**); HRESI-MS: m/z 215.07193 [M-H]$^-$ (calcd for $C_{13}H_{11}O_3$, 215.0708) (**Fig. S16.11**).

1.5.4 Compounds 11a/c

30 mg of compound **11** was fed to the *dpeH* gene-harboring *A. oryzae* transformant. After the fermentation, the crude extract was directly purified by semi-preparative HPLC, eluting with MeCN/H_2O (0-22 min, 50:50, v/v) to yield compounds **11c** (1.7 mg, t_R=12.0 min) and **11a** (1.0 mg, t_R=19.4 min).

11a: 3,5'-dimethyl-2,3'-oxybiphenyl-1',2'-diol. Black solid; maximum UV absorption wavelength 277 nm (**Fig. S15**); ^{1}H NMR (600 MHz, methanol-d_4): δ 7.22 (d, J = 7.0 Hz, 1H), 7.11 (t, J = 7.7 Hz, 1H),

6.99 (t, J = 7.4 Hz, 1H), 6.76 (d, J = 8.1 Hz, 1H), 6.40 (d, J = 1.4 Hz, 1H), 5.98 (d, J = 1.3 Hz, 1H), 2.27 (s, 3H), 2.10 (s, 3H); ^{13}C NMR (150 MHz, methanol-d_4): δ 156.9, 147.7, 146.7, 135.1, 132.3, 130.2, 130.1, 128.1, 124.4, 119.2, 112.4, 111.3, 21.1, 16.4 (**Table S9.4**; **Fig. S17.44-17.48**); HRESI-MS: *m/z* 229.08766 [M-H]$^-$ (calcd for $C_{14}H_{13}O_3$, 229.0865) (**Fig. S16.13**).

11c: 3-hydroxymethyl-5'-methyl-2,3'-oxybiphenyl-1'-ol. Pale purple solid; maximum UV absorption wavelength 277 nm (**Fig. S15**); ^{1}H NMR (600 MHz, methanol-d_4): δ 7.52 (d, J = 7.4 Hz, 1H), 7.25 (t, J = 7.3 Hz, 1H), 7.15 (t, J = 7.4 Hz, 1H), 6.87 (d, J = 8.0 Hz, 1H), 6.34 (s, 1H), 6.22 (s, 1H), 6.14 (s, 1H), 4.63 (s, 2H), 2.21 (s, 3H); ^{13}C NMR (150 MHz, methanol-d_4): δ 160.3, 159.9, 155.2, 141.9, 134.2, 129.7, 129.7, 125.1, 120.5, 111.9, 110.7, 103.3, 60.1, 21.7 (**Table S9.4**; **Fig. S17.49-17.54**); HRESI-MS: *m/z* 229.08771 [M-H]$^-$ (calcd for $C_{14}H_{13}O_3$, 229.0865) (**Fig. S16.14**).

1.5.5 Compound 12a

30 mg of compound **12** was fed to the *dpeH* gene-harboring *A. oryzae* transformant. After the fermentation, the crude extract was directly purified by semi-preparative HPLC, eluting with MeOH/H_2O (0-21 min, 47:53, v/v) to yield compound **12a** (2.0 mg, t_R=14.4 min).

12a: 5'-methyl-2,3'-oxybiphenyl-5,1',2'-triol. Pale brown solid; maximum UV absorption wavelength 281 nm (**Fig. S15**); ^{1}H NMR (600 MHz, methanol-d_4): δ 6.81 (d, J = 8.6 Hz, 2H), 6.73 (d, J = 8.6 Hz, 2H), 6.38 (s, 1H), 6.08 (s, 1H), 2.10 (s, 3H); ^{13}C NMR (150 MHz, methanol-d_4): δ 154.3, 151.8, 147.6, 147.3, 135.2, 130.0, 120.7, 120.7, 117.0, 117.0, 112.3, 111.6, 21.1 (**Table S9.5**; **Fig. S17.59-17.62**); HRESI-MS: *m/z* 231.06688 [M-H]$^-$ (calcd for $C_{13}H_{11}O_4$, 231.0657) (**Fig. S16.16**).

1.5.6 Compound 13a

20 mg of compound **13** was fed to the *dpeH* gene-harboring *A. oryzae* transformant. After the fermentation, the crude extract was directly purified by semi-preparative HPLC, eluting with MeOH/H_2O (0-25 min, 50:50, v/v) to yield compound **13a** (1.2 mg, t_R=16.3 min).

13a: 5'-methyl-2,3'-oxybiphenyl-1,1',2'-triol. Pale brown solid; maximum UV absorption wavelength 278 nm (**Fig. S15**); ^{1}H NMR (600 MHz, methanol-d_4): δ 6.94 (ddd, J = 8.4, 6.9, 1.6 Hz, 1H), 6.91 (dd, J = 8.0, 1.7 Hz, 1H), 6.80 (dd, J = 8.3, 1.7 Hz, 1H), 6.76 (ddd, J = 8.2, 7.0, 1.7 Hz, 1H), 6.41 (d, J = 1.4 Hz, 1H), 6.14 (d, J = 1.3 Hz, 1H), 2.12 (s, 3H); ^{13}C NMR (150 MHz, methanol-d_4): δ 149.4, 147.7, 146.6, 146.4, 135.2, 130.1, 125.2, 121.1, 120.1, 117.9, 112.7, 111.6, 21.2 (**Table S9.6**; **Fig. S17.67-17.70**); HRESI-MS: *m/z* 231.06686 [M-H]$^-$ (calcd for $C_{13}H_{11}O_4$, 231.0657) (**Fig. S16.18**).

1.5.7 Compound 14a

20 mg of compound **14** was fed to the *dpeH* gene-harboring *A. oryzae* transformant. After the fermentation, the crude extract was directly purified by semi-preparative HPLC, eluting with MeOH/H_2O (0-20 min, 52:48, v/v) to yield compound **14a** (3.0 mg, t_R=14.8 min).

14a: 3,5'-dimethyl-2,3'-oxybiphenyl-1,1',2'-triol. Pale brown solid; maximum UV absorption wavelength 277 nm (**Fig. S15**); ^{1}H NMR (600 MHz, DMSO-d_6): δ 6.92 (t, J = 7.8 Hz, 1H), 6.76 (d, J = 7.6 Hz, 1H), 6.68 (d, J = 7.5 Hz, 1H), 6.21 (s, 1H), 5.61 (s, 1H), 2.03 (s, 3H), 1.96 (s, 3H); ^{13}C NMR (150 MHz, DMSO-d_6): δ 150.3, 146.5, 146.3, 140.8, 132.0, 132.0, 126.6, 125.1, 121.1, 114.7, 110.0, 105.2, 20.8, 15.8 (**Table S9.7**; **Fig. S17.75-17.78**); HRESI-MS: *m/z* 245.08279 [M-H]$^-$ (calcd for $C_{14}H_{13}O_4$, 245.0814) (**Fig. S16.20**).

1.5.8 Compound 15a

50 mg of compound **15** was fed to the *dpeH* gene-harboring *A. oryzae* transformant. After the fermentation, the crude extract was directly purified by semi-preparative HPLC, eluting with MeOH/H_2O (0-22 min, 49:51, v/v) to yield compound **15a** (1.7 mg, t_R=15.5 min).

15a: 3,5'-dimethyl-2,3'-oxybiphenyl-5,1',2'-triol. Pale brown solid; maximum UV absorption wavelength 281 nm (**Fig. S15**); ^{1}H NMR (600 MHz, methanol-d_4): δ 6.72 (d, J = 8.6 Hz, 1H), 6.67 (d, J = 2.8 Hz, 1H), 6.58 (dd, J = 8.6, 2.9 Hz, 1H), 6.31 (br s, 1H), 5.83 (br s, 1H), 2.13 (s, 3H), 2.06 (s, 3H); ^{13}C NMR (150 MHz, methanol-d_4): δ 154.9, 148.7, 148.2, 147.3, 133.9, 132.3, 129.8, 122.2, 118.7, 114.5, 111.4, 109.1, 21.2, 16.5 (**Table S9.8**; **Fig. S17.83-17.86**); HRESI-MS: *m/z* 245.08273 [M-H]$^-$ (calcd for $C_{14}H_{13}O_4$, 245.0814) (**Fig. S16.22**).

1.5.9 Compound 16a

20 mg of compound **16** was fed to the *dpeH* gene-harboring *A. oryzae* transformant. After the fermentation, the crude extract was directly purified by semi-preparative HPLC, eluting with MeOH/H_2O (0-30 min, 36:64, v/v) to yield compound **16a** (1.0 mg, t_R=26.7 min).

16a: 5'-methyl-2,3'-oxybiphenyl-1,5,1',2'-tetraol. Pale brown solid; maximum UV absorption wavelength 283 nm (**Fig. S15**); ^{1}H NMR (600 MHz, methanol-d_4): δ 6.73 (d, J = 8.7 Hz, 1H), 6.40 (d, J = 2.7 Hz, 1H), 6.34 (s, 1H), 6.24 (dd, J = 8.7, 2.7 Hz, 1H), 6.03 (s, 1H), 2.09 (s, 3H); ^{13}C NMR (150 MHz, methanol-d_4): δ 155.8, 150.7, 148.2, 147.4, 138.5, 134.1, 129.9, 122.5, 111.8, 109.9, 107.6, 105.1, 21.2 (**Table S9.9**; **Fig. S17.91-17.94**); HRESI-MS: *m/z* 247.06209 [M-H]$^-$ (calcd for $C_{13}H_{11}O_5$, 247.0606) (**Fig. S16.24**).

1.5.10 Compounds 17a/b

30 mg of compound **17** was fed to the *dpeH* gene-harboring *A. oryzae* transformant. After the fermentation, the crude extract was directly purified by semi-preparative HPLC, eluting with MeOH/H_2O (0-25 min, 51:49, v/v) to yield compounds **17b** (2.0 mg, t_R=14.3 min) and **17a** (1.5 mg, t_R=21.7 min).

17a: cordyol C.[16] Pale brown solid; maximum UV absorption wavelength 279 nm (**Fig. S15**); ^{1}H NMR (600 MHz, methanol-d_4): δ 6.45 (d, J = 1.4 Hz, 1H), 6.29 (br s, 1H), 6.25 (br s, 1H), 6.21 (d, J = 1.3 Hz, 1H), 6.16 (t, J = 2.0 Hz, 1H), 2.21 (s, 3H), 2.15 (s, 3H); ^{13}C NMR (150 MHz, methanol-d_4): δ 160.7, 159.5, 147.8, 145.4, 141.5, 136.1, 130.1, 113.6, 113.2, 111.3, 110.4, 102.8, 21.8, 21.1 (**Table S9.10**; **Fig. S17.99-17.102**); HRESI-MS: *m*/*z* 245.08295 [M-H]$^-$ (calcd for $C_{14}H_{13}O_4$, 245.0814) (**Fig. S16.26**).

17b: violaceol I.[16] Brown solid; maximum UV absorption wavelength 282 nm (**Fig. S15**); ^{1}H NMR (500 MHz, methanol-d_4): δ 6.40 (s, 2H), 6.15 (s, 2H), 2.12 (s, 6H) (**Fig. S17.103**); HRESI-MS: *m*/*z* 261.07803 [M-H]$^-$ (calcd for $C_{14}H_{13}O_5$, 261.0763) (**Fig. S16.27**).

2 SI Tables

Table S1. Plasmids used in this study

Plasmids	Description	Source/Ref.
YEpADH2p-FLAG-URA	*URA3*, *ADH2p*, *ADH2t*, *AmpR*	1
YEpADH2p-FLAG-TRP	*TRP1*, *ADH2p*, *ADH2t*, *AmpR*	1
YEpADH2p-FLAG-LEU	*LEU2*, *ADH2p*, *ADH2t*, *AmpR*	1
pET-28a	*KanR*, *T7 promoter*	ZOMANBIO
pTAex3	*argB*, *PamyB*, *TamyB*, *AmpR*	3
pUSA	*sC*, *PamyB*, *TamyB*, *AmpR*	3
pUNA	*niaD*, *PamyB*, *TamyB*, *AmpR*	3
pAdeA	*adeA*, *AmpR*	3
YEpPreu6	a 6,618 bp fragment of *preu6* (*dpeA*) (SAT-KS-AT-PT-ACP1-ACP2-TE) was inserted in YEpADH2p-FLAG-URA	2
YEpTRP(DpeB)	a 1,557 bp fragment of *dpeB* was inserted in YEpADH2p-FLAG-TRP	This study
YEpTRP(DpeC)	a 765 bp fragment of *dpeC* was inserted in YEpADH2p-FLAG-TRP	This study
YEpTRP(DpeD)	a 939 bp fragment of *dpeD* was inserted in YEpADH2p-FLAG-TRP	This study
YEpTRP(DpeE)	an 882 bp fragment of *dpeE* was inserted in YEpADH2p-FLAG-TRP	This study
YEpTRP(DpeF)	a 1,296 bp fragment of *dpeF* was inserted in YEpADH2p-FLAG-TRP	This study
YEpTRP(DpeH)	a 1,161 bp fragment of *dpeH* was inserted in YEpADH2p-FLAG-TRP	This study
YEpTRP(DpeI)	a 1,041 bp fragment of *dpeI* was inserted in YEpADH2p-FLAG-TRP	This study
YEpLEU(DpeB-ΔXhoI)	a 1,557 bp fragment of *dpeB* with *Xho*I mutation was inserted in YEpADH2p-FLAG-LEU	This study
YEpLEU(DpeD-ΔXhoI)	a 939 bp fragment of *dpeD* with *Xho*I mutation was inserted in YEpADH2p-FLAG-LEU	This study
YEpLEU(DpeB+D)	a 1,557 bp fragment of *dpeB* and a 939 bp fragment of *dpeD* were inserted in YEpADH2p-FLAG-LEU	This study
YEpLEU(DpeC)	a 765 bp fragment of *dpeC* was inserted in YEpADH2p-FLAG-LEU	This study
YEpLEU(DpeB+D+C)	a 1,557 bp fragment of *dpeB* and a 939 bp fragment of *dpeD*, and a 765 bp fragment of *dpeC* were inserted in YEpADH2p-FLAG-LEU	This study
YEpLEU[(DpeB+D+C)-ΔBamHI]	YEpLEU(DpeB+D+C) with *Bam*HI mutation	This study
YEpLEU(DpeE-ΔBglII)	an 882 bp fragment of *dpeE* with *Bgl*II mutation was inserted in YEpADH2p-FLAG-LEU	This study
YEpLEU(DpeB+D+C+E)	a 1,557 bp fragment of *dpeB* and a 939 bp fragment of *dpeD*, and a 765 bp fragment of *dpeC*, and an 882 bp fragment of *dpeE* were inserted in YEpADH2p-FLAG-LEU	This study

YEpLEU[(DpeB+D+C+E)-ΔBamHI]	YEpLEU(DpeB+D+C+E) with *Bam*HI mutation	This study
YEpLEU(DpeF-ΔBglII, SalI)	a 1,296 bp fragment of *dpeF* with *Bgl*II and SalI mutations was inserted in YEpADH2p-FLAG-LEU	This study
YEpLEU(DpeB+D+C+E+F)	a 1,557 bp fragment of *dpeB* and a 939 bp fragment of *dpeD*, and a 765 bp fragment of *dpeC*, and an 882 bp fragment of *dpeE*, and a 1,296 bp fragment of *dpeF* were inserted in YEpADH2p-FLAG-LEU	This study
YEpLEU(DpeH-ΔBglII)	a 1,161 bp fragment of *dpeH* with *Bgl*II mutation was inserted in YEpADH2p-FLAG-LEU	This study
YEpLEU(DpeB+D+C+E+F+H)	a 1,557 bp fragment of *dpeB* and a 939 bp fragment of *dpeD*, and a 765 bp fragment of *dpeC*, and an 882 bp fragment of *dpeE*, and a 1,296 bp fragment of *dpeF*, and a 1,161 bp fragment of *dpeH* were inserted in YEpADH2p-FLAG-LEU	This study
pUSA(DpeA)	a 6,618 bp fragment of *dpeA* (SAT-KS-AT-PT-ACP1-ACP2-TE) was inserted in pUSA	This study
pTAex3(DpeB)	a 1,557 bp fragment of *dpeB* was inserted in pTAex3	This study
pTAex3(DpeC)	a 765 bp fragment of *dpeC* was inserted in pTAex3	This study
pTAex3(DpeD)	a 939 bp fragment of *dpeD* was inserted in pTAex3	This study
pTAex3(DpeE)	an 882 bp fragment of *dpeE* was inserted in pTAex3	This study
pTAex3(DpeF)	a 1,296 bp fragment of *dpeF* was inserted in pTAex3	This study
pTAex3(DpeH)	a 1,161 bp fragment of *dpeH* was inserted in pTAex3	This study
pTAex3(DpeI)	a 1,041 bp fragment of *dpeI* was inserted in pTAex3	This study
pTAex3(DpeE+F)	an 882 bp fragment of *dpeE* and a 1,296 bp fragment of *dpeF* were inserted in pTAex3	This study
pUSA(DpeA+C)	a 6,618 bp fragment of *dpeA* (SAT-KS-AT-PT-ACP1-ACP2-TE) and a 765 bp fragment of *dpeC* were inserted in pUSA	This study
pAdeA(DpeB)	a 1,557 bp fragment of *dpeB* was inserted in pAdeA	This study
pAdeA(DpeB+D)	a 1,557 bp fragment of *dpeB* and a 939 bp fragment of *dpeD* were inserted in pAdeA	This study
pUNA(DpeC)	a 765 bp fragment of *dpeC* was inserted in pUNA	This study
pUNA(DpeH)	a 1,161 bp fragment of *dpeH* was inserted in pUNA	This study
pUNA(DpeH+I)	a 1,161 bp fragment of *dpeH* and a 1,041 bp fragment of *dpeI* were inserted in pUNA	This study
pTAex3(AN7912)	a 1,110 bp fragment of *AN7912* was inserted in pTAex3	This study
pTAex3(M1)	a 177 bp fragment of *dpeH* (M^1-K^{59}) and a 936 bp fragment of *AN7912* (E^{59}-P^{369}) were inserted in pTAex3	This study
pTAex3(M2)	a 1,029 bp fragment of *AN7912* (M^1-L^{343}) and a 123 bp fragment of *dpeH* (L^{346}-Q^{386}) were inserted in pTAex3	This study
pTAex3(M3)	a 177 bp fragment of *dpeH* (M^1-K^{59}), an 855 bp fragment of *AN7912* (E^{59}-L^{343}), and a 126 bp fragment of *dpeH* (L^{346}-Q^{386}) were inserted in pTAex3	This study
pTAex3(M4)	a 1,035 bp fragment of *dpeH* (M^1-L^{345}) and an 81 bp fragment of *AN7912* (L^{344}-P^{369}) were inserted in pTAex3	This study

pTAex3(M5)	a 1,038 bp fragment of *dpeH* (M^{1}-L^{345}) was inserted in pTAex3	This study
pTAex3(M6)	a 1,032 bp fragment of *AN7912* (M^{1}-L^{343}) was inserted in pTAex3	This study
pTAex3(M7)	a 1,029 bp fragment of *AN7912* (M^{1}-L^{343}), a 72 bp fragment of *dpeH* (L^{346}-P^{369}), and a 39 bp fragment of *dpeH* (S^{375}-Q^{386}) were inserted in pTAex3	This study
pTAex3(M8)	a 1,029 bp fragment of *AN7912* (M^{1}-L^{343}) and a 114 bp fragment of *dpeH* (L^{346}-C^{382}) were inserted in pTAex3	This study
pTAex3(M9)	a 1,029 bp fragment of *AN7912* (M^{1}-L^{343}) and a 93 bp fragment of *dpeH* (L^{346}-S^{375}) were inserted in pTAex3	This study
pTAex3(M10)	a 1,029 bp fragment of *AN7912* (M^{1}-L^{343}) and a 75 bp fragment of *dpeH* (L^{346}-P^{369}) were inserted in pTAex3	This study
pET28a(DpeE)	an 882 bp fragment of *dpeE* was inserted in pET28a	This study
pET28a(DpeI)	a 1,041 bp fragment of *dpeI* was inserted in pET28a	This study

Table S2. DPE-producing fungi reported in the literature, and genome sequence assemblies used for comparative genomics

**Species**		**Genome, Source**	**Ref.**
Arthrinium	*arundinis*	____[a]	[19]
Aspergillus	*carneus*	____[a]	[20]
	falconensis	*A. falconensis* CBS 271.91-Gp0108111, JGI	[21]
	flavipes	____[a]	[22]
	flavus	*A. flavus* NRRL3357-GCA_014117465.1[b], GenBank	[23]
	jensenii	*A. jensenii* IBT 32279-Gp0312232, JGI	[24]
	nidulans	*A. nidulans* FGSC A4-GCA_000011425.1, GenBank	[25]
	sulphureus	*A. sulphureus* CBS 550.65-Gp0108227, JGI	[26-27]
	sydowii	*A sydowii* CBS 593.65-GCA_001890705.1, GenBank	[28-29]
	tabacinus	____[a]	[30]
	tennesseensis	*A. tennesseensis* IBT 32283-Gp0312234, JGI	[31]
	terreus	*A. terreus* ATCC 20542-GCA_016808415.1, GenBank	[32-33]
	unguis	*A. unguis* F6_8S_P_4A-GCA_018408605.1, GenBank	[34-35]
	versicolor	*A. versicolor* CBS 583.65-GCA_001890125.1, GenBank	[36-42]
Boeremia	*exigua*	*B. exigua* MPI-SDFR-AT-0100-GCA_020726555.1, GenBank	[43]
Claviceps	*purpurea*	*C. purpurea* 20.1-GCA_000347355.1, GenBank	[44]
Coniothyrium	sp.	*C. glycines* Pg-43-GCA_025742365.1[b], GenBank	[45]
Cordyceps	sp.	*Cordyceps* sp. RAO-2017-GCA_002591385.1[b], GenBank	[46]
Corynespora	*cassiicola*	*C. cassiicola* Philippines-GCA_003016335.1, GenBank	[10, 47-48]
Dendrospora	*tenella*	____[a]	[49]
Diplodia	*corticola*	*D. corticola* CBS 112549-GCA_001883845.1[b], GenBank	[50-51]
Diploicia	*canescens*	____[a]	[52]
Epichloe	*bromicola*	*E. bromicola* ATCC 200750-GCA_000729905.1[b], GenBank	[53]
Epicoccum	*sorghinum*	*E. sorghinum* BS2-1-GCA_020272525.1, GenBank	[54]
Geomyces	sp.	____[a]	[55]
Isaria	*felina*	*I. feline* SYSU-MS7908-GCA_016490725.1, GenBank	[27]
Lobaria	*orientalis*	____[a]	[56]
Metarhizium	*anisopliae*	*M. anisopliae* JEF-290-GCA_013305495.1[b], GenBank	[57]
Neosartorya	*spinosa*	____[a]	[58]

Parmotrema	*indicum*	——[a]	[59]
	praesorediosum	——[a]	[60]
Penicillium	*chrysogenum*	*P. chrysogenum* IBT 35668-GCA_028827035.1[b], GenBank	[61]
	griseofulvum	*P. griseofulvum* PG3-GCA_001561935.1, GenBank	[62]
Pestalotiopsis	*fici*	*P. fici* CGMCC3.15140-GCA_000516985.1, GenBank	[63-64]
Phoma	sp.	*Phoma* sp. XZ068-GCA_004835665.1[b], GenBank	[65]
Phomopsis	*fukushii*	——[a]	[66]
Preussia	*isomera*	*P. isomera* XL1326-our lab	This study
Pseudogymnoascus	sp.	*Pseudogymnoascus* sp. WSF 3629-GCA_001662585.1, GenBank	[67]
Pseudopithomyces	*maydicus*	*P. maydicus* SBW1-GCA_026873275.1, GenBank	[68]
Simplicillium	sp.	*Simplicillium* sp. C3G150-2-GCA_022702485.1, GenBank	[69]
Spiromastix	sp.	*Spiromastix* sp. SCSIO F190-GCA_014805645.1[b], GenBank	[70]
Steganospora	sp.	——[a]	[71]
Stereocaulon	*halei*	——[a]	[72]
Talaromyces	*thailandensis*	*T. thailandensis* OC-R06-P5-GCA_019828575.1[b], GenBank	[73]
Tritirachium	sp.	——[a]	[74]
Verticillium	sp.	*V. dahliae* VdLs.17-GCA_000150675.2[b], GenBank	[75]

[a] No genome sequence data is available.

[b] No DPE biosynthetic gene cluster was identified in the genome by comparative genomics.

Table S3. Putative DPE gene clusters identified in 20 species of fungi

Species		Location of the DPE clusters
Aspergillus	*falconensis*	jgi|Aspfalc1|177525|gm1.4639_g - jgi|Aspfalc1|177531|gm1.4645_g
	jensenii	jgi|Aspjen1|460397|gm4.11203_g - jgi|Aspjen1|460404|gm4.11210_g
	nidulans	AN7908 - AN7915, XP_681177 - XP_681184
	sulphureus	jgi|Aspsul1|259232|gm1.2267_g - jgi|Aspsul1|259240|gm1.2275_g
	sydowii	XP_040699613 - XP_040699620
	tennesseensis	jgi|Aspten1|739230|gm1.9761_g - jgi|Aspten1|739237|gm1.9768_g
	terreus	KAG2416263 - KAG2416270
	unguis	GCA_018408605|NODE_3|1271263 - 1289478
	versicolor	XP_040670517 - XP_040670524
Boeremia	*exigua*	XP_046000144 - XP_046000150
Claviceps	*purpurea*	CCE33499 - CCE33502
Corynespora	*cassiicola*	PSN66428 - PSN66433
Epicoccum	*sorghinum*	GCA_020272525|tig00000001|2535617 - 2568980
Isaria	*felina*	GCA_020726555|NODE_28|96883 - 124422
Penicillium	*griseofulvum*	XP_040651868 - XP_040651880
Pestalotiopsis	*fici*	PtaA - PtaM, AGO59035 - AGO59052
Preussia	*isomera*	DpeA - DpeI, PP925597
Pseudogymnoascus	sp.	OBT45891 - OBT45897
Pseudopithomyces	*maydicus*	GCA_026873275|contig_39|1058177 - 1079858
Simplicillium	sp.	GCA_022702485|Scaffold7|2258920 - 2278980

Table S4. DPE compounds produced by species with a **Type I** biosynthetic gene cluster

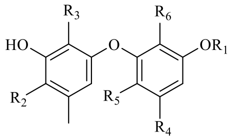

I. diorcinol: $R_1=R_2=R_3=R_5=R_6=H$, $R_4=CH_3$

II. cordyol E: $R_1=CH_3$, $R_2=R_3=R_5=R_6=H$, $R_4=CH_3$

III. 3-hydroxydiorcinol: $R_1=R_3=R_5=R_6=H$, $R_2=OH$, $R_4=CH_3$

IV. 7-ethyldiorcinol: $R_1=R_2=R_3=R_5=R_6=H$, $R_4=CH_2CH_3$

V. 4-carboxydiorcinal: $R_1=R_3=R_5=R_6=H$, $R_2=COOH$, $R_4=CH_3$

VI. 4-methoxycarbonyl diorcinol: $R_1=R_3=R_5=R_6=H$, $R_2=COOCH_3$, $R_4=CH_3$

VII. 4-carbethoxydiorcinal: $R_1=R_3=R_5=R_6=H$, $R_2=COOCH_2CH_3$, $R_4=CH_3$

VIII. cordyol C: $R_1=R_2=R_5=R_6=H$, $R_3=OH$, $R_4=CH_3$

IX. gerfelin: $R_1=R_3=R_5=H$, $R_2=COOH$, $R_4=CH_3$, $R_6=OH$

X. methylgerfelin: $R_1=R_3=R_5=H$, $R_2=COOCH_3$, $R_4=CH_3$, $R_6=OH$

XI. pseudopithoether A: $R_1=R_2=R_3=R_6=H$, $R_4=CH_3$, $R_5=COOCH_3$

XII. aspergilol E: $R_1=R_3=R_5=H$, $R_2=COOCH_3$, $R_4=CH_3$, $R_6=OH$

XIII. violaceol I: $R_1=R_2=R_5=H$, $R_3=R_6=OH$, $R_4=CH_3$

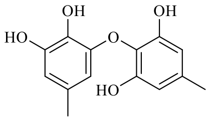

XXIII. violaceol II

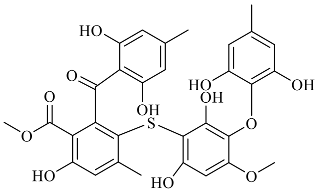

XXIV. diphenyl ketone-diphenyl ether dimer

XIV. diorcinol B: $R_1=R_2=R_3=R_6=H$, $R_4=CH_3$, $R_5=$ 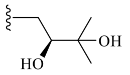

XV. 9-acetyldiorcinol B: $R_1=R_2=R_3=R_6=H$, $R_4=CH_3$, $R_5=$ 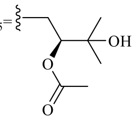

XVI. diorcinol C: $R_1=R_2=R_3=R_6=H$, $R_4=CH_3$, $R_5=$ 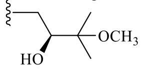

XVII. diorcinol D: $R_1=R_2=R_3=R_6=H$, $R_4=CH_3$, $R_5=$ 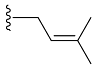

XVIII. diorcinol E: $R_1=R_2=R_3=R_6=H$, $R_4=CH_3$, $R_5=$ 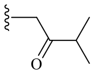

XIX. diorcinol F: $R_1=R_2=R_5=H$, $R_3=OH$, $R_4=CH_3$, $R_6=OCH_3$

XX. diorcinol G: $R_1=R_5=R_6=H$, $R_2=R_3=$ 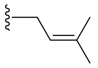 , $R_4=CH_3$

XXI. diorcinol I: $R_1=R_2=R_3=R_5=H$, $R_4=CH_3$, $R_6=$ 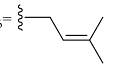

XXII. diorcinol J: $R_1=R_2=R_3=R_6=H$, $R_4=CH_3$, $R_5=$ 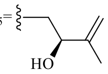

**Species**		**Structures**
Aspergillus	*falconensis*	**I** [21]
	jensenii	**I, XIII, XXIII** [24]
	nidulans	**I, V, IX** [25]
	sulphureus	**I** [26]; **XIV, XVI, XVII, XVIII, XXII** [27]
	sydowii	**I** [28-29]; **III, IV, V, VI, XII, XIII, XIX** [29], **VIII** [28]; etc.*
	tennesseensis	**XIV, XV, XVI, XVII, XVIII, XXII** [31]
	versicolor	**I** [36-42]; **II** [36,40]; **V, VII, X** [36]; **VI** [36,41-42]; **VIII** [36,39]; **XIV, XVI** [37-38,41]; **XVII** [37-39]; **XVIII** [37,41]; **XIX, XX, XXI** [39]; **XXII** [41]; **XXIII** [39,42]; etc.*
Claviceps purpurea		**I, V, IX** [44]
Isaria feline		**XIV, XVI, XVII, XVIII, XXII** [27]
Pseudogymnoascus sp.		**XXIV** [67]
Pseudopithomyces maydicus		**XI** [68]
Simplicillium sp.		**I** [69]

* etc.: Several additional DPEs have been detected from these species

Table S5. DPE compounds produced by species with a **Type II** biosynthetic gene cluster

Species	Structures
Aspergillus terreus	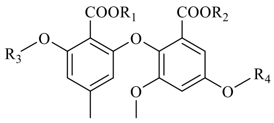 **dicarboxylic acid** [33]**:** R_1=R_2=R_3=R_4=H<br>**asterric acid** [32-33]**:** R_1=R_3=R_4=H, R_2=CH_3<br>**methyl asterrate** [33]**:** R_1=R_2=CH_3, R_3=R_4=H<br>**methyl di-*O*-methylasterrate** [33]**:** R_1=R_2=R_3=R_4=CH_3
Penicillium griseofulvum	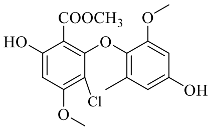<br>**4-chloro-7,4′-dihydroxy-5,2′-dimethoxy-2-methylformate-6′-methybenzophone** [62]
Pestalotiopsis fici	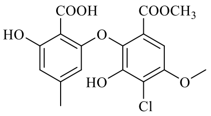<br>**pestheic acid** [63]

Table S6. DPE compounds produced by species with a **Type III** biosynthetic gene cluster

**Species**	**Structures**
Aspergillus unguis	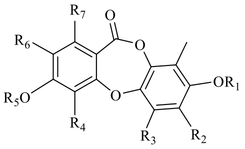<br>**asperunguissidone A** [35]: R_1=R_2=R_4=R_5=R_6=H, R_7=CH_3, R_3= 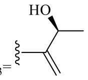<br>**asperunguissidone B** [35]: R_1=R_2=R_4=R_5=H, R_6=Cl, R_7=CH_3, R_3= 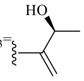<br>**unguinol** [35]: R_1=R_2=R_4=R_5=R_6=H, R_7=CH_3, R_3= **2-chlorounguinol** [34-35]: R_1=R_2=R_4=R_5=H, R_6=Cl, R_7=CH_3, R_3= 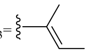<br>**aspergillusidone C** [34-35]: R_1=R_4=R_5=H, R_2=R_6=Cl, R_7=CH_3, R_3= 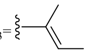<br>**nornidulin** [34-35]: R_1=R_5=H, R_2=R_4=R_6=Cl, R_7=CH_3, R_3= 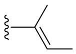<br>**nidulin** [34-35]: R_1=R_7=CH_3, R_2=R_4=R_6=Cl, R_5=H, R_3= 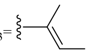<br>**aspergillusidone A** [34-35]: R_1=R_4=R_5=R_6=H, R_2=COOH, R_7=CH_3, R_3= 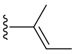<br>**aspergisidone** [35]: R_1=R_5=R_6=H, R_2=COOH, R_4=CH_3, R_3=R_7= 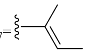<br>**folipastatin** [35]: R_1=R_2=R_5=R_6=H, R_4=CH_3, R_3=R_7= 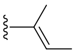<br>**emeguisin A** [35]: R_1=R_5=R_6=H, R_2=Cl, R_4=CH_3, R_3=R_7= 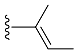<br>**emeguisin B** [35]: R_1=R_6=H, R_2=Cl, R_4=R_5=CH_3, R_3=R_7= 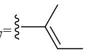<br>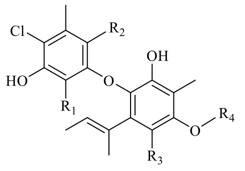<br>**aspergillusether A** [34]: R_1= R_3=Cl, R_2=$COOCH_3$, R_4=CH_3<br>**aspergillusether C** [35]: R_1=R_3=R_4=H, R_2=$COOCH_3$<br>**aspergillusether D** [35]: R_1=R_4=H, R_2=$COOCH_3$, R_3=Cl<br>**aspergillusether E** [35]: R_1=R_2=R_4=H, R_3=Cl<br>**aspergillusether F** [35]: R_1=R_3=Cl, R_2=R_4=H
Boeremia exigua	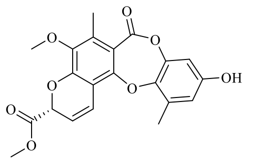 **boremexin A** [43]<br>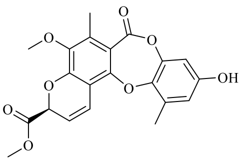 **boremexin B** [43]<br>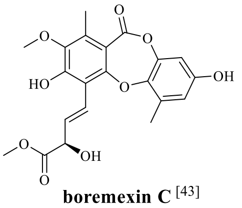 **boremexin C** [43]<br>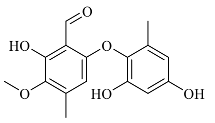 **boremexin D** [43]<br>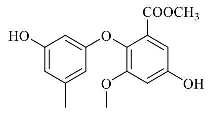 **boremexin E** [43]<br>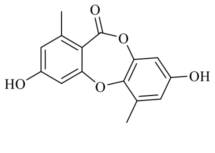 **corynesidone A (3)** [43]<br>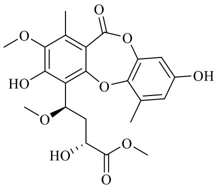 **curdepsidone B** [43]<br>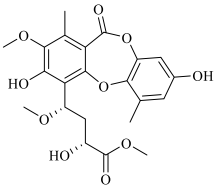 **curdepsidone C** [43]<br>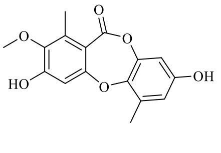 **curdepsidone F** [43]
Corynespora cassiicola	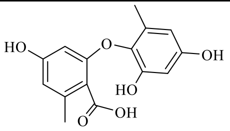 **corynether A (4)** [10, 47-48]<br>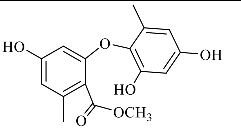 **corynether B** [10]<br>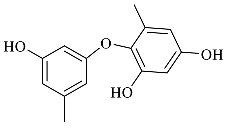 **diaryl ether (5)** [47]<br>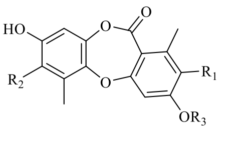<br>**corynesidone A (3)** [10, 47-48]: R_1=R_2=R_3=H<br>**corynesidone B** [47]: R_1=OH, R_2=COOH, R_3=H<br>**corynesidone C** [48]: R_1=OH, R_2=R_3=H<br>**corynesidone D (2)** [48]: R_1=R_3=H, R_2=COOH<br>**corynesidone E** [10]: R_1=OH, R_2=H, R_3=CH_3

Epicoccum sorghinum	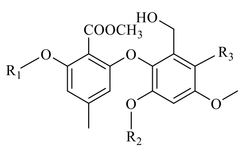 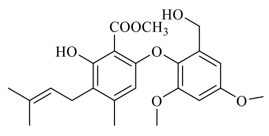 **epicoccether D** [54] 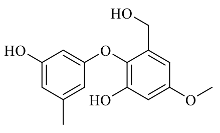 **epicoccether H** [54] 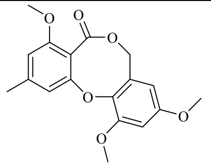 **epicoccether I** [54]<br>**epicoccether A** [54]**:** R_1=R_3=H, R_2= 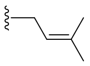<br>**epicoccether B** [54]**:** R_1=R_2= 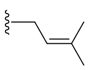 , R_3=H<br>**epicoccether C** [54]**:** R_1= 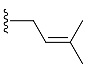 , R_2=CH_3, R_3=H<br>**epicoccether E** [54]**:** R_1=R_2=R_3= 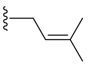<br>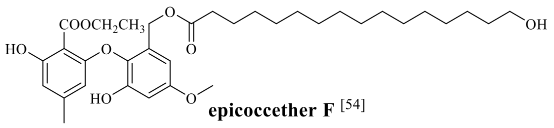 **epicoccether F** [54]<br>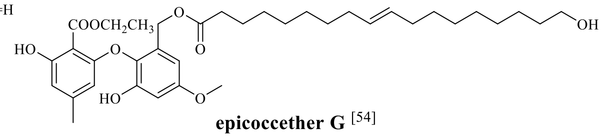 **epicoccether G** [54]
Preussia sp.	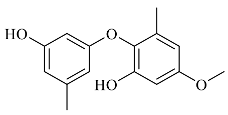 **cyperine (6)** [11, 76]<br>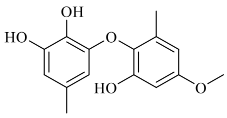 **5-methoxy-3,5'-dimethyl-2,3'-oxybiphenyl-1,1',2'-triol (7)** [76]<br>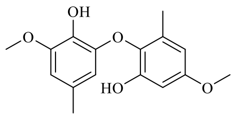 **1',5-dimethoxy-3,5'-dimethyl-2,3'-oxybiphenyl-1,2'-diol** [76]

Table S7. Antibacterial activities of compounds **1-8** (MIC, μg/mL)[a]

Strains	**1**	**2**	**3**	**4**	**5**	**6**	**7**	**8**	Ciprofloxacin
Carbapenems-resistant *Pseudomonas aeruginosa*	>100	>100	>100	>100	>100	>100	25	25	0.78
Carbapenems-resistant *Acinetobacter baumannii*	>100	>100	50	>100	>100	>100	25	12.5	50
Carbapenems-resistant *Klebsiella pneumoniae*	>100	>100	>100	>100	>100	>100	12.5	12.5	>100
Carbapenems-resistant *Escherichia coli*	>100	>100	50	>100	>100	>100	50	50	12.5
Multidrug-resistant *Staphylococcus epidermidis*	>100	>100	25	>100	>100	>100	12.5	6.25	12.5
Multidrug-resistant *Enterococcus faecalis*	>100	>100	12.5	>100	>100	>100	12.5	12.5	0.78
Multidrug-resistant *Enterococcus faecium*	>100	>100	25	>100	>100	>100	25	12.5	0.78
Methicillin-resistant *Staphylococcus aureus*	>100	>100	12.5	>100	>100	>100	25	6.25	0.78

[a] MIC – minimum inhibitory concentration.

Table S8. Primers used in this study

Name	Sequence (5′→3′)	PCR product (Size in bp)	For construction of
06F-DpeB-F	GACTACAAAGACGATGACGACAAGCTTCATATGGAAAGCATCAGTATCCAACGCATG	DpeB-06F (1,617 bp)	YEpTRP(DpeB)
06F-DpeB-R	AGTGATGGTGATGGTGATGTCCGTTTAAACTCAAGCATATATCCGGTCCACT		
06F-DpeC-F	GACTACAAAGACGATGACGACAAGCTTCATATGAGTGACTCGTGCTGCCT	DpeC-06F (825 bp)	YEpTRP(DpeC)
06F-DpeC-R	AGTGATGGTGATGGTGATGTCCGTTTAAACCTACACGTTATCCGCATGTAACC		
06F-DpeD-F	GACTACAAAGACGATGACGACAAGCTTCATATGGCACAATCCACCCGCATTG	DpeD-06F (999 bp)	YEpTRP(DpeD)
06F-DpeD-R	AGTGATGGTGATGGTGATGTCCGTTTAAACTCAACACCCCTTCAATCTAGG		
06F-DpeE-F	GACTACAAAGACGATGACGACAAGCTTCATATGTCGCCATCATTCCATCCAGCCACCTC	DpeE-06F (942 bp)	YEpTRP(DpeE)
06F-DpeE-R	AGTGATGGTGATGGTGATGTCCGTTTAAACCTAAATGTTTCTTTGCGTCC		
06F-DpeF-F	GACTACAAAGACGATGACGACAAGCTTCATATGTCGAACCTGAGGGCACTC	DpeF-06F (1,356 bp)	YEpTRP(DpeF)
06F-DpeF-R	AGTGATGGTGATGGTGATGTCCGTTTAAACTCAACCCCTCCAGACACATTC		
06F-DpeH-F	GACTACAAAGACGATGACGACAAGCTTCATATGTTTCGCTCCATACCTTTGGTGGC	DpeH-06F (1,221 bp)	YEpTRP(DpeH)
06F-DpeH-R	AGTGATGGTGATGGTGATGTCCGTTTAAACCTACTGGTAGATATAGCAG		
06F-DpeI-F	GACTACAAAGACGATGACGACAAGCTTCATATGTCGAACCAAGGTGCATACCTC	DpeI-06F (1,101 bp)	YEpTRP(DpeI)
06F-DpeI-R	AGTGATGGTGATGGTGATGTCCGTTTAAACTTAGACCTCGACAACCAACTTGGTACC		
DpeB-ΔXhoI-R	GGGTGGTAGCACACGAGGCATGCTTCCAGGAGT	DpeB-ΔXhoI-1 (1,197 bp)	YEpLEU(DpeB-ΔXhoI)
DpeB-ΔXhoI-F	TGGAAGCATGCCTCGTGTGCTACCACCCCAAGGTCAAGT	DpeB-ΔXhoI-2 (448 bp)	
DpeD-ΔXhoI-R	GTTGTGCTTCTCCAGCTCTTCCCATACAGGAACA	DpeD-ΔXhoI-1 (486 bp)	YEpLEU(DpeD-ΔXhoI)
DpeD-ΔXhoI-F	GTATGGGAAGAGCTGGAGAAGCACAACGCGGTTGT	DpeD-ΔXhoI-2 (540 bp)	
DpeBDC-BlpI-F	GAGGGGCCTGCGCAGTTGTTGGAGCATTTT	(DpeB+D+C)-ΔBamHI-1 (1,524 bp)	YEpLEU[(DpeB+D+C)-ΔBamHI]
DpeBDC-ΔBamHI-R	TCGTAAACACCGGGTCCATCTCCGGCGCCACAAT		
DpeBDC-ΔBamHI-F	CGCCGGAGATGGACCCGGTGTTTACGAGGGAGTT	(DpeB+D+C)-ΔBamHI-2 (426 bp)	
DpeBDC-XhoI-R	CTATAGGGCGAATTGGGTACCGG		
DpeE-ΔBglII-R	TTCTTCCCTCAGGTCTCTTGAGAATCGGTCCAA	DpeE-ΔBglII-1 (852 bp)	YEpLEU(DpeE-ΔBglII)
DpeE-ΔBglII-F	CGATTCTCAAGAGACCTGAGGGAAGAATTGGAAACG	DpeE-ΔBglII-2 (117 bp)	
DpeBDCE-PstI-F	CCTGGAGCGGGAACAAAACAGAAACAAGCTCTGCAGTCGCTTGCAGAGCTTGTAAAATCTGGTGGCTGGATACAACTCATCGAAGCGG	(DpeB+D+C+E)-ΔBamHI (700 bp)	YEpLEU[(DpeB+D+C+E)-ΔBamHI]
DpeF-ΔBglII-1R	TAAGATAGCGAGGTCTTGCGCTGCGCTGTACAGTG	DpeF-ΔBglII,SalI-1 (225 bp)	YEpLEU(DpeF-ΔBglII,SalI)
DpeF-ΔBglII-1F	CGCAGCGCAAGACCTCGCTATCTTAGCGATTGGTCCAGT	DpeF-ΔBglII,SalI-2 (556 bp)	
DpeF-ΔSalI-R	GTCGTAGCAGTCCACCACATGGTCCGCCTTCATAG		
DpeF-ΔSalI-F	GGACCATGTGGTGGACTGCTACGACTGGAAAGGGCTT	DpeF-ΔBglII,SalI-3 (505 bp)	
DpeF-ΔBglII-2R	CGCCAGTGCGAATATCTCTTTCCACTCCTCTACAG		
DpeF-ΔBglII-2F	GTGGAAAGAGATATTCGCACTGGCGGATTCGGGTTTTG	DpeF-ΔBglII,SalI-4 (145 bp)	

DpeH-ΔBglII-R	CAGTGCACTTAGGTCTCCCCATTCCTTGCGCACGGCAAGT	DpeH-ΔBglII-1 (231 bp)	YEpLEU(DpeH-ΔBglII)
DpeH-ΔBglII-F	AAGGAATGGGGAGACCTAAGTGCACTGCAACGTTTGG	DpeH-ΔBglII-2 (1,017 bp)	
pUSA-DpeA-1F	ACAGCAAGCTCCGAATTCGAGCTCGGTACCATGTCTAATTCTACACGTGACTATCCC	DpeA-pUSA-1 (1,269 bp)	pUSA(DpeA)
pUSA-DpeA-1R	GTCGGTACCTTTGGAGATCAGGTC		
pUSA-DpeA-2F	TAGCCATGATGACAGGCGTGAGGTT	DpeA-pUSA-3 (1,950 bp)	
pUSA-DpeA-2R	CTTCACGAGCTACTACAGATCCCCGGGTACCTCAAGCGTTCTGAATCAAAAAGTC		
pTAex3-DpeB-F	TCCGAATTCGAGCTCGGTACATGGAAAGCATCAGTATCCAAC	DpeB-pTAex3 (1,597 bp)	pTAex3(DpeB)
pTAex3-DpeB-R	TACTACAGATCCCCGGGTACTCAAGCATATATCCGGTCCAC		
pTAex3-DpeC-F	TCCGAATTCGAGCTCGGTACATGAGTGACTCGTGCTGCCTGAAAG	DpeC-pTAex3 (805 bp)	pTAex3(DpeC)
pTAex3-DpeC-R	TACTACAGATCCCCGGGTACCTACACGTTATCCGCATGTA		
pTAex3-DpeD-F	TCCGAATTCGAGCTCGGTACATGGCACAATCCACCCGCATTG	DpeD-pTAex3 (979 bp)	pTAex3(DpeD)
pTAex3-DpeD-R	TACTACAGATCCCCGGGTACTCAACACCCCTTCAATCTAG		
pTAex3-DpeE-F	TCCGAATTCGAGCTCGGTACATGTCGCCATCATTCCATCCAGC	DpeE-pTAex3 (922 bp)	pTAex3(DpeE)
pTAex3-DpeE-R	TACTACAGATCCCCGGGTACCTAAATGTTTCTTTGCGTCC		
pTAex3-DpeF-F	TCCGAATTCGAGCTCGGTACATGTCGAACCTGAGGGCACTC	DpeF-pTAex3 (1,336 bp)	pTAex3(DpeF)
pTAex3-DpeF-R	TACTACAGATCCCCGGGTACTCAACCCCTCCAGACACATT		
pTAex3-DpeH-F	TCCGAATTCGAGCTCGGTACATGTTTCGCTCCATACCTTT	DpeH-pTAex3 (1,201 bp)	pTAex3(DpeH)
pTAex3-DpeH-R	TACTACAGATCCCCGGGTACCTACTGGTAGATATAGCAGT		
pTAex3-DpeI-F	TCCGAATTCGAGCTCGGTACATGTCGAACCAAGGTGCATA	DpeI-pTAex3 (1,081 bp)	pTAex3(DpeI)
pTAex3-DpeI-R	TACTACAGATCCCCGGGTACTTAGACCTCGACAACCAACTTG		
PamyB-DpeF-TamyB-F	GGGTTACCCCATCATGGTGTTTTGATCATTT	PamyB-DpeF-TamyB (2,208 bp)	pTAex3(DpeE+F)
PamyB-DpeF-TamyB-R	GGGTAACCGTAAGATACATGAGCTTCGGTG		
pUSA-DpeA-NdeI-F	CTTCCGGATGGCTCGAGTTTTTCAGCAAGATATCATTCATGGCACGAAGGACGTCATC	DpeA+C-1 (436 bp)	pUSA(DpeA+C)
pUSA-DpeA-TAA-R	GACTCTAGAGGATCCTTTCCTATAATAGACTAGC		
PamyB-DpeC-TamyB-F	CGCTAGTCTATTATAGGAAAGGATCCTCTAGAGTCCCATCATGGTGTTTTGATCATT	DpeA+C-2 (1,731 bp)	
PamyB-DpeC-TamyB-R	CGAGGAGCCATATTTTGGATTTTTATATCCAAGATGTAAGATACATGAGCTTCGGTG		
pUSA-DpeA-sC-F	ATCTTGGATATAAAAATCCAAAATATGGCTCCTCGTTC	DpeA+C-3 (1,793 bp)	
pUSA-DpeA-FspAI-R	GAGAATATTGTAGGAGATCTTCTAGAAAGATGCGTGCCAGACAGCCTCACGGGGAC		
PamyB-DpeB-TamyB-F	AAGCTTGCATGCCTGCAGGTCGACTCTAGACCATCATGGTGTTTTGATCATTTT	PamyB-DpeB-TamyB (2,513 bp)	pAdeA(DpeB)
PamyB-DpeB-TamyB-R	TGCGCAGAATCCATATGACTAGTAGATCCTGTAAGATACATGAGCTTCGGTG		
PamyB-DpeD-TamyB-F	GGACTAGTCCATCATGGTGTTTTGATC	PamyB-DpeD-TamyB (1,853 bp)	pAdeA(DpeB+D)
PamyB-DpeD-TamyB-R	GGACTAGTCCGTAAGATACATGAGCTTCGGTG		

pUNA-DpeC-F	GCAAGCTCCGAATTCGAGCTCGGTACCCGGGATGAG TGACTCGTGCTGCCTGAAAG	DpeC-pUNA (826 bp)	pUNA(DpeC)
pUNA-DpeC-R	CTCTCCACCCTTCACGAGCTACTACAGATCCTACACG TTATCCGCATGTAACCACT		
pUNA-DpeH-F	GCAAGCTCCGAATTCGAGCTCGGTACCCGGGATGTT TCGCTCCATACCTTTGGTGGC	DpeH-pUNA (1,222 bp)	pUNA(DpeH)
pUNA-DpeH-R	CTCTCCACCCTTCACGAGCTACTACAGATCCTACTGG TAGATATAGCAGTATTTC		
PamyB-DpeI-TamyB-F	CTAGTCTAGACCATCATGGTGTTTTGATCAT	PamyB-DpeI-TamyB (1,957 bp)	pUNA(DpeH+I)
PamyB-DpeI-TamyB-R	CTAGTCTAGAGTAAGATACATGAGCTTCGGTG		
pTAex3-AN7912-F	ACAGCAAGCTCCGAATTCGAGCTCGGTACATGTTGG CCTTCAACCCGCTTGTCAC	AN7912-pTAex3 (1,169 bp)	pTAex3(AN7912)
pTAex3-AN7912-R	CACCCTTCACGAGCTACTACAGATCCCCGGTTACGG CTGGGCGGTACTATCATC		
DpeHK59-R	CCGGGTCTCGACATCCAGGGTGCTCCACTCCTTGCGC ACGGCAAGTTTGTTAG	DpeH(M^{1}-K^{59}) (227 bp)	pTAex3(M1)
AN7912E59-F	GAGTGGAGCACCCTGGATGTCG	AN7912(E^{59}-P^{369}) (966 bp)	
AN7912L343-R	TAGCGTTAGCGTGCCGTCCAGGGCATAC	AN7912(M^{1}-L^{343}) (1,058 bp)	pTAex3(M2)
DpeHL346-F	GAGTATGCCCTGGACGGCACGCTAACGCTACTTGAC ATTCCGCCTTCGCGCAATGC	DpeH(L^{346}-Q^{386}) (176 bp)	
DpeHL345-R	TAGCGTCAGTGTTCCATCCAAG	DpeH(M^{1}-L^{345}) (1,055 bp)	pTAex3(M4)
AN7912L344-F	GAATTTGCCTTGGATGGAACACTGACGCTACTGAAC TGTAAGAACCTACC	AN7912(L^{344}-P^{369}) (141 bp)	
DpeHL345-R2	CACCCTTCACGAGCTACTACAGATCCCCGGCTATAG CGTCAGTGTTCCATCCAAG	DpeH(M^{1}-L^{345})-2 (1,088 bp)	pTAex3(M5)
AN7912L343-R2	CACCCTTCACGAGCTACTACAGATCCCCGGTTATAGC GTTAGCGTGCCGTCCAGGGCA	AN7912(M^{1}-L^{343})-2 (1,091 bp)	pTAex3(M6)
DpeHP369-R	TGGGATGCTCGAGGTGAAGTC	DpeH(L^{346}-P^{369}) (102 bp)	pTAex3(M7)
DpeHS375-F	ATGGAATATGACTTCACCTCGAGCATCCCAAGTCCC ACCAAGGAGAAATAC	DpeH(S^{375}-Q^{386}) (89 bp)	
pTAex3-DpeH-R2	CACCCTTCACGAGCTACTACAGATCCCCGGCTAGCA GTATTTCTCCTTGGTGGGACTC	M2-Δ(Y^{381}-Q^{384}) (1,202 bp)	pTAex3(M8)
pTAex3-DpeH-R3	CACCCTTCACGAGCTACTACAGATCCCCGGCTAACTC ATTCCAGCTCTCACTGGGATG	M2-Δ(P^{374}-Q^{384}) (1,181 bp)	pTAex3(M9)
pTAex3-DpeH-R4	CACCCTTCACGAGCTACTACAGATCCCCGGCTATGG GATGCTCGAGGTGAAGTC	M2-Δ(V^{368}-Q^{384}) (1,163 bp)	pTAex3(M10)
28a-DpeE-F	AGCAGCGGCCTGGTGCCGCGCGGCAGCCATATGTCG CCATCATTCCATCCAG	28a-DpeE (939 bp)	pET28a(DpeE)
28a-DpeE-R	ATCTCAGTGGTGGTGGTGGTGGTGCTCGAGAATGTTT CTTTGCGTCCAAAGAAC		
28a-DpeI-F	AGCAGCGGCCTGGTGCCGCGCGGCAGCCATATGTCG AACCAAGGTGCATACCTC	28a-DpeI (1,098 bp)	pET28a(DpeI)
28a-DpeI-R	ATCTCAGTGGTGGTGGTGGTGGTGCTCGAGGACCTC GACAACCAACTTGG		

Table S9. ^{1}H NMR and ^{13}C NMR data

Table S9.1. Compounds **7** and **8** (in methanol-d_4)

No.	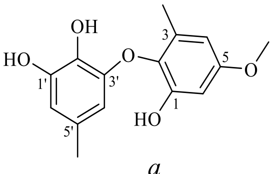 **7** [a]		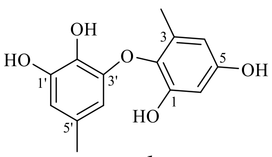 **8** [b]	
	δ_C, type	δ_H (J in Hz)	δ_C, type	δ_H (J in Hz)
1	151.9, C		151.8, C	
2	136.1, C		135.3, C	
3	134.1, C		134.0, C	
4	108.0, CH	6.31, d (2.9)	109.5, CH	6.19, d (2.8)
5	158.7, C		156.0, C	
6	101.4, CH	6.36, d (2.9)	102.6, CH	6.26, d (2.8)
5-OCH_3	56.0, CH_3	3.74, s		
3-CH_3	16.6, CH_3	2.05, s	16.4, CH_3	2.01, s
1'	147.2, C		147.2, C	
2'	132.7, C		132.7, C	
3'	148.1, C		148.2, C	
4'	106.8, CH	5.74, d (1.2)	106.8, CH	5.77, d (1.9)
5'	129.8, C		129.8, C	
6'	111.0, CH	6.28, d (1.2)	111.0, CH	6.28, d (2.0)
5'-CH_3	21.3, CH_3	2.03, s	21.4, CH_3	2.05, s

[a] ^{1}H and ^{13}C NMR spectra were recorded at 600 and 150 MHz, respectively.

[b] ^{1}H and ^{13}C NMR spectra were recorded at 500 and 125 MHz, respectively.

Table S9.2. Compounds **9**, **9a** and **9b** (in DMSO-d_6)

No.	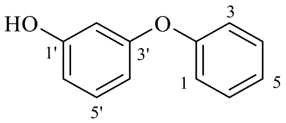 **9**		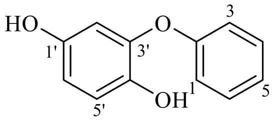 **9a**		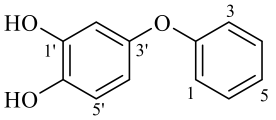 **9b**	
	δ_C, type	δ_H (J in Hz)	δ_C, type	δ_H (J in Hz)	δ_C, type	δ_H (J in Hz)
1	119.0 CH	7.01, m	116.5, CH	6.85, d (8.7)	117.2, CH	6.89, d (8.1)
2	156.5, C		157.8, C		158.3, C	
3	119.0, CH	7.01, m	116.5, CH	6.85, d (8.7)	117.2, CH	6.89, d (8.1)
4	130.0, CH	7.38, tt (7.5, 2.2)	129.6, CH	7.30, tt (7.5, 2.2)	129.8, CH	7.31, tt (7.7, 2.2)
5	123.5, CH	7.14, m	122.0, CH	7.01, t (7.4)	122.2, CH	7.03, t (7.4)
6	130.0, CH	7.38, tt (7.5, 2.2)	129.6, CH	7.30, tt (7.5, 2.2)	129.8, CH	7.31, tt (7.7, 2.2)
1'	158.8, C-OH	9.60, s	150.3, C		146.3, C	
2'	105.4, CH	6.36, t (2.3)	108.5, CH	6.33, d (2.8)	107.7, CH	6.42, d (2.6)
3'	157.9, C		142.8, C		148.2, C	
4'	109.0, CH	6.41, ddd (8.1, 2.3, 0.8)	141.5, C		109.9, CH	6.30, dd (8.4, 2.6)
5'	130.5, CH	7.14, m	117.6, CH	6.76, d (8.7)	116.0, CH	6.71, d (8.5)
6'	110.5, CH	6.53, ddd (8.2, 2.3, 0.8)	111.6, CH	6.44, dd (8.7, 2.8)	142.0, C	

^{1}H and ^{13}C NMR spectra were recorded at 500 and 125 MHz, respectively.

Table S9.3. Compounds **10** and **10a** (in methanol-d_4)

No.	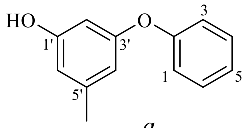 **10** [a]		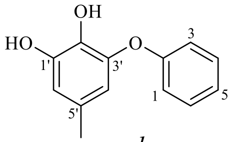 **10a** [b]	
	δ_C, type	δ_H (J in Hz)	δ_C, type	δ_H (J in Hz)
1	120.1, CH	6.95, d (8.0)	118.4, CH	6.91, d (8.7)
2	158.8, C		159.7, C	
3	120.1, CH	6.95, d (8.0)	118.4, CH	6.91, d (8.5)
4	130.9, CH	7.31, t (7.3)	130.6, CH	7.28, m
5	124.4, CH	7.07, t (7.3)	123.4, CH	7.01, t (7.4)
6	130.9, CH	7.31, t (7.3)	130.6, CH	7.28, m
1'	159.8, C		148.0, C	
2'	104.2, CH	6.19, s	136.2, C	
3'	159.9, C		145.4, C	
4'	111.7, CH	6.26, s	113.4, CH	6.20, d (1.2)
5'	141.9, C		130.2, C	
6'	112.2, CH	6.36, s	113.3, CH	6.46, d (1.4)
5'-CH_3	21.7, CH_3	2.21, s	21.1, CH_3	2.14, s

[a] ^{1}H and ^{13}C NMR spectra were recorded at 600 and 150 MHz, respectively.

[b] ^{1}H and ^{13}C NMR spectra were recorded at 500 and 125 MHz, respectively.

Table S9.4. Compounds **11**, **11a** and **11c** (in methanol-d_4)

No.	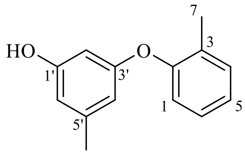 **11**		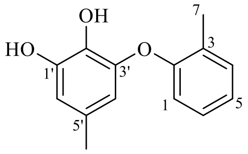 **11a**		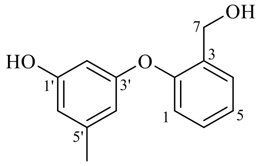 **11c**	
	δ_C, type	δ_H (J in Hz)	δ_C, type	δ_H (J in Hz)	δ_C, type	δ_H (J in Hz)
1	121.4, CH	6.87, d (8.0)	119.2, CH	6.76, d (8.1)	120.5, CH	6.87, d (8.0)
2	155.8, C		156.9, C		155.2, C	
3	131.3, C		130.2, C		134.2, C	
4	132.5, CH	7.24, d (7.5)	132.3, CH	7.22, d (7.0)	129.7, CH	7.52, d (7.4)
5	125.4, CH	7.05, t (7.4)	124.4, CH	6.99, t (7.4)	125.1, CH	7.15, t (7.4)
6	128.4, CH	7.16, t (7.7)	128.1, CH	7.11, t (7.7)	129.7, CH	7.25, t (7.3)
7	16.4, CH_3	2.17, s	16.4, CH_3	2.27, s	60.1, CH_2	4.63, s
1'	159.8, C		147.7, C		159.9, C	
2'	102.5, CH	6.06, s	135.1, C		103.3, CH	6.14, s
3'	160.6, C		146.7, C		160.3, C	
4'	110.0, CH	6.15, s	111.3, CH	5.98, d (1.3)	110.7, CH	6.22, s
5'	141.8, C		130.1, C		141.9, C	
6'	111.3, CH	6.30, s	112.4, CH	6.40, d (1.4)	111.9, CH	6.34, s
5'-CH_3	21.7, CH_3	2.19, s	21.1, CH_3	2.10, s	21.7, CH_3	2.21, s

^1H and ^{13}C NMR spectra were recorded at 600 and 150 MHz, respectively.

Table S9.5. Compounds **12** and **12a** (in methanol-d_4)

No.	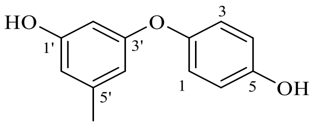 **12**		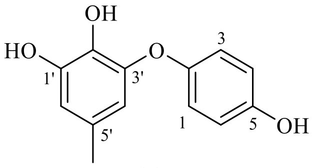 **12a**	
	δ_C, type	δ_H (J in Hz)	δ_C, type	δ_H (J in Hz)
1	122.3, CH	6.84, d (8.5)	120.7, CH	6.81, d (8.6)
2	150.6, C		151.8, C	
3	122.3, CH	6.84, d (8.5)	120.7, CH	6.81, d (8.6)
4	117.2, CH	6.77, d (8.6)	117.0, CH	6.73, d (8.6)
5	155.0, C		154.3, C	
6	117.2, CH	6.77, d (8.6)	117.0, CH	6.73, d (8.6)
1'	159.6, C		147.6, C	
2'	102.8, CH	6.11, s	135.2, C	
3'	161.5, C		147.3, C	
4'	110.3, CH	6.19, s	111.6, CH	6.08, s
5'	141.6, C		130.0, C	
6'	111.2, CH	6.28, s	112.3, CH	6.38, s
5'-CH_3	21.7, CH_3	2.19, s	21.1, CH_3	2.10, s

^{1}H and ^{13}C NMR spectra were recorded at 600 and 150 MHz, respectively.

Table S9.6. Compounds **13** and **13a** (in methanol-d_4)

No.	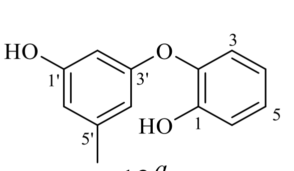 **13** [a]		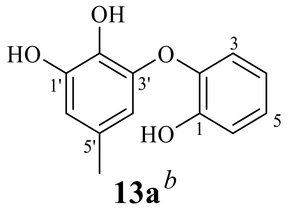 **13a** [b]	
	δ_C, type	δ_H (J in Hz)	δ_C, type	δ_H (J in Hz)
1	150.4, C		149.4, C	
2	145.1, C		146.4, C	
3	122.3, CH	6.88, dd (8.0, 1.6)	120.1, CH	6.80, dd (8.3, 1.7)
4	121.2, CH	6.80, ddd (8.0, 7.3, 1.6)	121.1, CH	6.76, ddd (8.2, 7.0, 1.7)
5	126.1, CH	6.99, ddd (8.0, 7.3, 1.6)	125.2, CH	6.94, ddd (8.4, 6.9, 1.6)
6	118.2, CH	6.92, dd (8.0, 1.6)	117.9, CH	6.91, dd (8.0, 1.7)
1'	159.6, C		147.7, C	
2'	102.7, CH	6.16, t (2.1)	135.2, C	
3'	160.6, C		146.6, C	
4'	110.3, CH	6.24, m	111.6, CH	6.14, d (1.3)
5'	141.6, C		130.1, C	
6'	111.4, CH	6.31, m	112.7, CH	6.41, d (1.4)
5'-CH_3	21.7, CH_3	2.20, s	21.2, CH_3	2.12, s

[a] ^{1}H and ^{13}C NMR spectra were recorded at 500 and 125 MHz, respectively.

[b] ^{1}H and ^{13}C NMR spectra were recorded at 600 and 150 MHz, respectively.

Table S9.7. Compounds **14** and **14a** (in DMSO-d_6)

No.	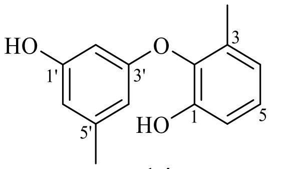 **14**		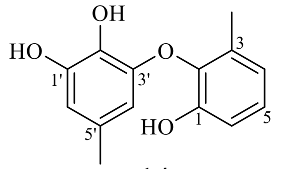 **14a**	
	δ_C, type	δ_H (J in Hz)	δ_C, type	δ_H (J in Hz)
1	150.2, C		150.3, C	
2	139.9, C		140.8 C	
3	132.0, C		132.0, C	
4	121.1, CH	6.70, d (7.4)	121.1, CH	6.68, d (7.5)
5	125.4, CH	6.94, t (7.8)	125.1, CH	6.92, t (7.8)
6	114.7, CH	6.78, d (8.0)	114.7, CH	6.76, d (7.6)
3-CH_3	15.9, CH_3	2.02, s	15.8, CH_3	2.03, s
1'	158.3, C		146.5, C	
2'	98.9, CH	5.91, t (1.9)	132.0, C	
3'	158.8, C		146.3, C	
4'	106.3, CH	6.05, br s	105.2, CH	5.61, s
5'	139.5, C		126.6, C	
6'	109.2, CH	6.18, br s	110.0, CH	6.21, s
5'-CH_3	21.3, CH_3	2.13, s	20.8, CH_3	1.96, s

^{1}H and ^{13}C NMR spectra were recorded at 600 and 150 MHz, respectively.

Table S9.8. Compounds **15** and **15a** (in methanol-d_4)

No.	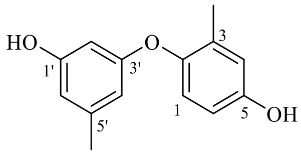 **15**		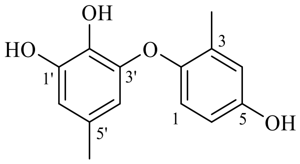 **15a**	
	δ_C, type	δ_H (J in Hz)	δ_C, type	δ_H (J in Hz)
1	123.3, CH	6.76, d (8.6)	122.2, CH	6.72, d (8.6)
2	147.8, C		148.7, C	
3	132.9, C		132.3, C	
4	118.7, CH	6.68, br s	118.7, CH	6.67, d (2.8)
5	155.3, C		154.9, C	
6	114.7, CH	6.61, br d	114.5, CH	6.58, dd (8.6, 2.9)
3-CH_3	16.5, CH_3	2.07, s	16.5, CH_3	2.13, s
1'	159.6, C		147.3, C	
2'	101.4, CH	6.01, s	133.9, C	
3'	161.7, C		148.2, C	
4'	109.0, CH	6.11, s	109.1, CH	5.83, br s
5'	141.6, C		129.8, C	
6'	110.6, CH	6.24, s	111.4, CH	6.31, br s
5'-CH_3	21.8, CH_3	2.18, s	21.2, CH_3	2.06, s

^1H and ^{13}C NMR spectra were recorded at 600 and 150 MHz, respectively.

Table S9.9. Compounds **16** and **16a** (in methanol-d_4)

No.	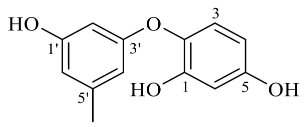 **16**		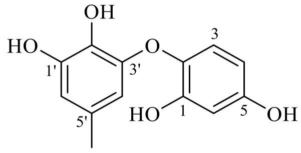 **16a**	
	δ_C, type	δ_H (J in Hz)	δ_C, type	δ_H (J in Hz)
1	151.3, C		150.7, C	
2	137.4, C		138.5, C	
3	123.7, CH	6.73, d (8.6)	122.5, CH	6.73, d (8.7)
4	107.7, CH	6.26, dd (8.6, 2.2)	107.6, CH	6.24, dd (8.7, 2.7)
5	156.2, C		155.8, C	
6	105.2, CH	6.41, d (2.3)	105.1, CH	6.40, d (2.7)
1'	159.5, C		147.4, C	
2'	101.8, CH	6.11, s	134.1, C	
3'	161.7, C		148.2, C	
4'	109.4, CH	6.19, s	109.9, CH	6.03, s
5'	141.3, C		129.9, C	
6'	110.7, CH	6.25, s	111.8, CH	6.34, s
5'-CH_3	21.8, CH_3	2.19, s	21.2, CH_3	2.09, s

^{1}H and ^{13}C NMR spectra were recorded at 600 and 150 MHz, respectively.

Table S9.10. Compounds **17** and **17a** (in methanol-d_4)

No.	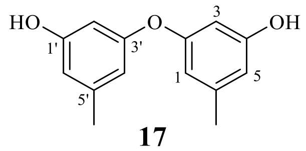 **17**		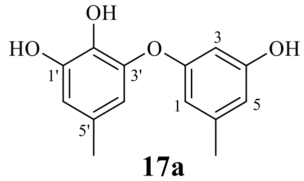 **17a**	
	δ_C, type	δ_H (J in Hz)	δ_C, type	δ_H (J in Hz)
1	111.9, CH	6.27, br s	110.4, CH	6.25, br s
2	159.8, C		160.7, C	
3	104.4, CH	6.20, t (2.1)	102.8, CH	6.16, t (2.0)
4	159.7, C		159.5, C	
5	112.1, CH	6.36, br s	111.3, CH	6.29, br s
6	141.8, C		141.5, C	
6-CH_3	21.7, CH_3	2.22, s	21.8, CH_3	2.21, s
1'	159.7, C		147.8, C	
2'	104.4, CH	6.20, t (2.1)	136.1, C	
3'	159.8, C		145.4, C	
4'	111.9, CH	6.27, br s	113.6, CH	6.21, d (1.3)
5'	141.8, C		130.1, C	
6'	112.1, CH	6.36, br s	113.2, CH	6.45, d, (1.4)
5'-CH_3	21.7, CH_3	2.22, s	21.1, CH_3	2.15, s

^{1}H and ^{13}C NMR spectra were recorded at 600 and 150 MHz, respectively.

3 SI Figures

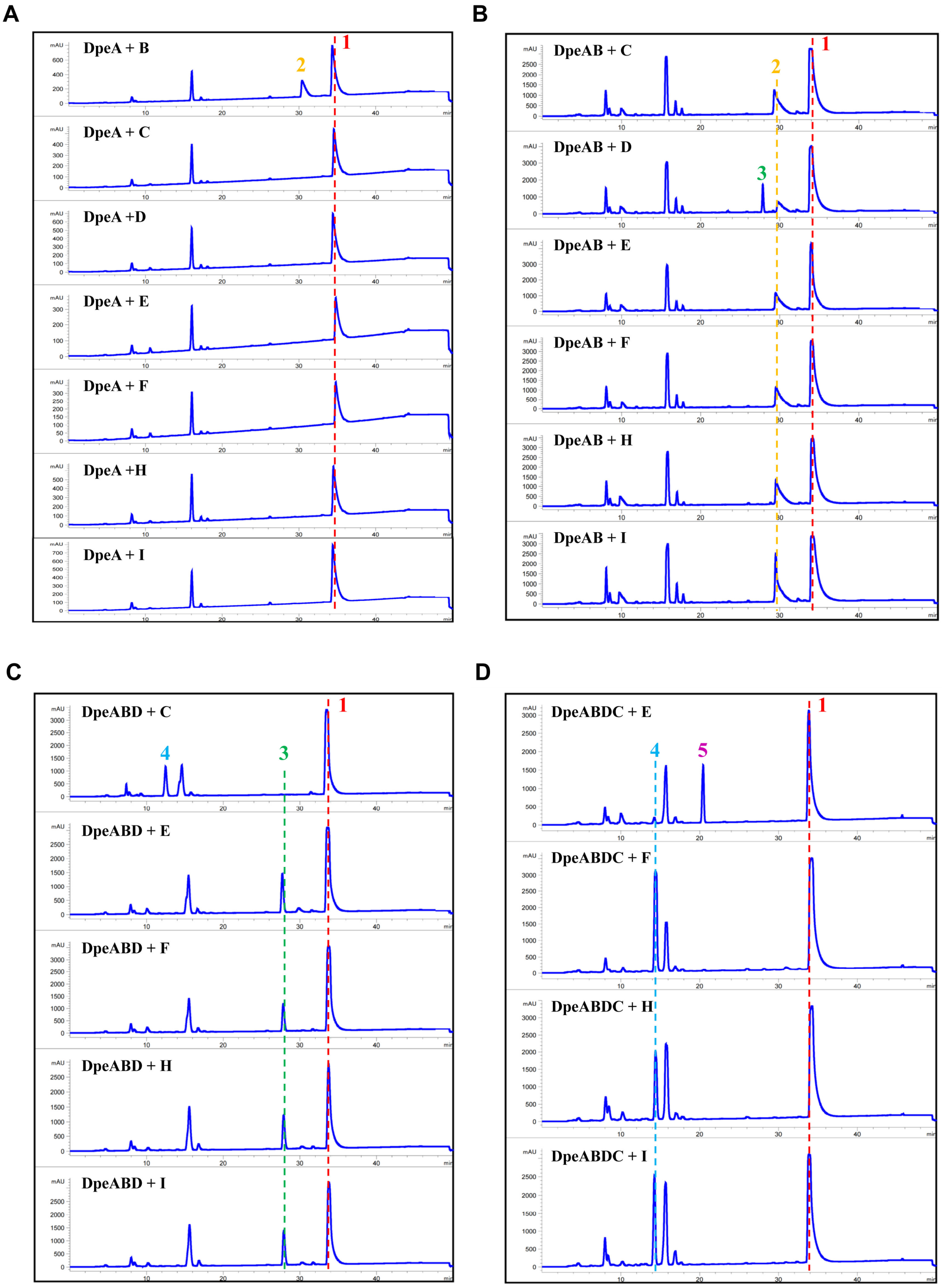

Figure S1. Stepwise reconstitution of the *dpe* cluster by heterologous expression in *S. cerevisiae* BJ5464-NpgA (λ = 210 nm).

	***S. cerevisiae* BJ5464-NpgA transformed by**
**A**	YEpPreu6 + YEpTRP(DpeB/C/D/E/F/H/I)
**B**	YEpPreu6 + YEpLEU(DpeB) + YEpTRP(DpeC/D/E/F/H/I)
**C**	YEpPreu6 + YEpLEU(DpeB+D) + YEpTRP(DpeC/E/F/H/I)
**D**	YEpPreu6 + YEpLEU(DpeB+D+C) + YEpTRP(DpeE/F/H/I)

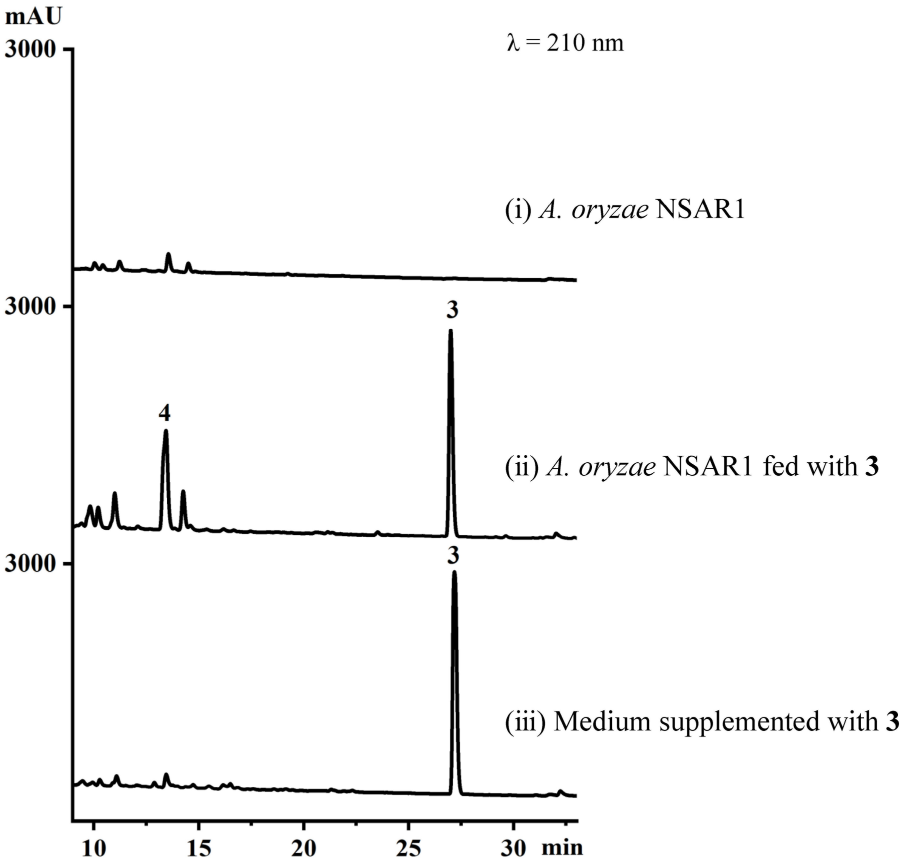

Figure S2. Transformation of compound **3** to **4** by *A. oryzae* NSAR1.

A. oryzae NSAR1 was grown in 500 mL Erlenmeyer flasks containing 150 mL of DPY medium at 30 ºC with shaking at 160 rpm for 4 days. Then, an equivalent volume of CD medium and 10 mg of compound **3** (dissolved in DMSO) were added to the fermentation broth, and the resulting culture was cultivated with shaking at 30 ºC for an additional 2 days. Controls included un-inoculated medium with compound **3** (iii) and *A. oryzae* NSAR1 culture with no feeding (i).

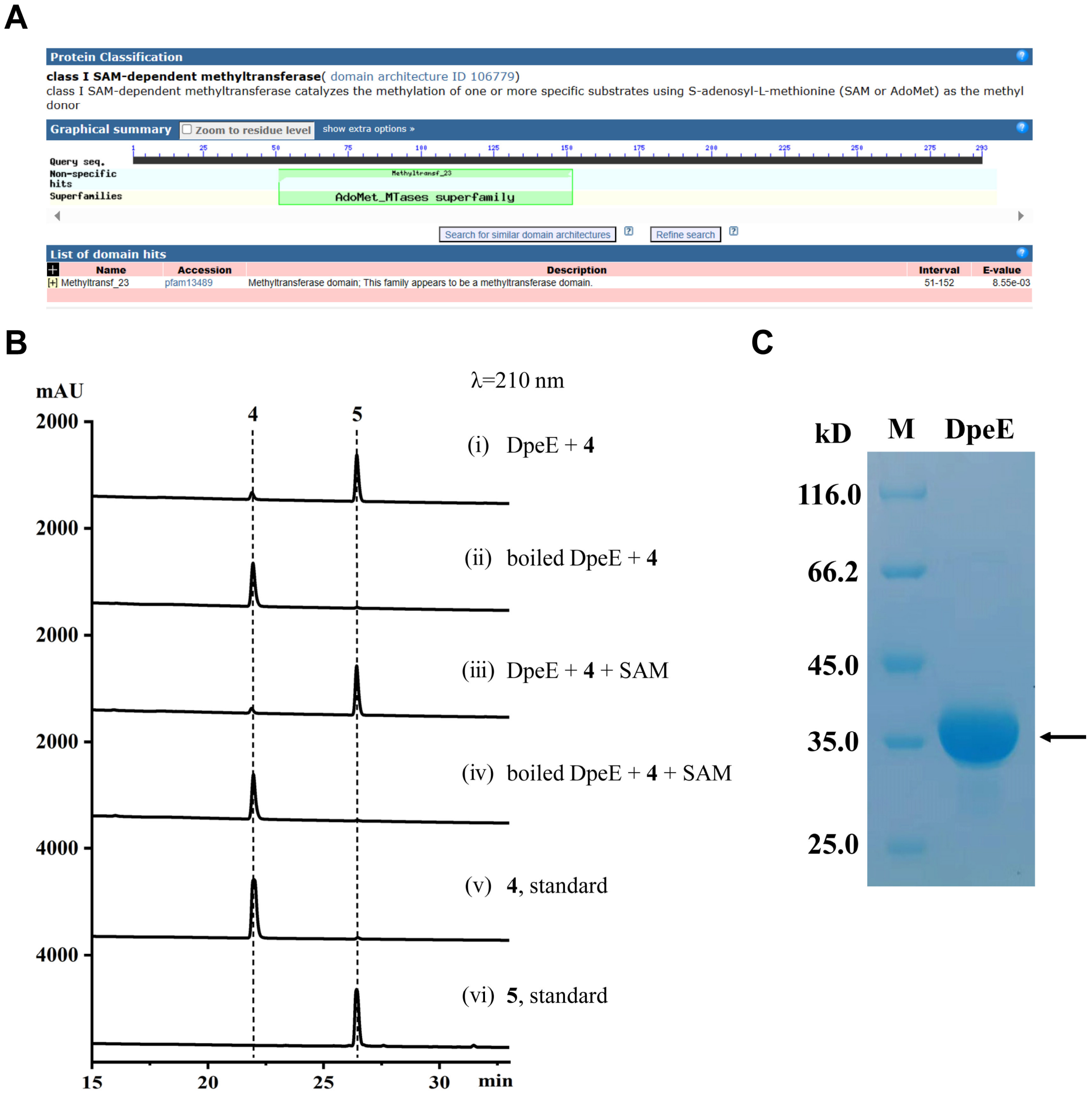

Figure S3. Confirmation of the function of DpeE.

A. BLASTP search predicts DpeE as a SAM-dependent methyltransferase. B. *In vitro* assays of recombinant DpeE with compound **4** (2 h incubation). C. DpeE (~34.6 KDa) purified from *E. coli* BL21-CodonPlus (DE3)-RIPL.

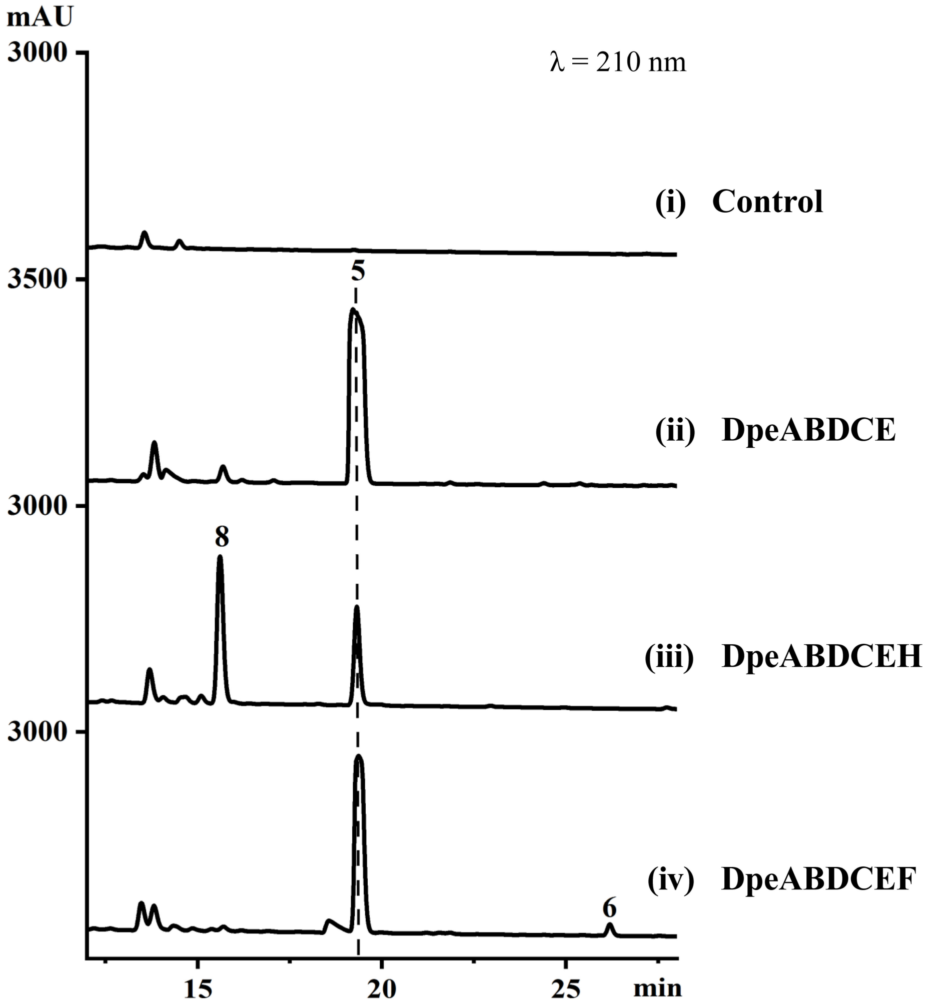

Figure S4. *A. oryzae* NSAR1 preferentially uses DpeH to transform **5** to **8**.
Control, *A. oryzae* NSAR1 transformed by the corresponding empty vectors.

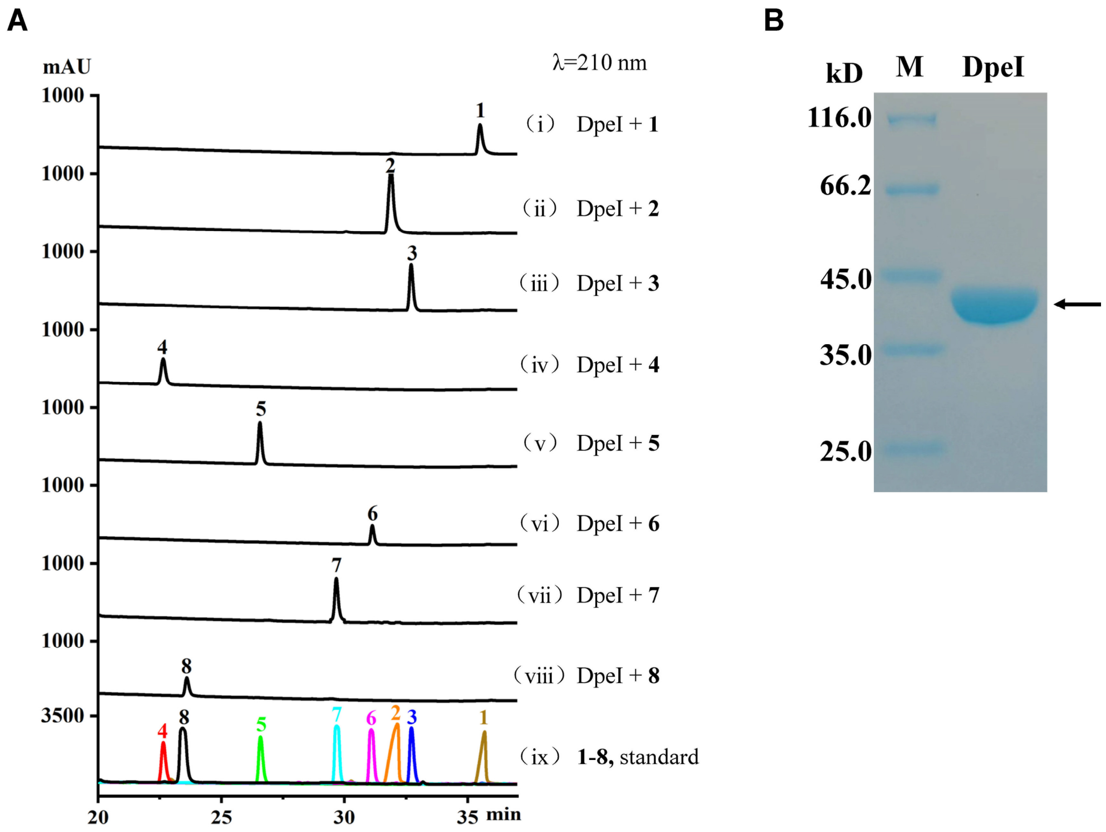

Figure S5. Assays with purified, recombinant DpeI.

A. *In vitro* assays of recombinant DpeI with compounds **1-8** (6 h incubation). B. DpeI (~38.6 KDa) purified from *E. coli* BL21-CodonPlus (DE3)-RIPL.

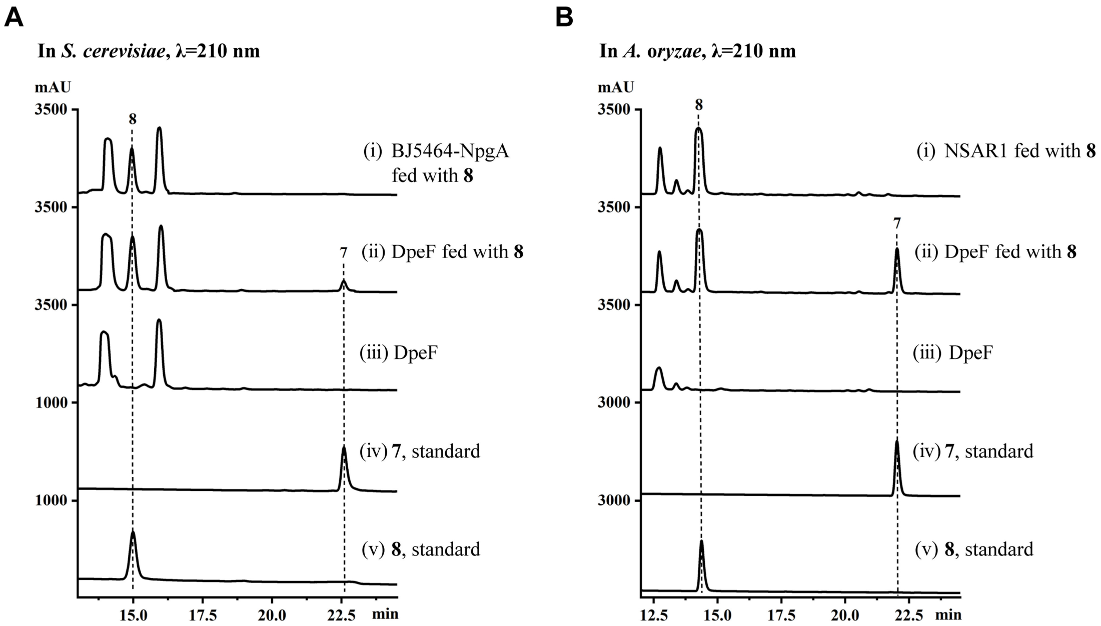

Figure S6. Biotransformation of compound **8** to **7** with DpeF-producing *S. cerevisiae* or *A. oryzae* strains.

Controls included wild-type strains fed with compound **8** (i) and DpeF transformants with no feeding (iii).

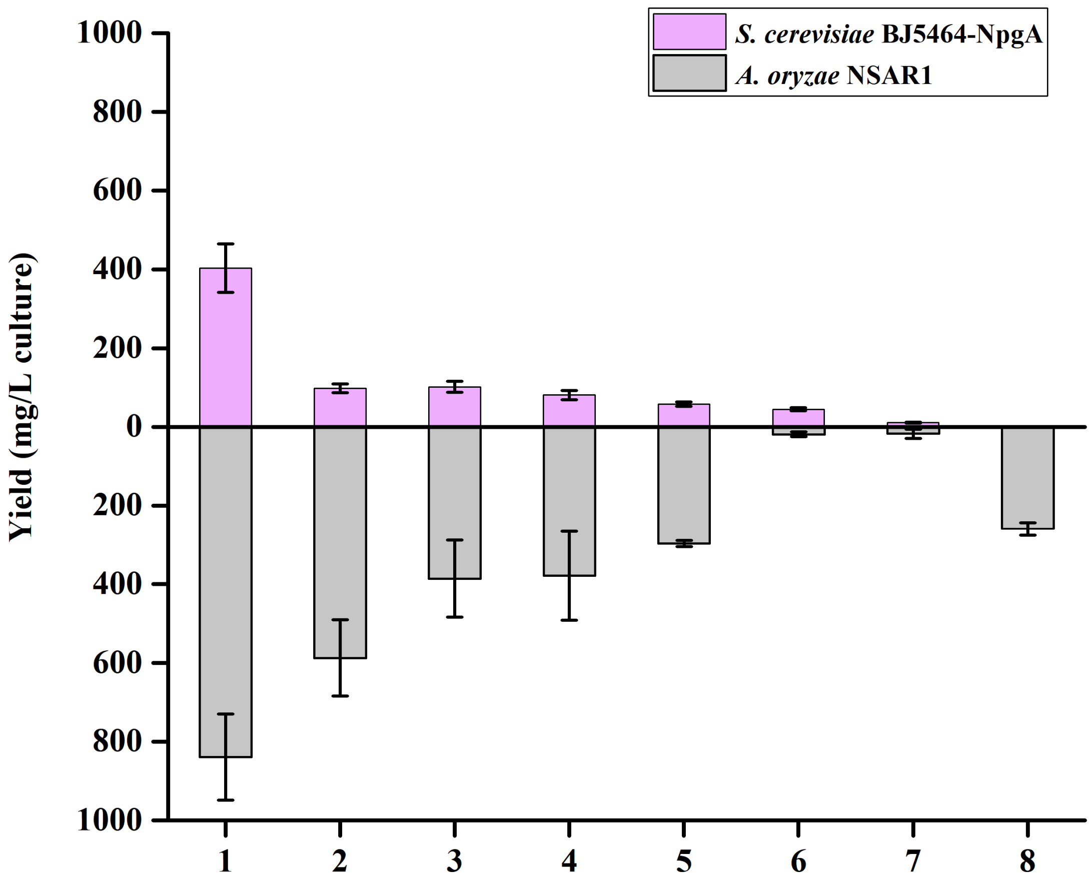

Figure S7. Quantitative analysis of the production of compounds **1-8** in the *S. cerevisiae* or *A. oryzae* systems (mean ± SD, $n = 10$ [two independent experiments of five transformants each]).

S. cerevisiae or *A. oryzae* transformants used for compound quantitation

	***S. cerevisiae* BJ5464-NpgA transformed by**	***A. oryzae* NSAR1 transformed by**
**1**	YEpPreu6	pUSA(DpeA)
**2**	YEpPreu6 + YEpTRP(DpeB)	pUSA(DpeA) + pAdeA(DpeB)
**3**	YEpPreu6 + YEpLEU(DpeB) + YEpTRP(DpeD)	pUSA(DpeA) + pAdeA(DpeB) + pTAex3(DpeD)
**4**	YEpPreu6 + YEpLEU(DpeB+D) + YEpTRP(DpeC)	pUSA(DpeA+C) + pAdeA(DpeB+D)
**5**	YEpPreu6 + YEpLEU(DpeB+D+C) + YEpTRP(DpeE)	pUSA(DpeA+C) + pAdeA(DpeB+D) + pTAex3(DpeE)
**6**	YEpPreu6 + YEpLEU(DpeB+D+C+E) + YEpTRP(DpeF)	pUSA(DpeA+C) + pAdeA(DpeB+D) + pTAex3(DpeE+F) + pUNA(DpeH+I)
**7**	YEpPreu6 + YEpLEU(DpeB+D+C+E+F) + YEpTRP(DpeH)	pUSA(DpeA+C) + pAdeA(DpeB+D) + pTAex3(DpeE+F) + pUNA(DpeH+I)
**8**	——	pUSA(DpeA+C) + pAdeA(DpeB+D) + pTAex3(DpeE) + pUNA(DpeH)

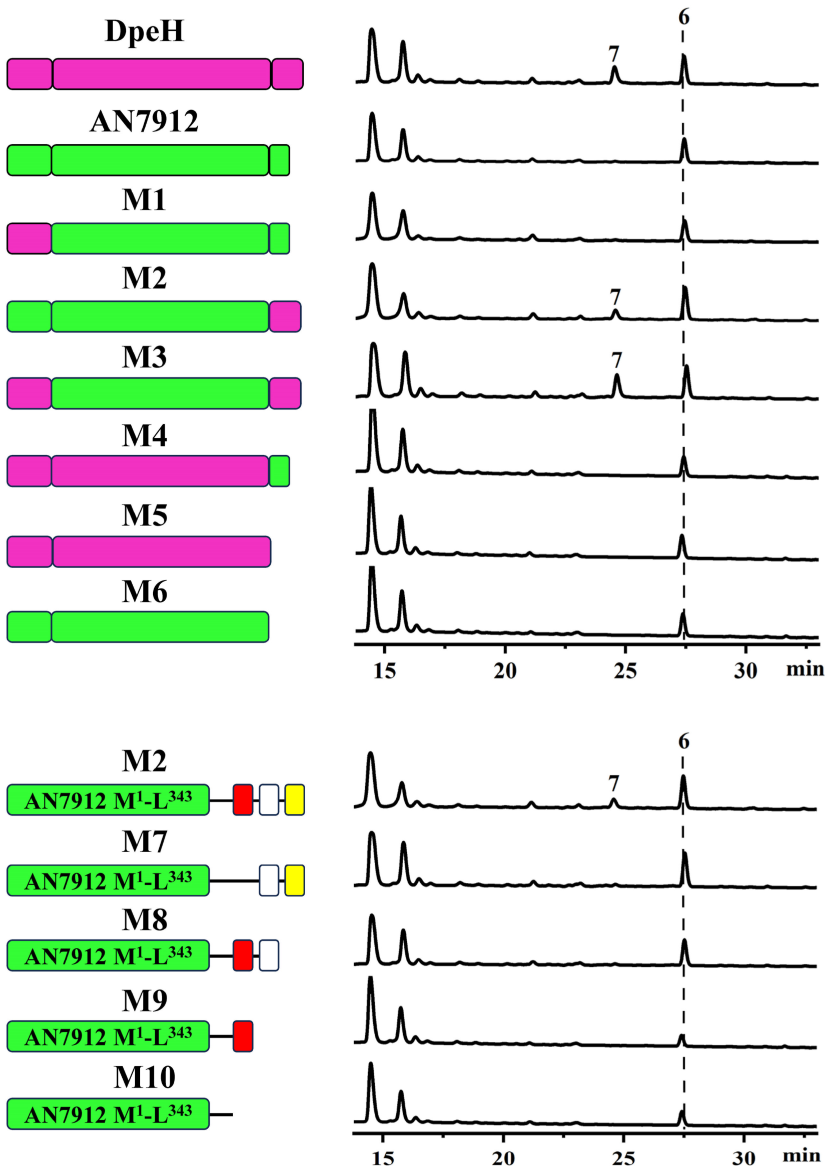

Figure S8. Product profiles (210 nm) of the *A. oryzae* NSAR1 strains expressing the indicated enzymes and challenged with substrate **6**.

In mutant enzymes M1-M10, AN7912-derived regions are shown in *green*. In mutants M1-M5, segments from DpeH are in *magenta*. In mutants M7-M10 (derived from M2), *missing boxes* indicate deletions; *red boxes* show the α-helix, *white boxes* represent the linker, and *yellow boxes* indicate the β-sheet of DpeH.

**DpeH**	**1**	MFRS I PLVALVALFS IVFPGTQA–ADSVLMKQIVQNYT S NTKANLPPNQACQANKLAVRK	**59**
**AN7912**	**1**	MLAFNPLV—TALAALIFLFCQANANPPLMQRLVHEYQWKTKQGLPRQGACT PHNLAVRR	**58**
**DpeH**	**60**	EWGDLSALQRLDYIRAVKCLASRPSKIDPAKAPGARSRYDDFEATHIIQTPLVHGTGLFFAWH	**122**
**AN7912**	**59**	EWS TLDVETRLEYIEAVKCLARLPS I IDPELAPGARS RFDDFQATHIRHTRTIHATGSFFAWH	**121**
**DpeH**	**123**	RHL LYLYETALREECGYNGFQPYWDWAKYADKPQRANPLYDGSLTSMSGNGRFIPNRNG	**181**
**AN7912**	**122**	RHFVYLYEKALREECGYTGYQPYWEWSHWANLPI TANPLYDGSNASLSGNGVYIPNRNG	**180**
**DpeH**	**182**	TYQPLPLP I PNP–PALYCPPGTGGGYVYEGPFVNWELHLGPGVDLSHTKNAQYVKPNPRPD	**241**
**AN7912**	**181**	TLQLFP I PNPSPDTA IYT PPGTGGGY IYDGPLVDWELHLGP — VLYSYDNGQY I PPNPRPD	**239**
**DpeH**	**242**	GLGYNPRRMIRDFNNTLLQDFNTY P AITDMLQTKKTIAEYQTYFFVNPHTQAHLFISGYDND	**303**
**AN7912**	**240**	GLGYNPRP L IRDFNNTLLQQGASWDI ILNMLVNVTDMHEFHPLFFQGPHLAGHIFISGVDND	**301**
**DpeH**	**304**	LWTSPGDPLFWFHHAQVDRLY S IWQSLDFPNREFALDGTLTLLD I P — PSRNATLDD IMEYD	**363**
**AN7912**	**302**	I FTSPGDPLFWFHHAQVDR IWTIWQALDLETREYALDGTLTLLNCKNLPFRR I LAGELTVCD	**363**
**DpeH**	**364**	FT SS I PVRAGMSPTKEKYCYIYQ	**386**
**AN7912**	**364**	DSTAQP	**369**

Figure S9. Amino acid alignment of DpeH and AN7912.

Amino acids highlighted in yellow or green backgrounds indicate the α-helix or β-sheet structures, respectively, predicted by AlphaFold2 (https://www.alphafold.ebi.ac.uk/).

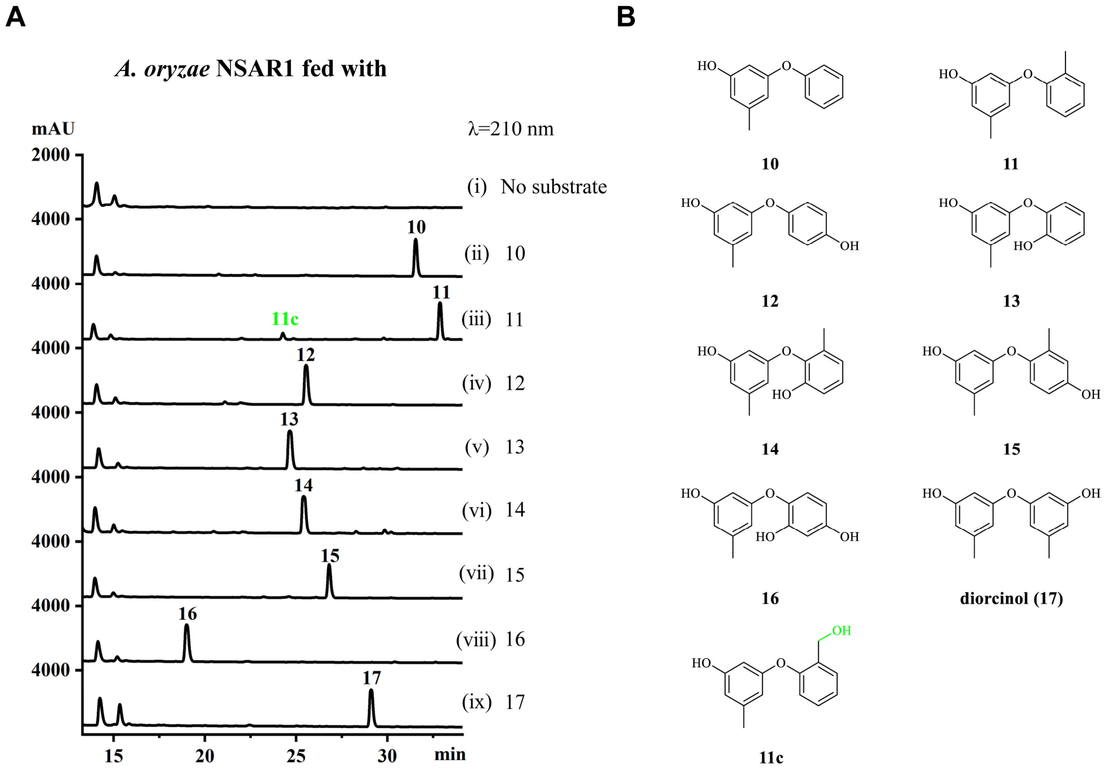

Figure S10. Product profiles (reversed-phase HPLC traces, 210 nm) of the *A. oryzae* NSAR1 strain fed with substrates **10-17**.

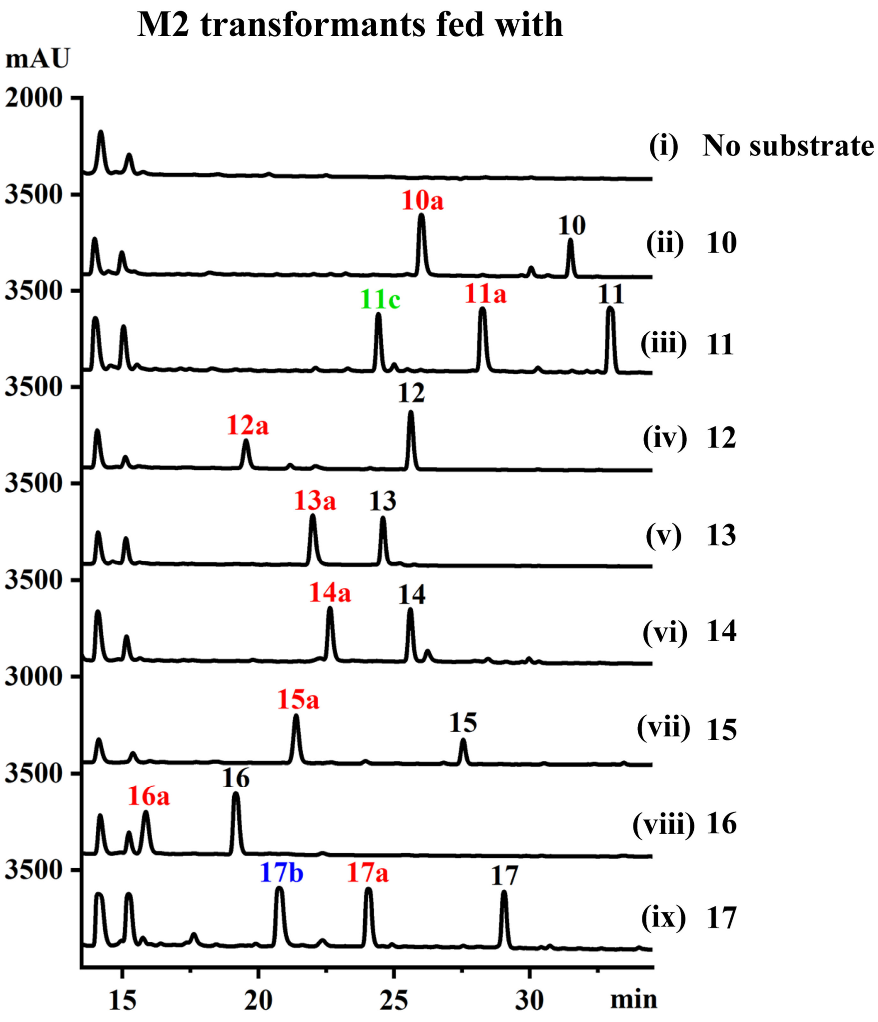

Figure S11. Product profiles (210 nm) of the *A. oryzae* NSAR1 strains expressing M2, and fed with substrates **10-17**, respectively.

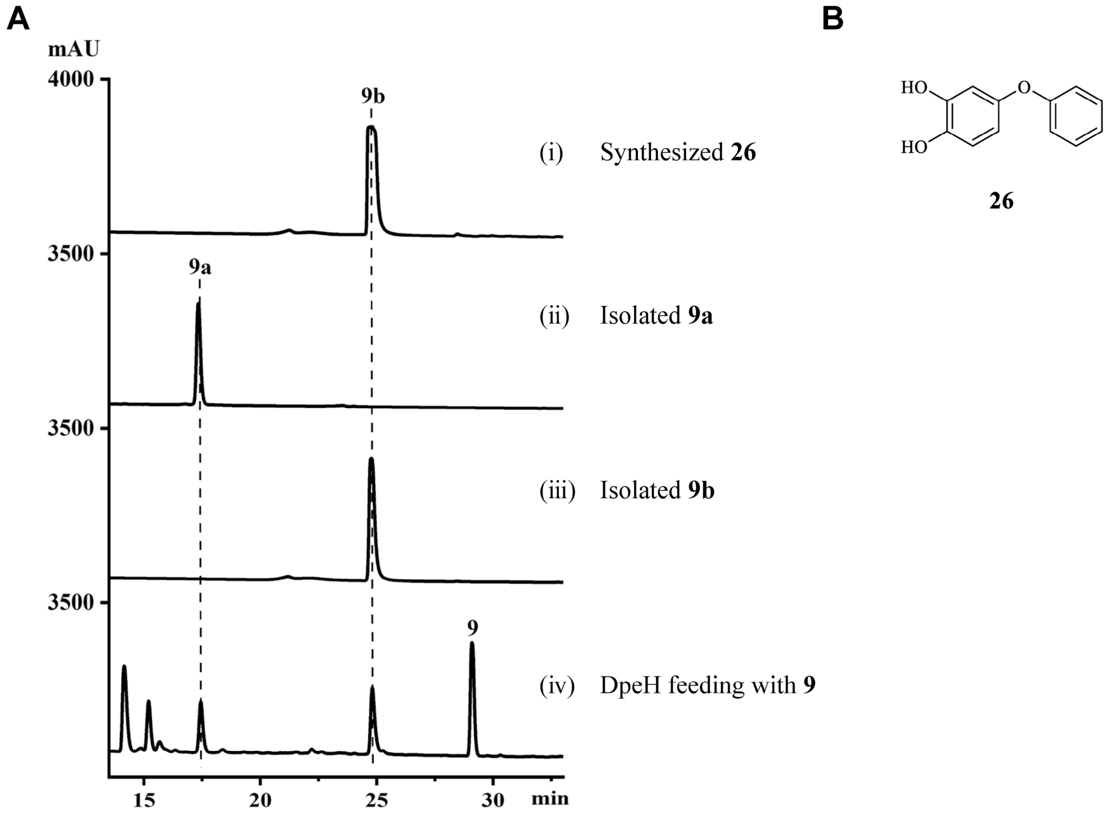

Figure S12. Product profiles (210 nm) of **9a**, **9b** and synthesized **26**, respectively.

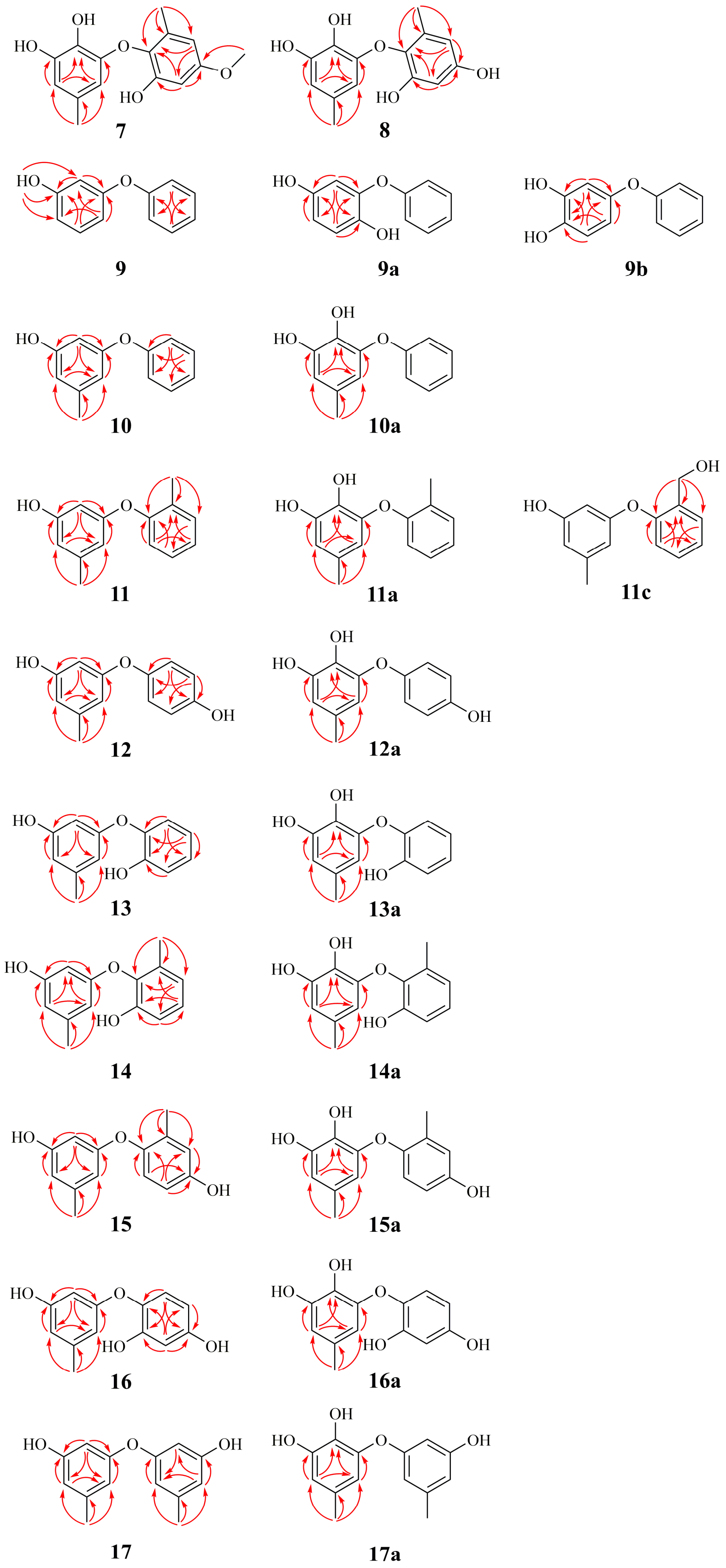

Figure S13. Chemical structures and key HMBC (→) correlations of the DPEs.

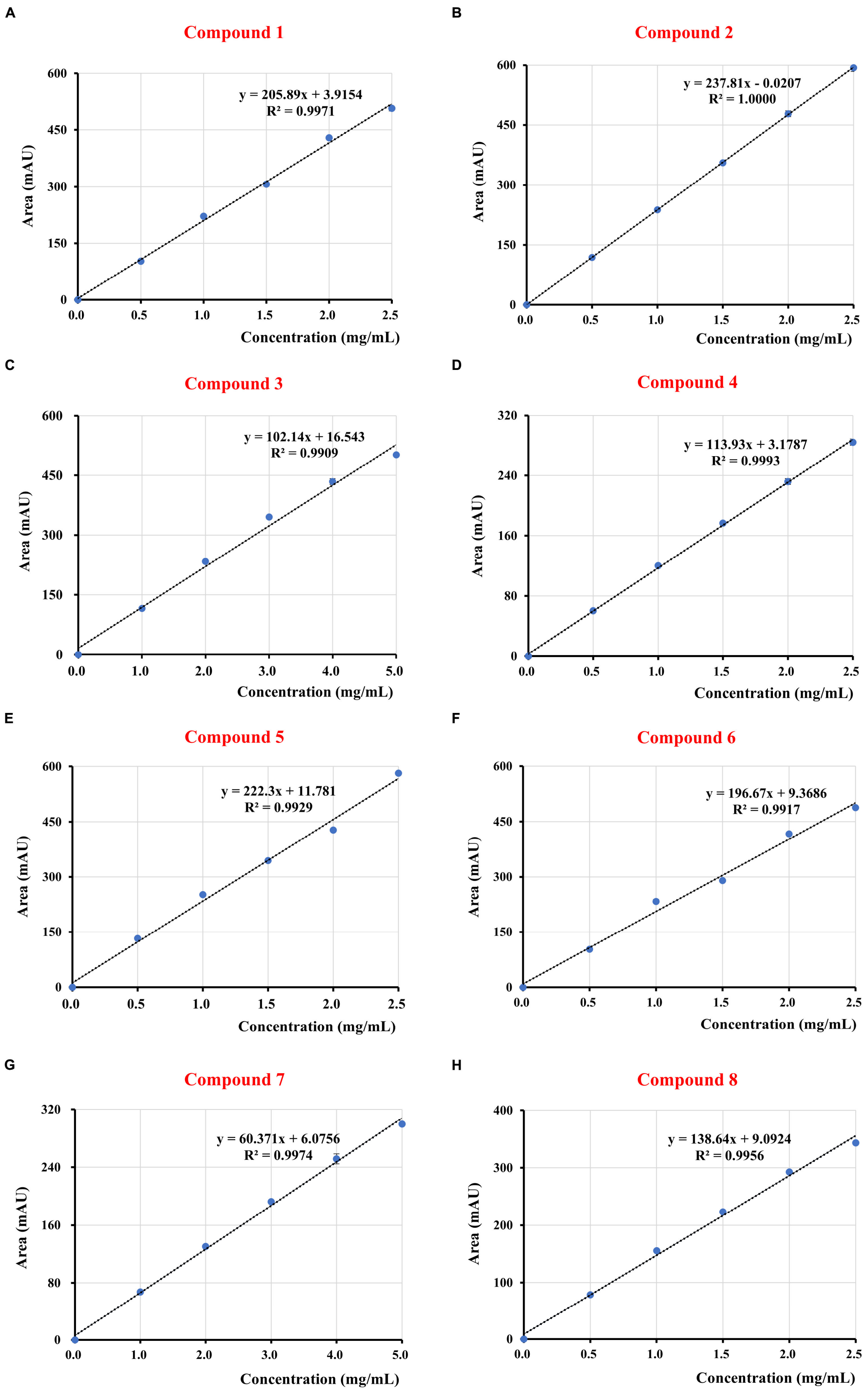

Figure S14. Standard curves of Compounds **1-8** (**A-H**).

Compounds **1-8** were accurately weighed and diluted to yield a series of stock solutions. Every stock solution was analyzed by UHPLC to record the peak area (DAD, 210 nm), with the injection volume set to 2 μL. Three technical replicates for each stock solution were analyzed.

Figure S15. UV-VIS spectra of the DEPs and DPEs.

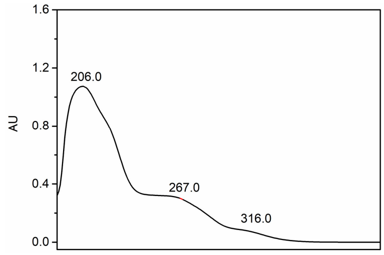

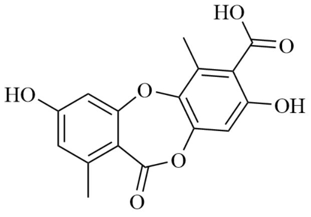

2

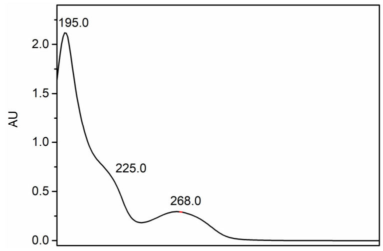

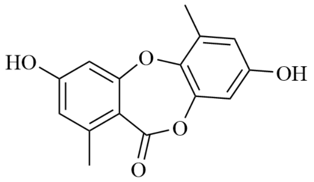

3

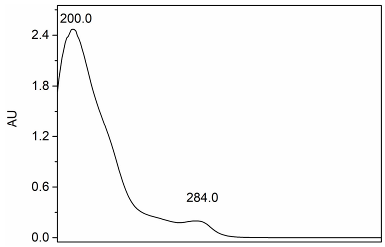

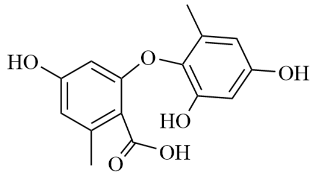

4

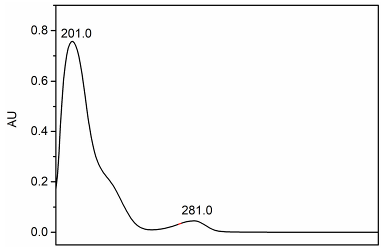

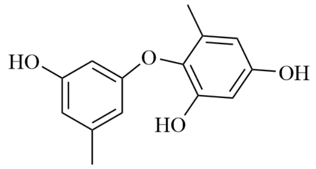

5

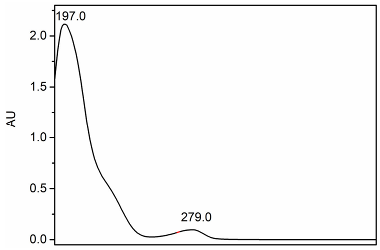

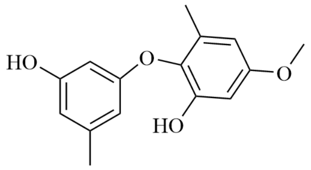

6

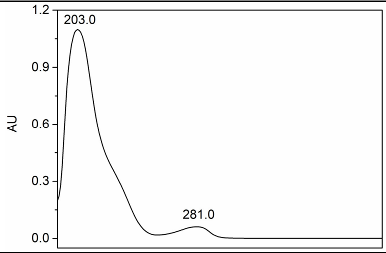

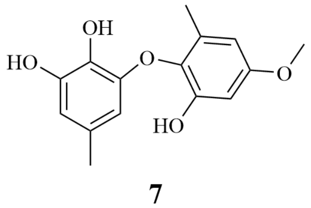

7

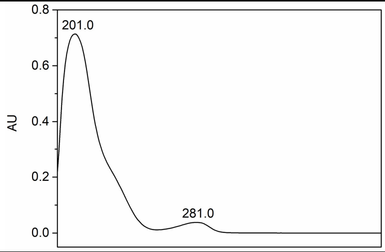

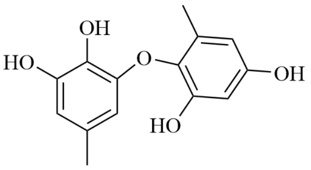

8

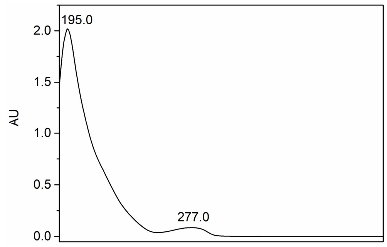

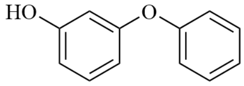

9

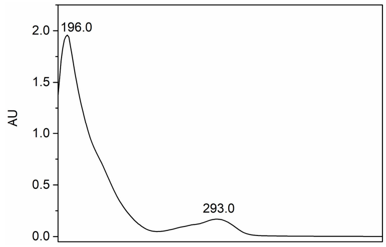

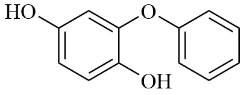

9a

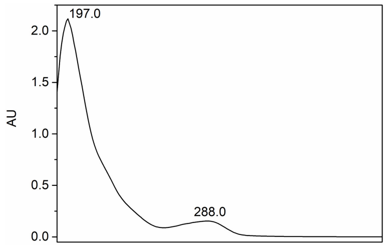

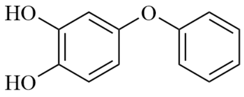

9b

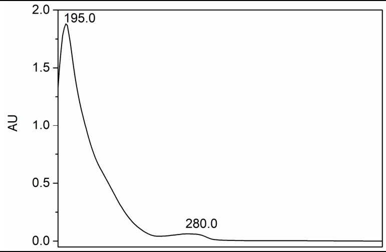

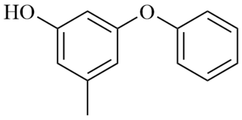

10

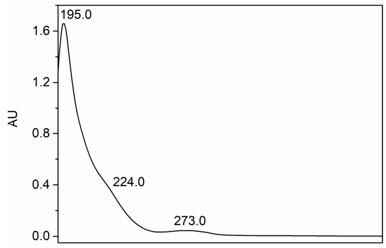

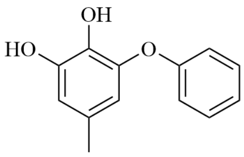

10a

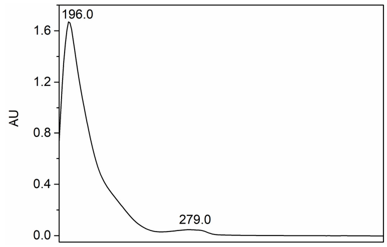

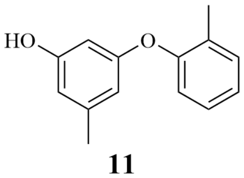

11

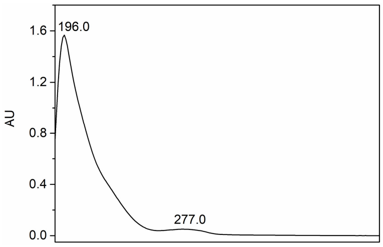

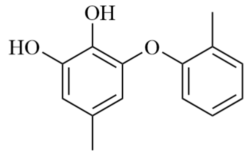

11a

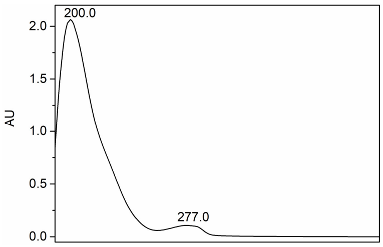

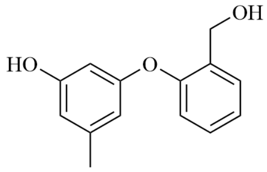

11c

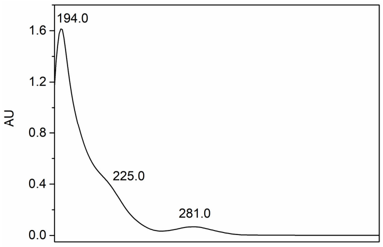

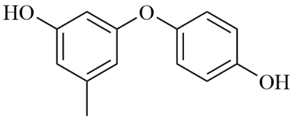

12

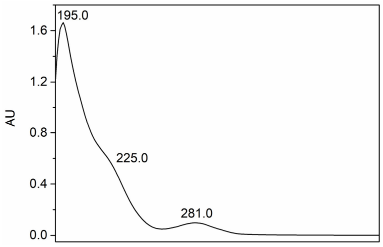

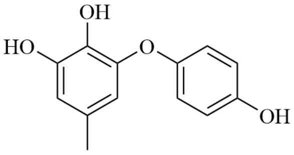

12a

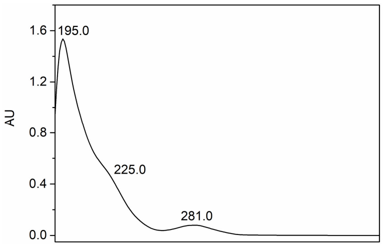

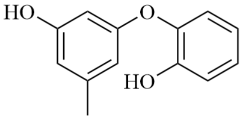

13

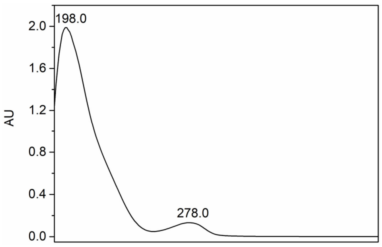

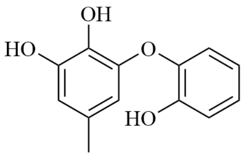

13a

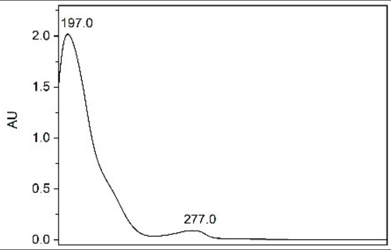

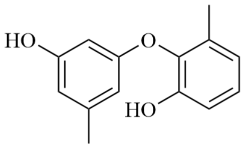

14

UV spectrum	Structure
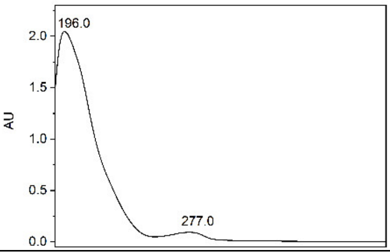	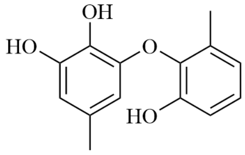 **14a**
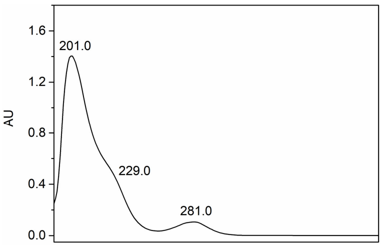	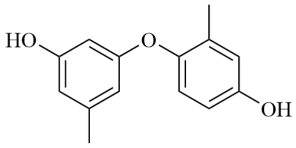 **15**
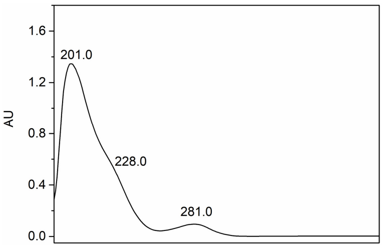	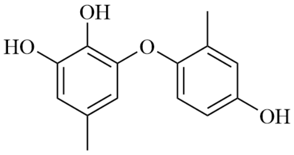 **15a**
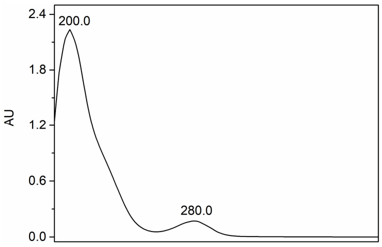	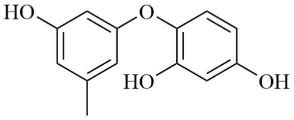 **16**
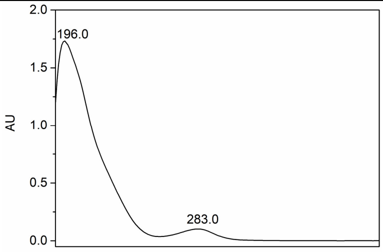	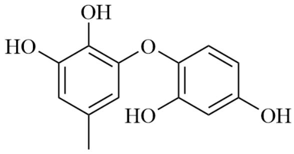 **16a**
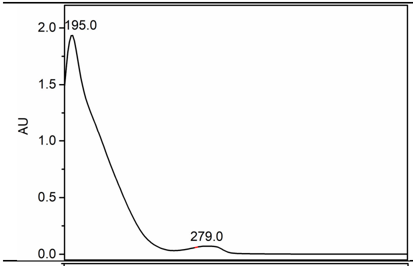	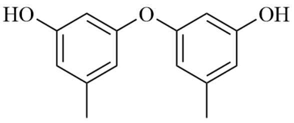 **17**
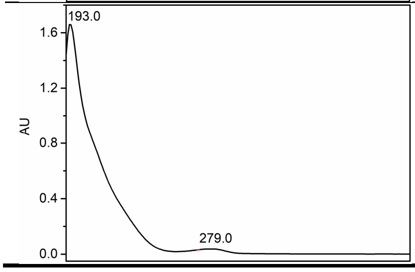	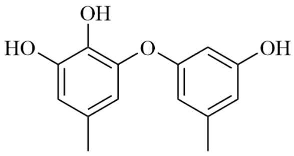 **17a**

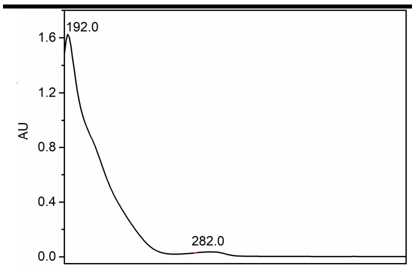

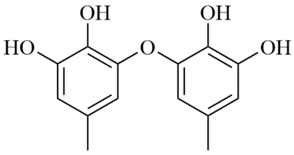

17b

Figure S16. HRESIMS spectra of the DEPs and DPEs.

Figure S16.1. (-)-HRESIMS spectrum of **2**.

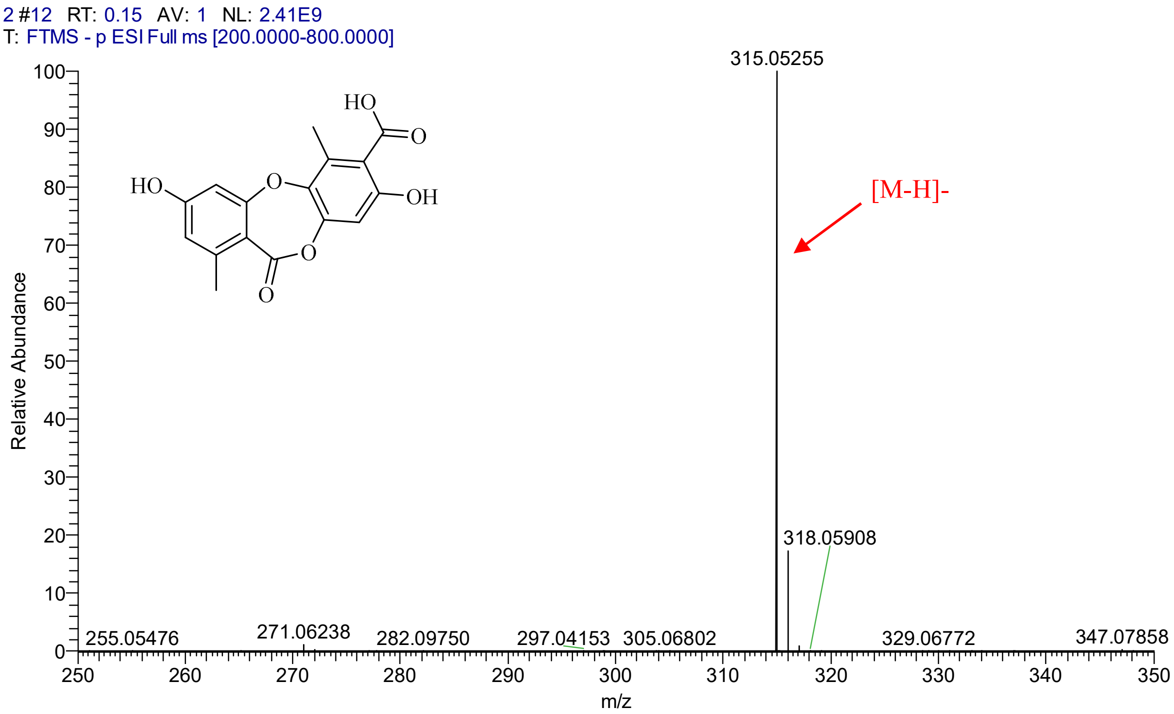

Figure S16.2. (+)-HRESIMS spectrum of **3**.

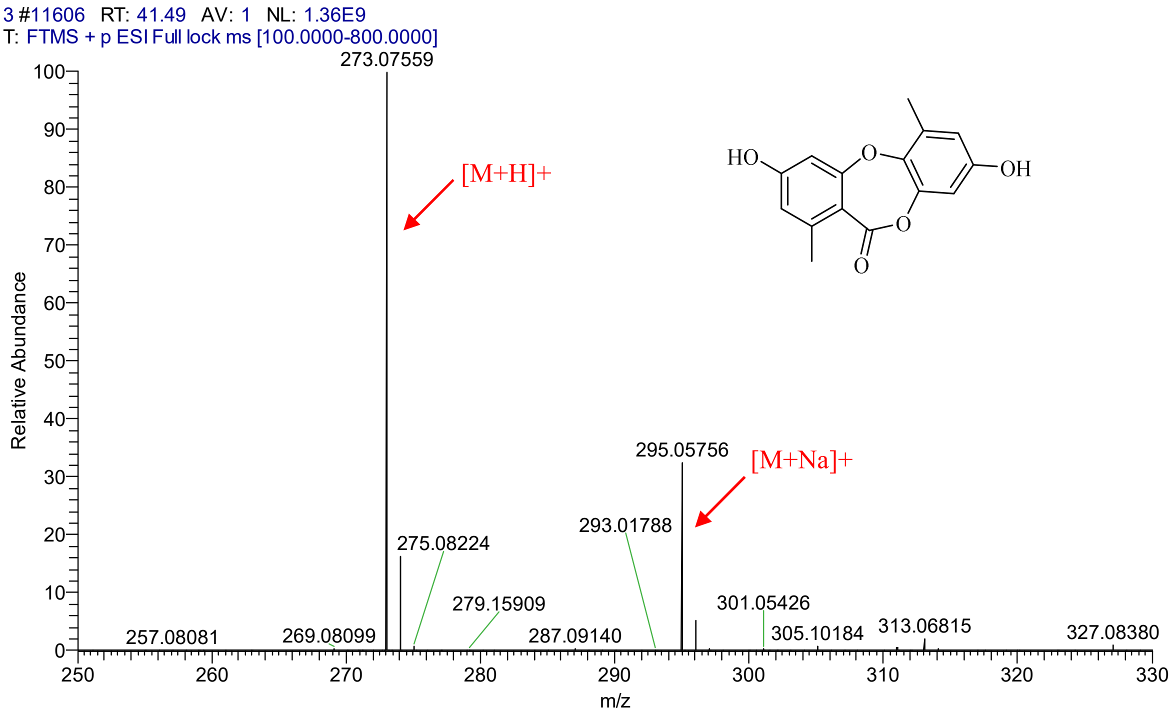

Figure S16.3. (+)-HRESIMS spectrum of **4**.

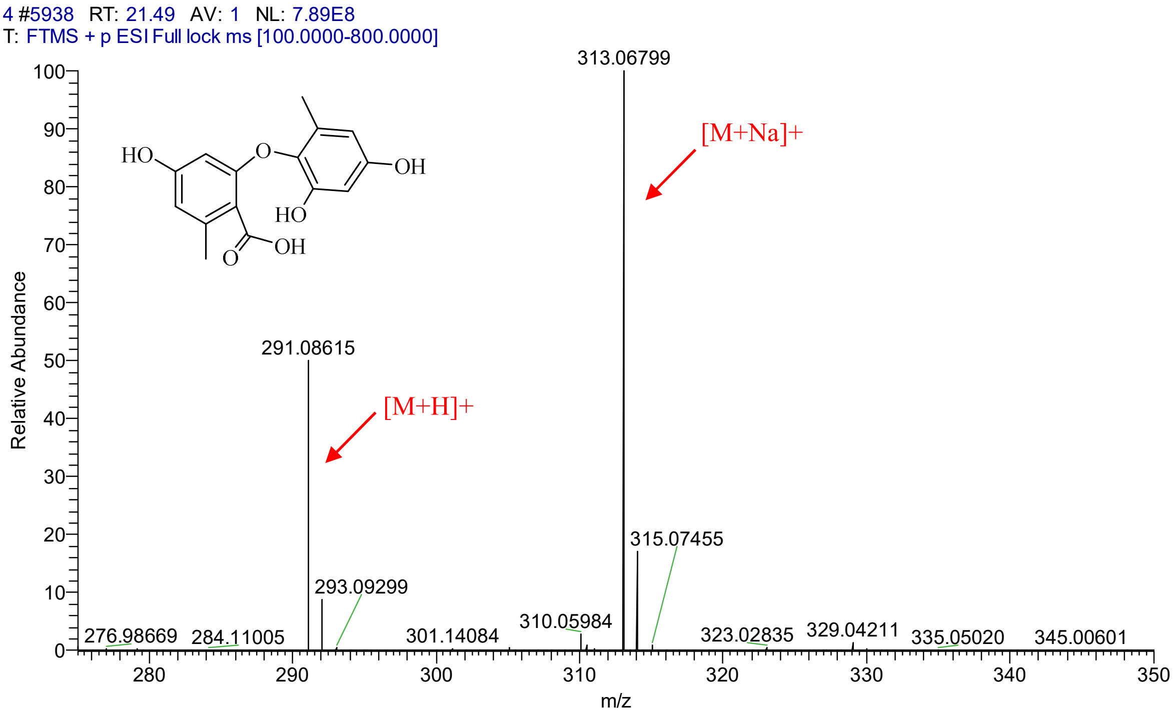

Figure S16.4. (+)-HRESIMS spectrum of **5**.

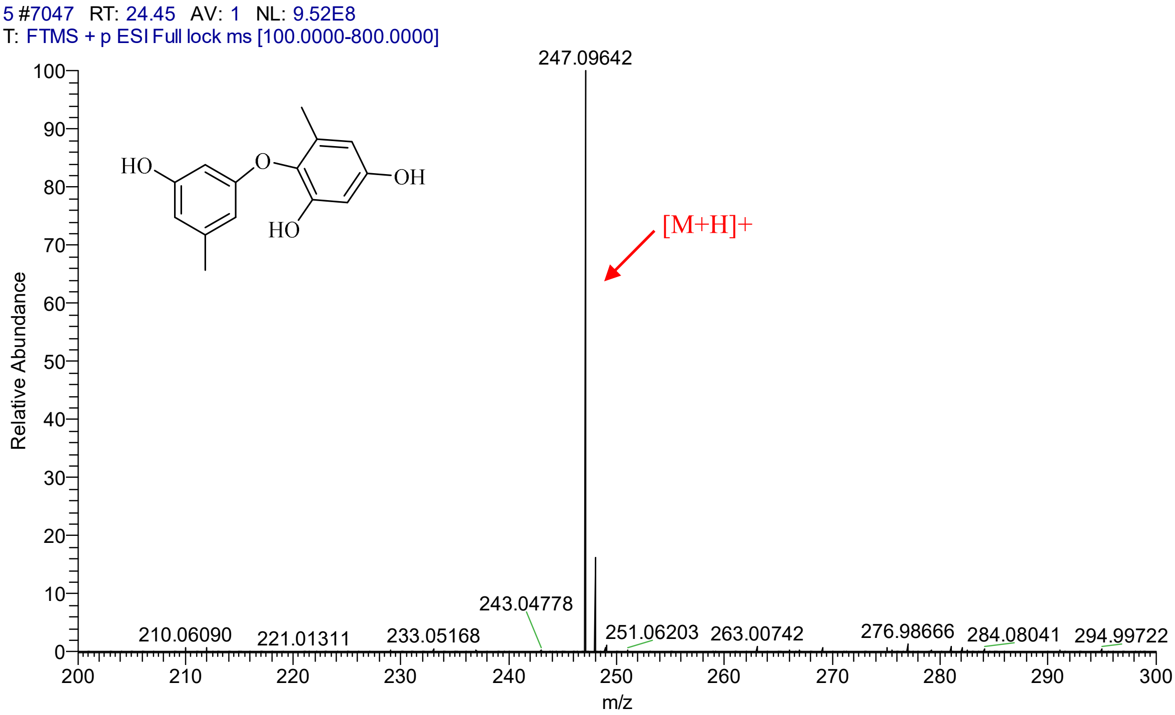

Figure S16.5. (+)-HRESIMS spectrum of **6**.

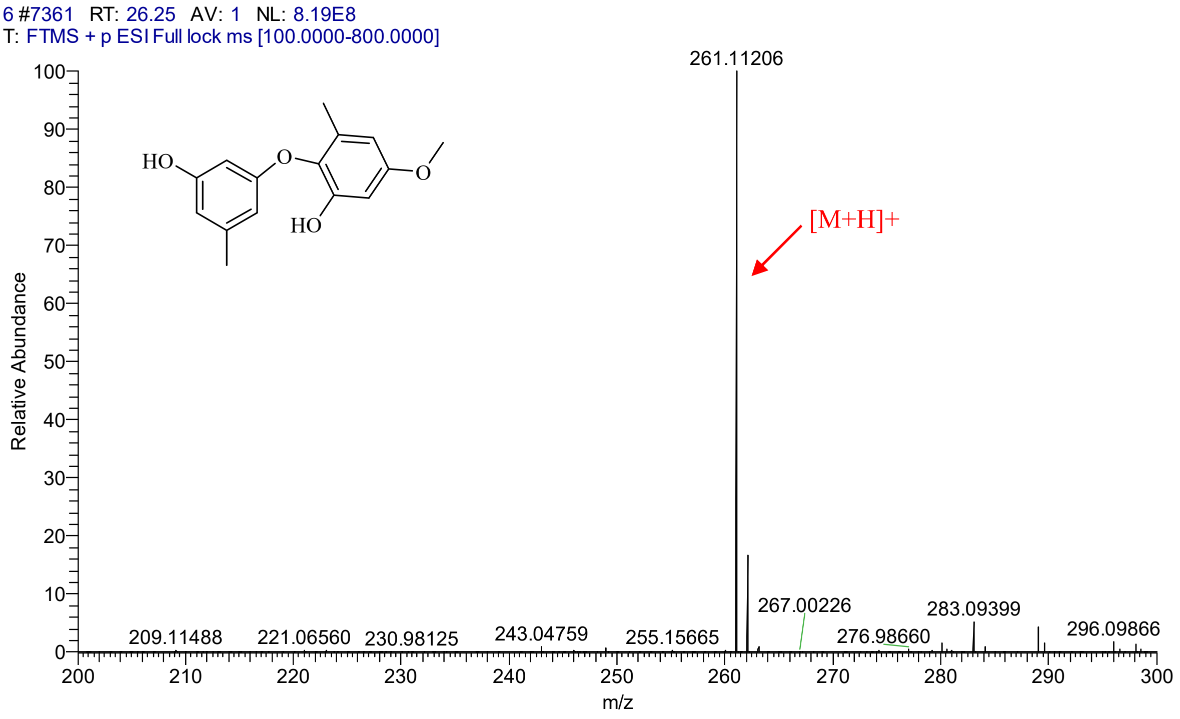

Figure S16.6. (-)-HRESIMS spectrum of **7**.

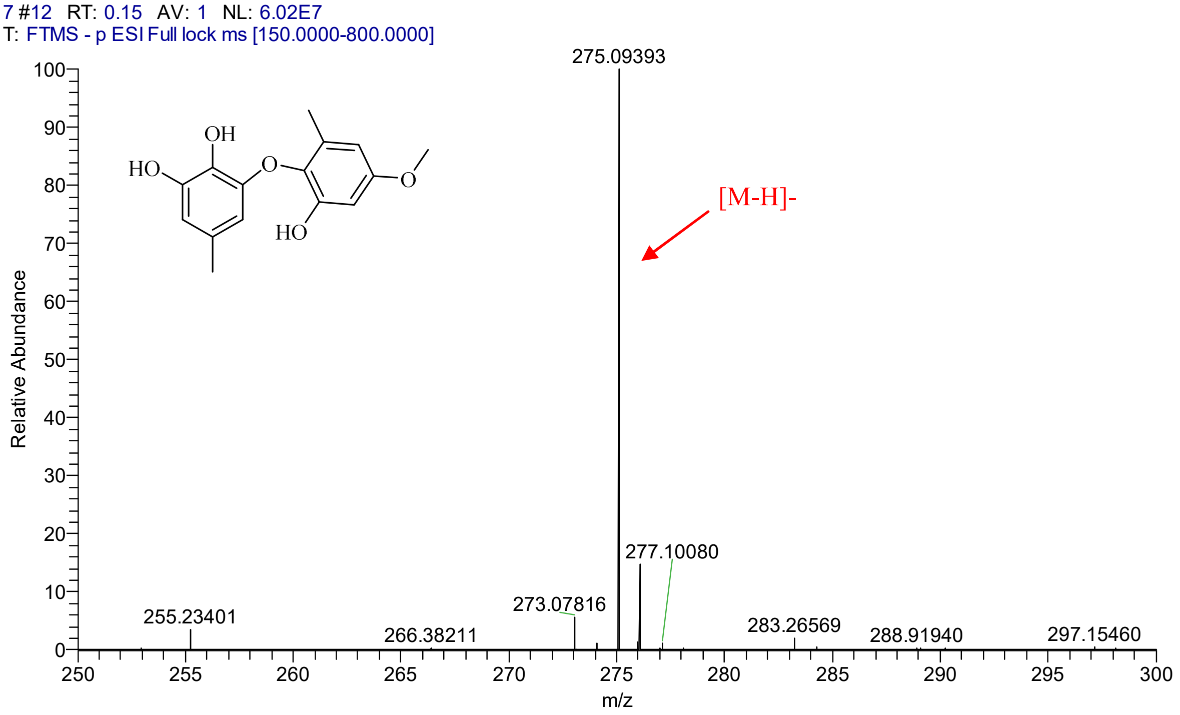

Figure S16.7. (+)-HRESIMS spectrum of **8**.

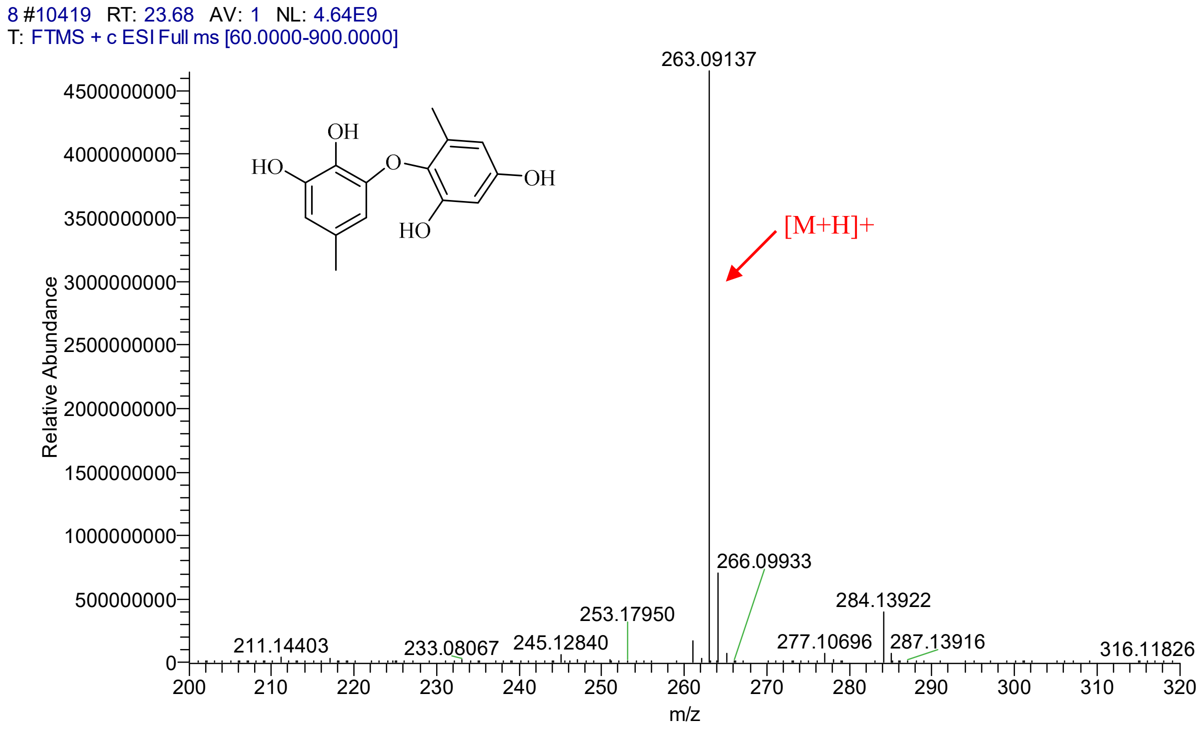

Figure S16.8. (-)-HRESIMS spectrum of **9a**.

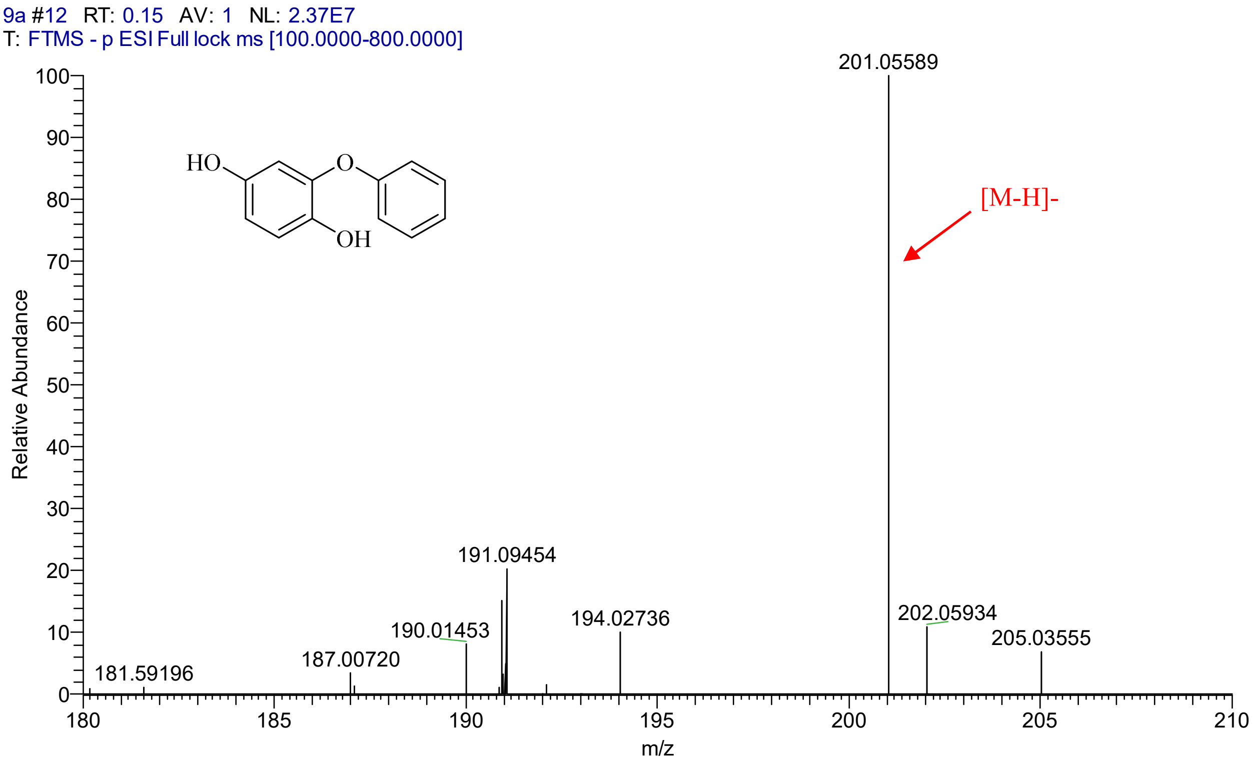

Figure S16.9. (-)-HRESIMS spectrum of **9b**.

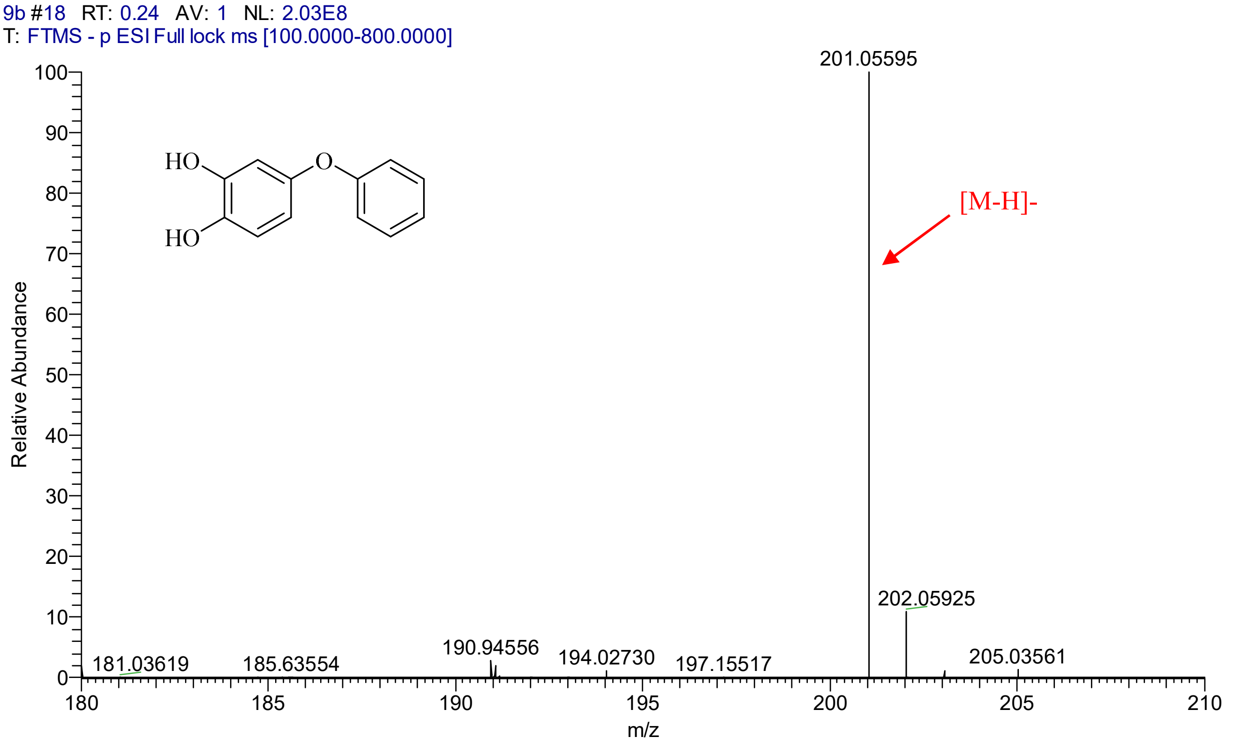

Figure S16.10. (-)-HRESIMS spectrum of **10**.

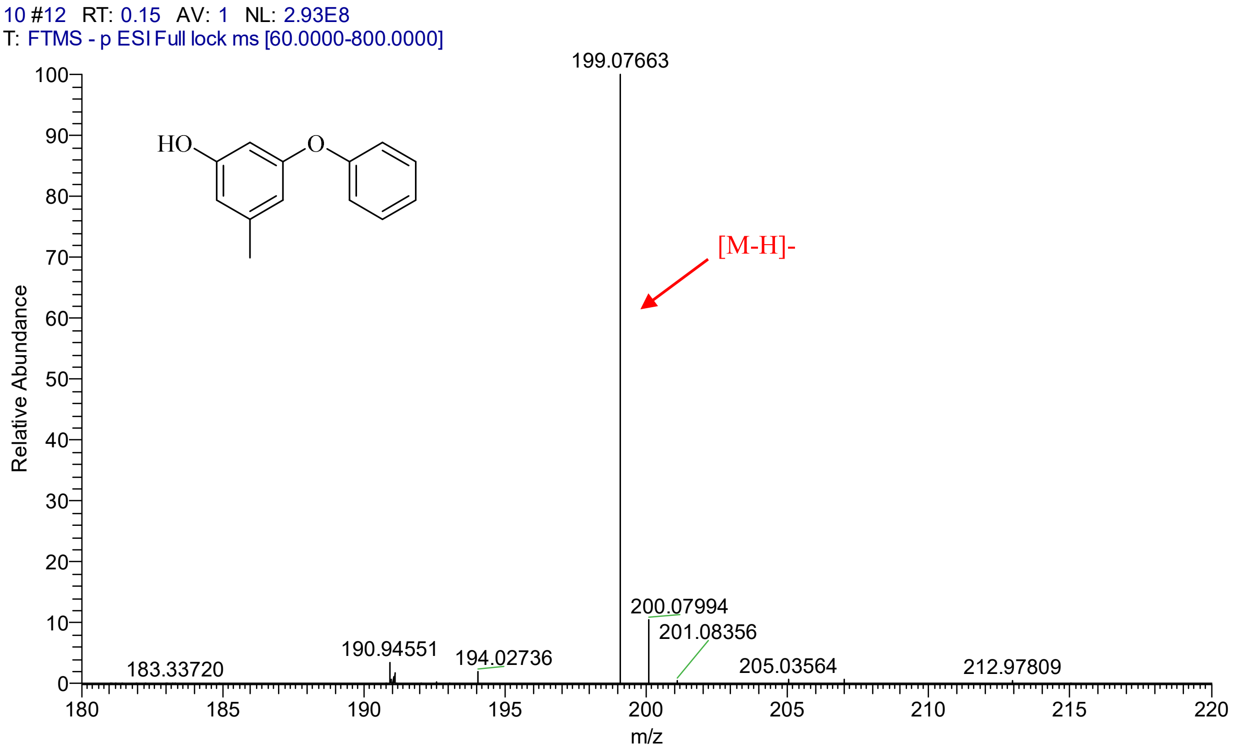

Figure S16.11. (-)-HRESIMS spectrum of **10a**.

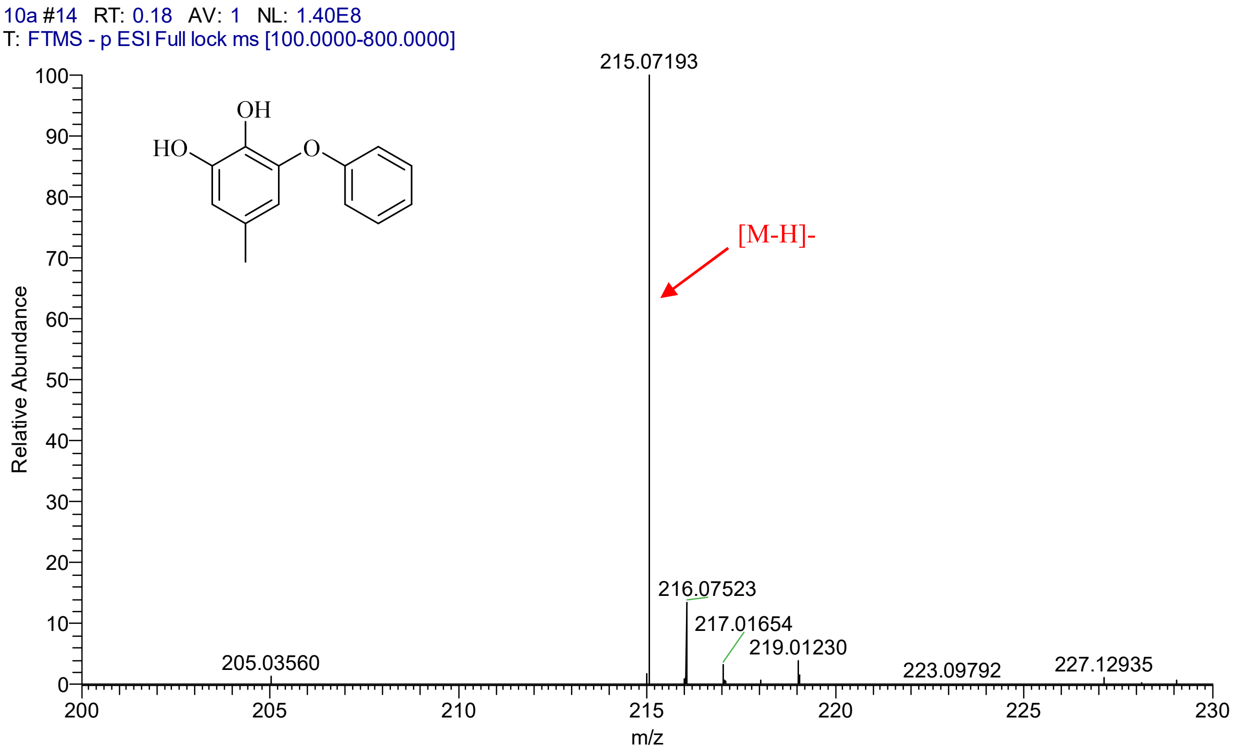

Figure S16.12. (+)-HRESIMS spectrum of **11**.

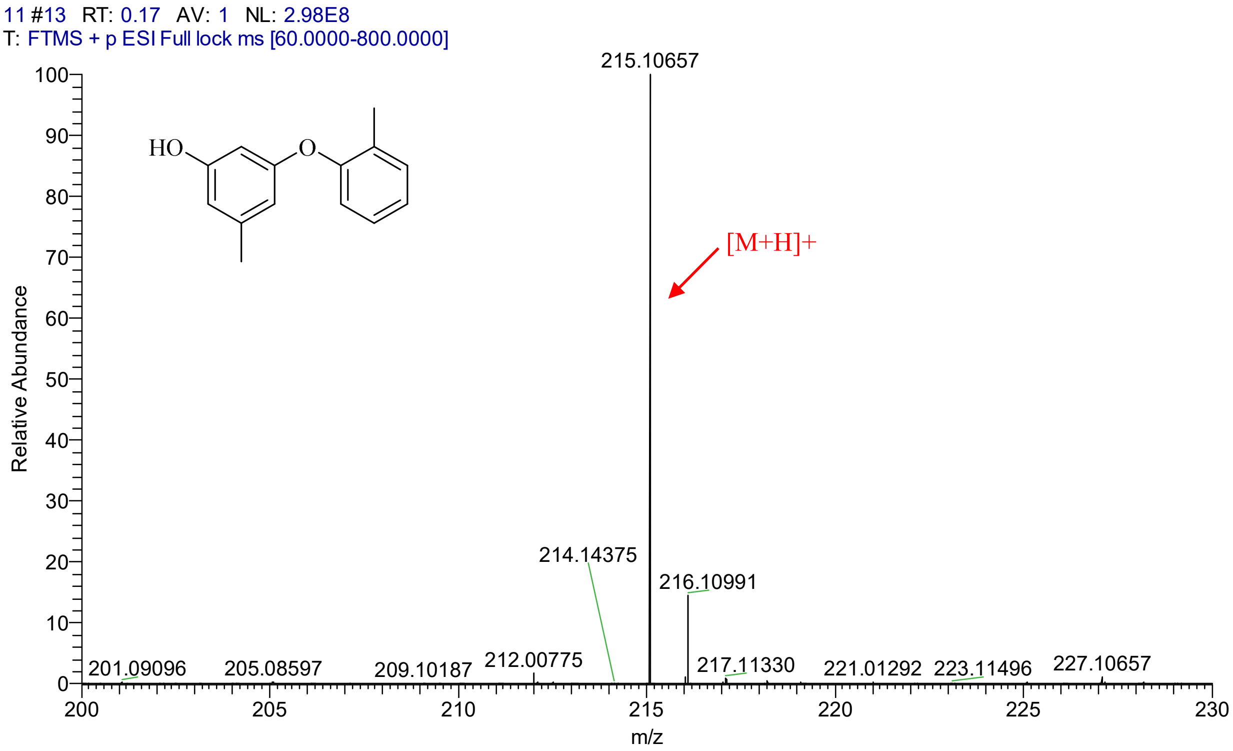

Figure S16.13. (-)-HRESIMS spectrum of **11a**.

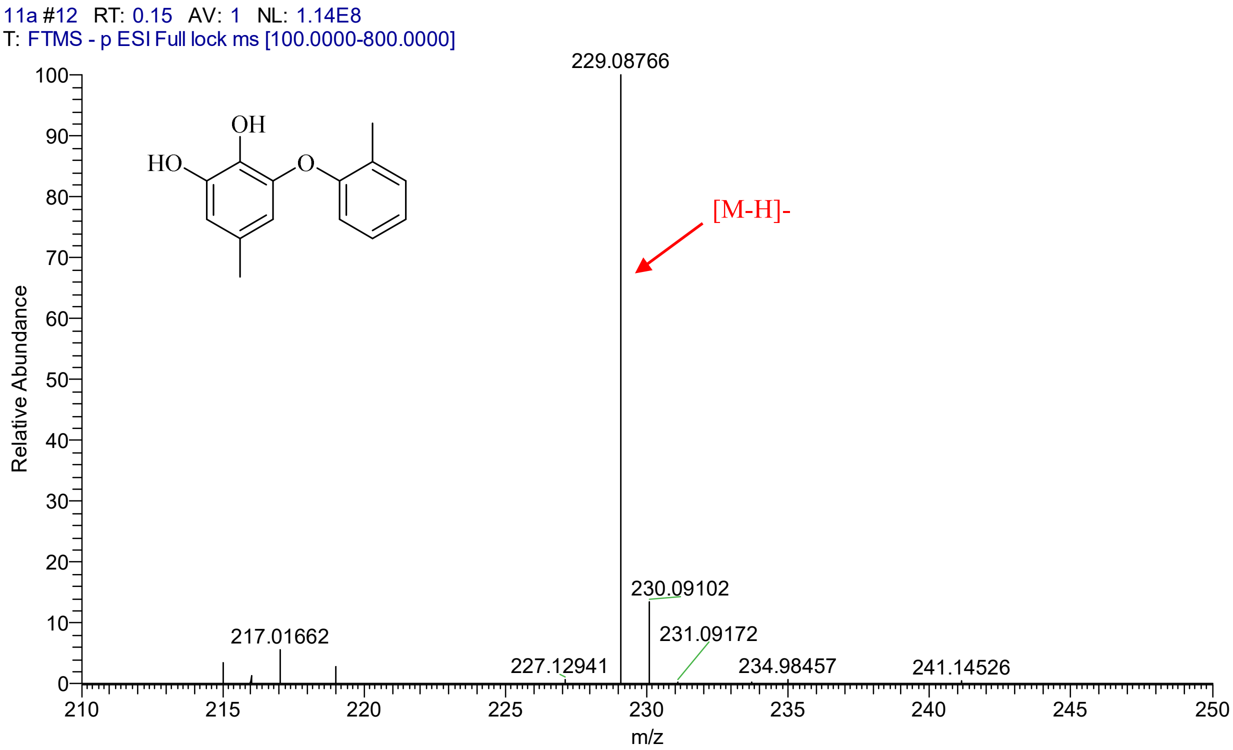

Figure S16.14. (-)-HRESIMS spectrum of **11c**.

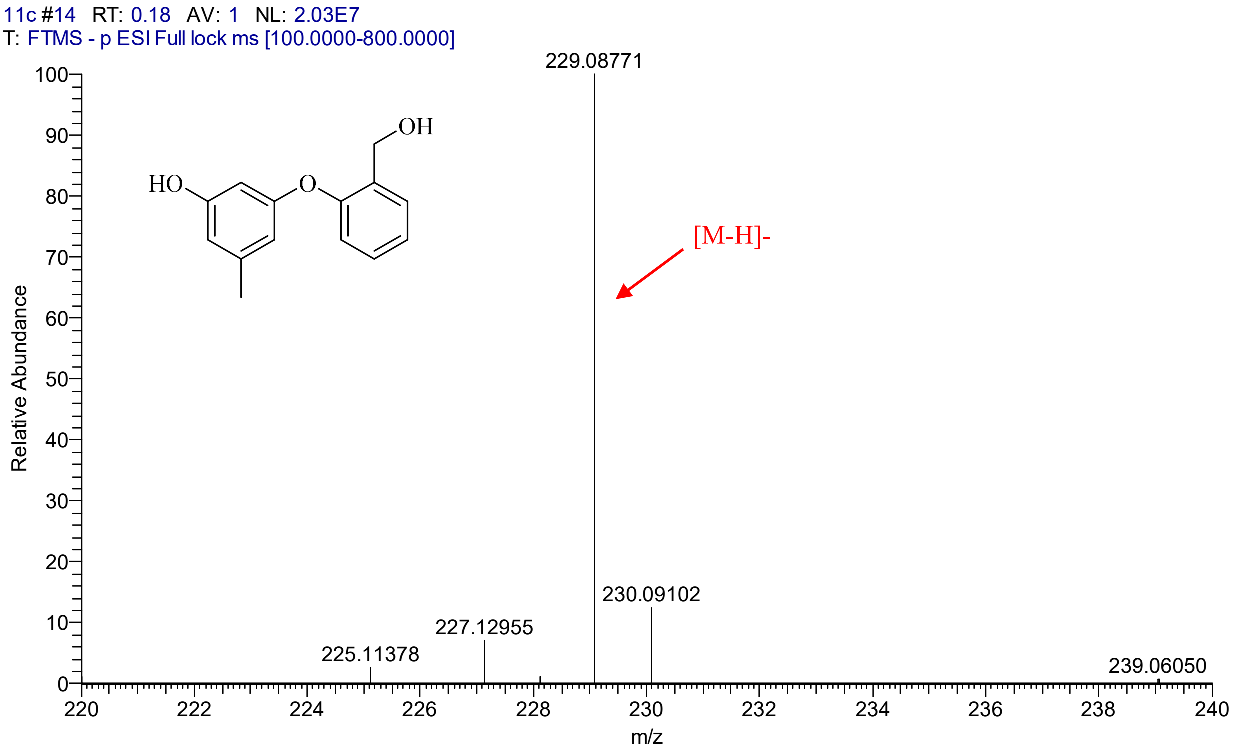

Figure S16.15. (-)-HRESIMS spectrum of **12**.

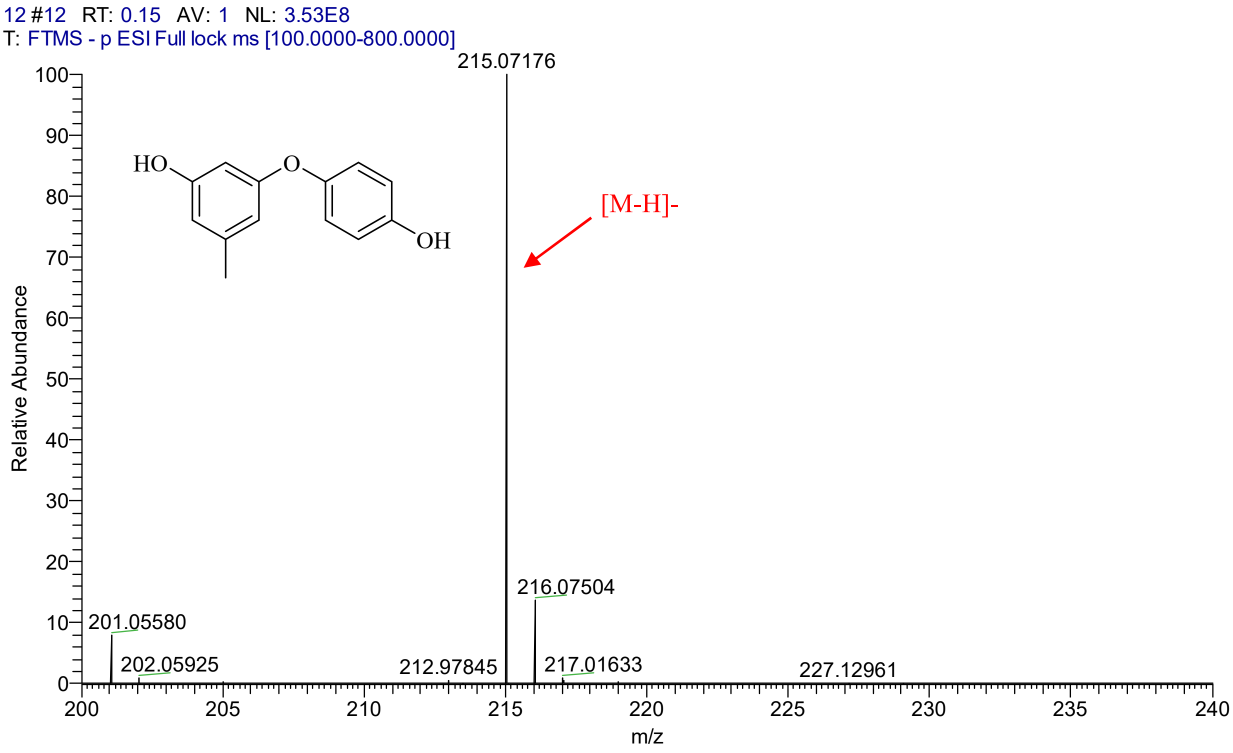

Figure S16.16. (-)-HRESIMS spectrum of **12a**.

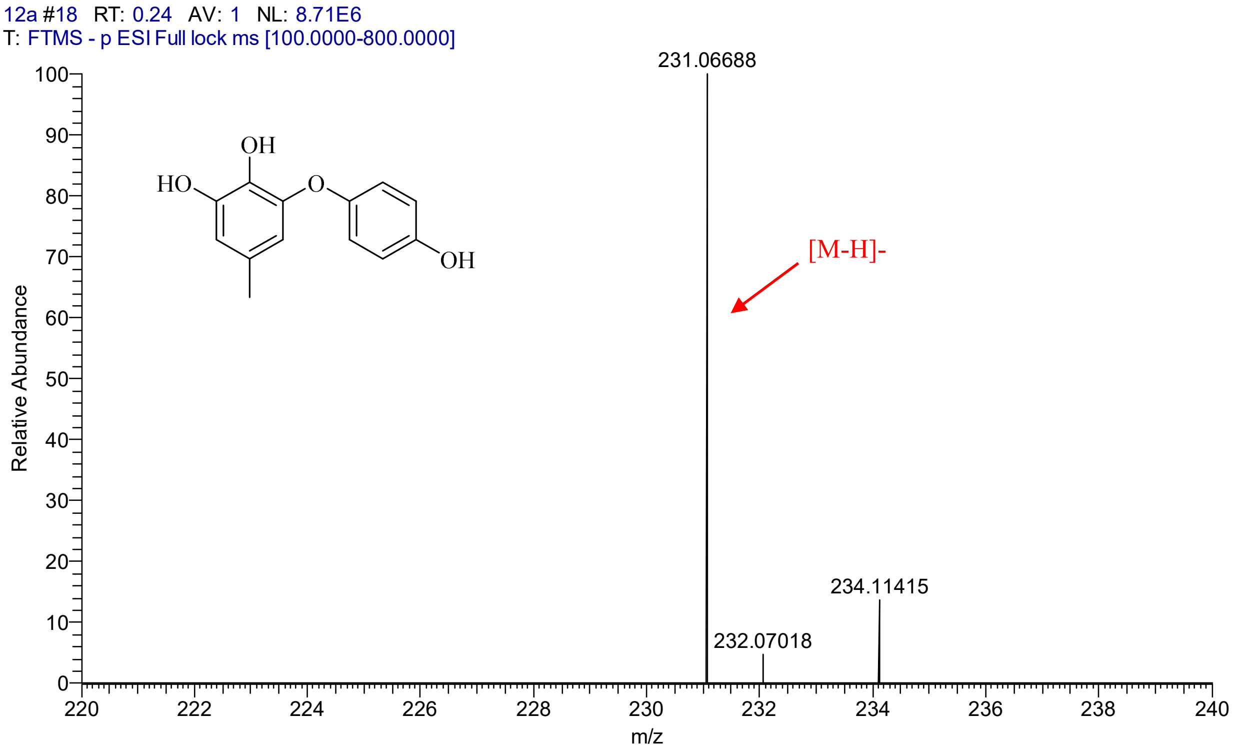

Figure S16.17. (-)-HRESIMS spectrum of **13**.

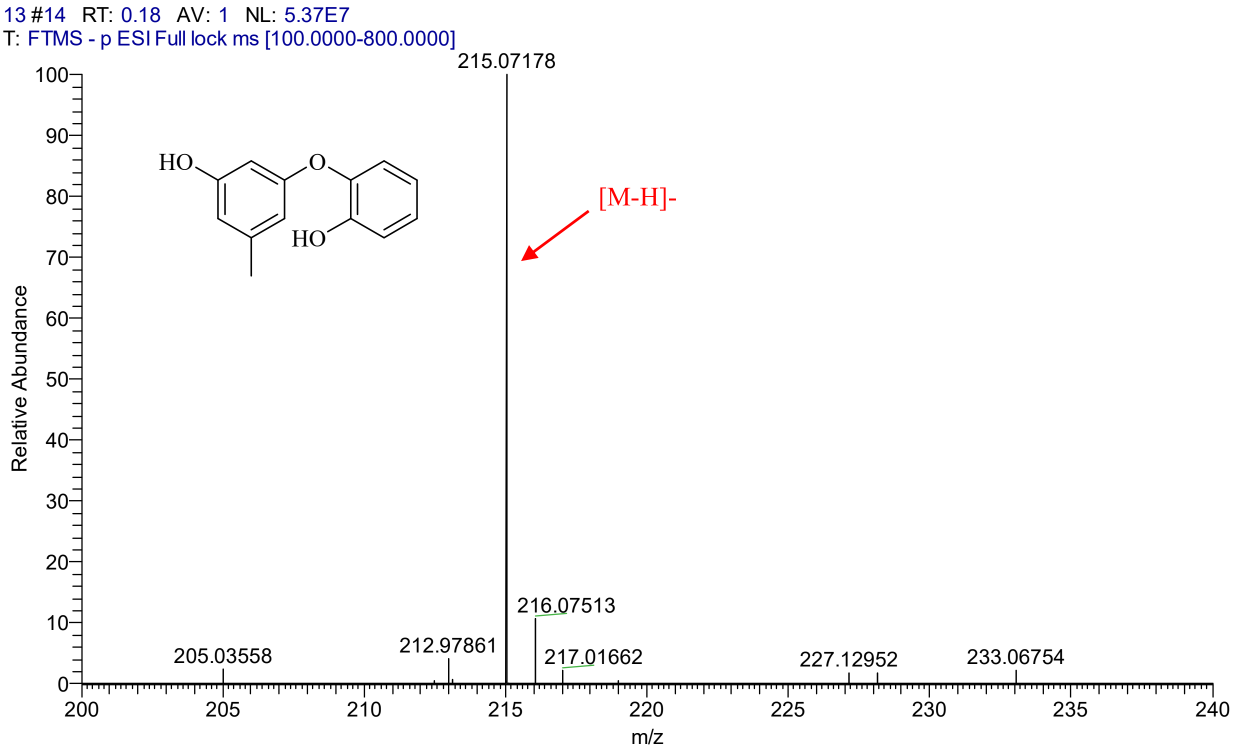

Figure S16.18. (-)-HRESIMS spectrum of **13a**.

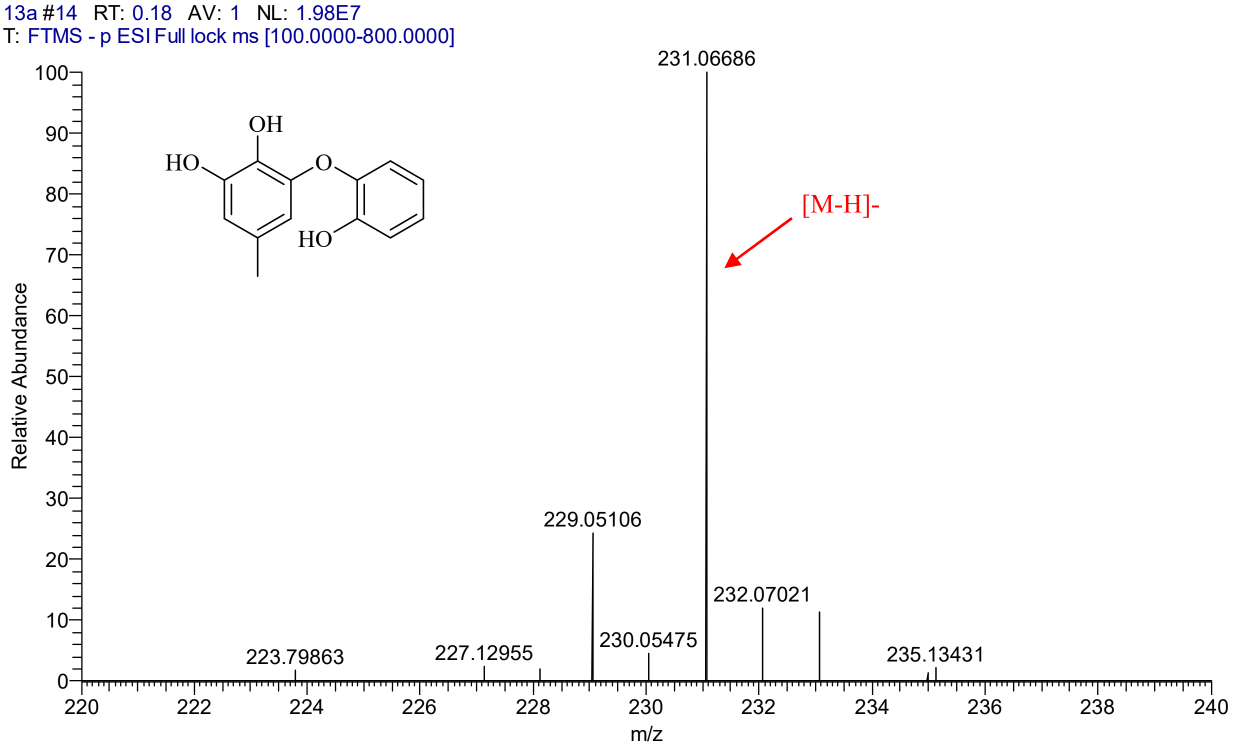

Figure S16.19. (-)-HRESIMS spectrum of **14**.

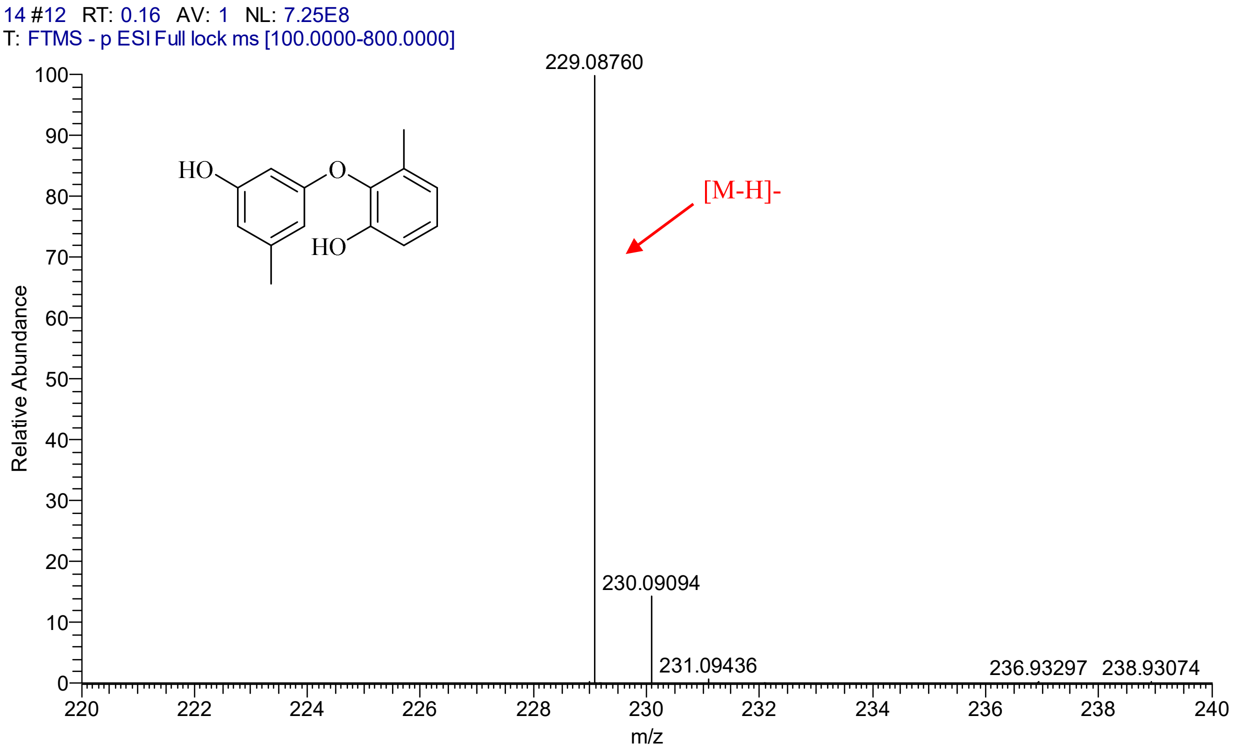

Figure S16.20. (-)-HRESIMS spectrum of **14a**.

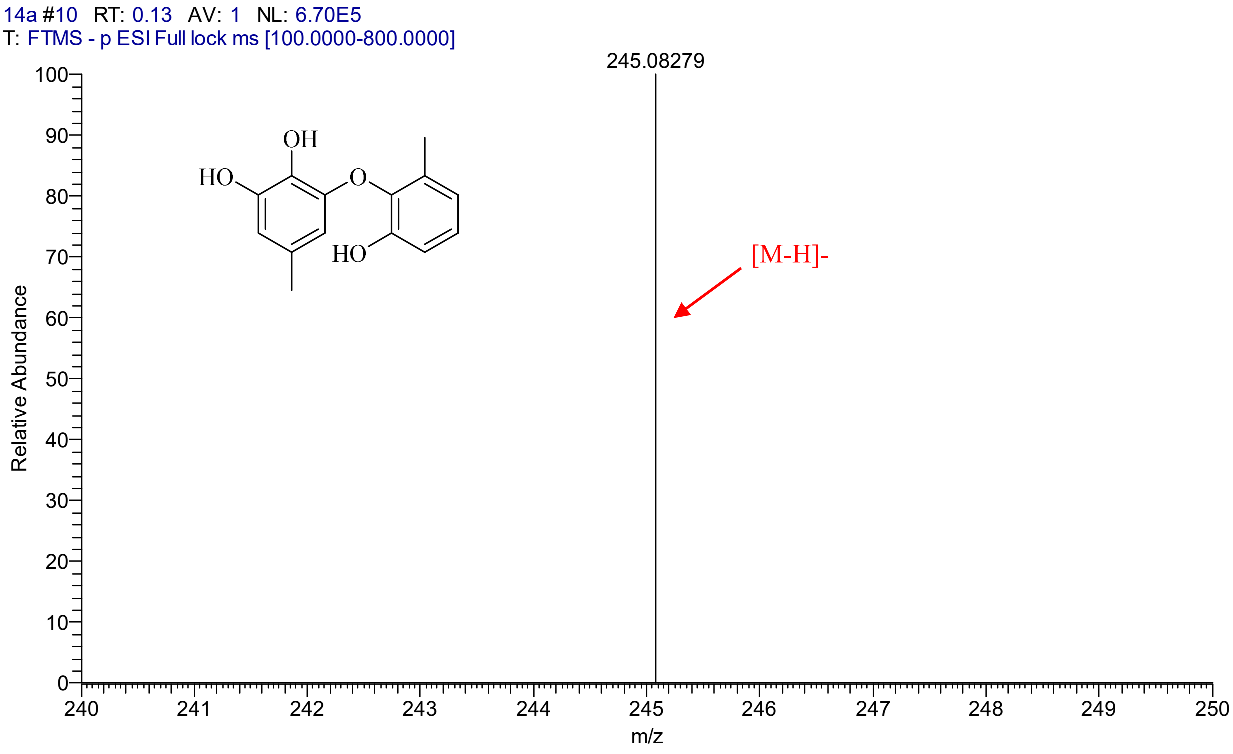

Figure S16.21. (-)-HRESIMS spectrum of **15**.

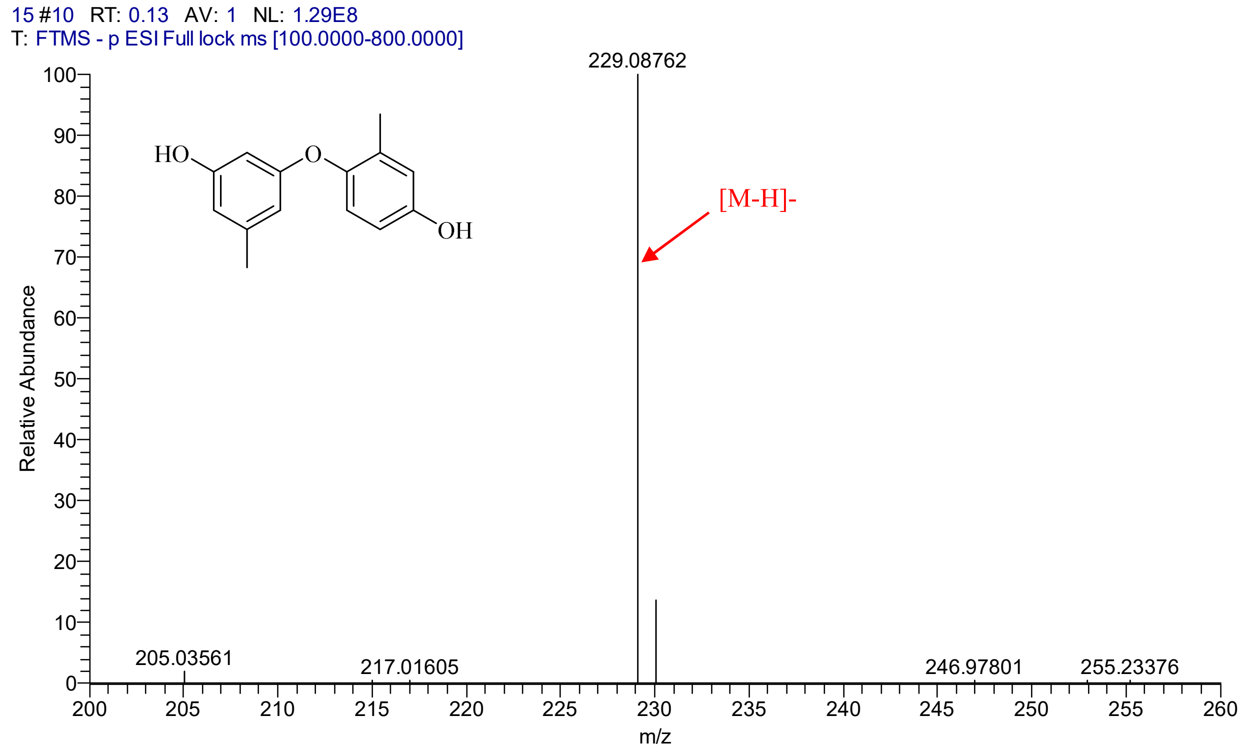

Figure S16.22. (-)-HRESIMS spectrum of **15a**.

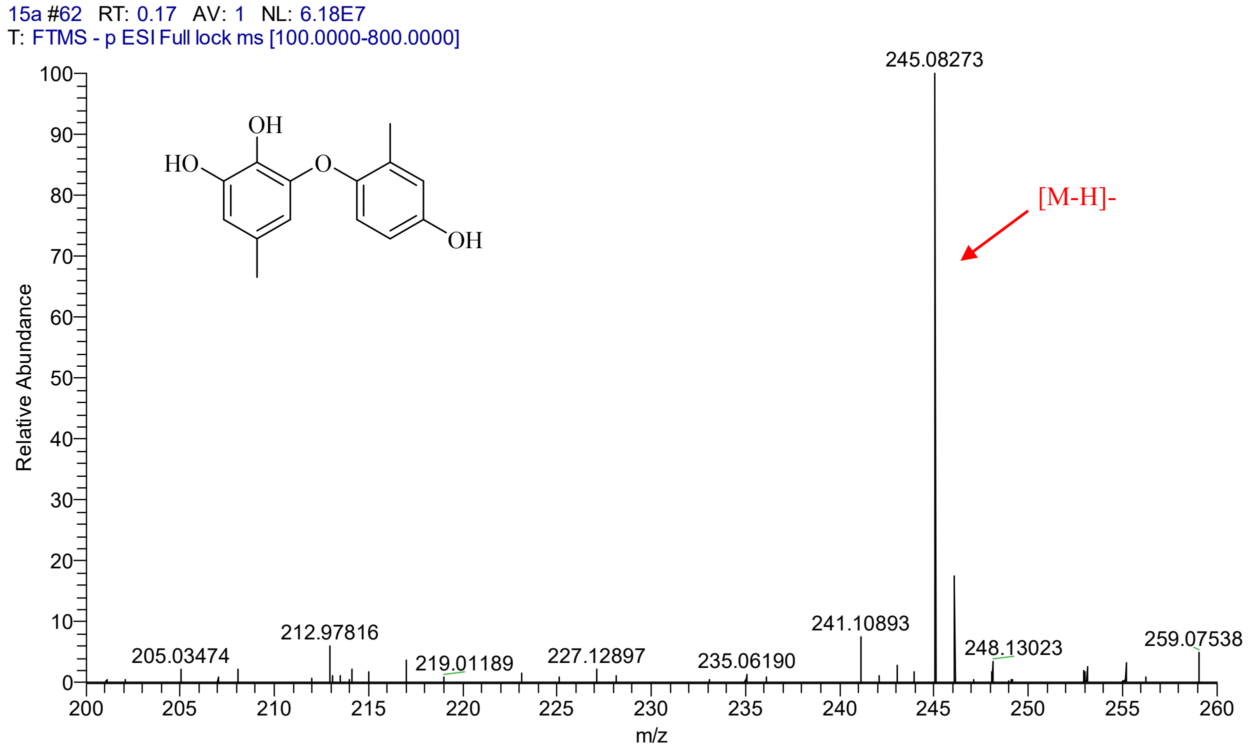

Figure S16.23. (-)-HRESIMS spectrum of **16**.

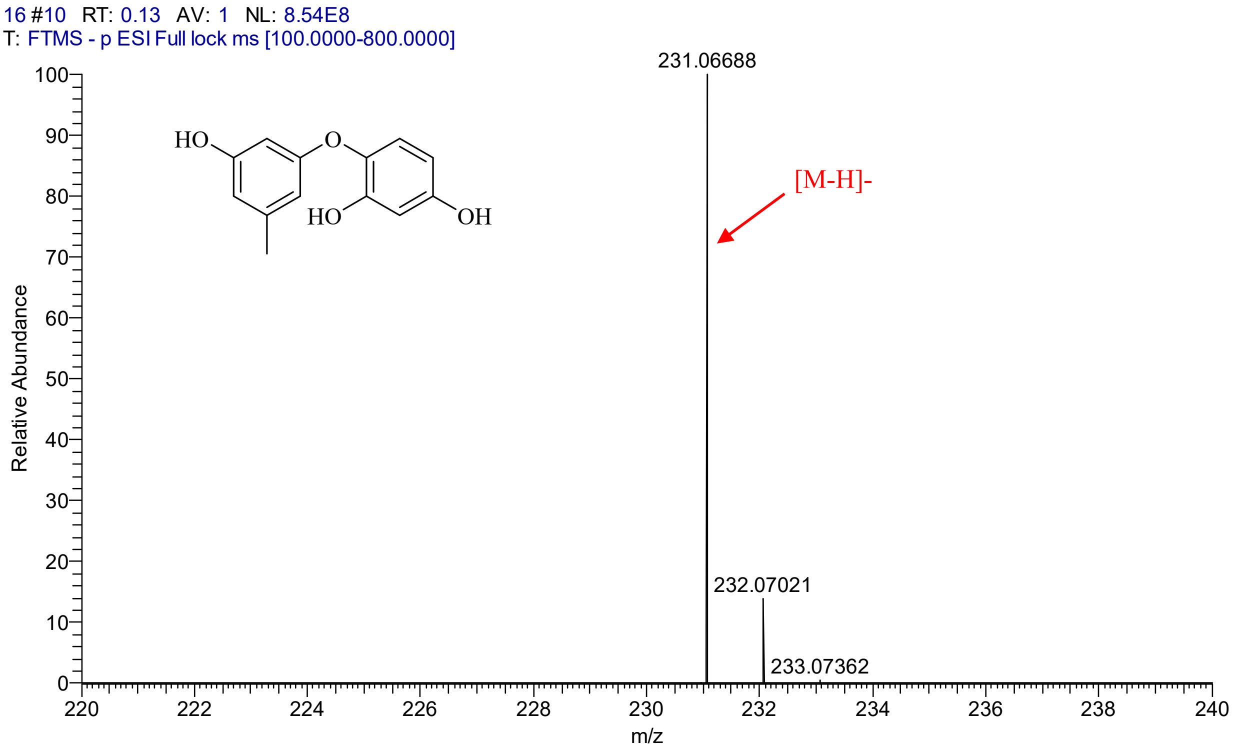

Figure S16.24. (-)-HRESIMS spectrum of **16a**.

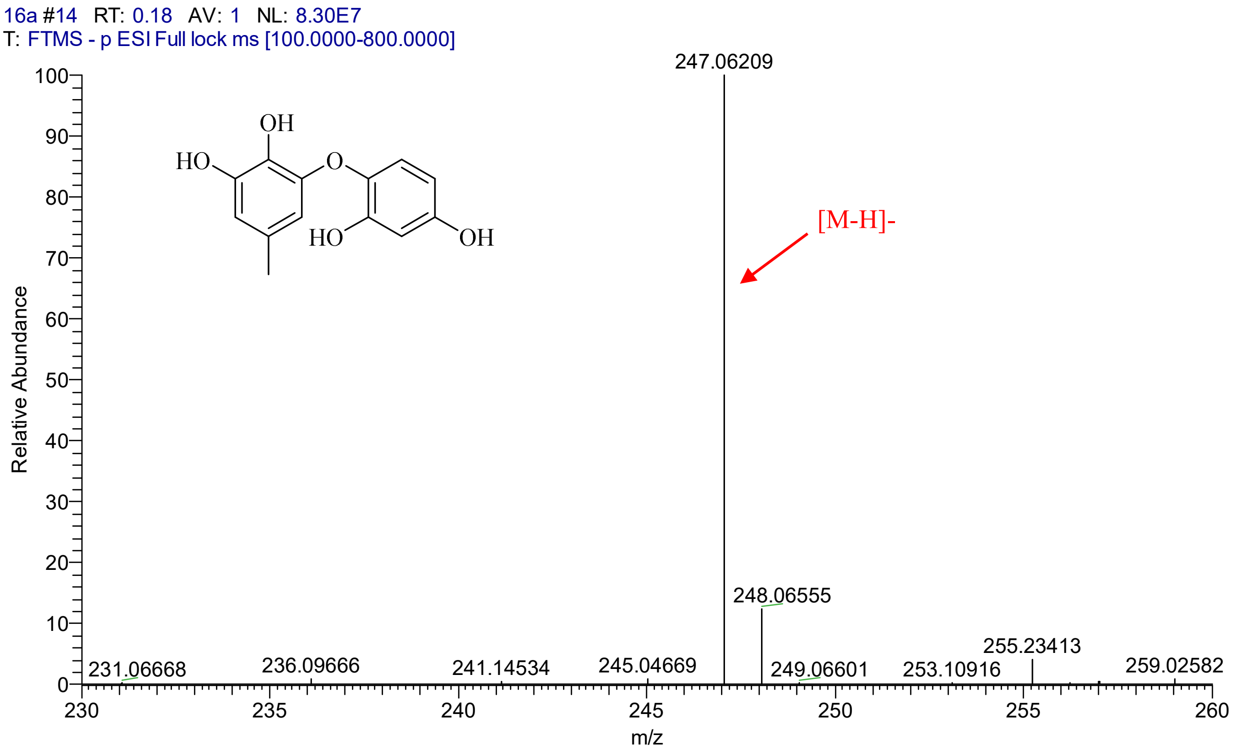

Figure S16.25. (-)-HRESIMS spectrum of **17**.

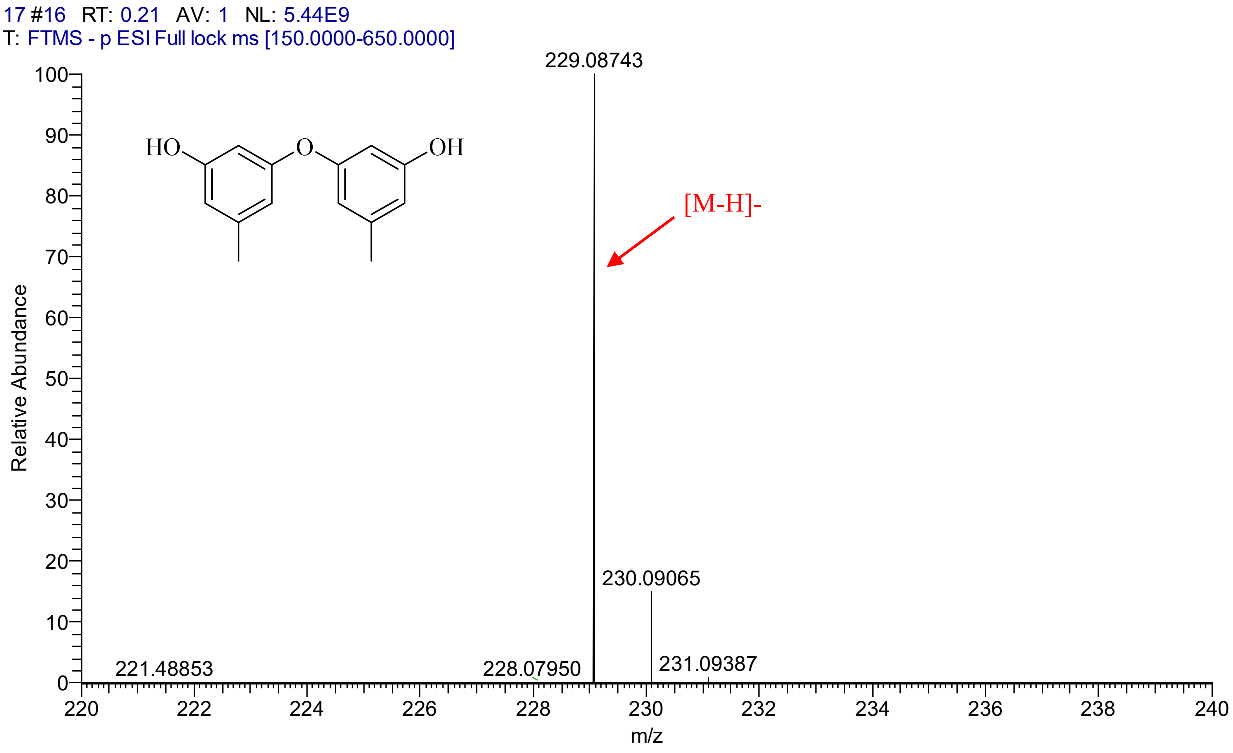

Figure S16.26. (-)-HRESIMS spectrum of **17a**.

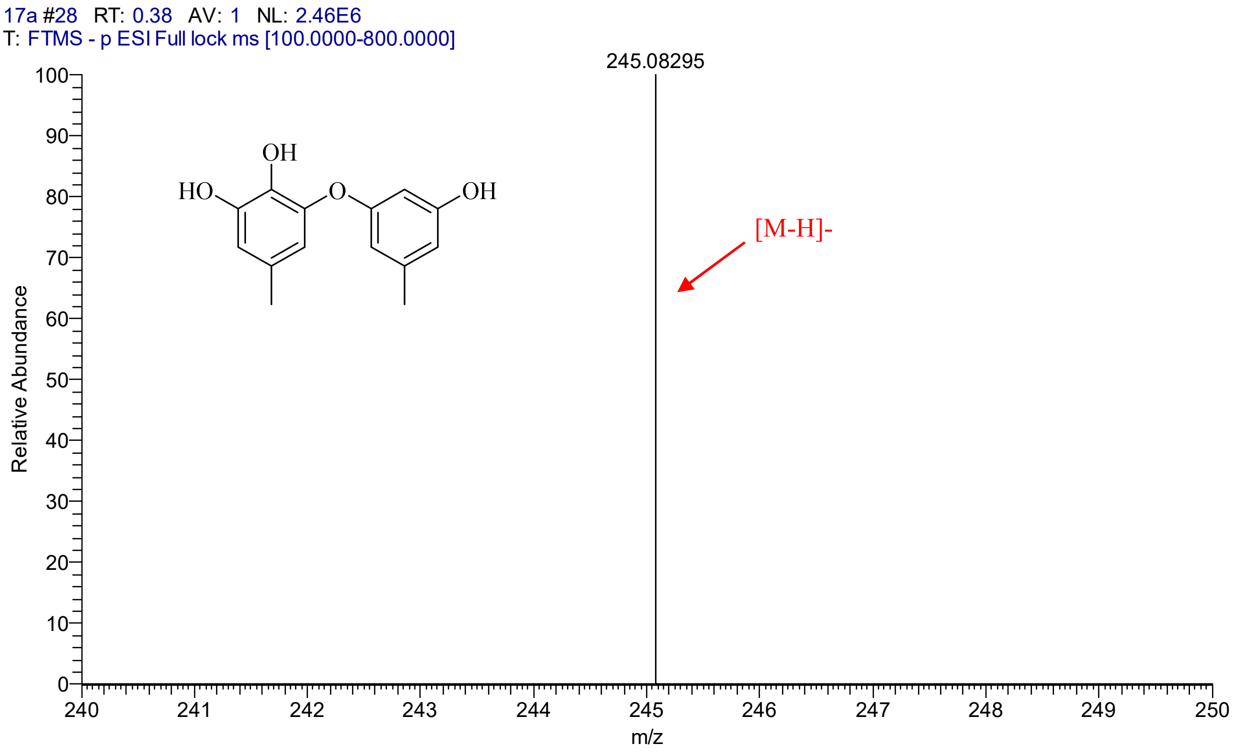

Figure S16.27. (-)-HRESIMS spectrum of **17b**.

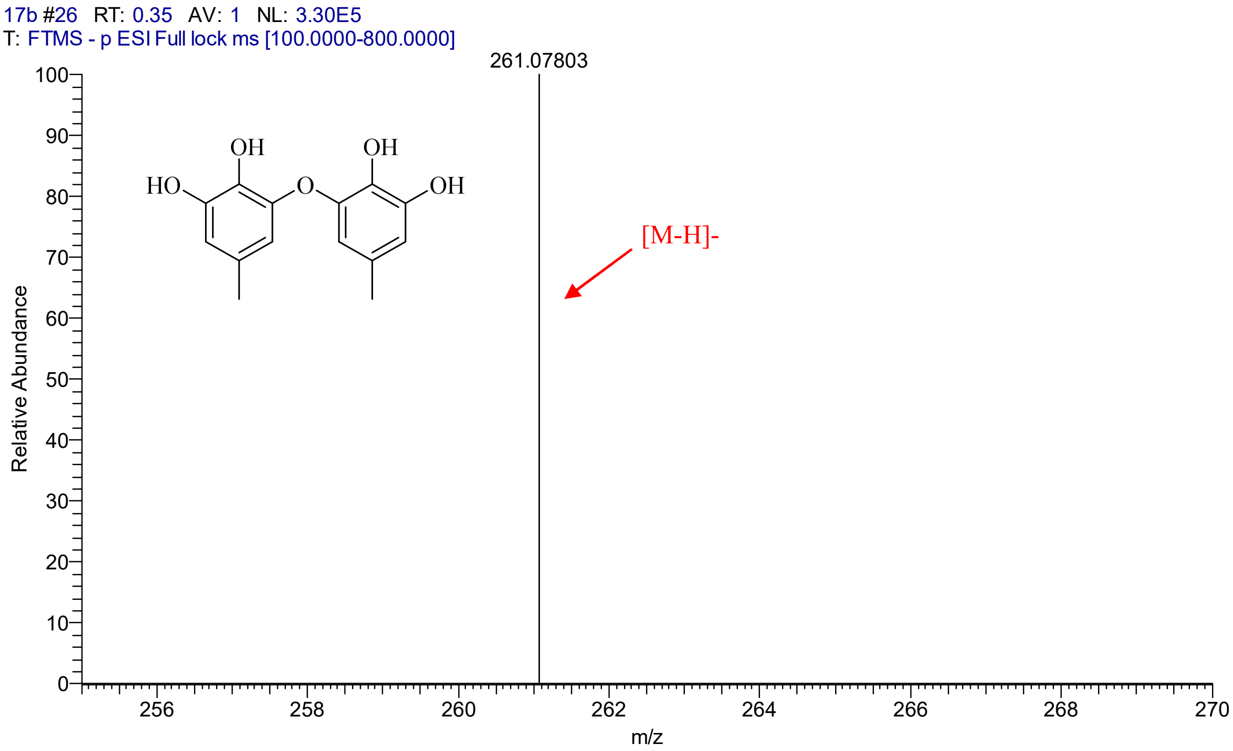

Figure S16.28. (+)-HRESIMS spectrum of **18**.

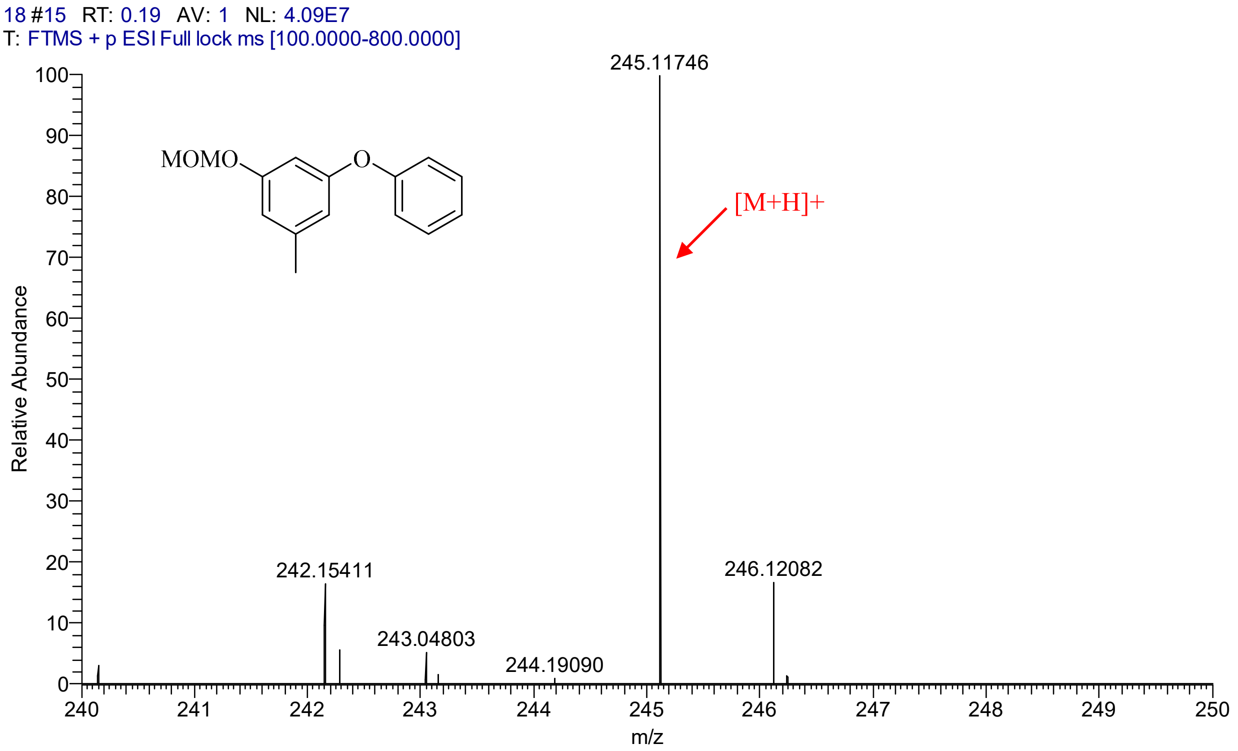

Figure S16.29. (+)-HRESIMS spectrum of **19**.

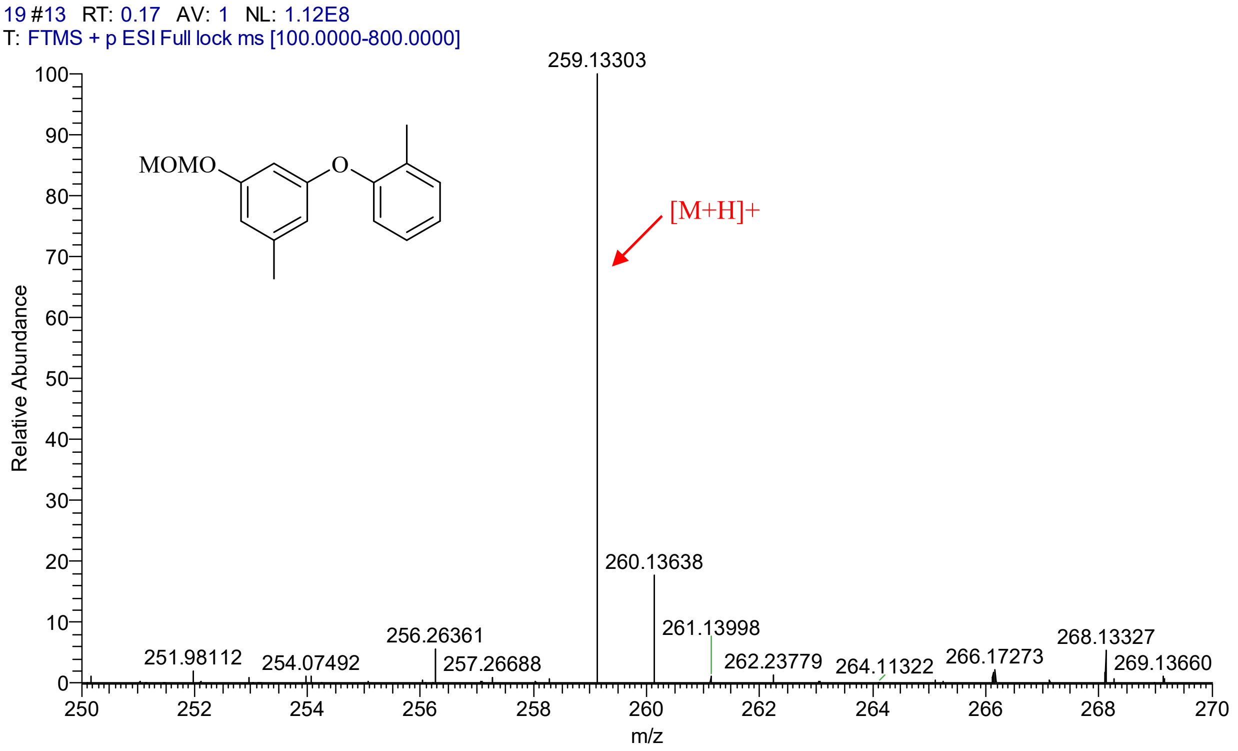

Figure S16.30. (+)-HRESIMS spectrum of **20**.

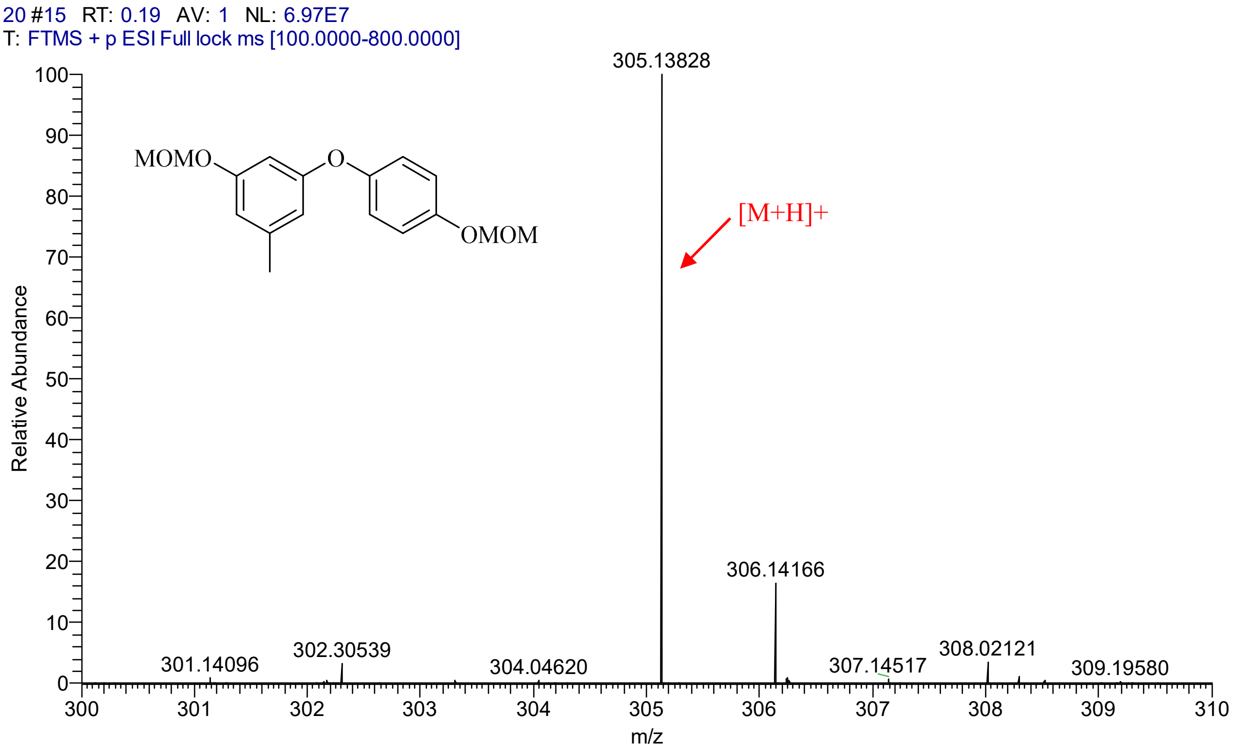

Figure S16.31. (+)-HRESIMS spectrum of **21**.

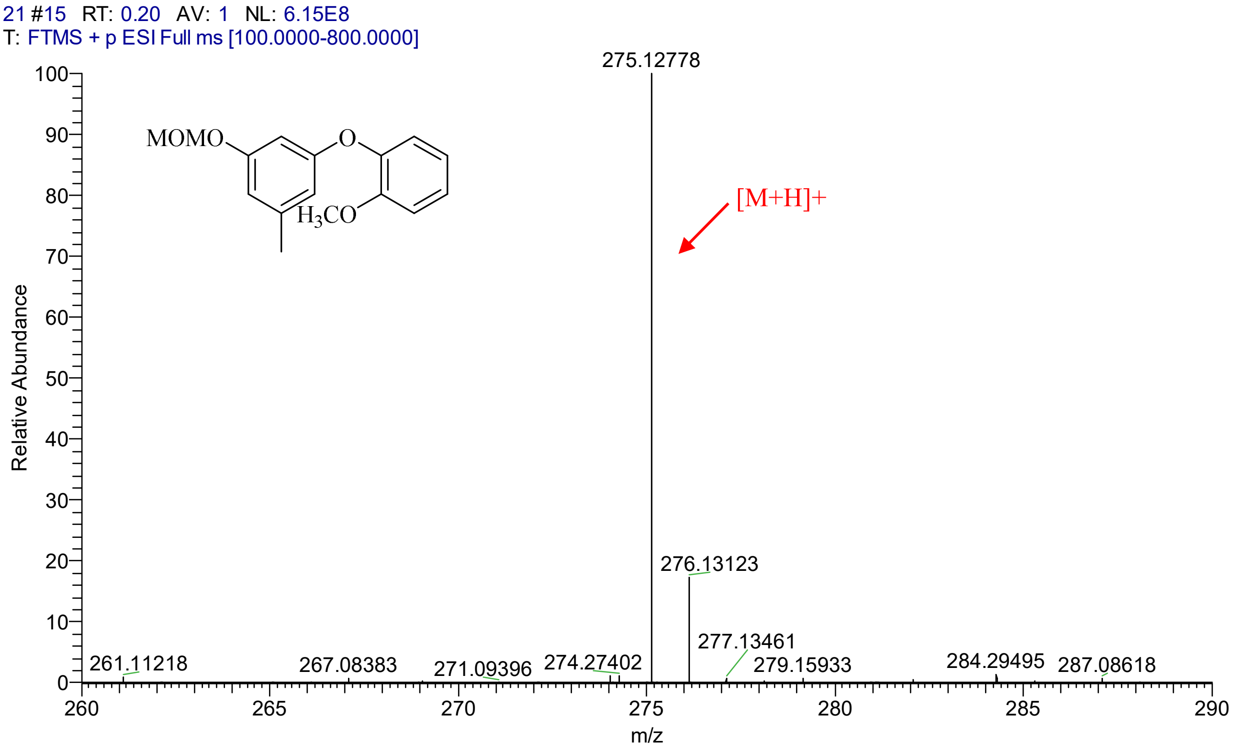

Figure S16.32. (+)-HRESIMS spectrum of **22**.

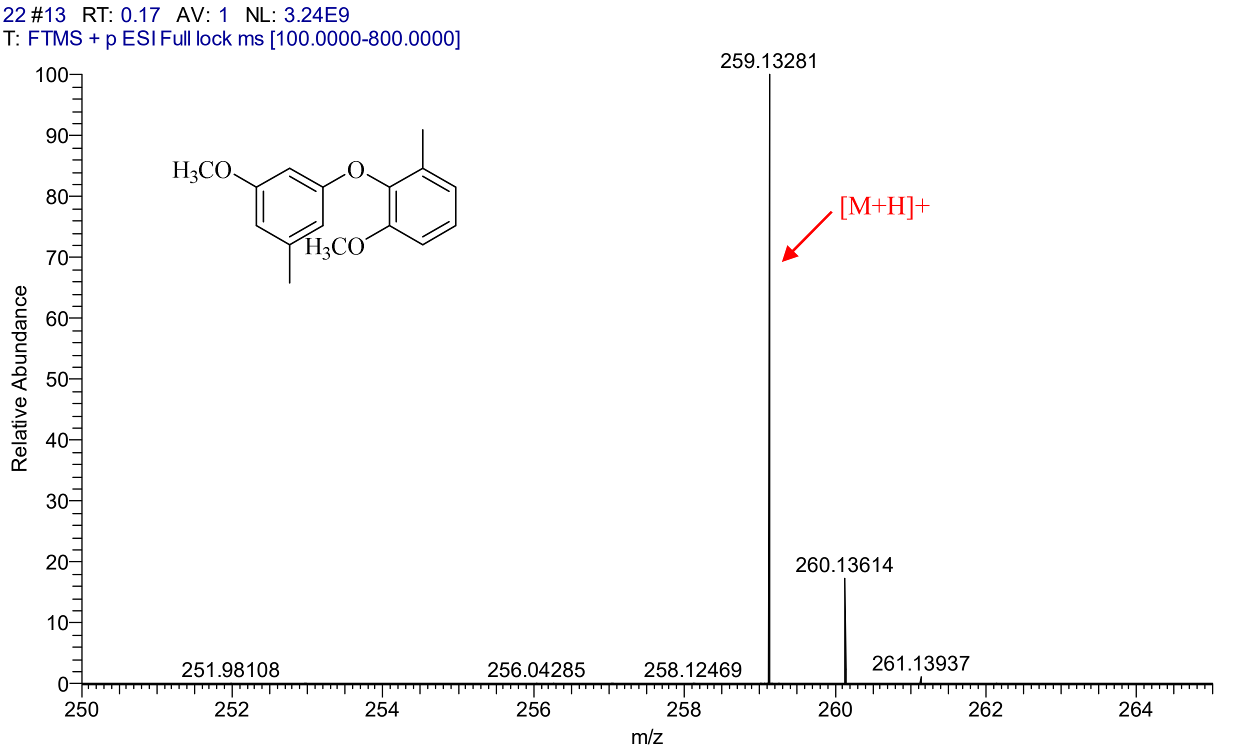

Figure S16.33. (+)-HRESIMS spectrum of **23**.

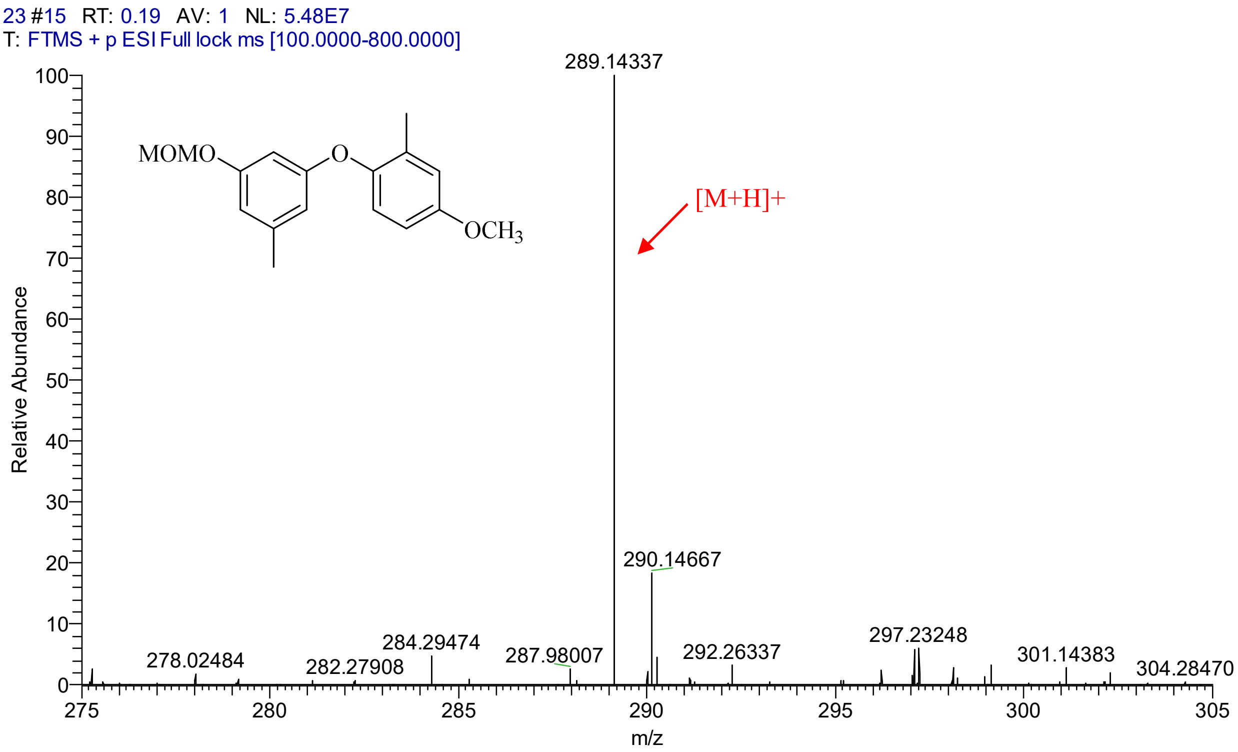

Figure S16.34. (+)-HRESIMS spectrum of **24**.

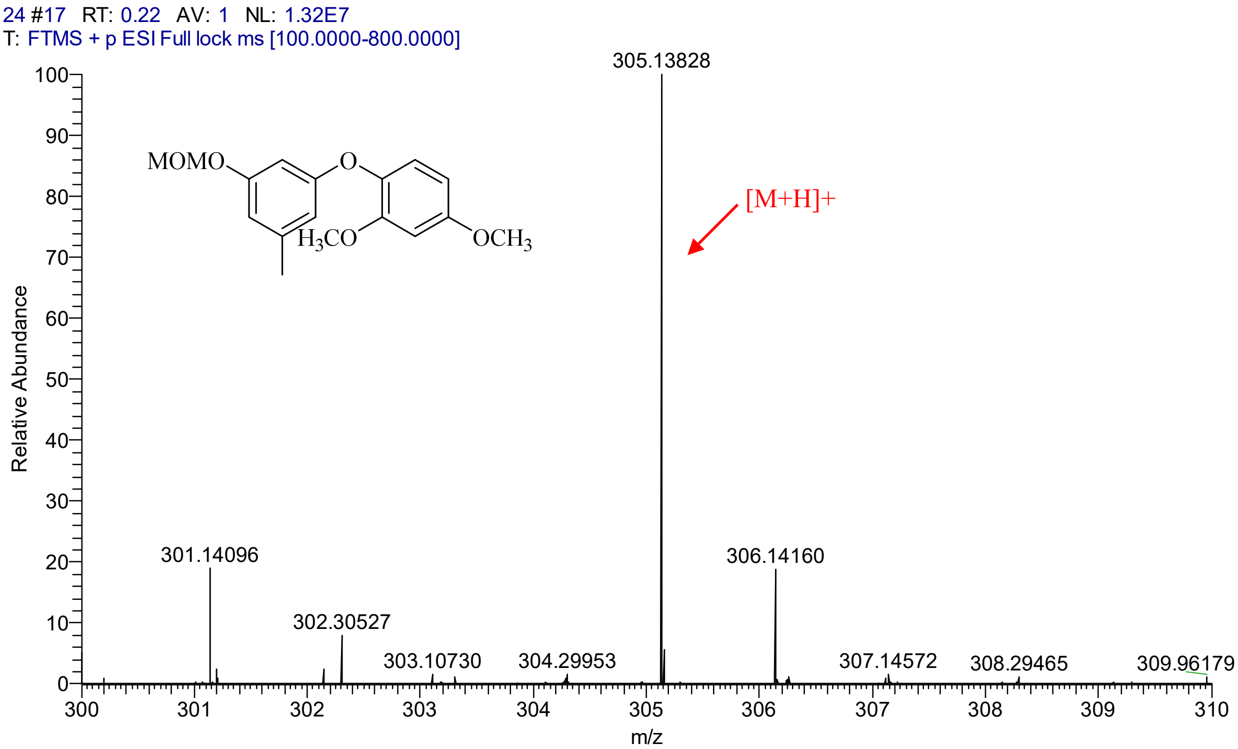

Figure S16.35. (+)-HRESIMS spectrum of **25**.

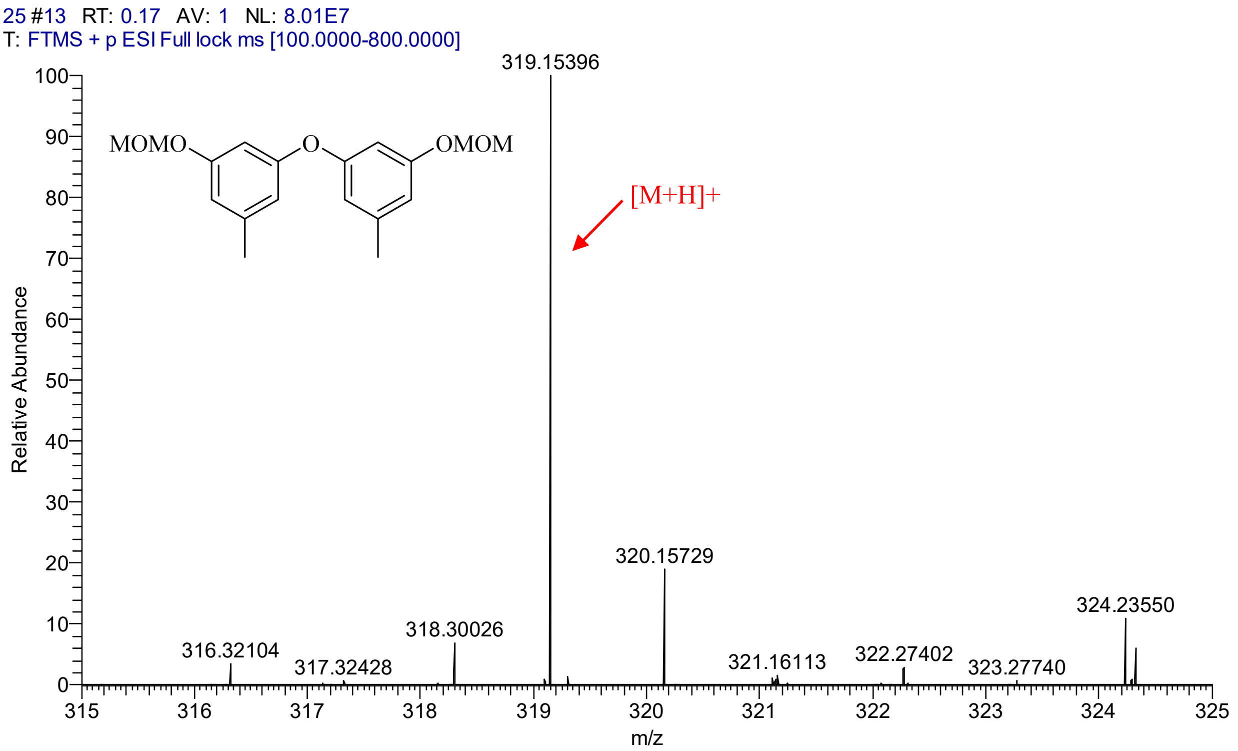

Figure S16.36. (-)-HRESIMS spectrum of **26**.

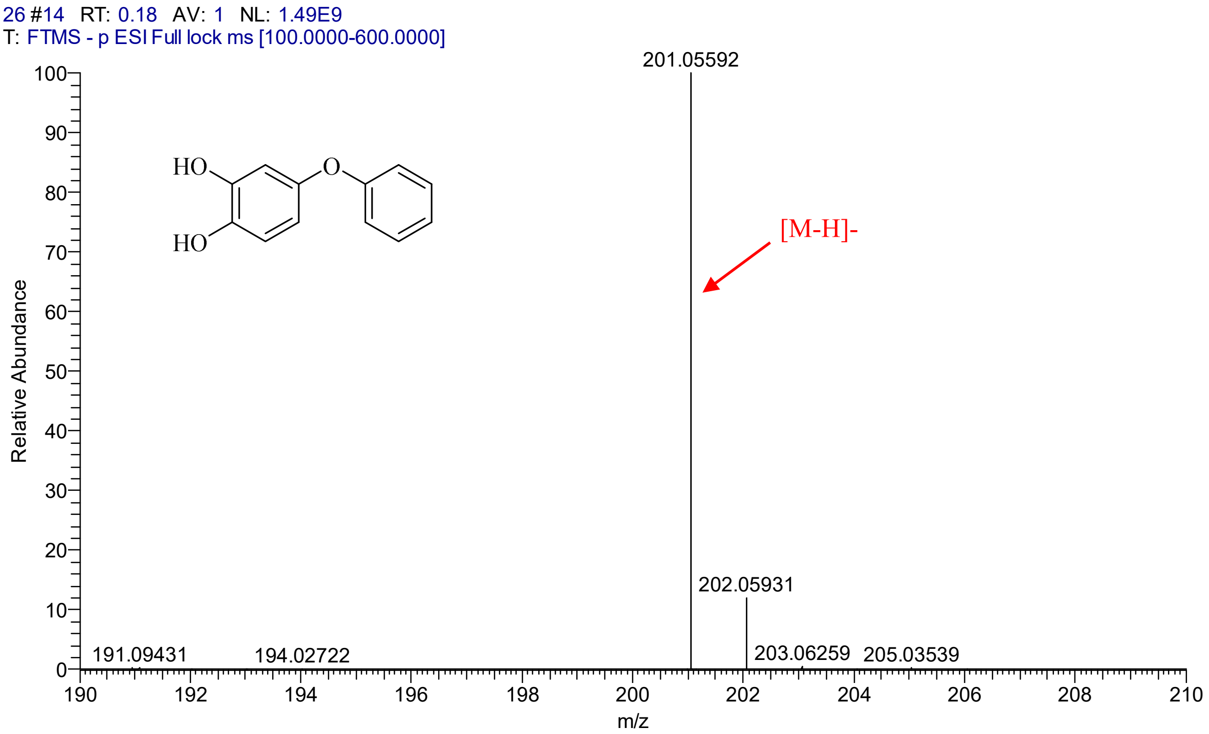

Figure S16.37. (+)-HRESIMS spectrum of **27**.

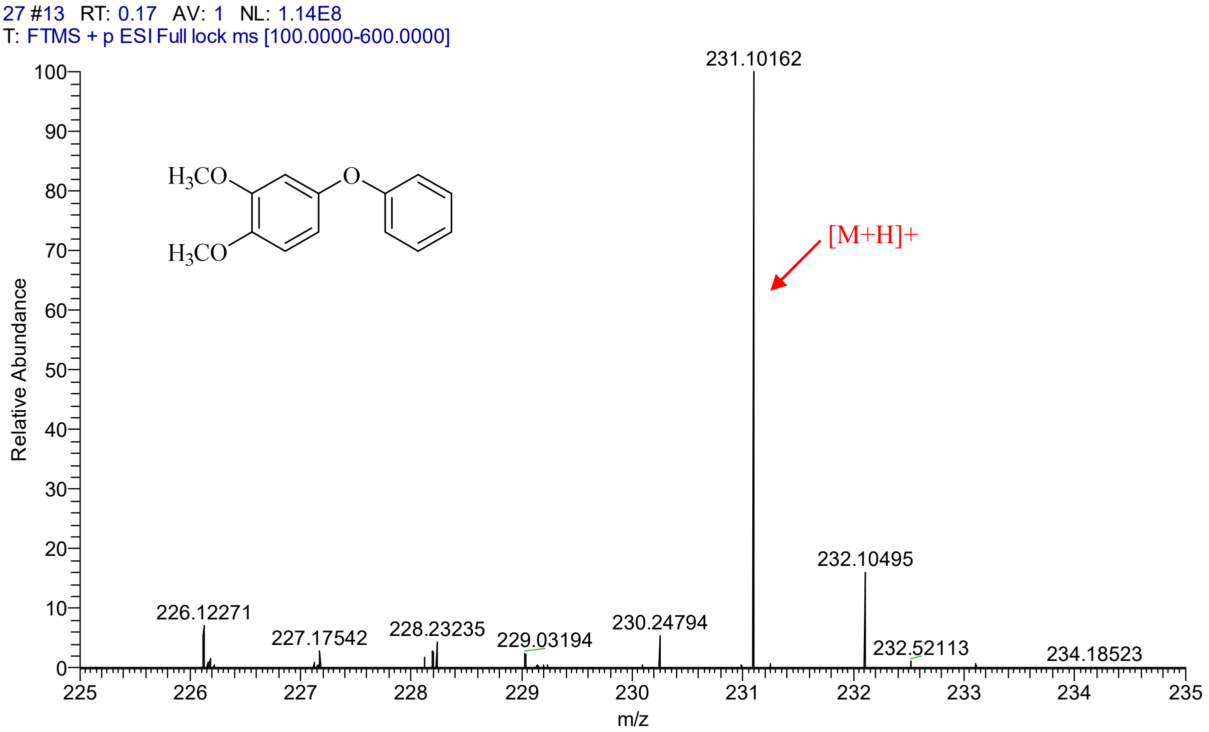

Figure S17. NMR spectra of the DEPs and DPEs.

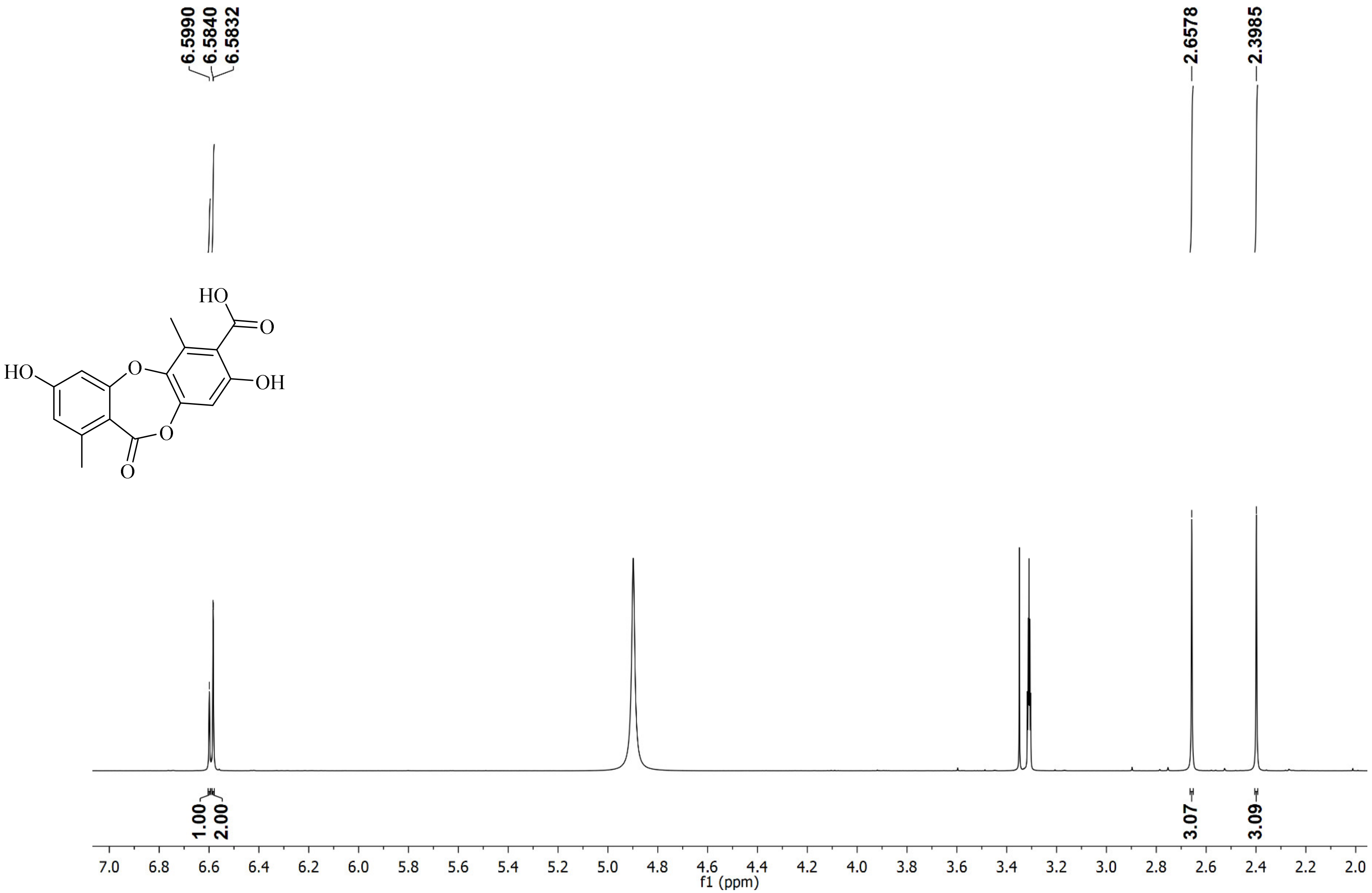

Figure S17.1. ^{1}H NMR spectrum of **2** in methanol-d_4.

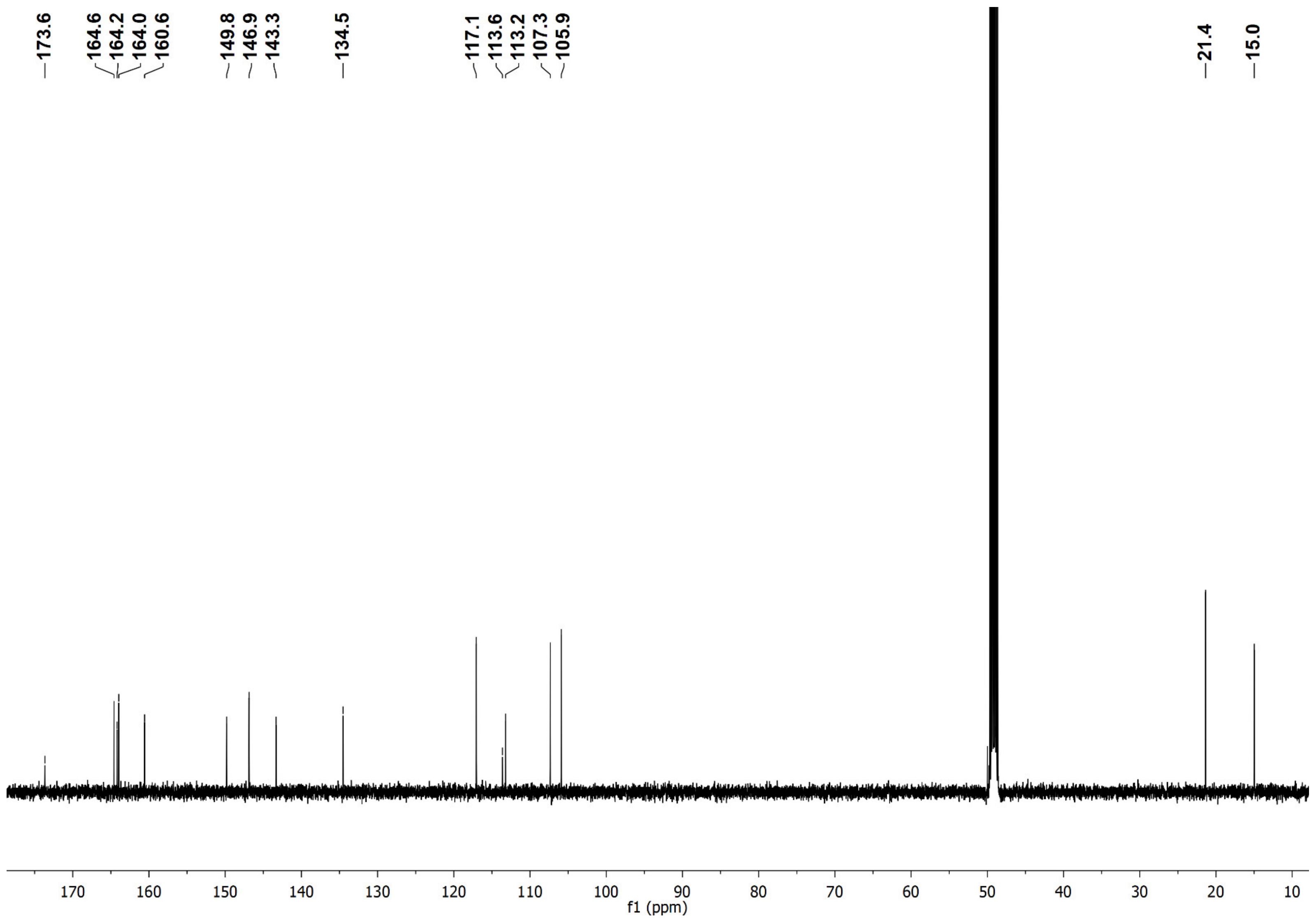

Figure S17.2. ^{13}C NMR spectrum of **2** in methanol-d_4.

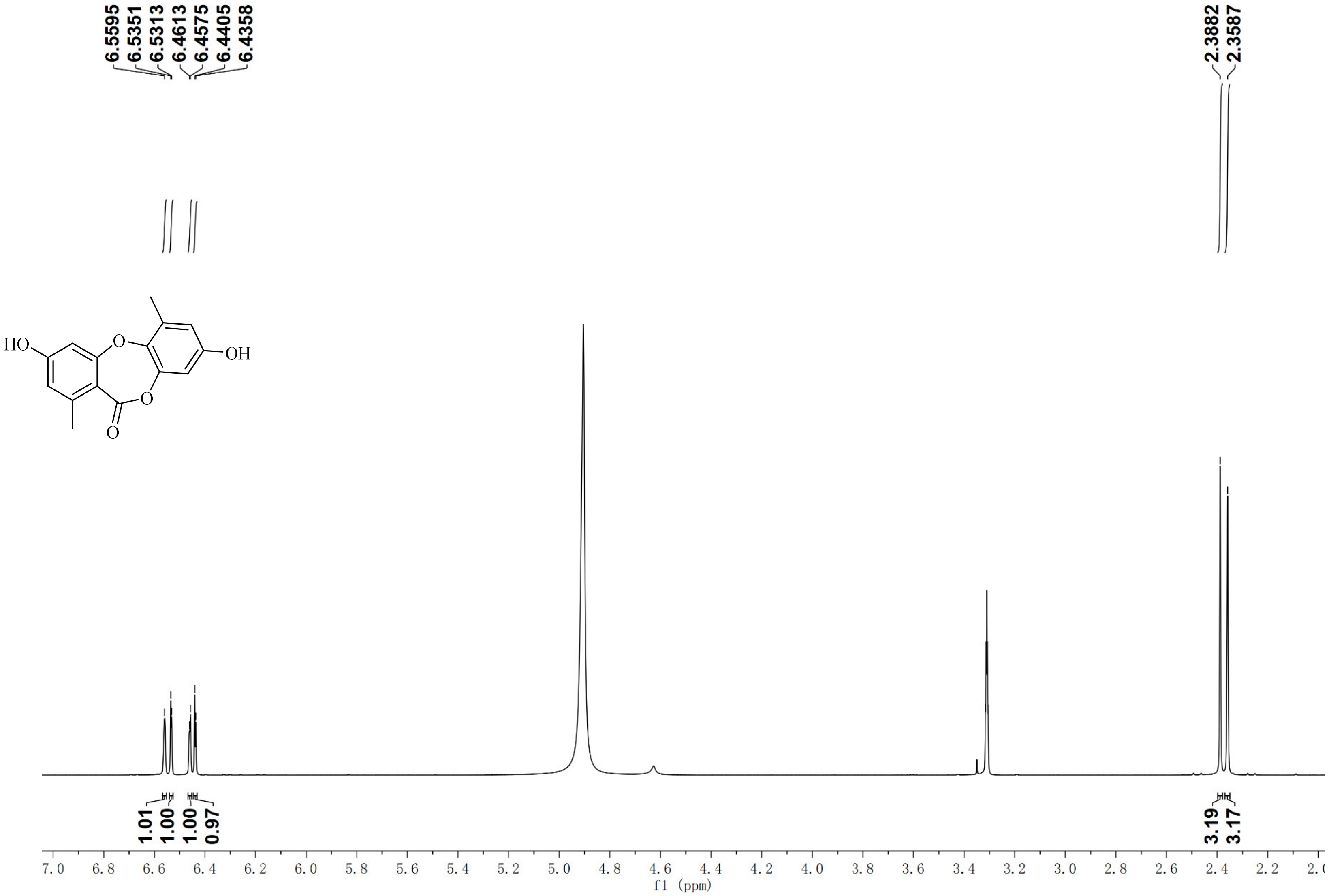

Figure S17.3. ^{1}H NMR spectrum of **3** in methanol-d_4.

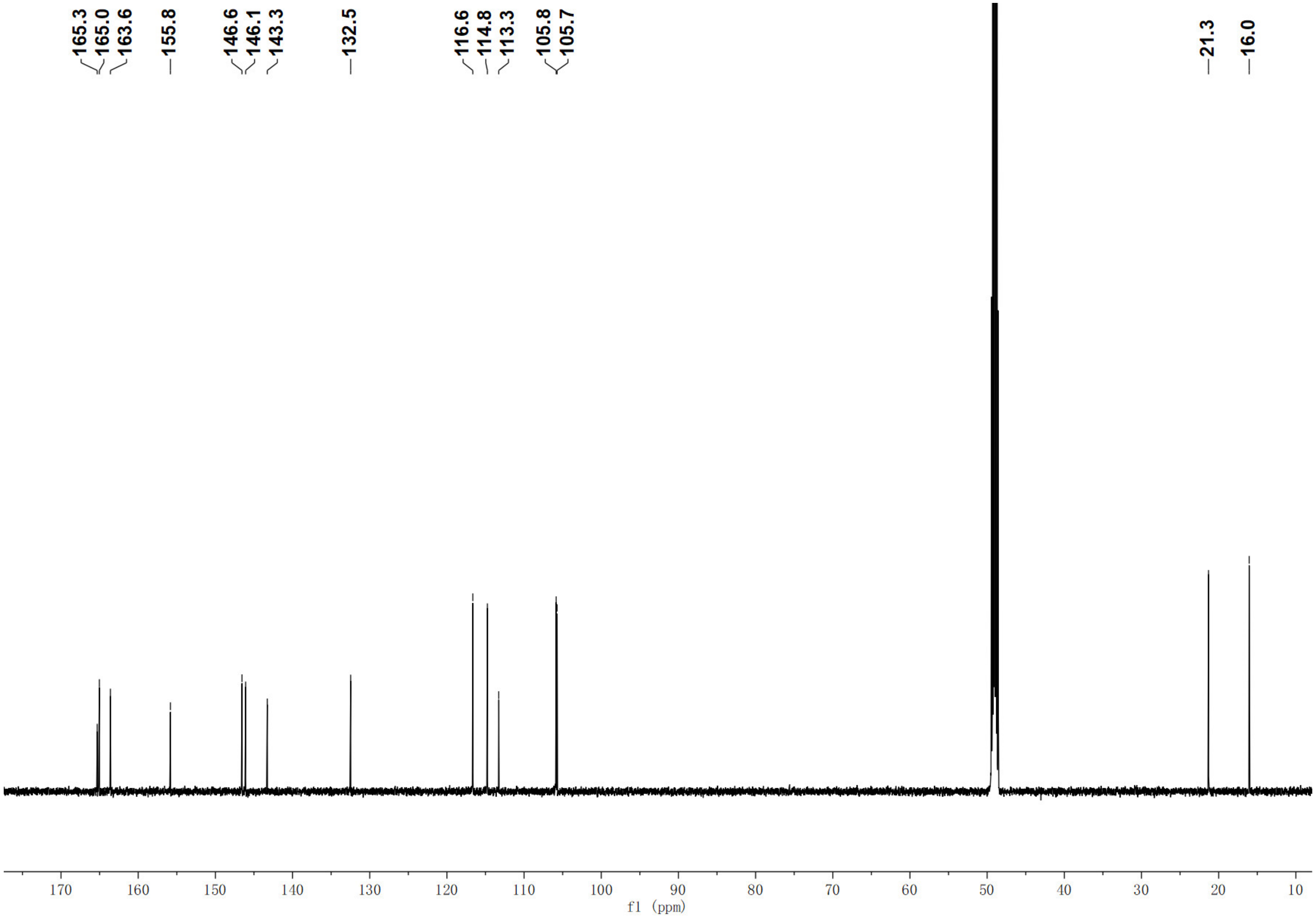

Figure S17.4. ^{13}C NMR spectrum of **3** in methanol-d_4.

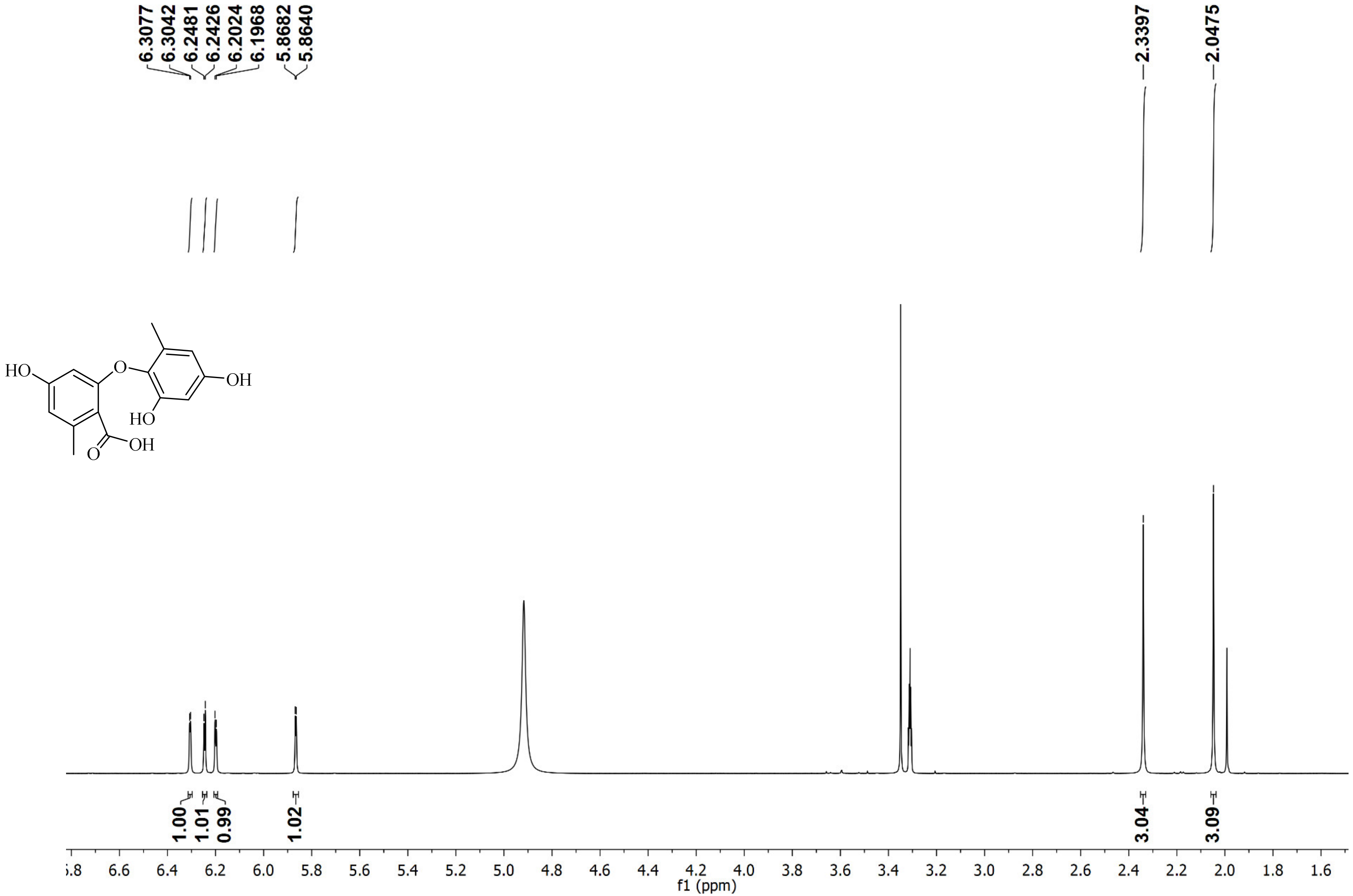

Figure S17.5. ^{1}H NMR spectrum of **4** in methanol-d_4.

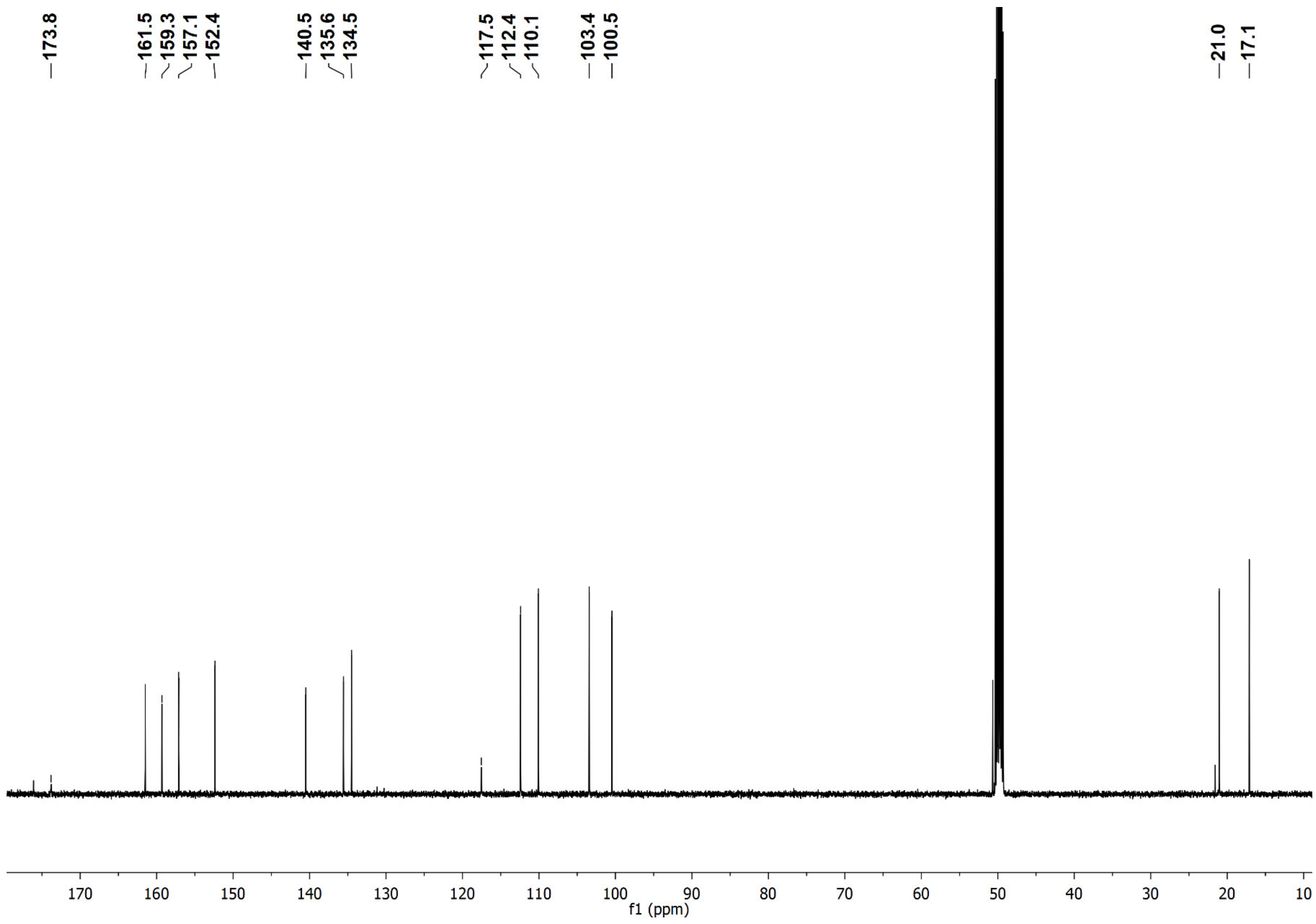

Figure S17.6. ^{13}C NMR spectrum of **4** in methanol-d_4.

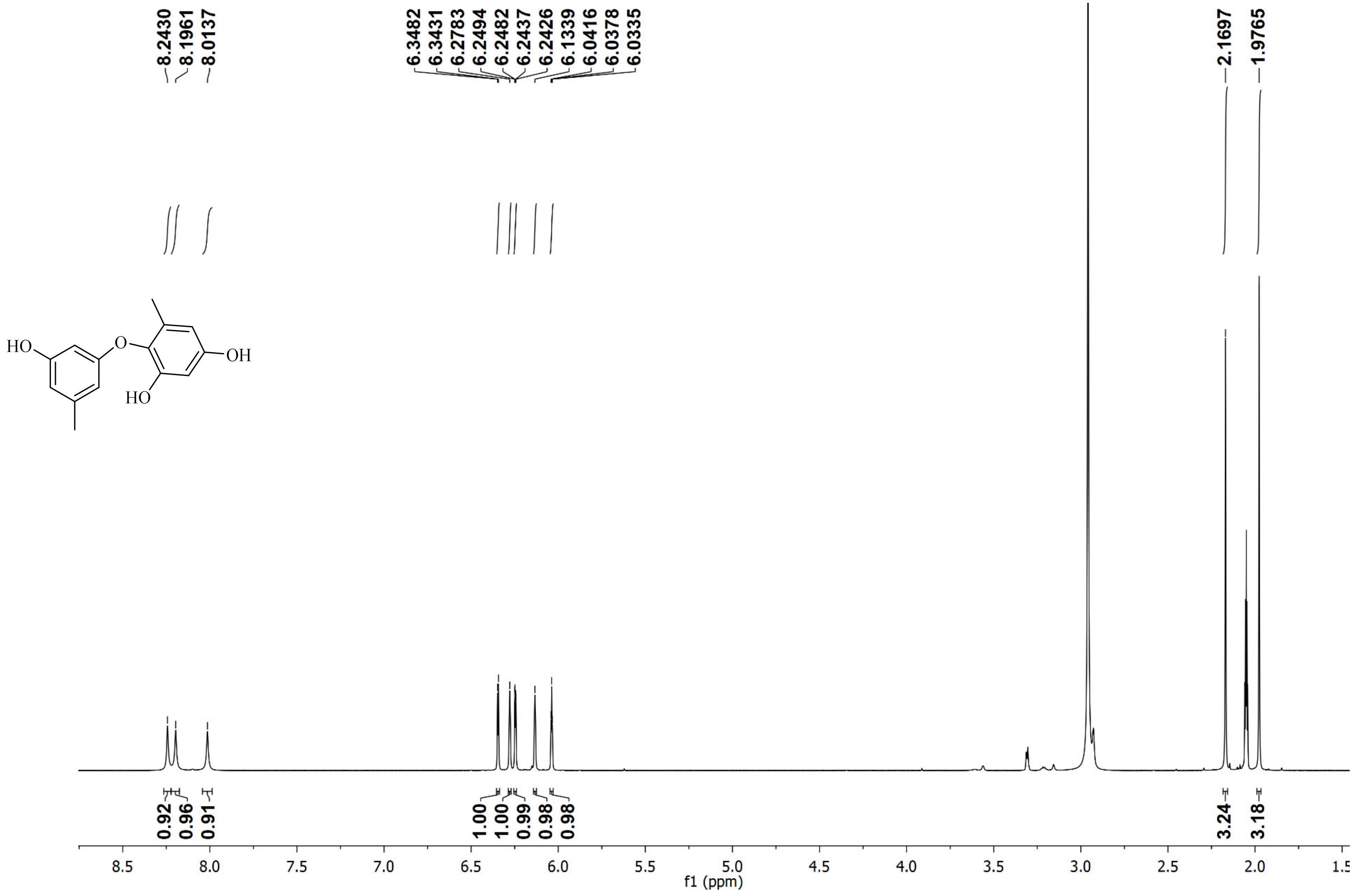

Figure S17.7. ^{1}H NMR spectrum of **5** in acetone-d_6.

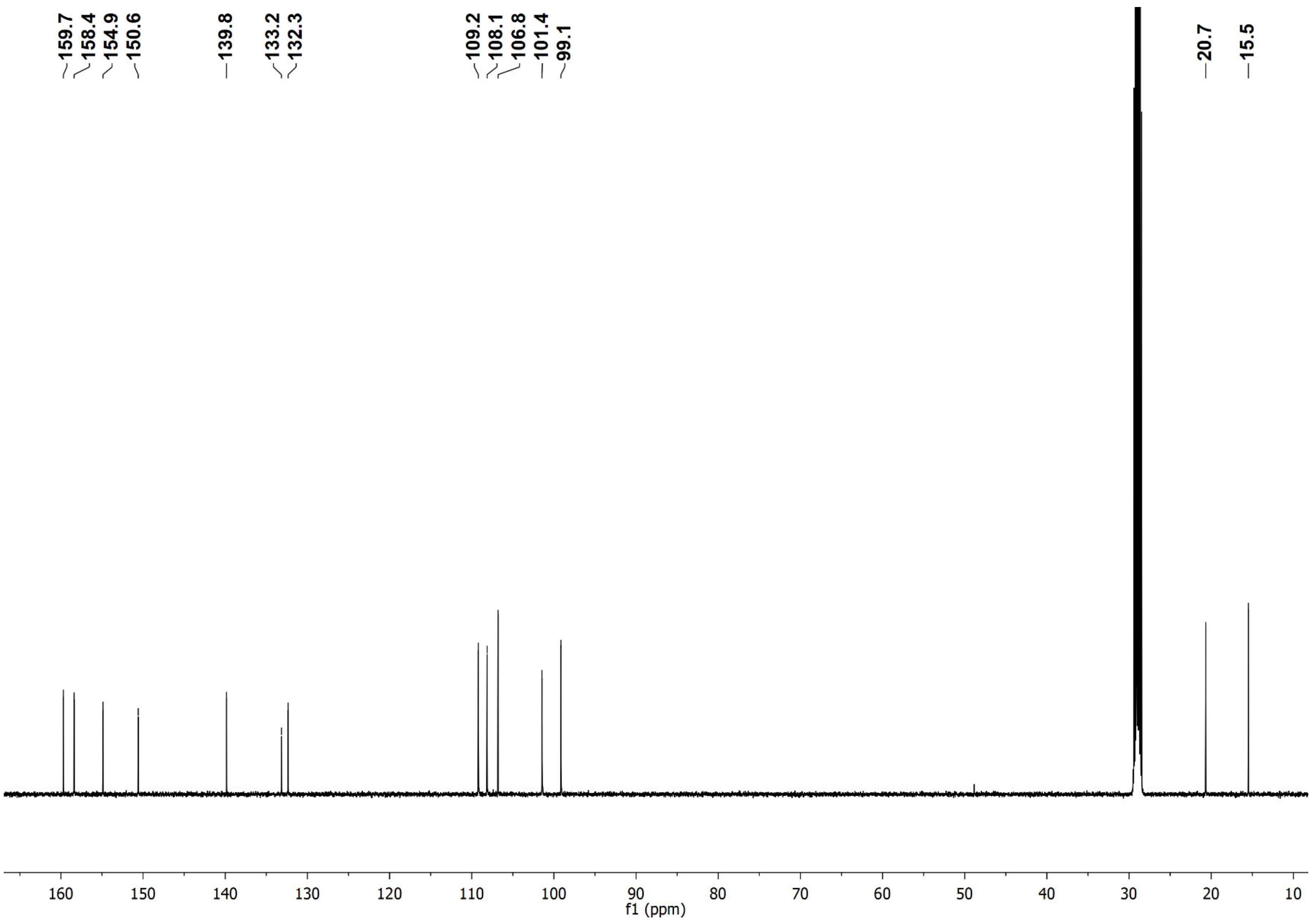

Figure S17.8. ^{13}C NMR spectrum of **5** in acetone-d_6.

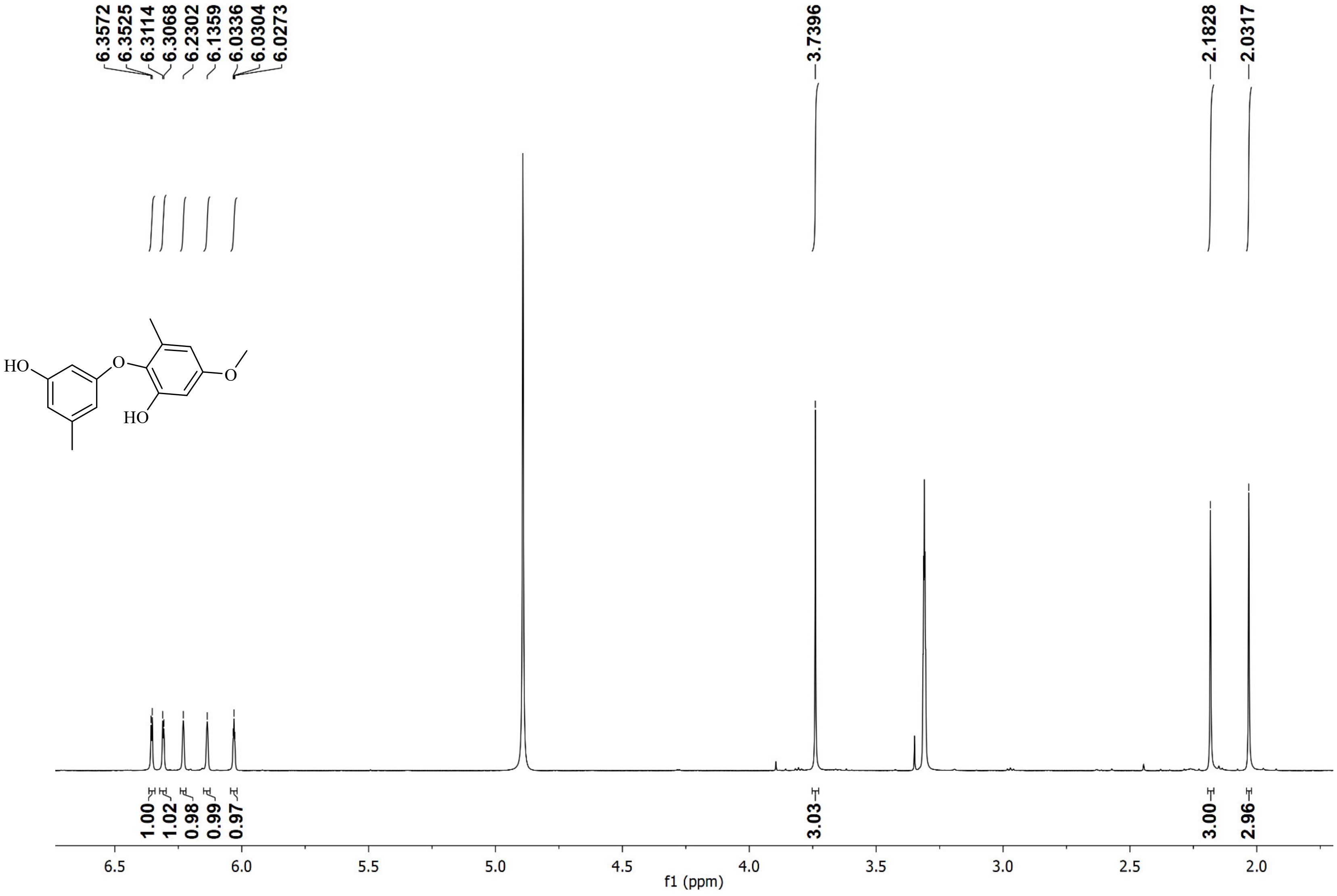

Figure S17.9. ^{1}H NMR spectrum of **6** in methanol-d_4.

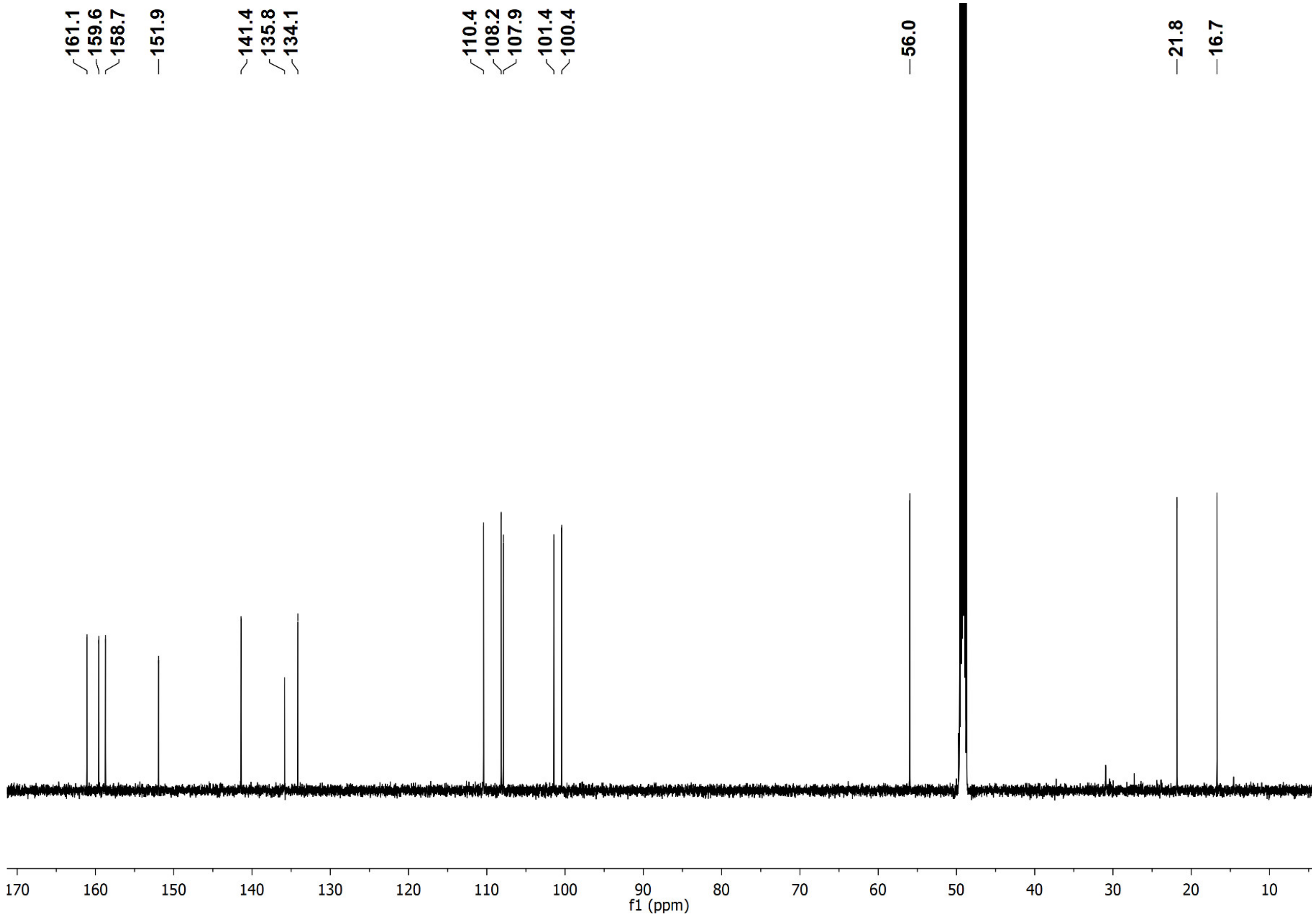

Figure S17.10. ^{13}C NMR spectrum of **6** in methanol-d_4.

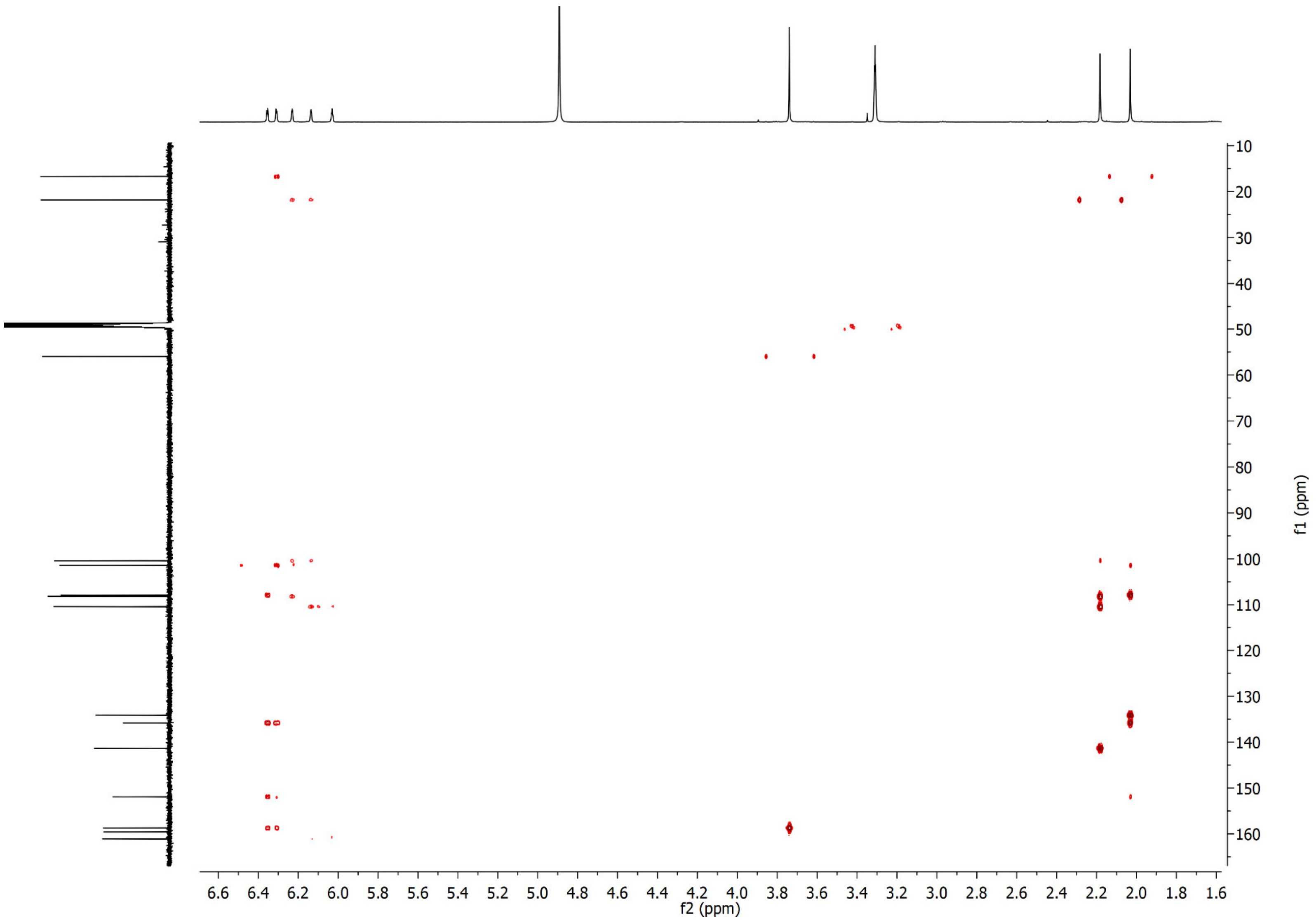

Figure S17.11. HMBC spectrum of **6** in methanol-d_4.

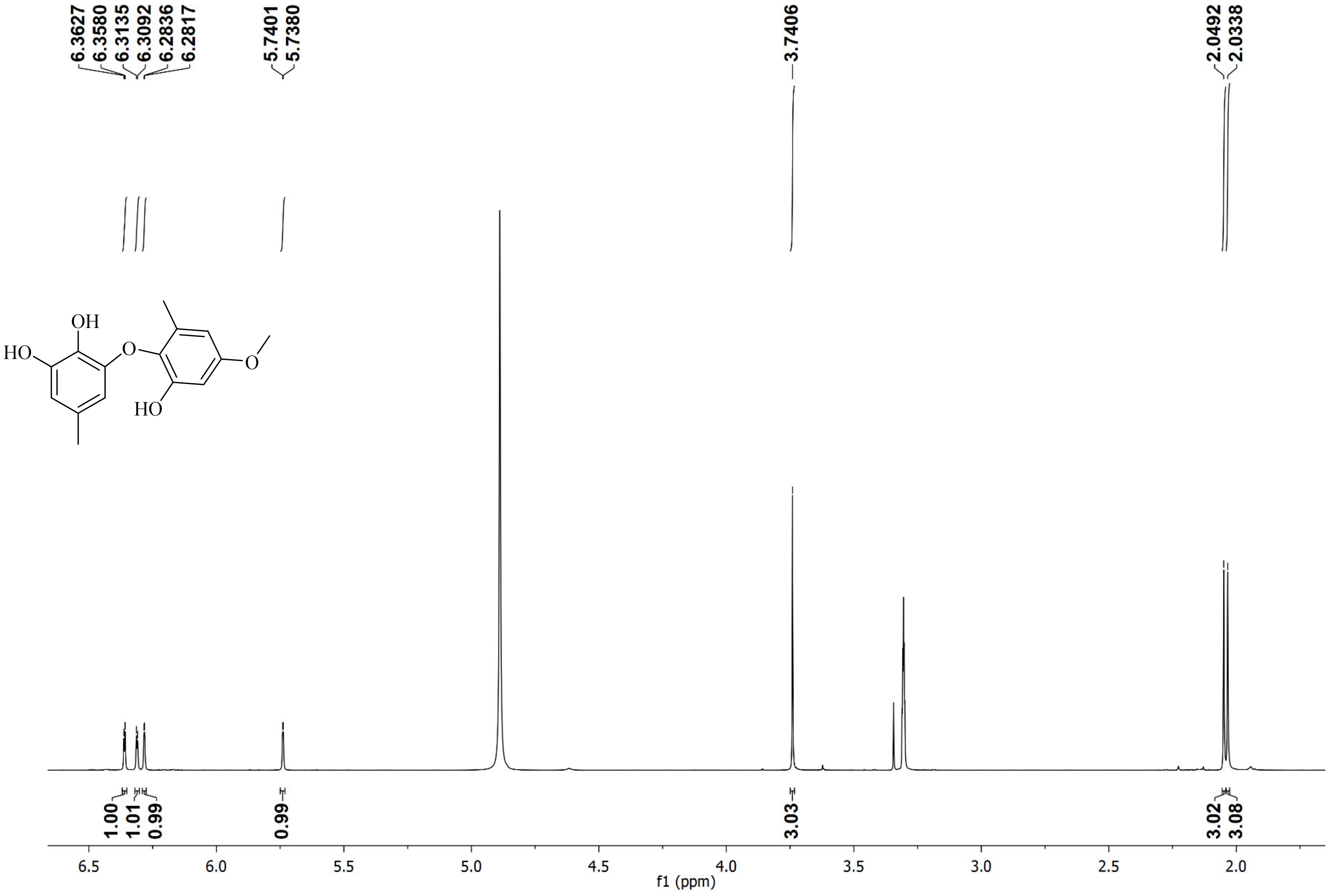

Figure S17.12. ^{1}H NMR spectrum of **7** in methanol-d_4.

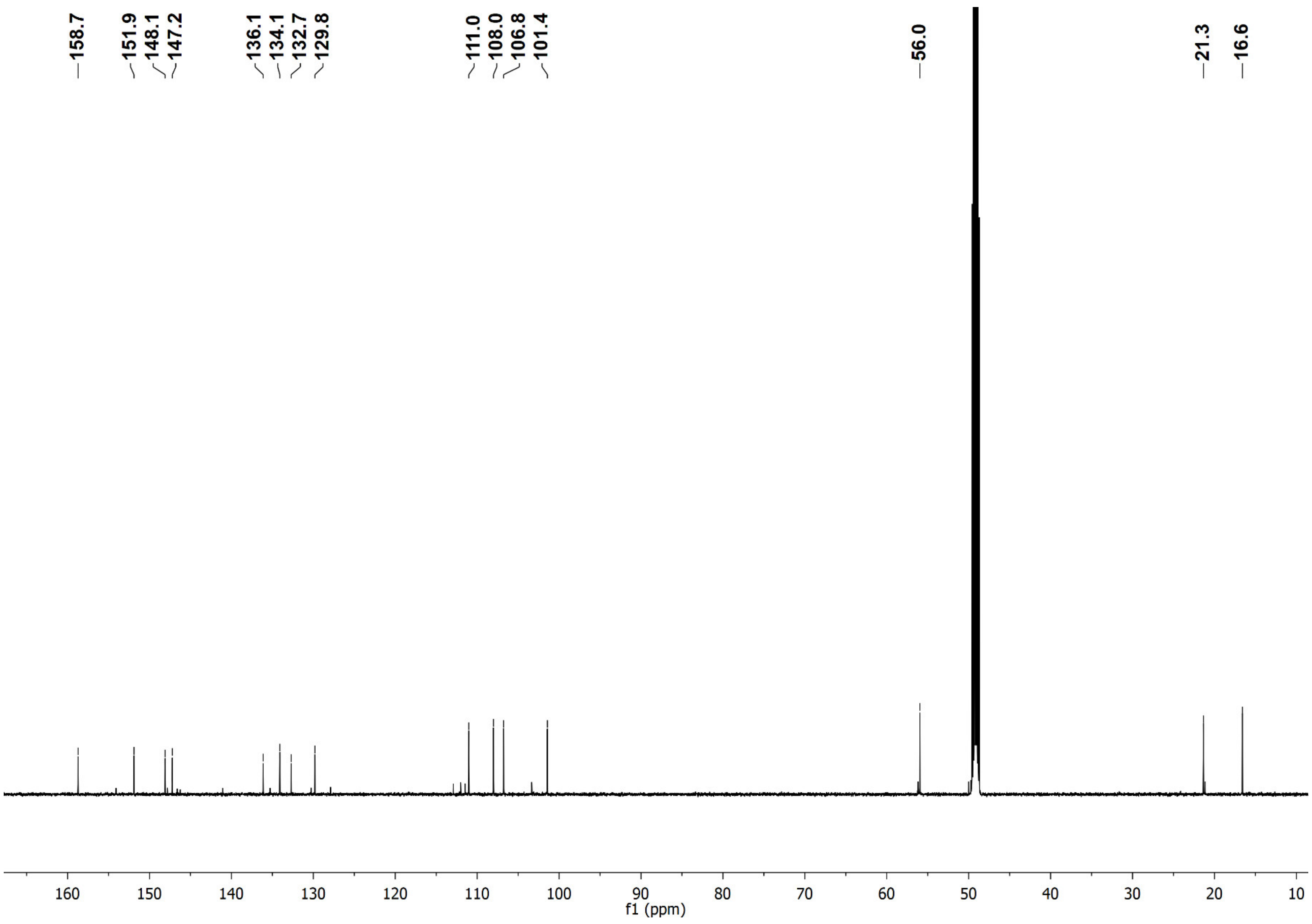

Figure S17.13. ^{13}C NMR spectrum of **7** in methanol-d_4.

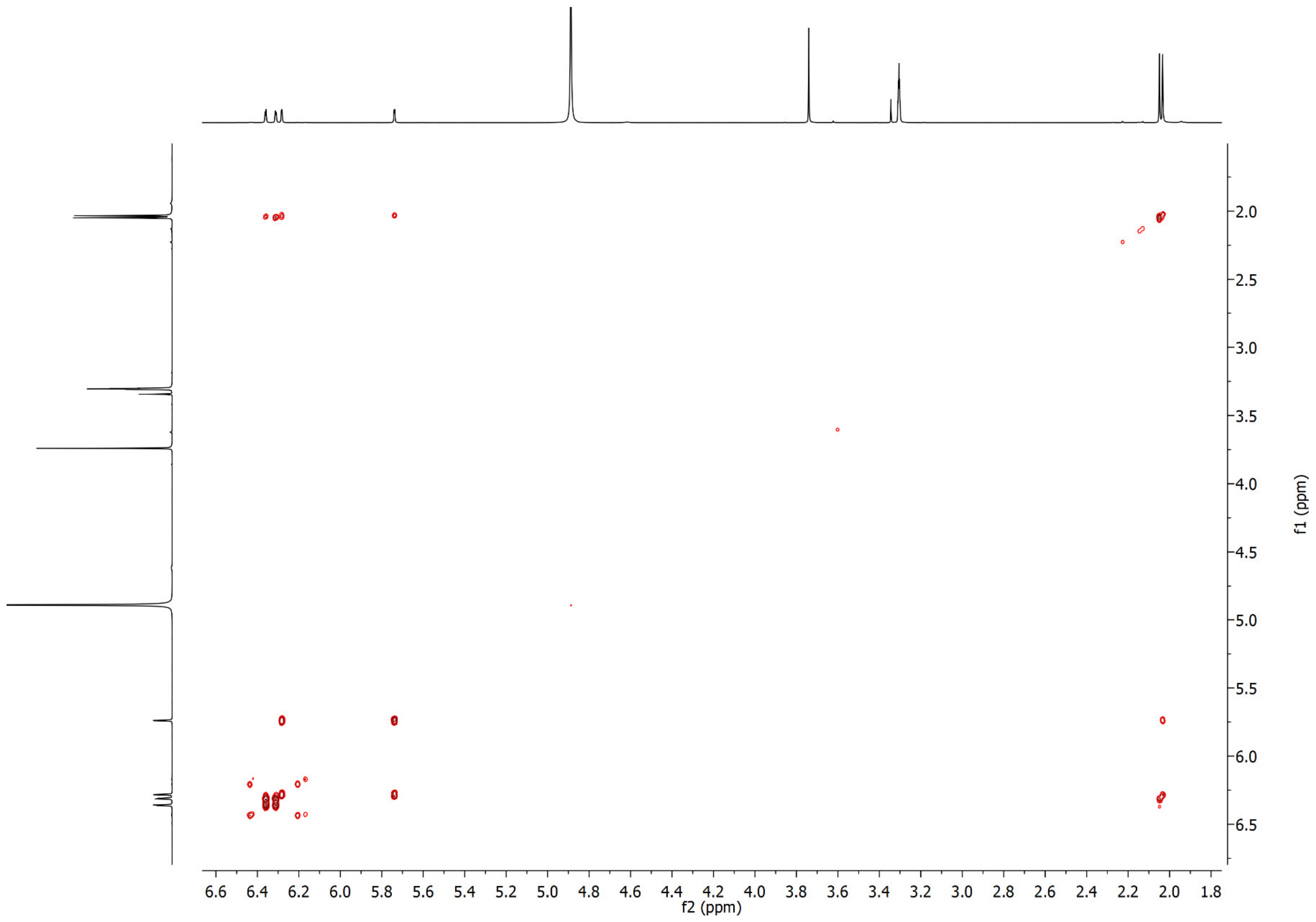

Figure S17.14. ¹H-¹H COSY spectrum of **7** in methanol-d_4.

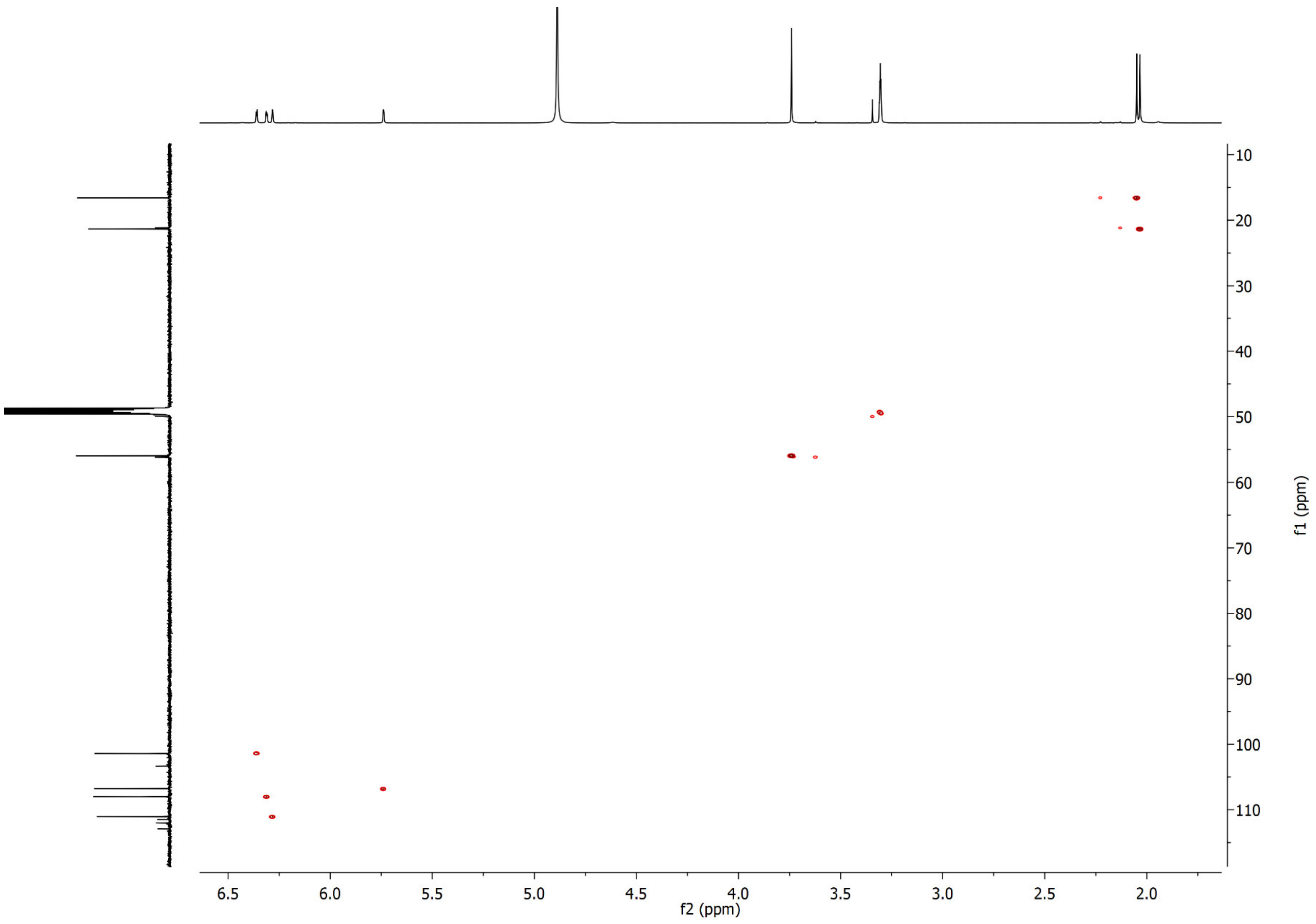

Figure S17.15. HSQC spectrum of **7** in methanol-d_4.

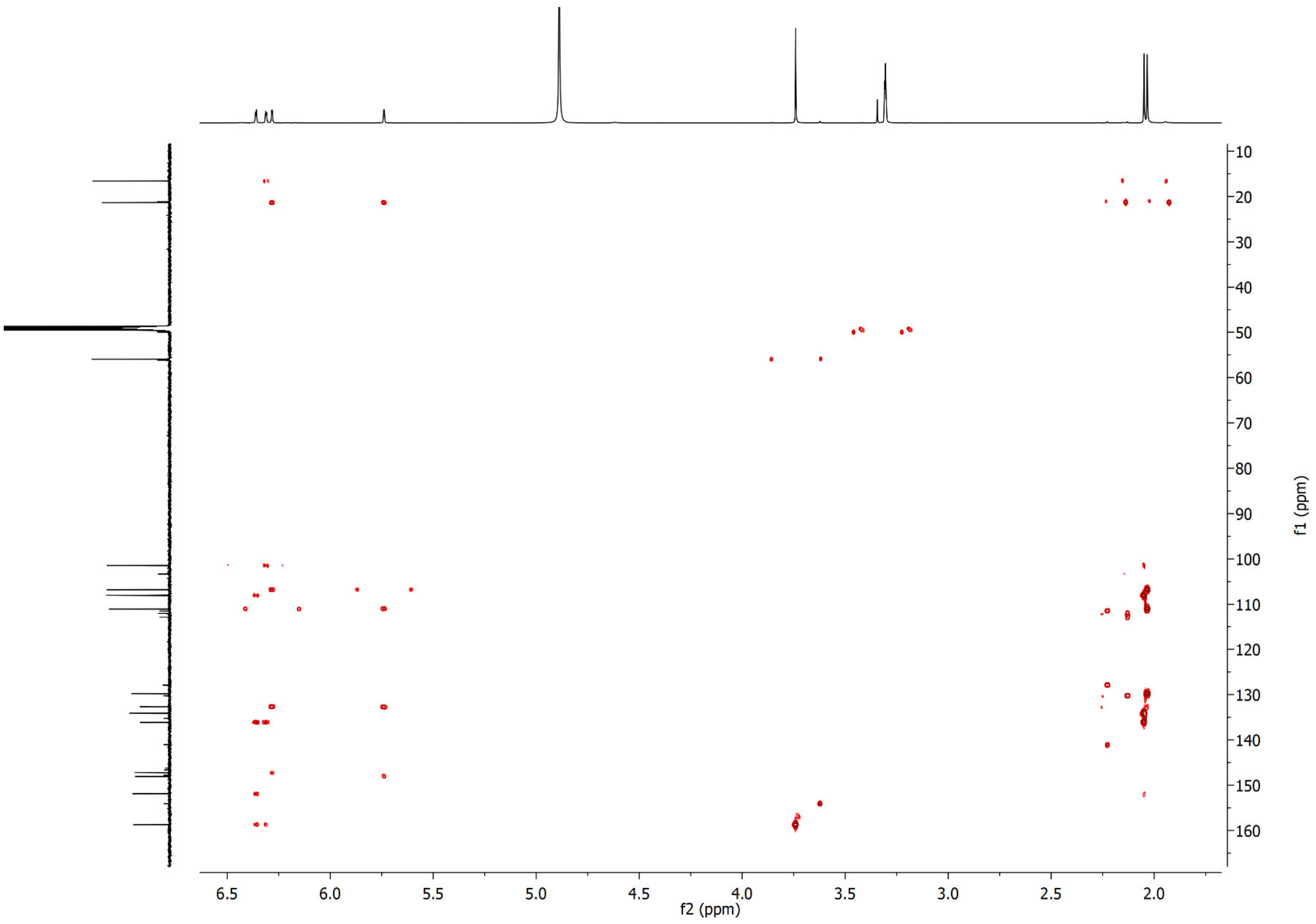

Figure S17.16. HMBC spectrum of **7** in methanol-d_4.

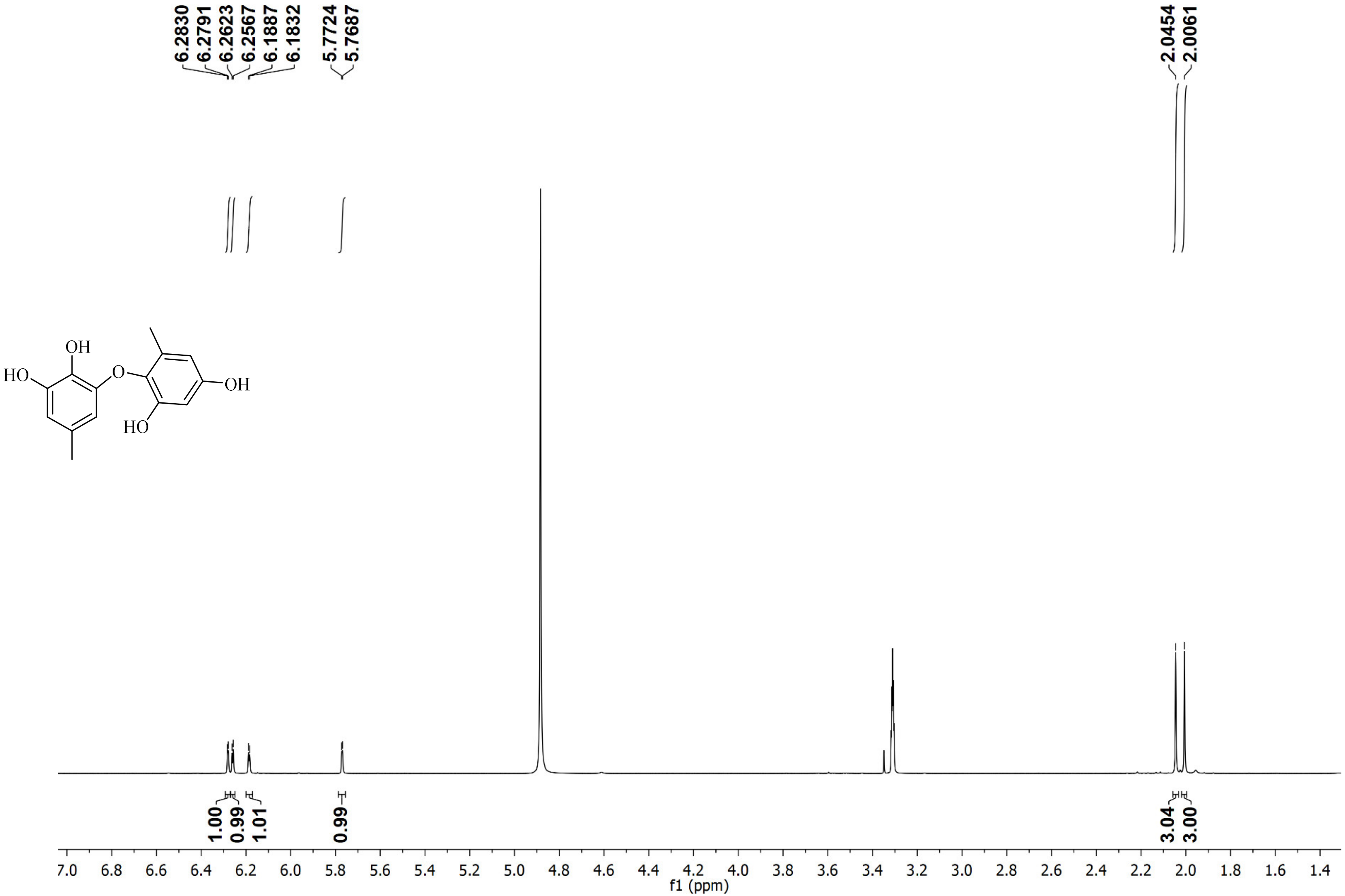

Figure S17.17. ^{1}H NMR spectrum of **8** in methanol-d_4.

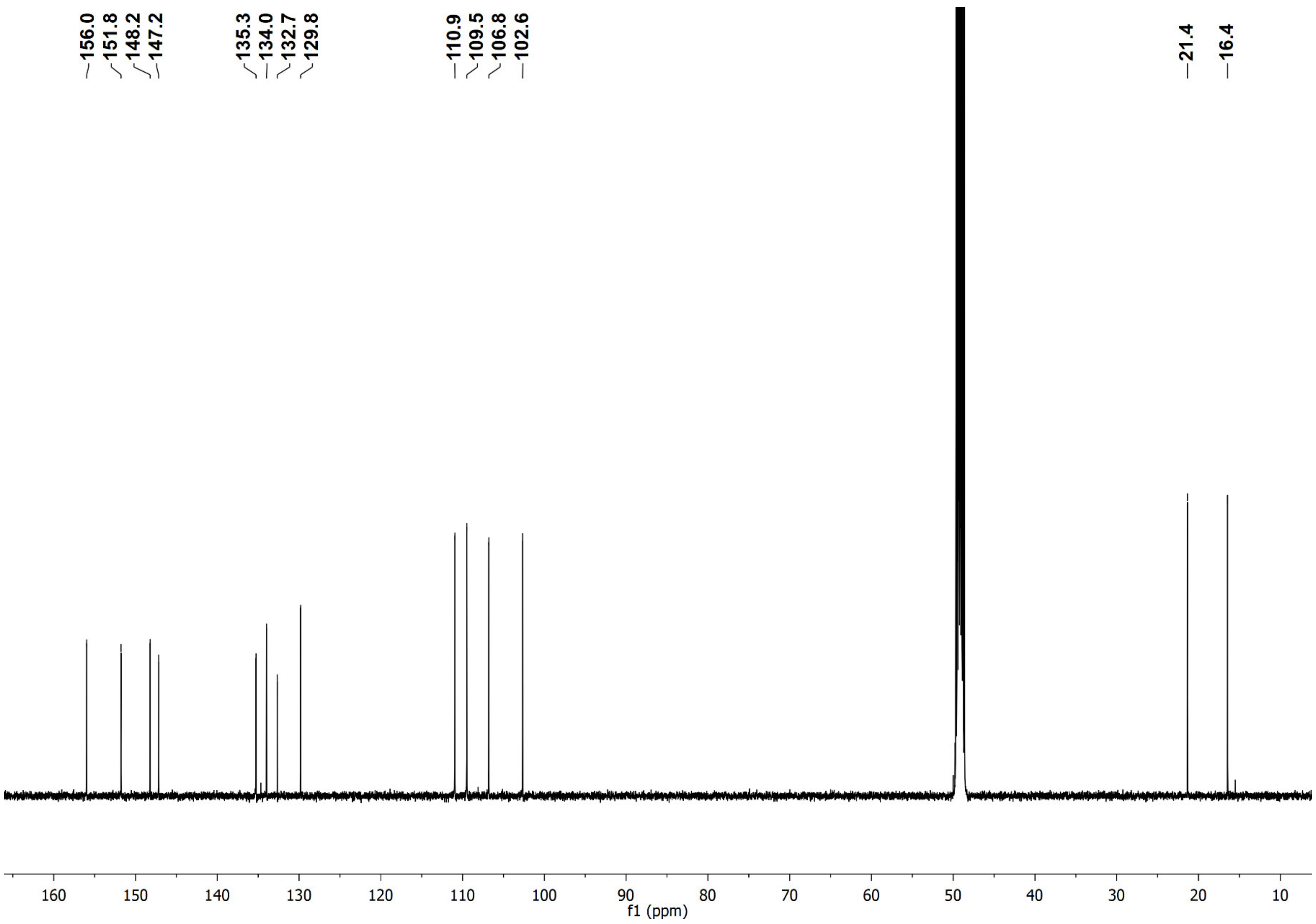

Figure S17.18. ^{13}C NMR spectrum of **8** in methanol-d_4.

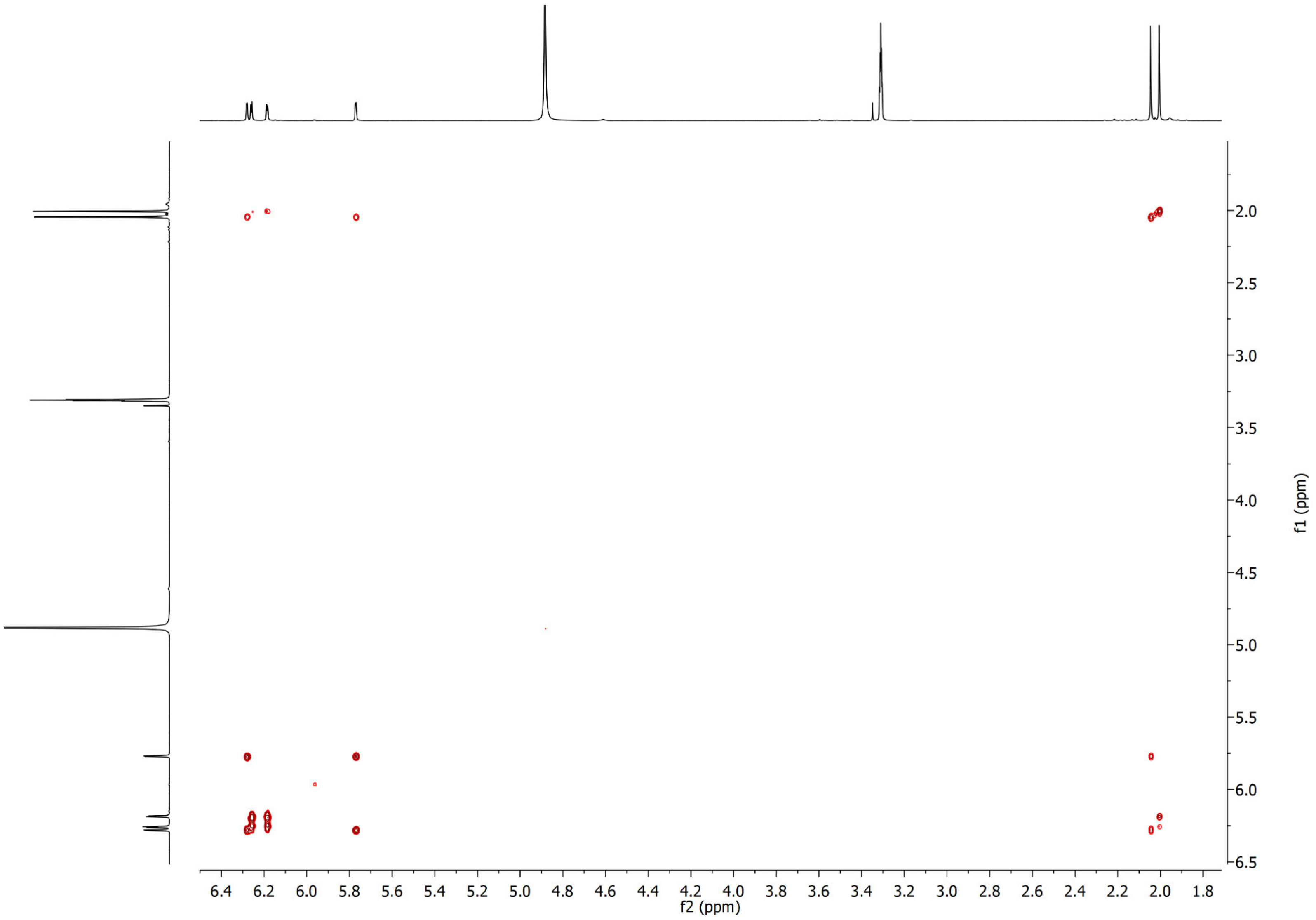

Figure S17.19. ^{1}H-^{1}H COSY spectrum of **8** in methanol-d_4.

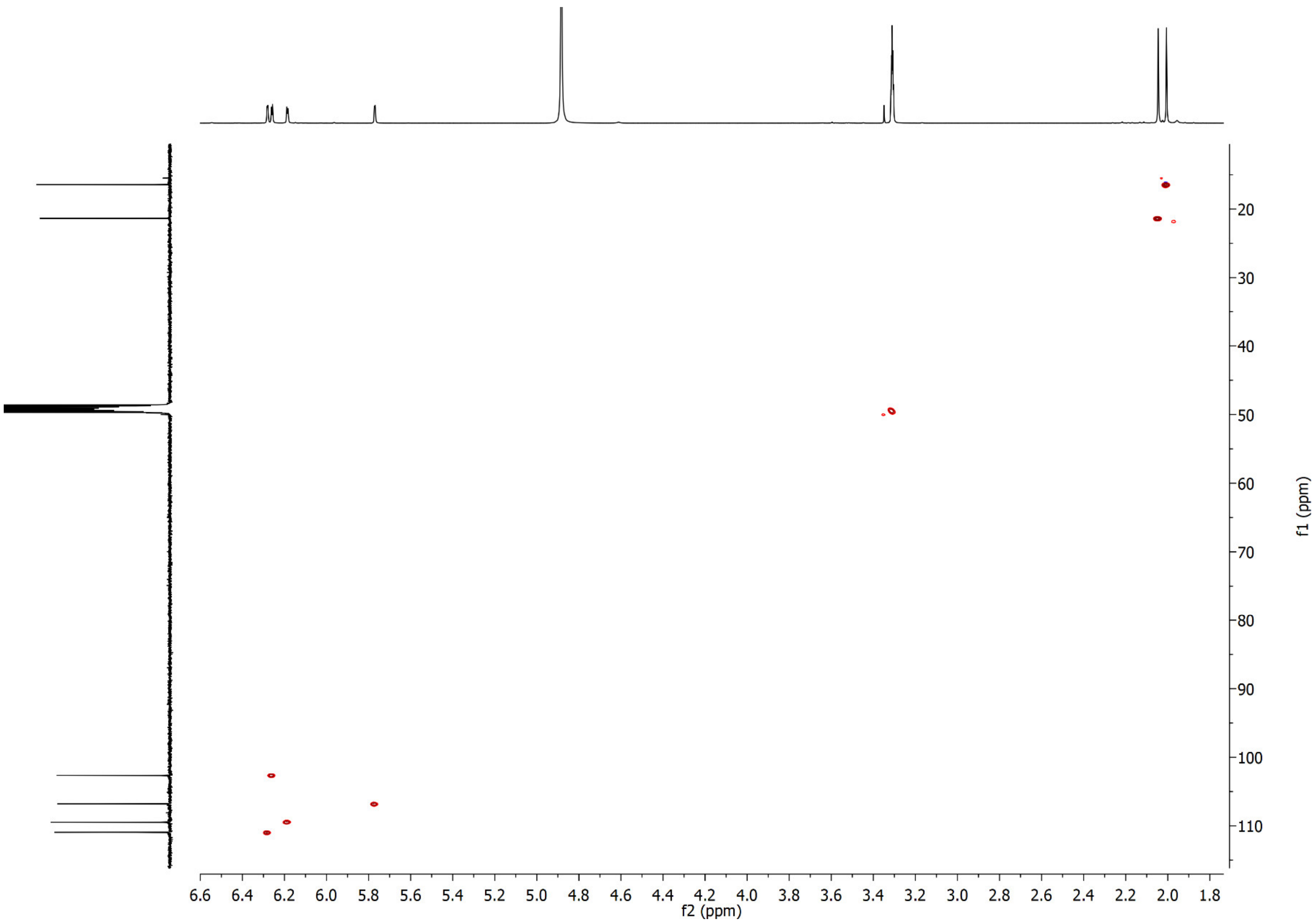

Figure S17.20. HSQC spectrum of **8** in methanol-d_4.

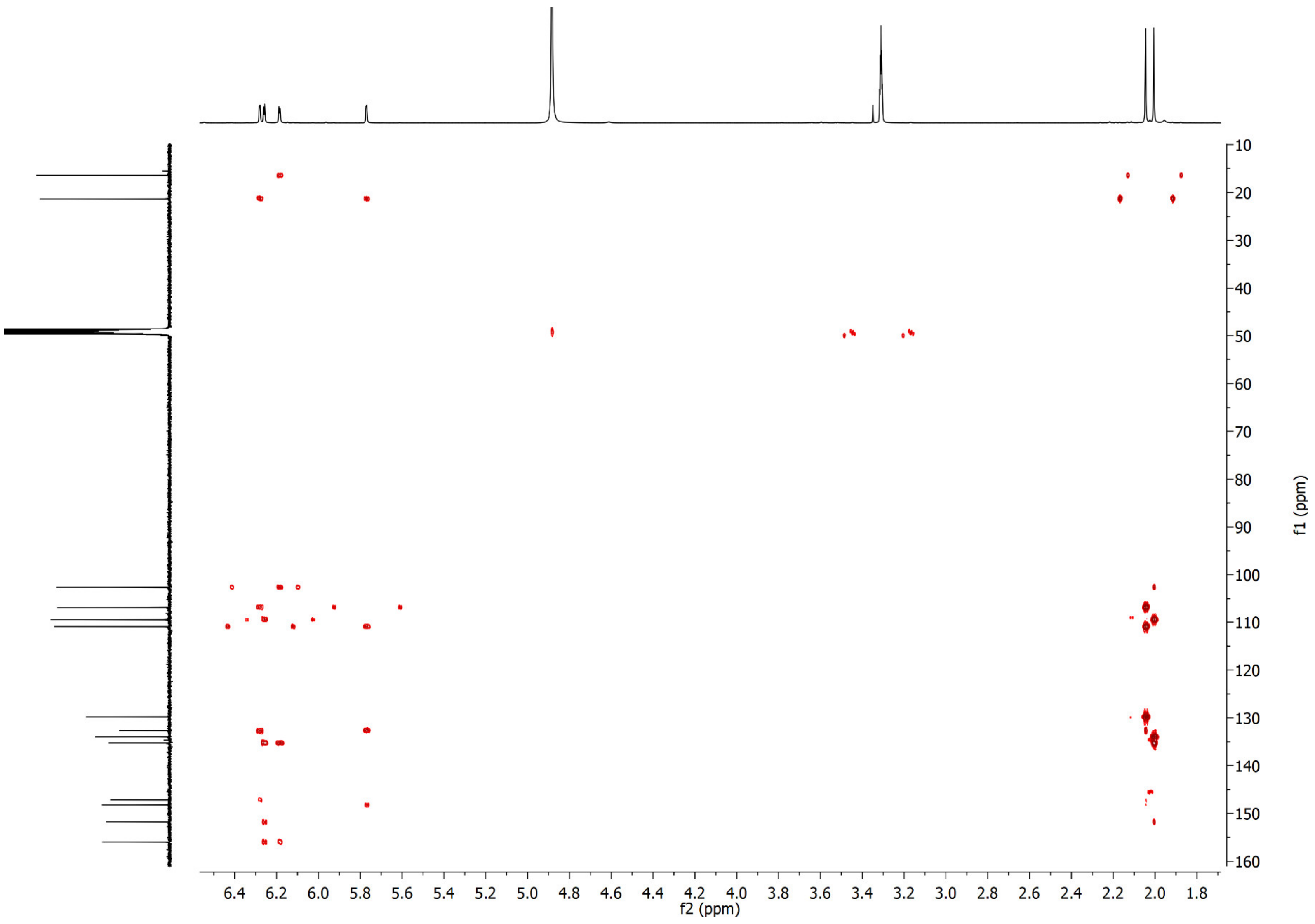

Figure S17.21. HMBC spectrum of **8** in methanol-d_4.

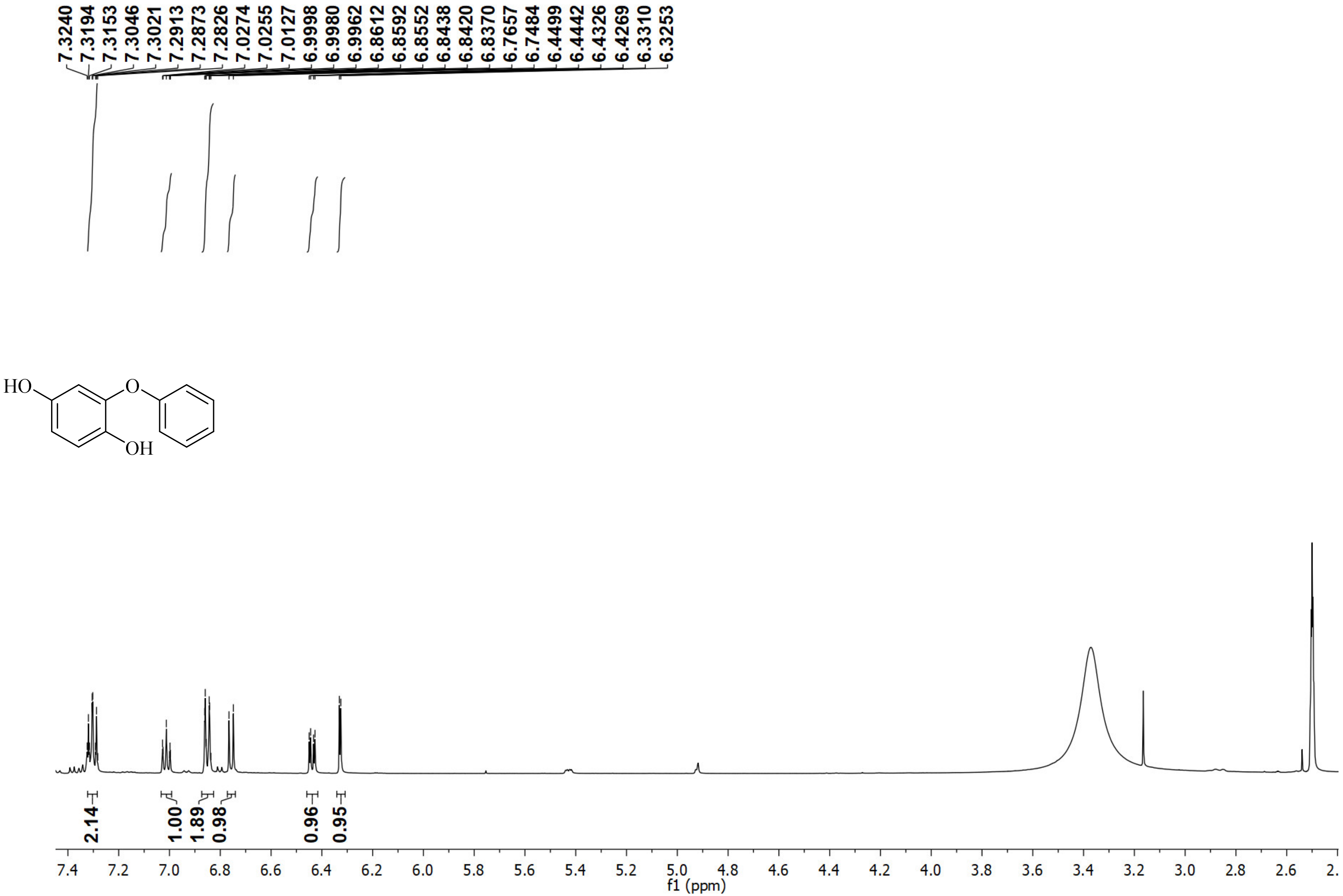

Figure S17.22. ^{1}H NMR spectrum of **9a** in DMSO-d_6.

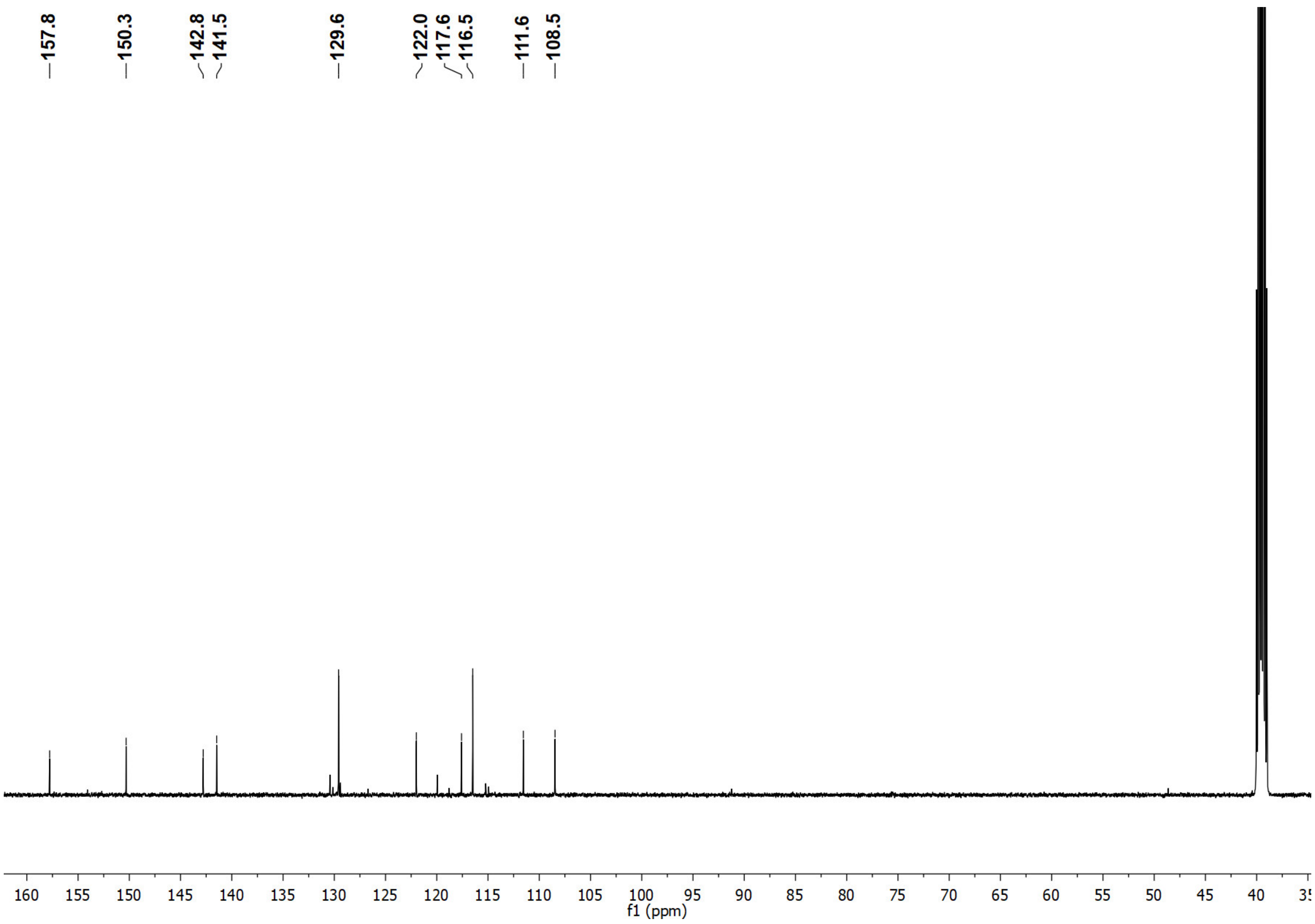

Figure S17.23. ^{13}C NMR spectrum of **9a** in DMSO-d_6.

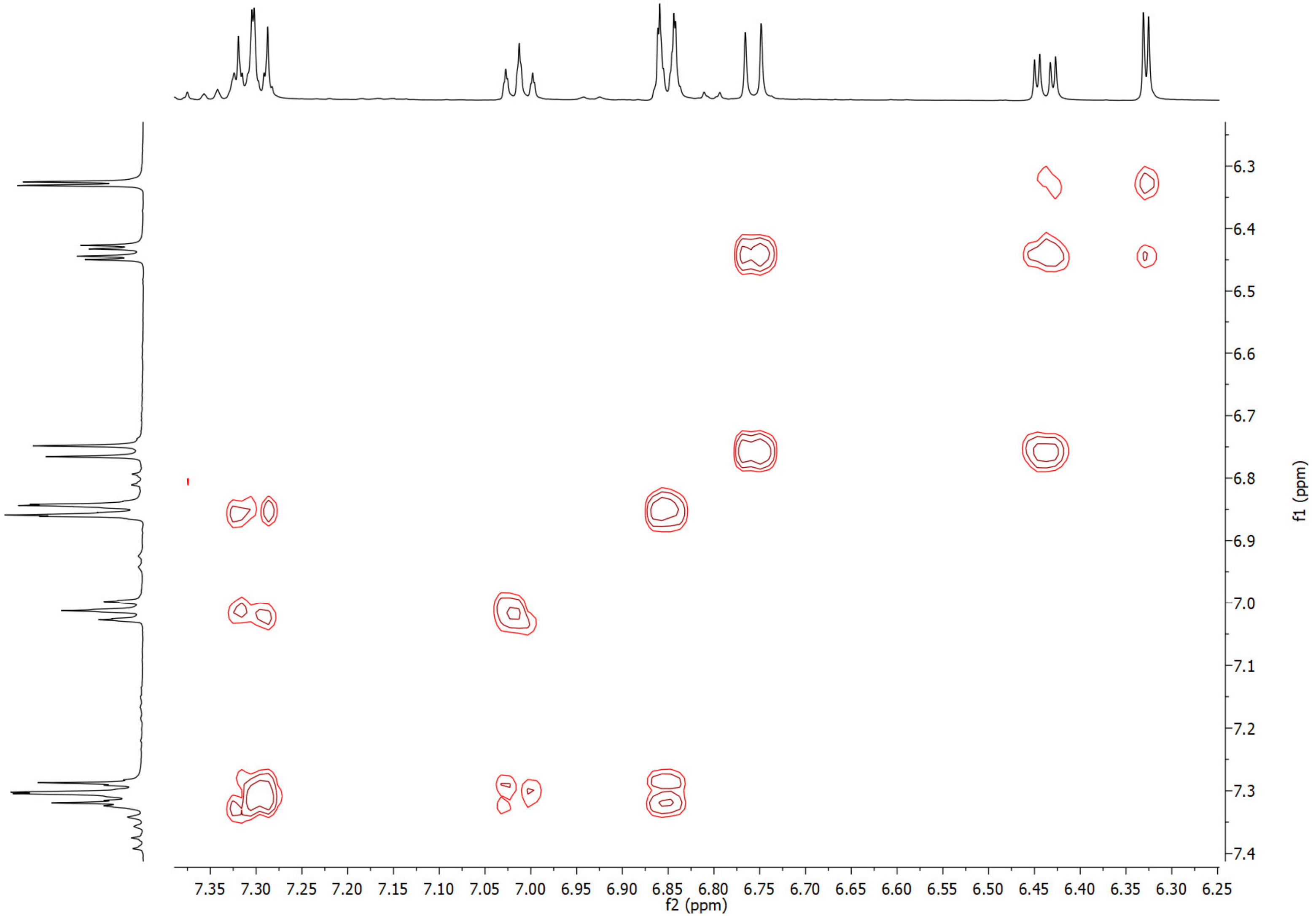

Figure S17.24. ^{1}H-^{1}H COSY spectrum of **9a** in DMSO-d_6.

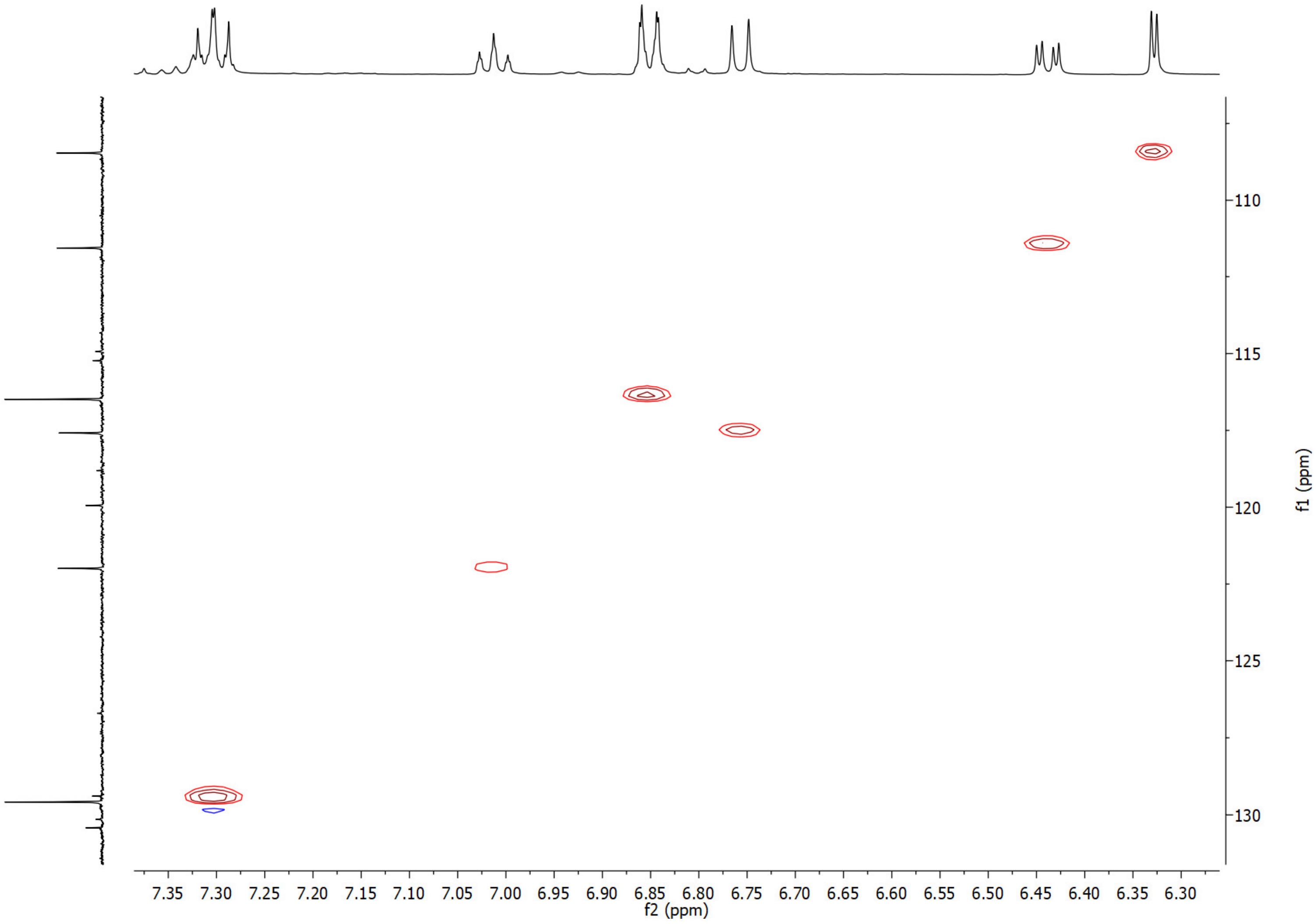

Figure S17.25. HSQC spectrum of **9a** in DMSO-d_6.

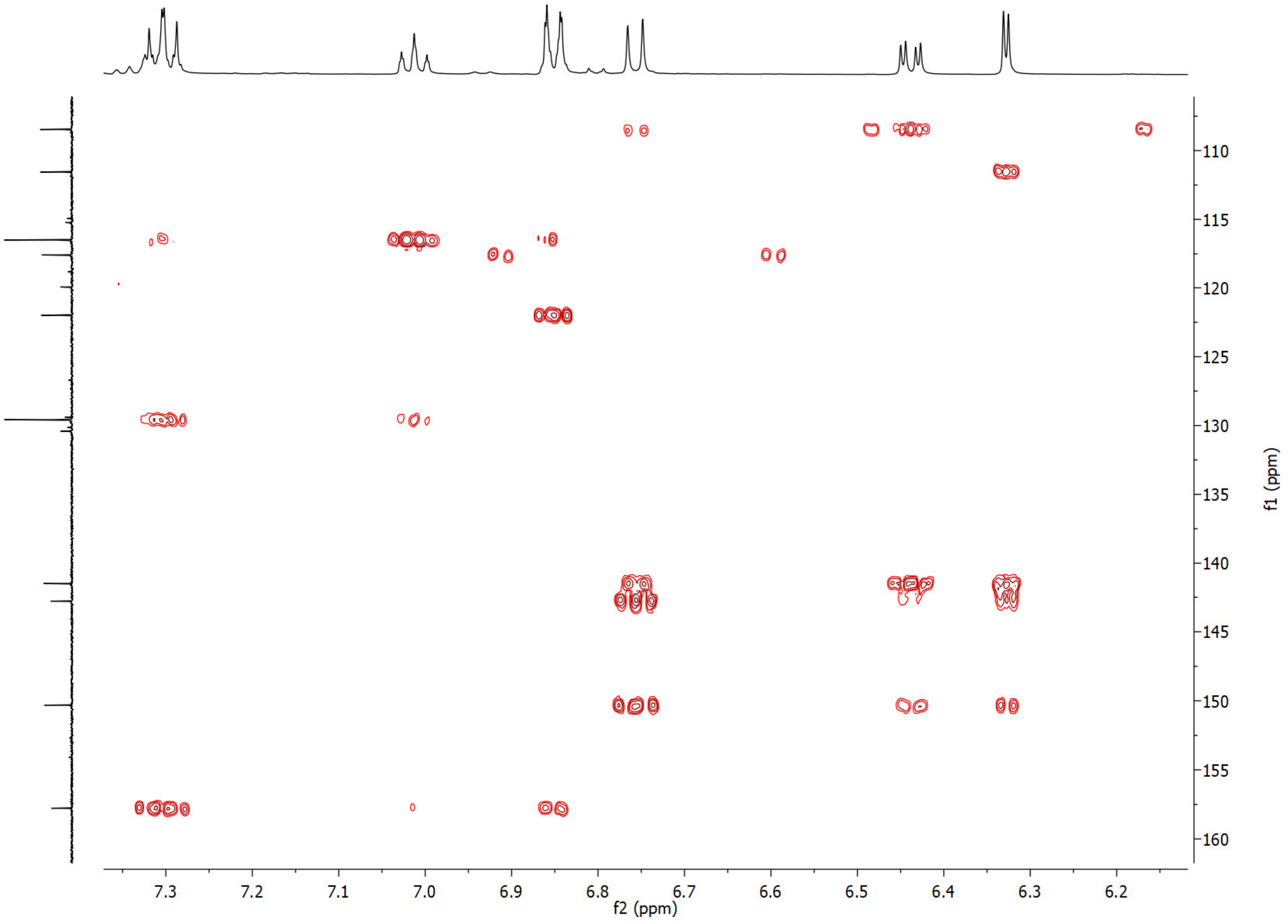

Figure S17.26. HMBC spectrum of **9a** in DMSO-d_6.

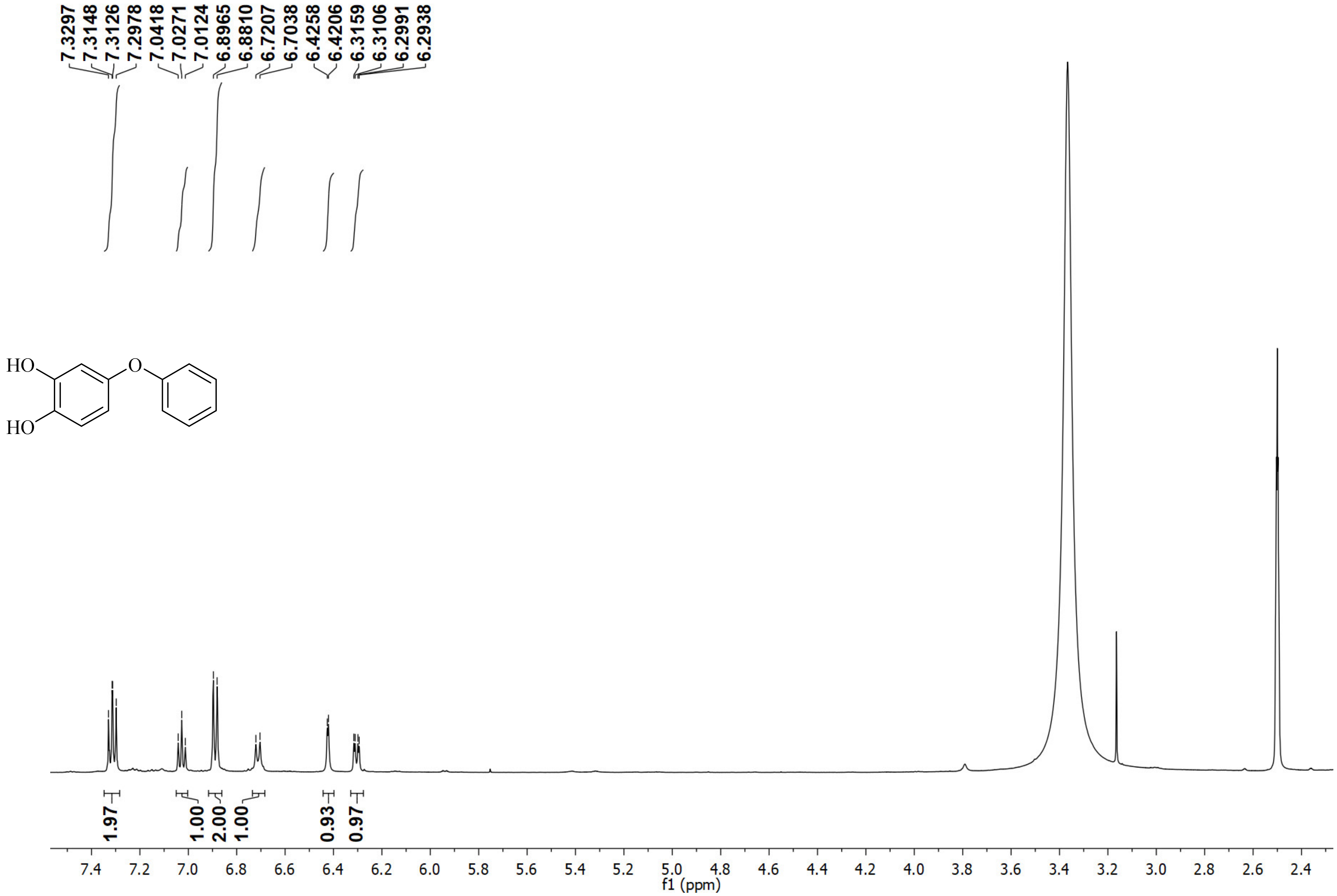

Figure S17.27. ^{1}H NMR spectrum of **9b** in DMSO-d_6.

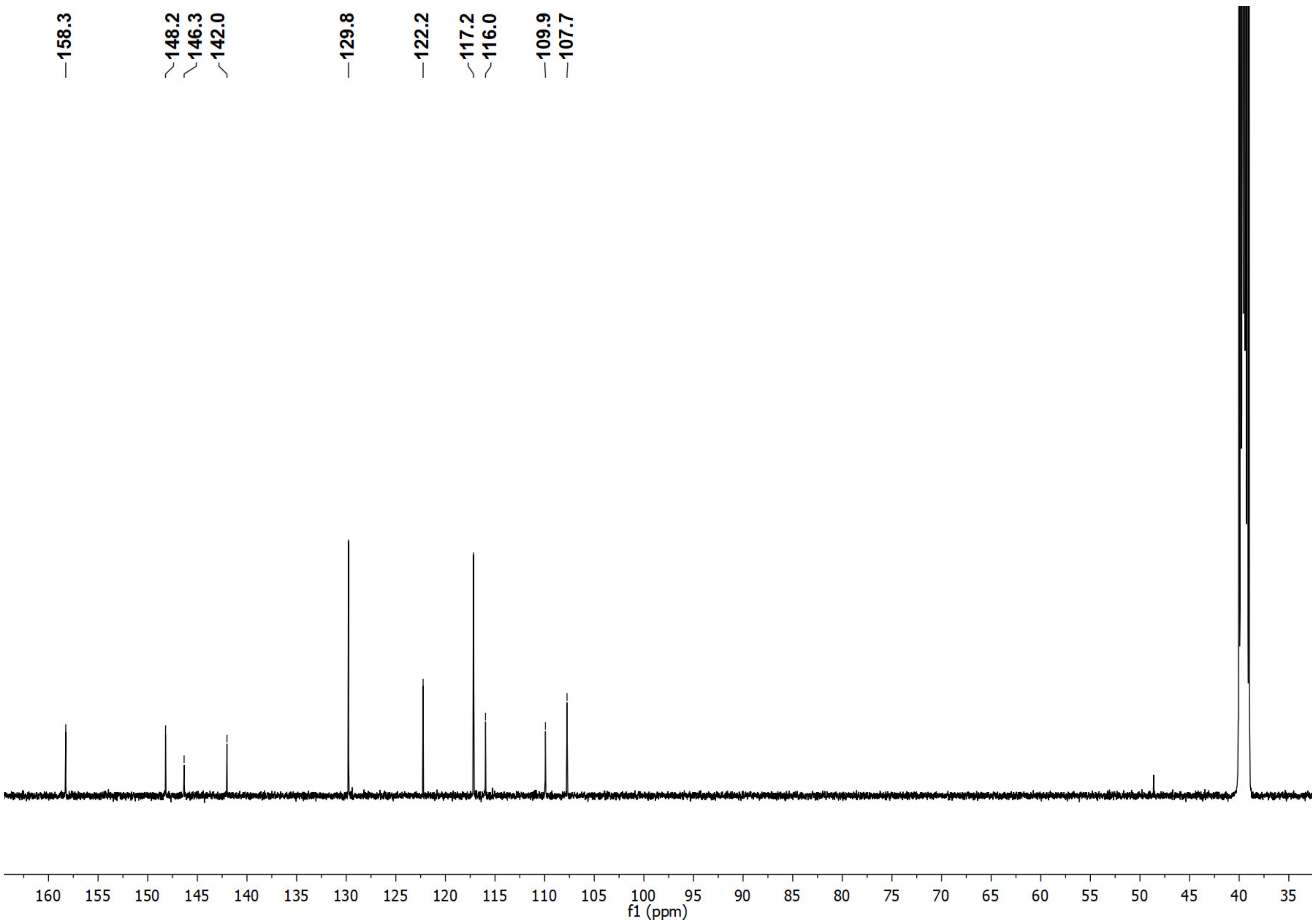

Figure S17.28. ^{13}C NMR spectrum of **9b** in DMSO-d_6.

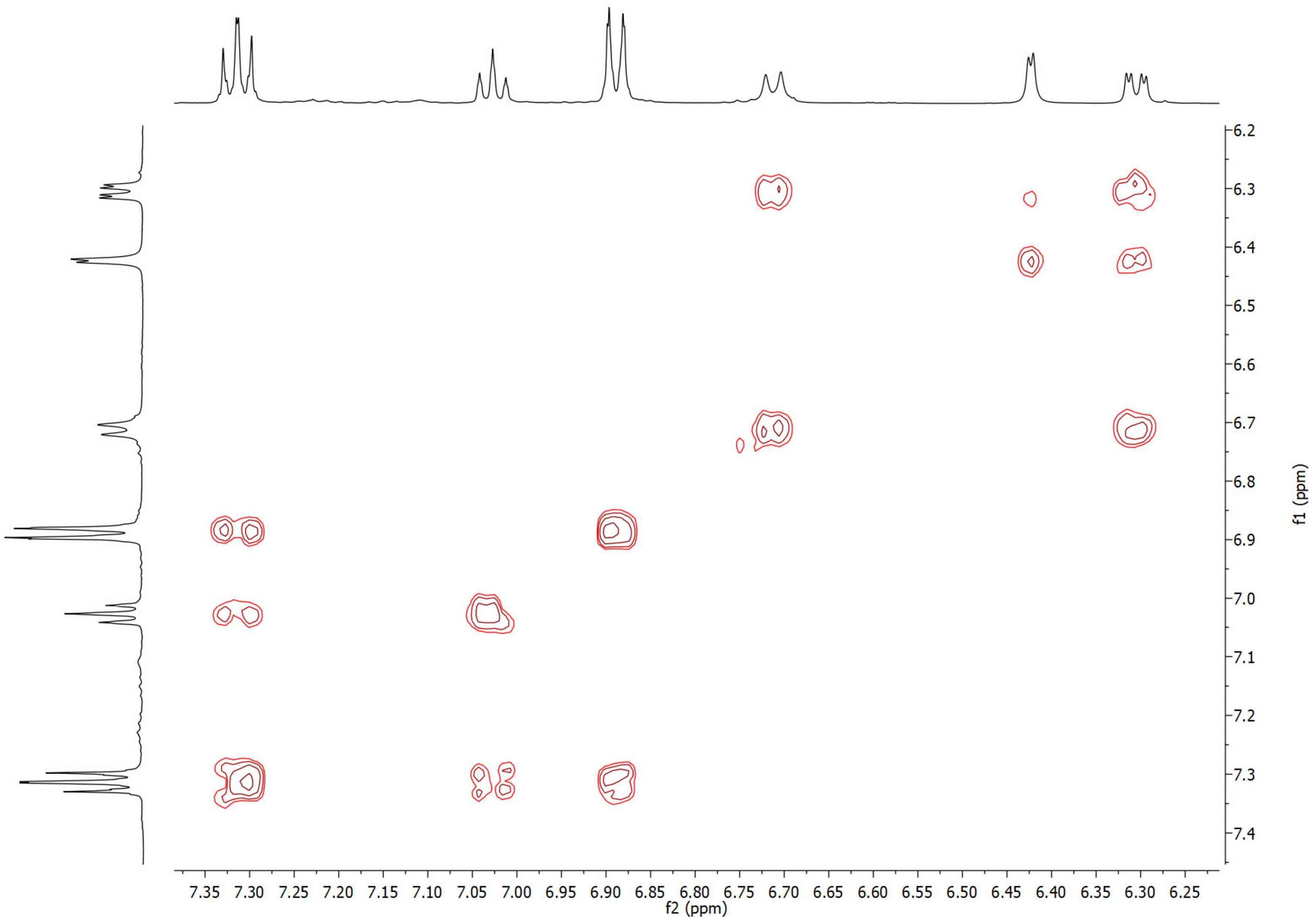

Figure S17.29. ^{1}H-^{1}H COSY spectrum of **9b** in DMSO-d_6.

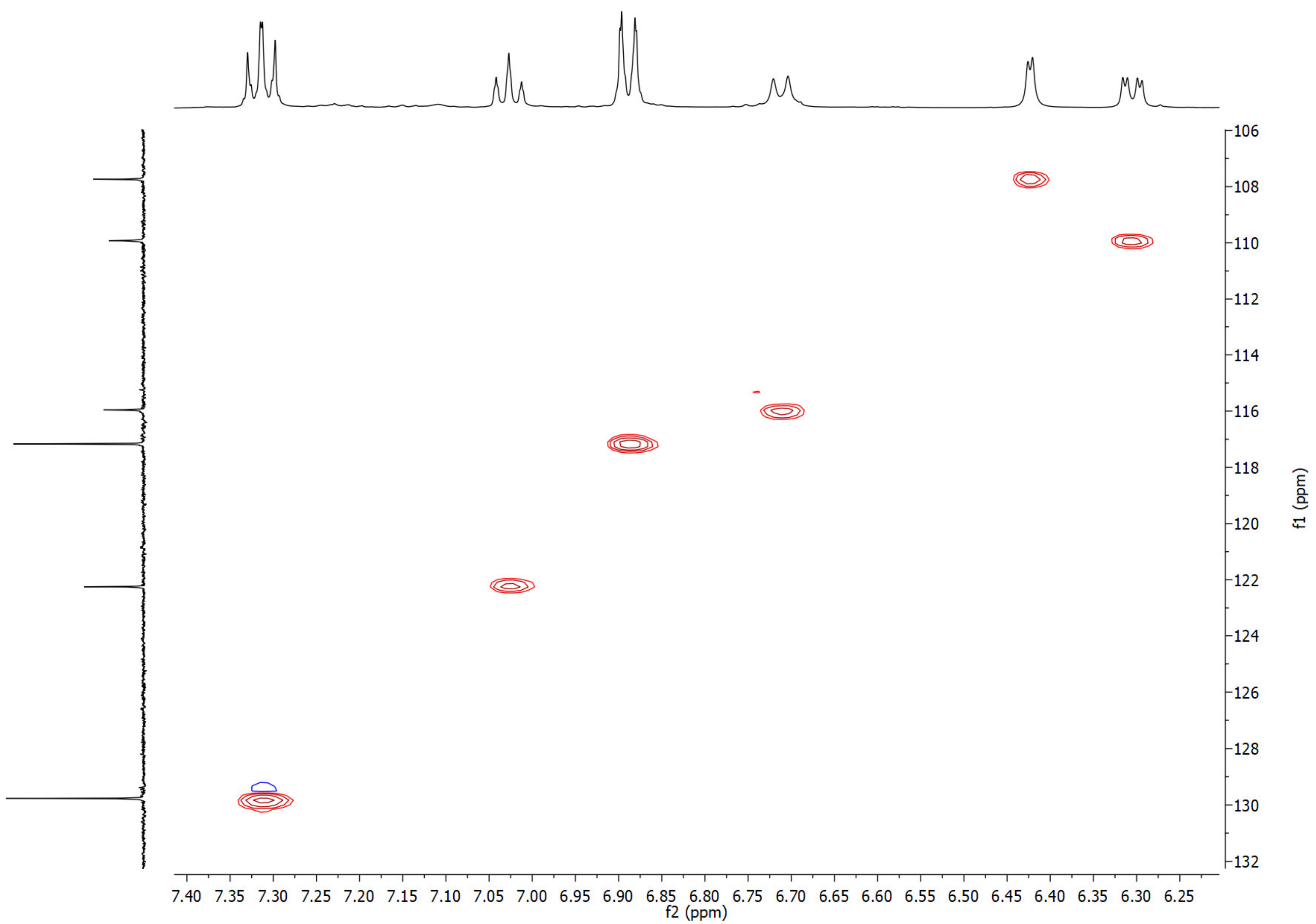

Figure S17.30. HSQC spectrum of **9b** in DMSO-d_6.

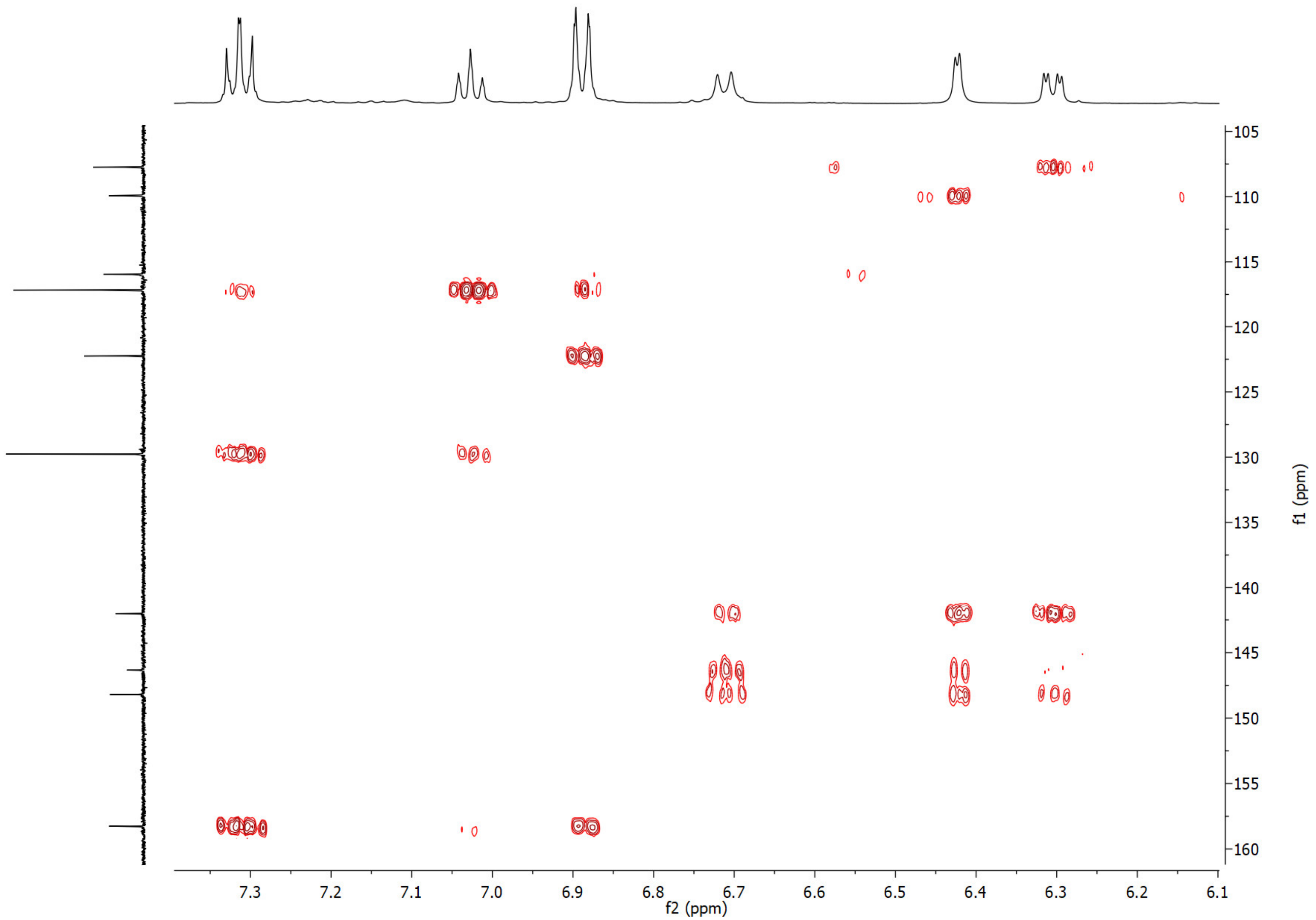

Figure S17.31. HMBC spectrum of **9b** in DMSO-d_6.

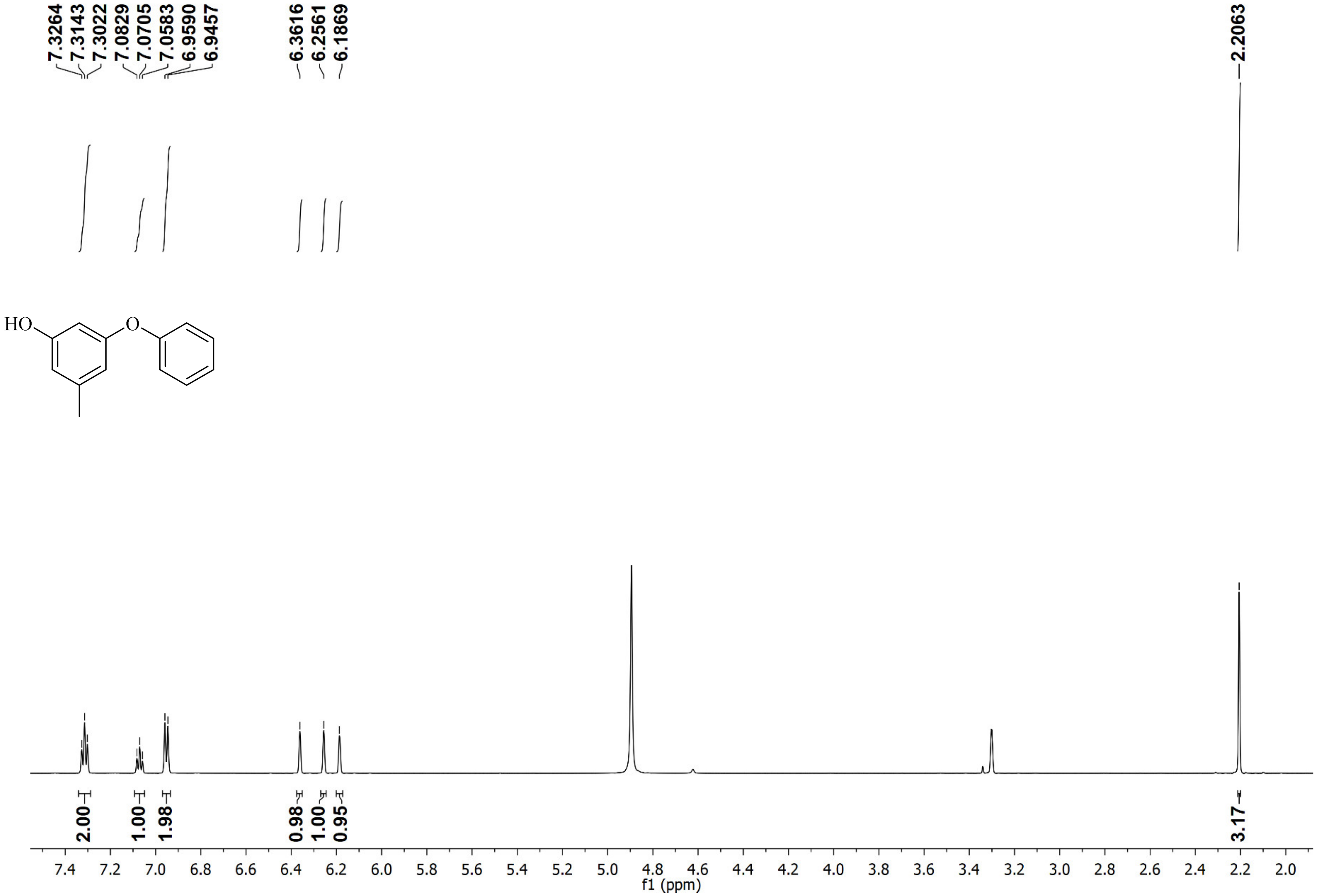

Figure S17.32. ^{1}H NMR spectrum of **10** in methanol-d_4.

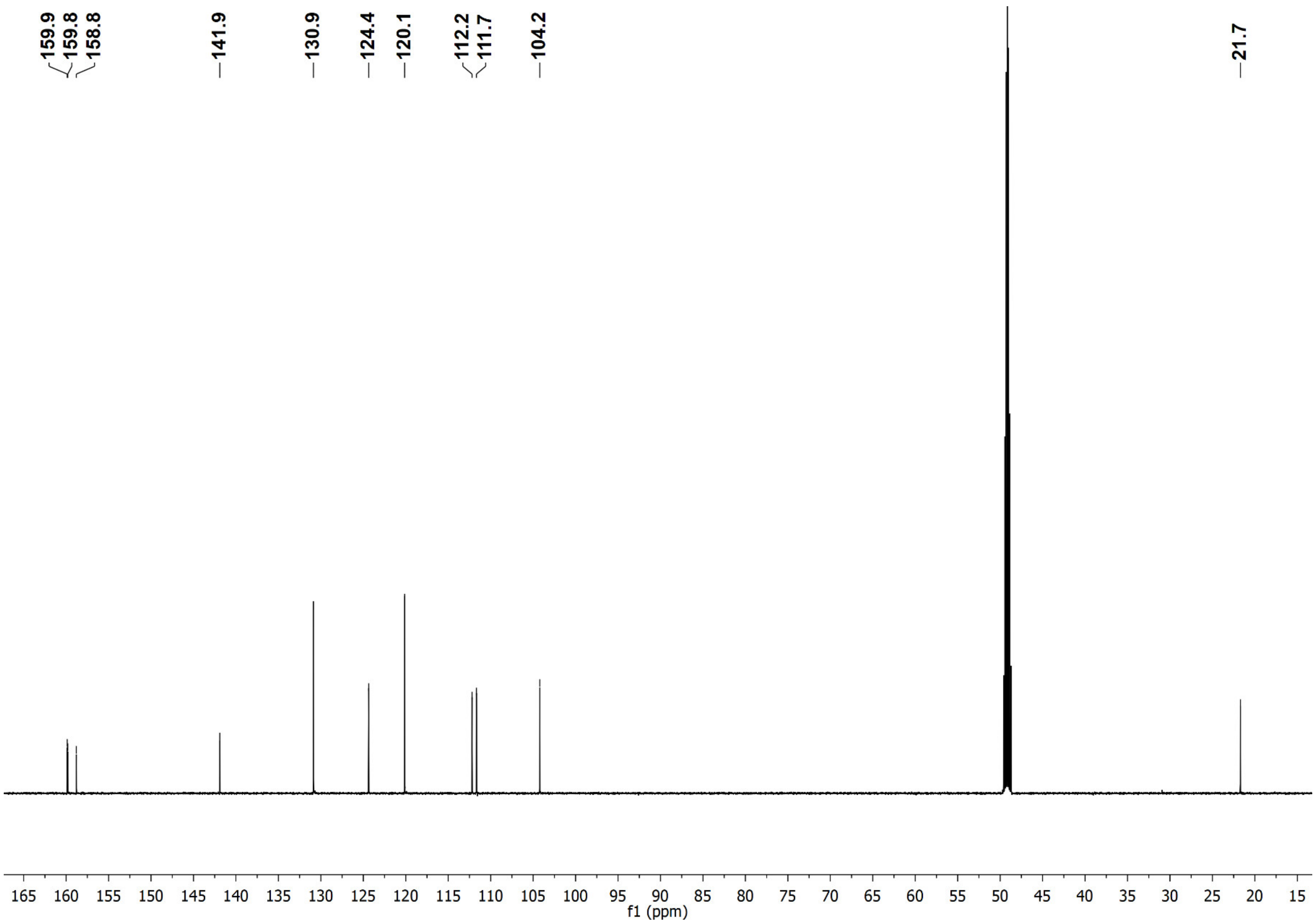

Figure S17.33. ^{13}C NMR spectrum of **10** in methanol-d_4.

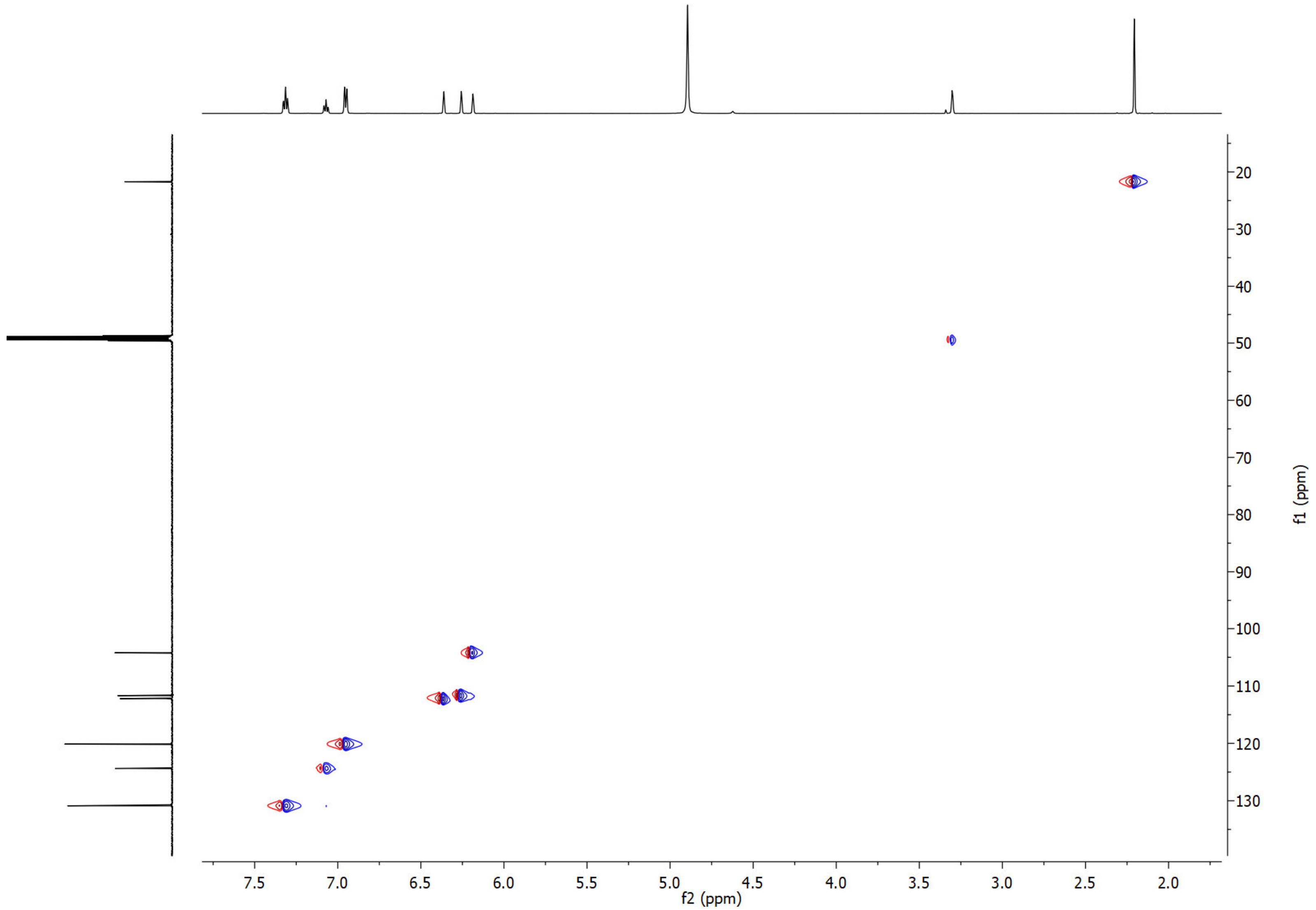

Figure S17.34. HSQC spectrum of **10** in methanol-d_4.

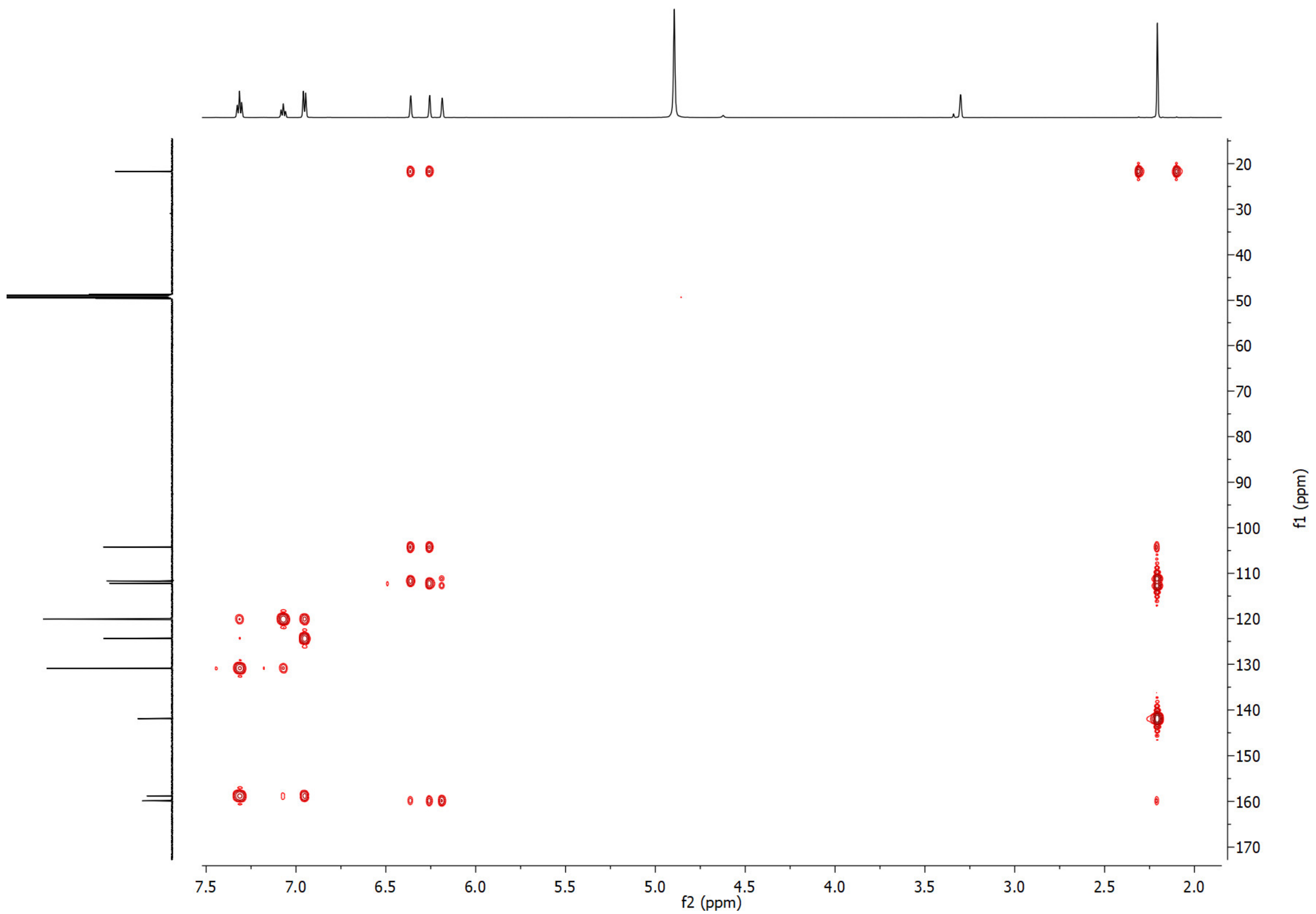

Figure S17.35. HMBC spectrum of **10** in methanol-d_4.

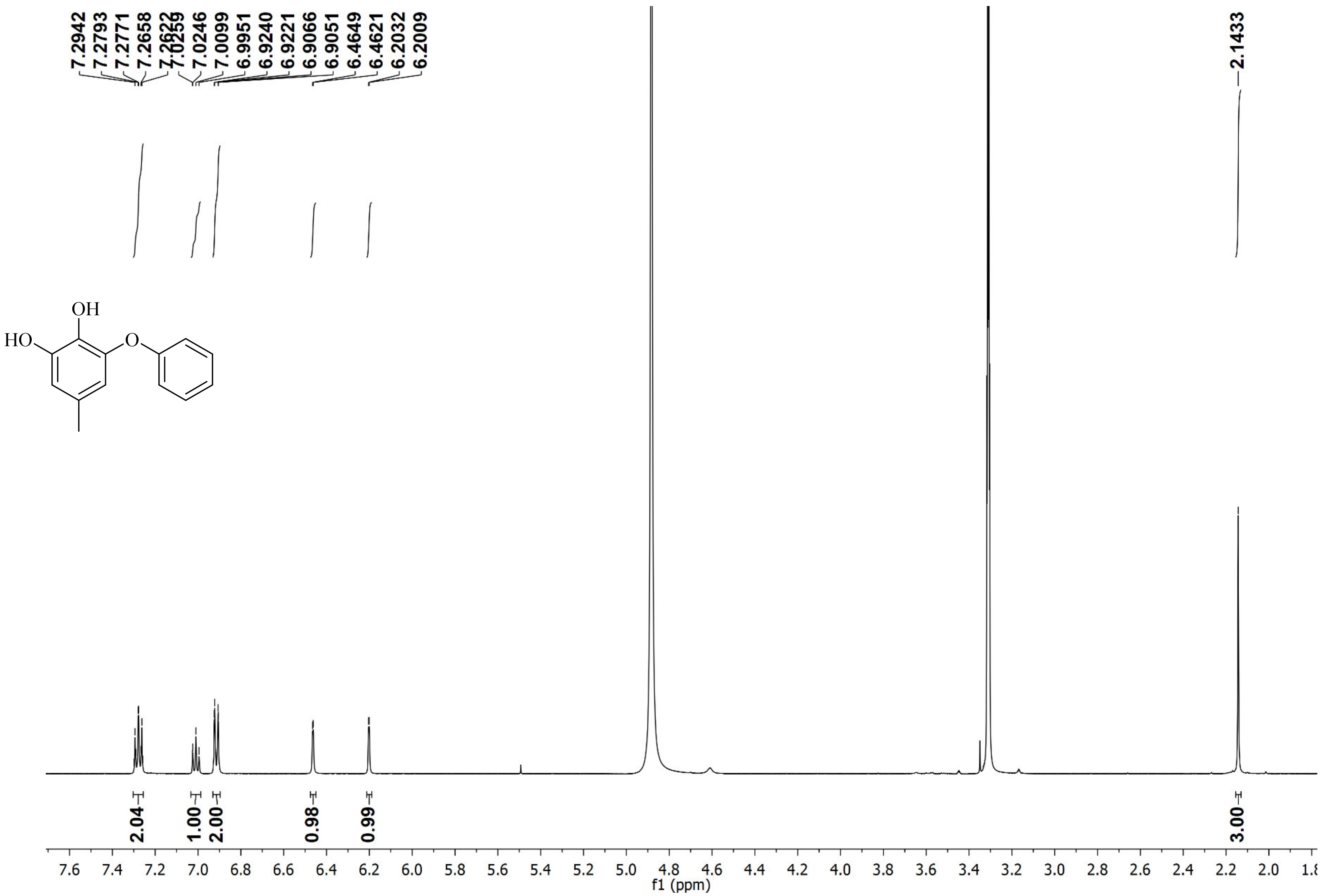

Figure S17.36. ^{1}H NMR spectrum of **10a** in methanol-d_4.

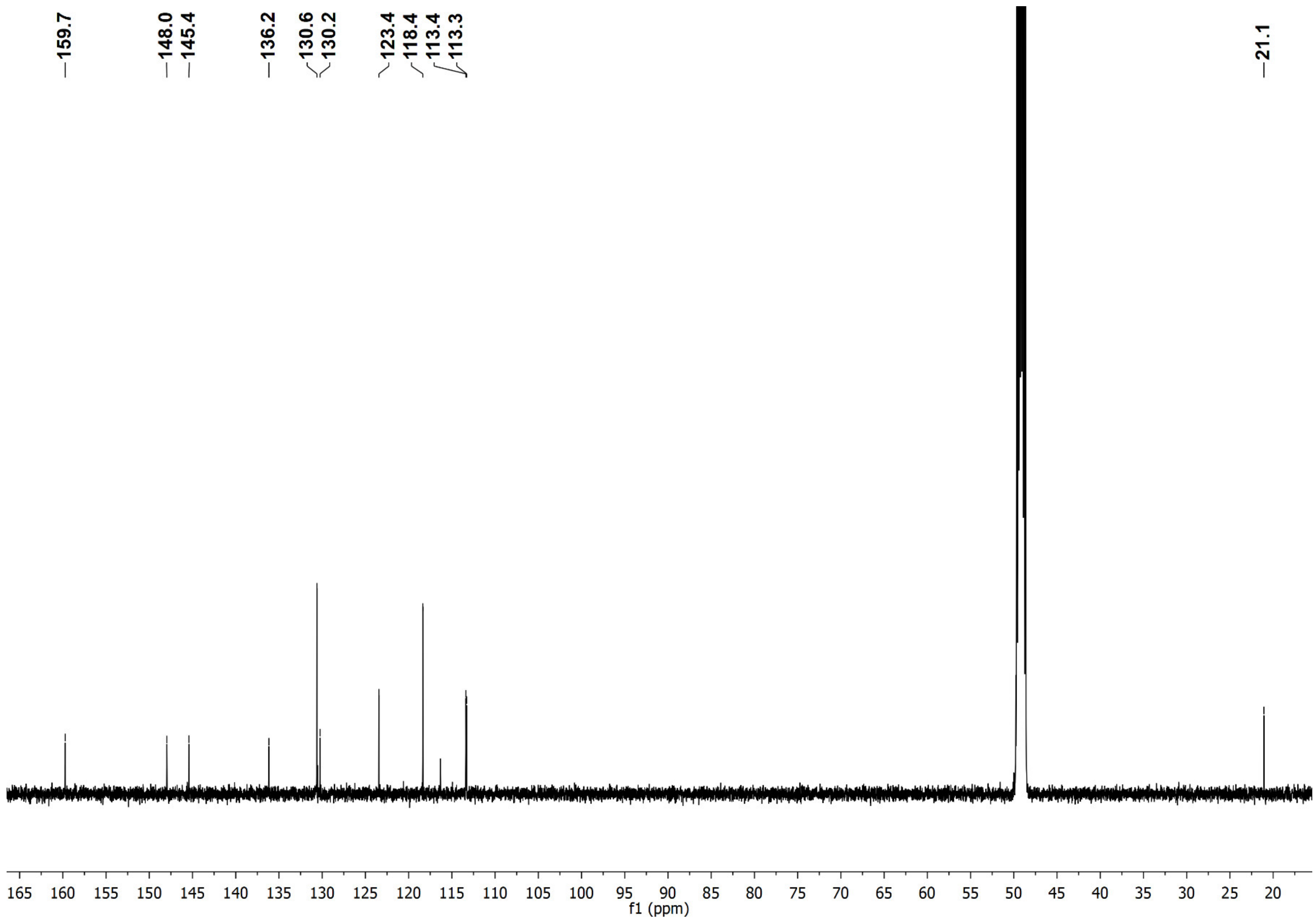

Figure S17.37. ^{13}C NMR spectrum of **10a** in methanol-d_4.

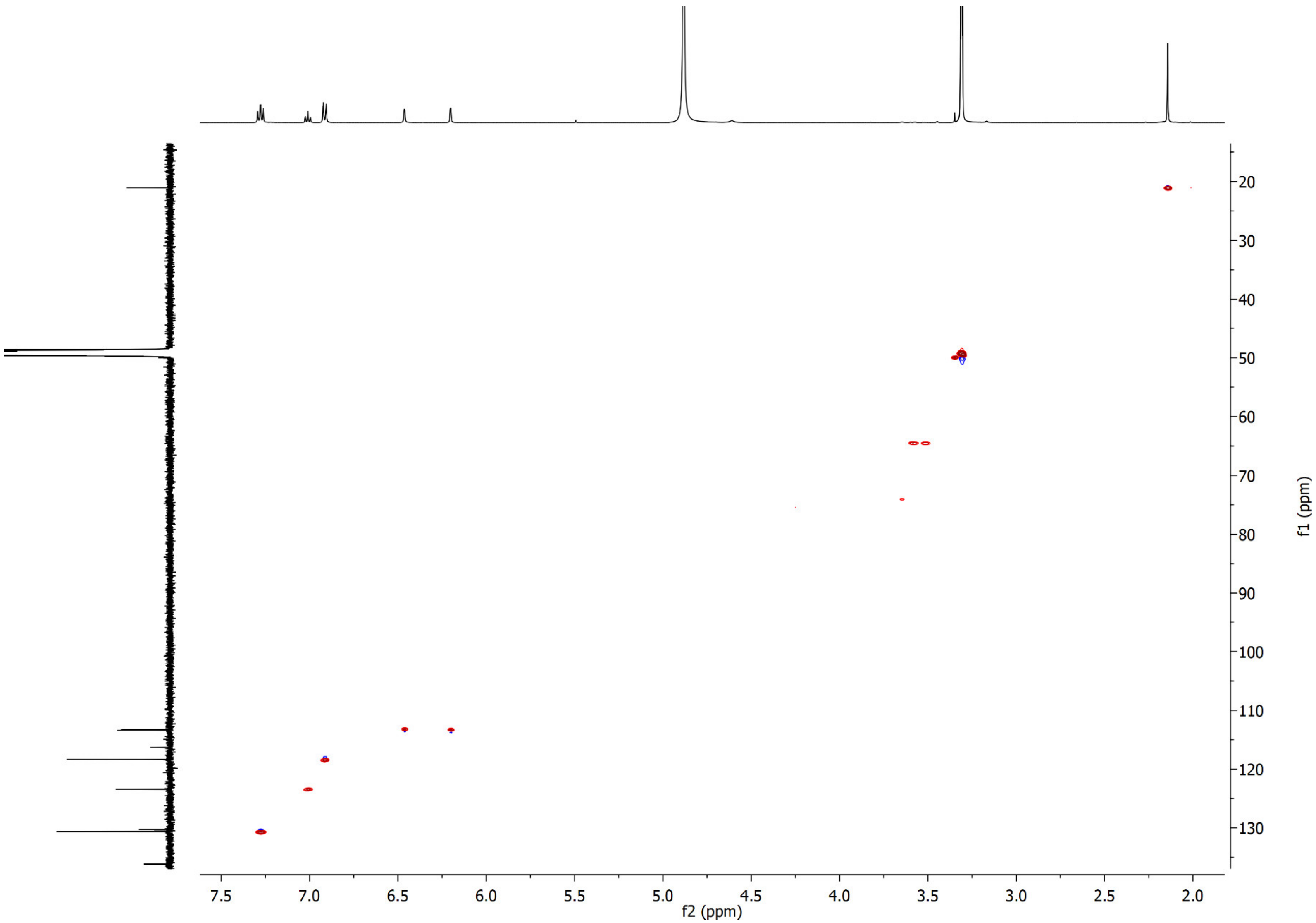

Figure S17.38. HSQC spectrum of **10a** in methanol-d_4.

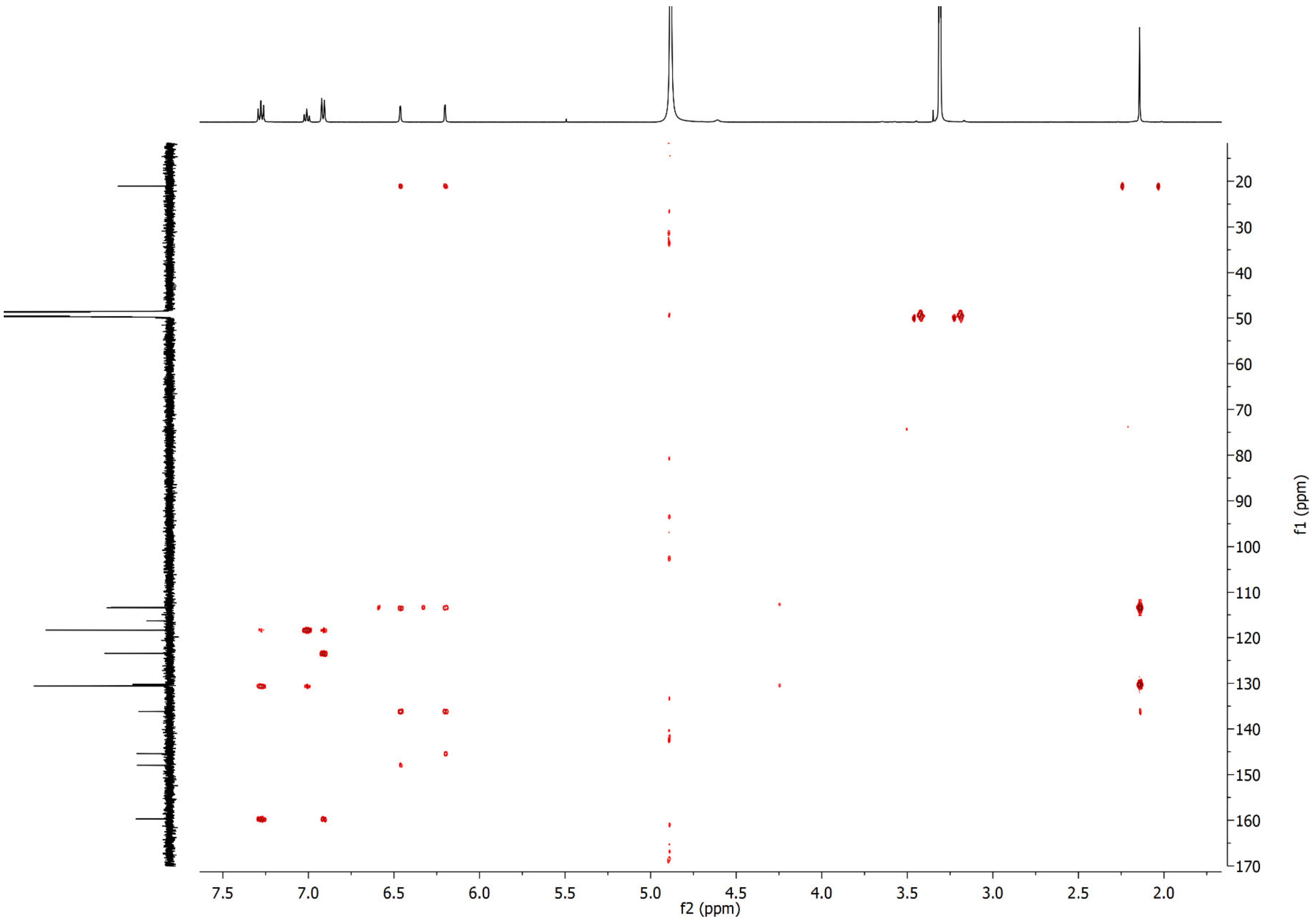

Figure S17.39. HMBC spectrum of **10a** in methanol-d_4.

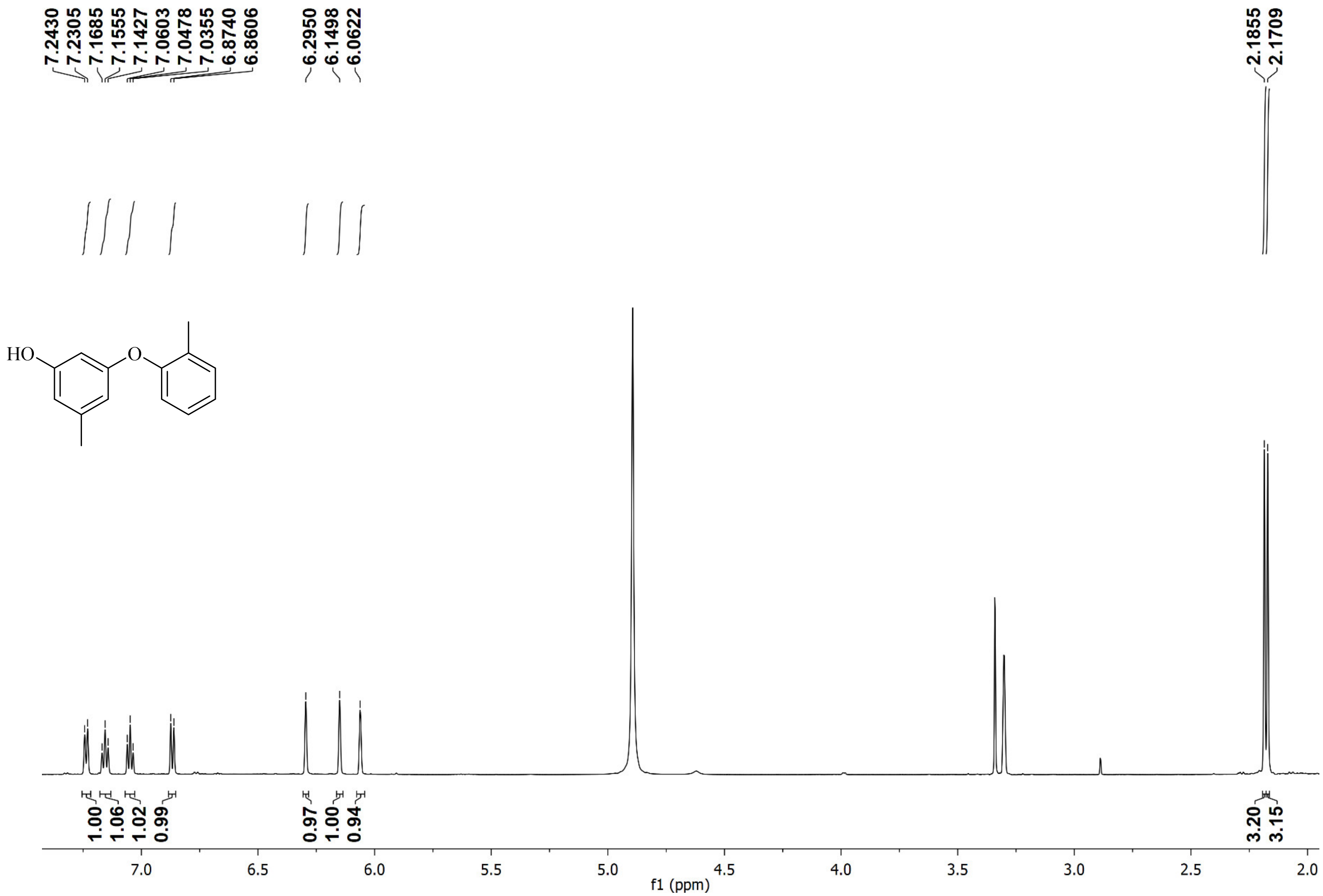

Figure S17.40. ^{1}H NMR spectrum of **11** in methanol-d_4.

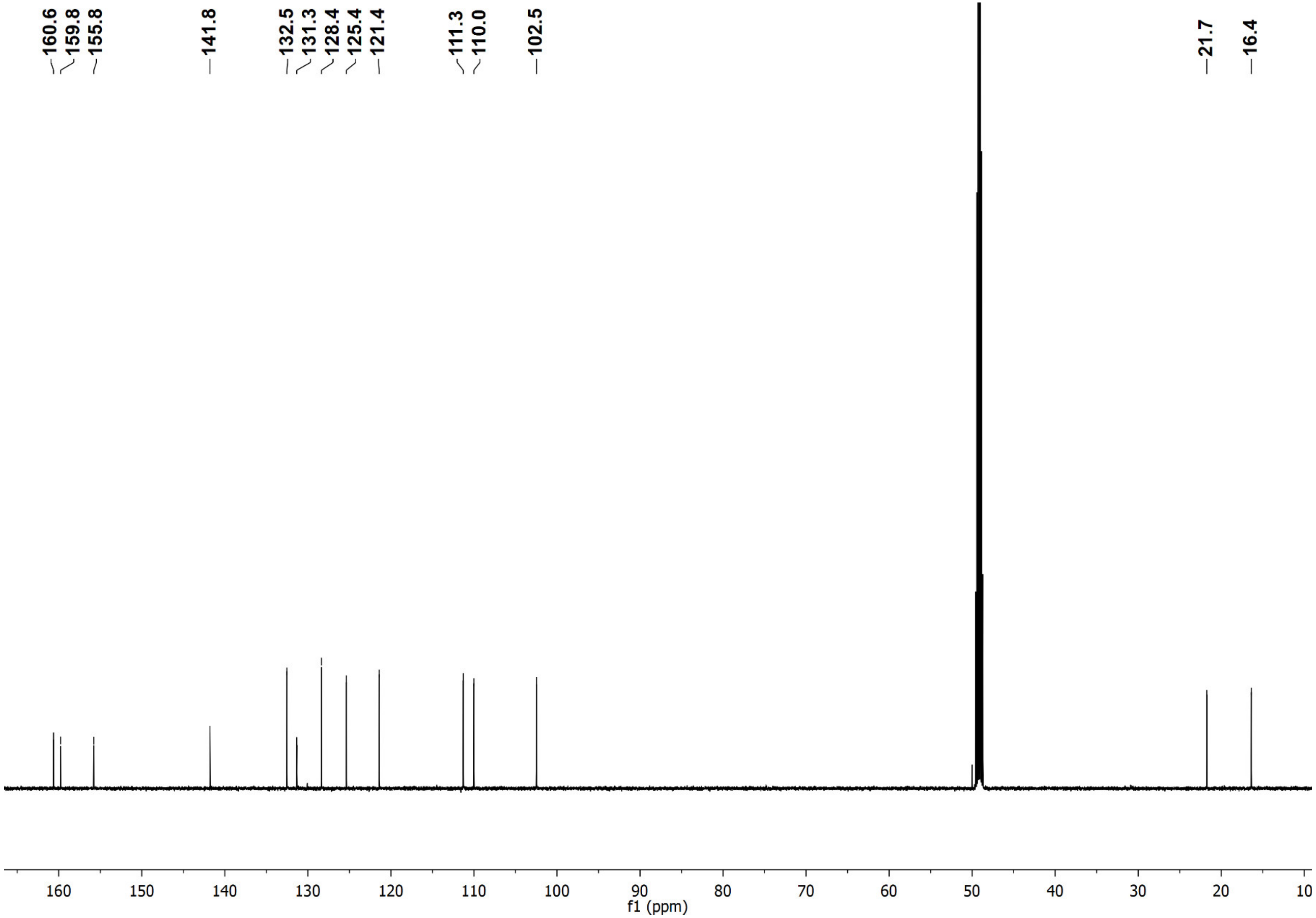

Figure S17.41. ^{13}C NMR spectrum of **11** in methanol-d_4.

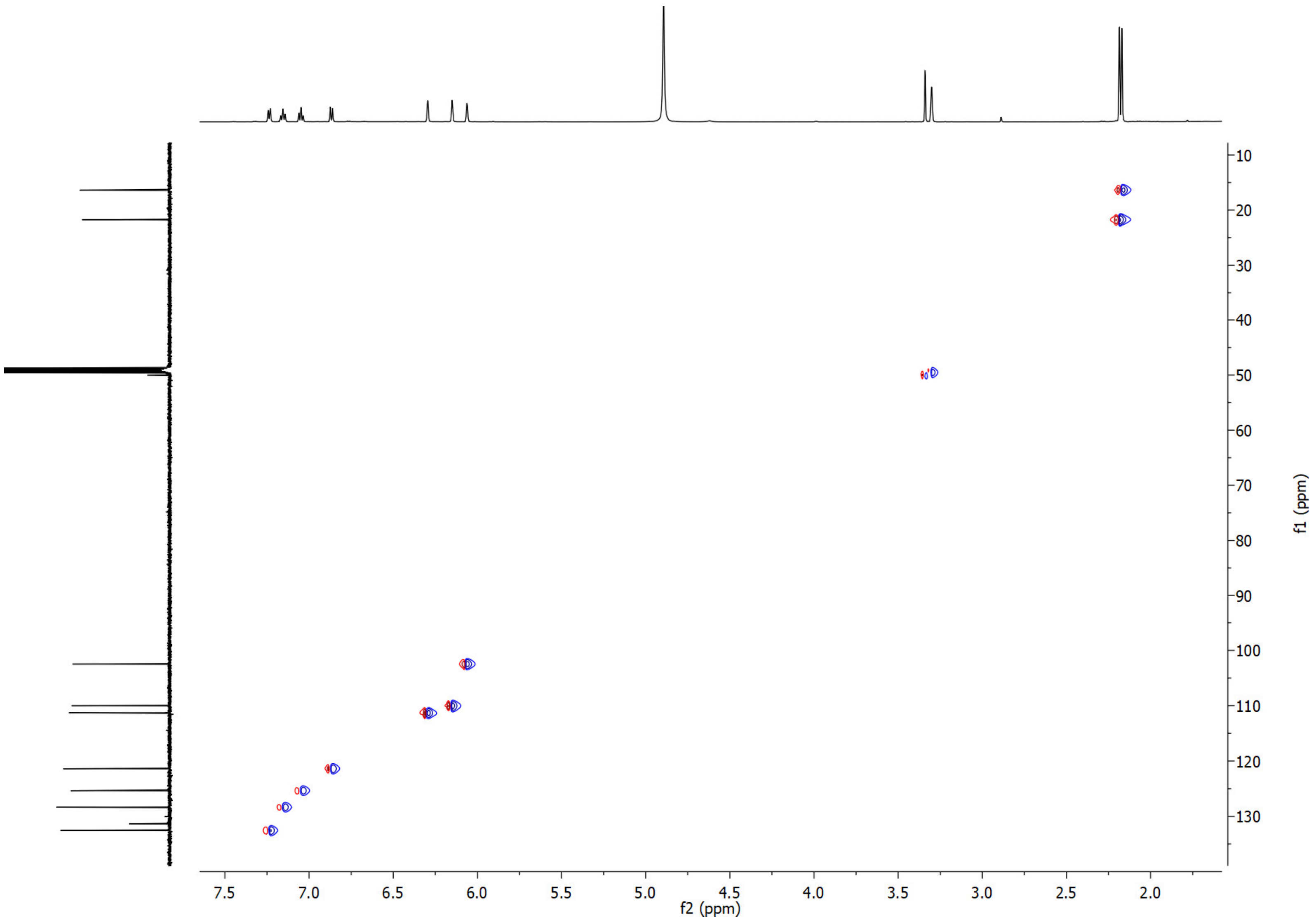

Figure S17.42. HSQC spectrum of **11** in methanol-d_4.

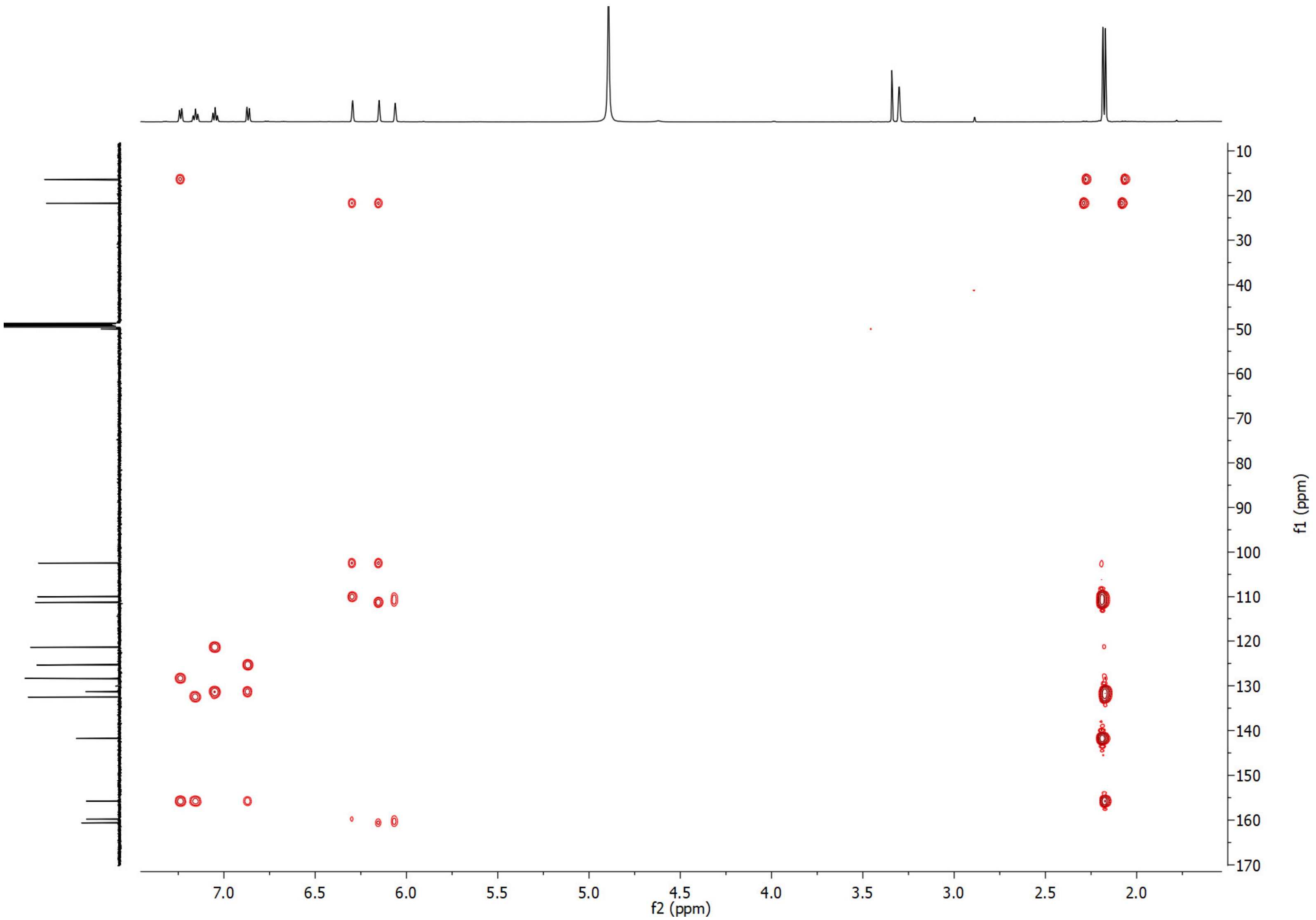

Figure S17.43. HMBC spectrum of **11** in methanol-d_4.

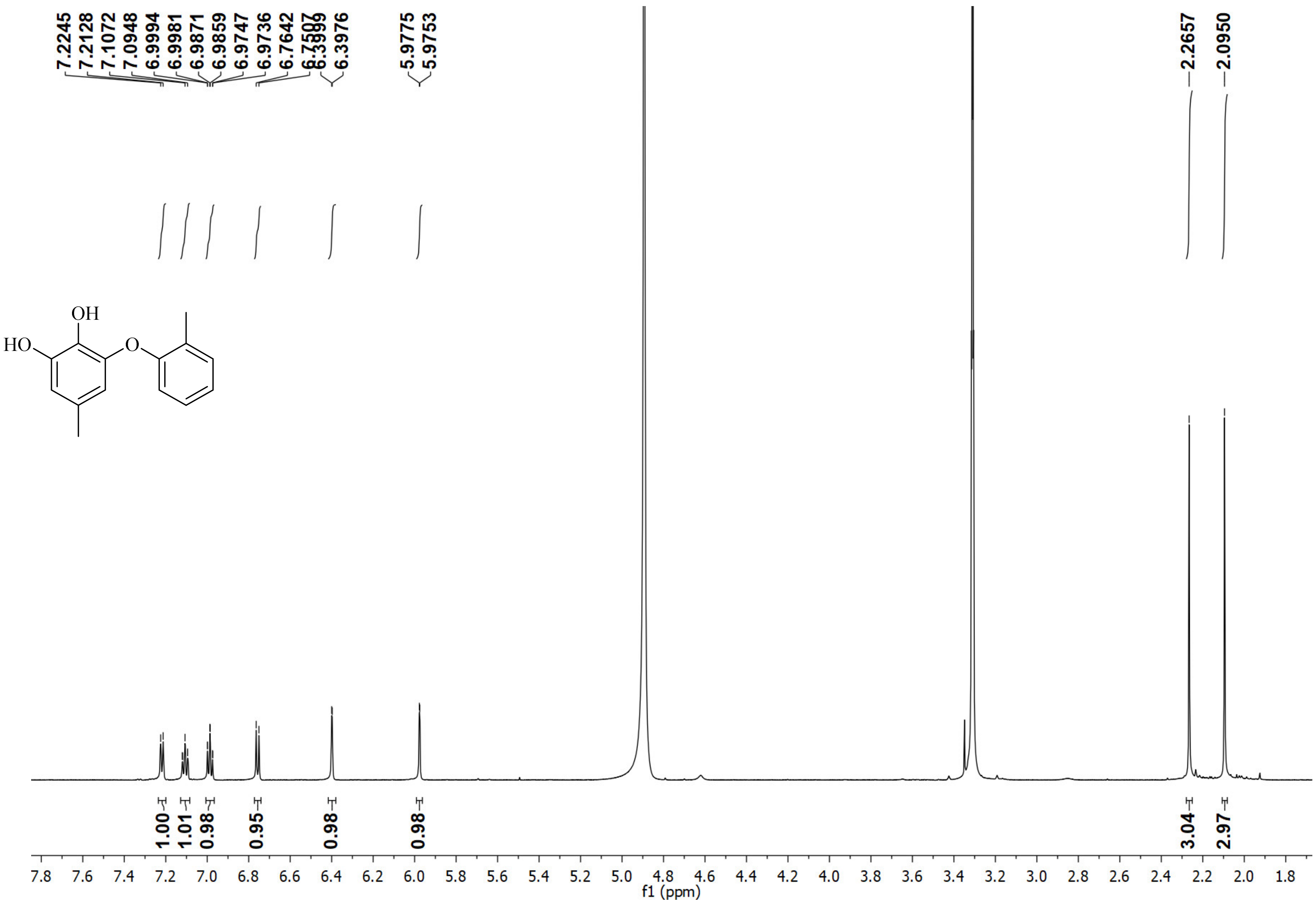

Figure S17.44. ^{1}H NMR spectrum of **11a** in methanol-d_4.

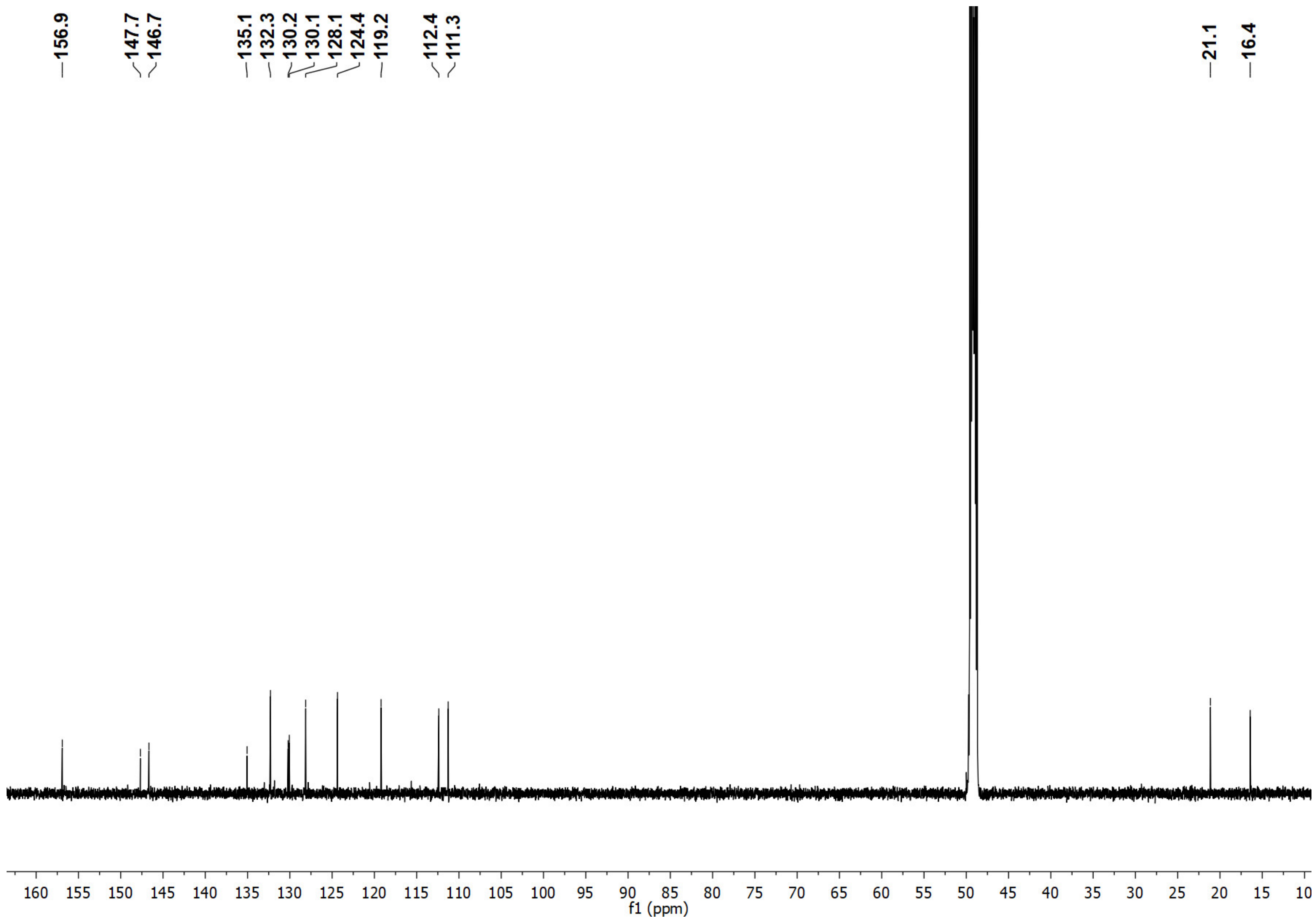

Figure S17.45. ^{13}C NMR spectrum of **11a** in methanol-d_4.

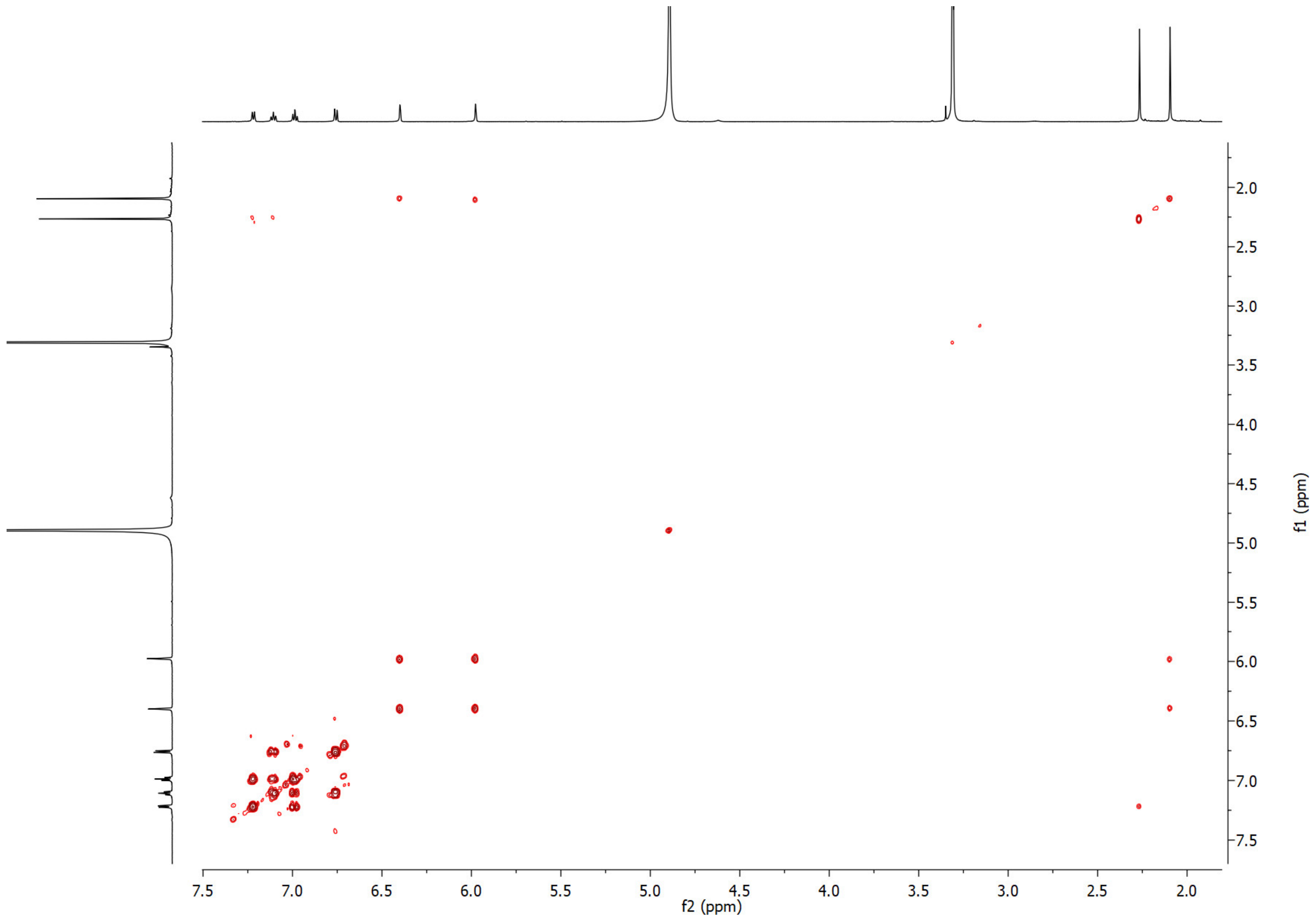

Figure S17.46. ^{1}H-^{1}H COSY spectrum of **11a** in methanol-d_4.

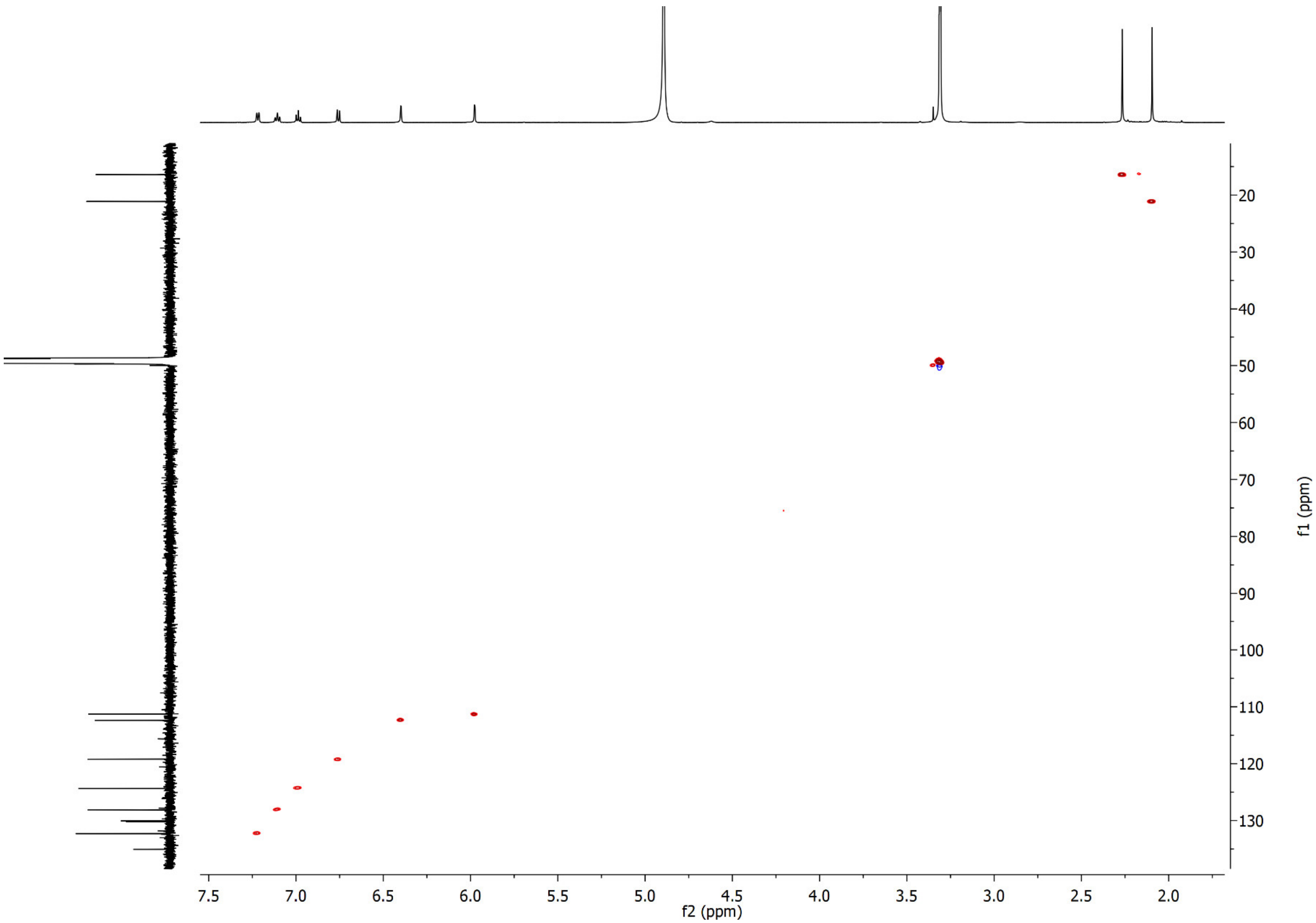

Figure S17.47. HSQC spectrum of **11a** in methanol-d_4.

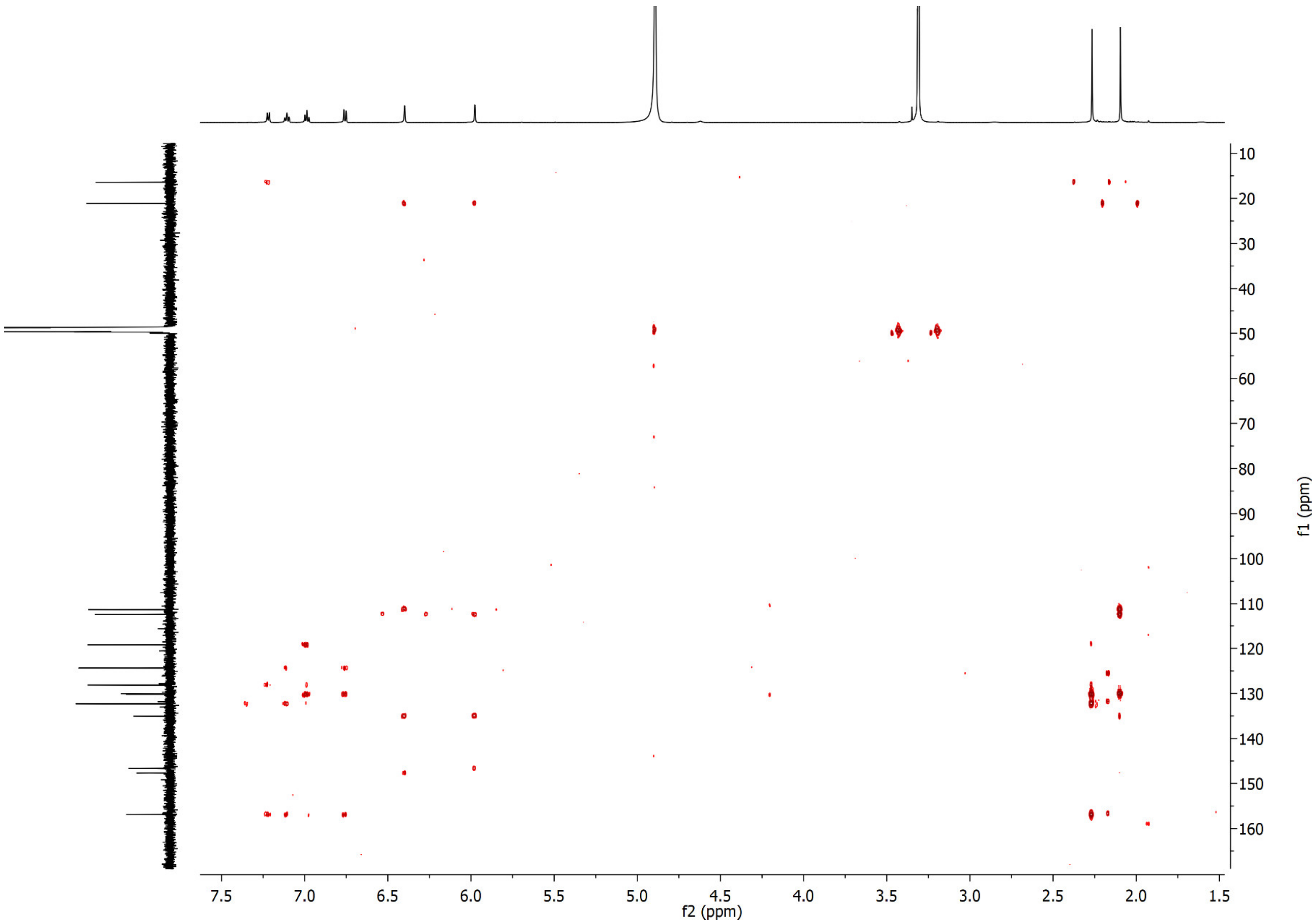

Figure S17.48. HMBC spectrum of **11a** in methanol-d_4.

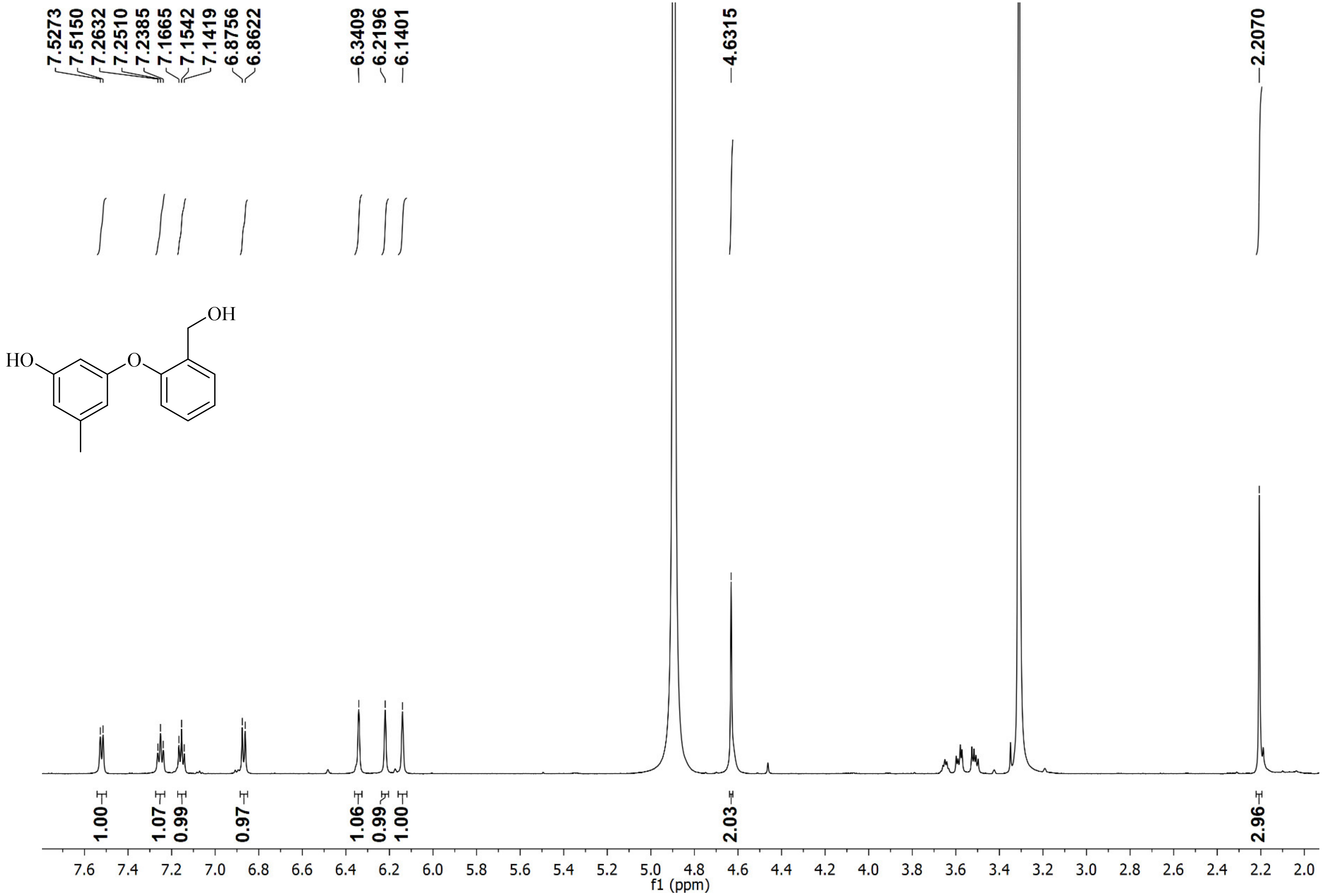

Figure S17.49. ^{1}H NMR spectrum of **11c** in methanol-d_4.

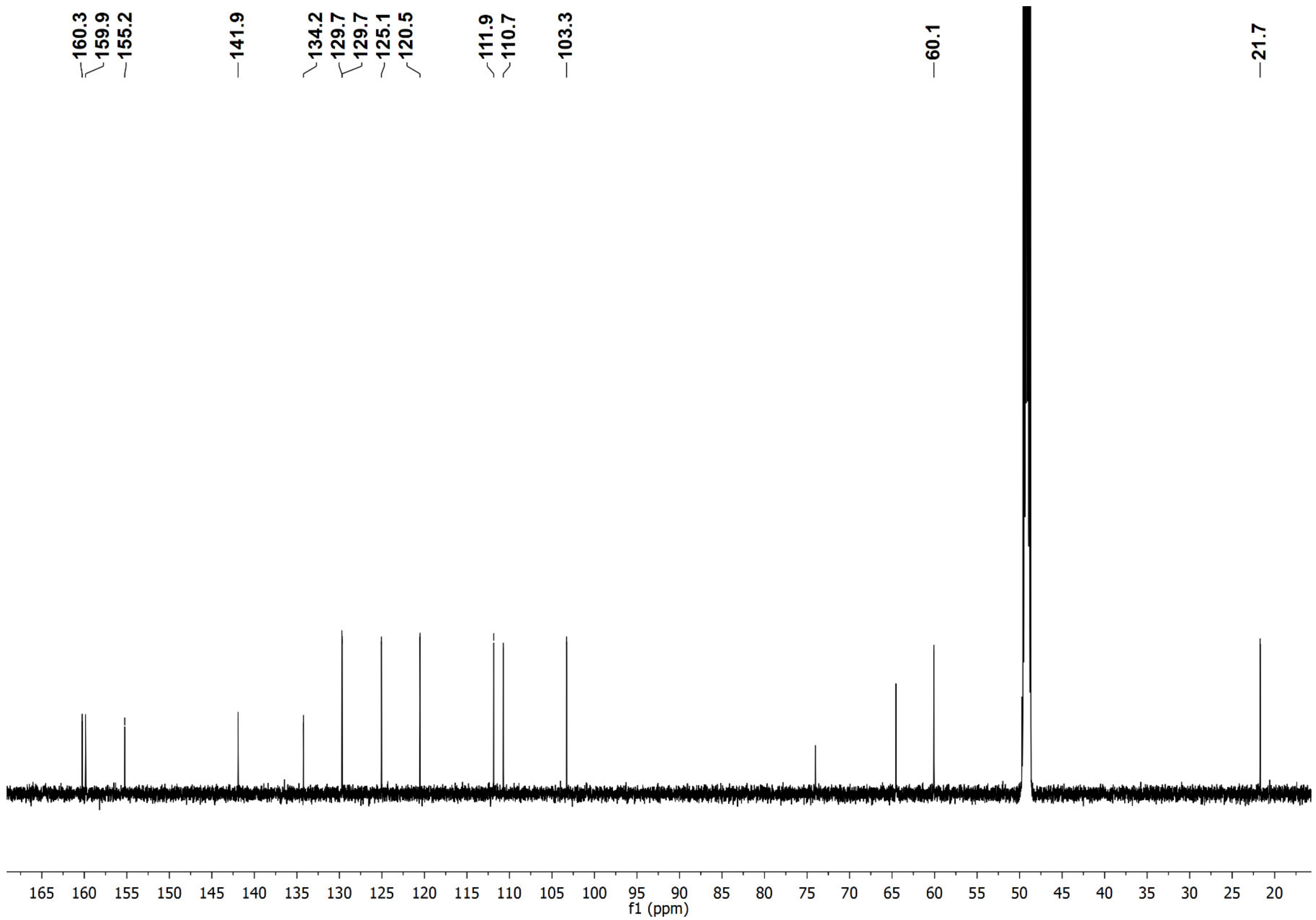

Figure S17.50. ^{13}C NMR spectrum of **11c** in methanol-d_4.

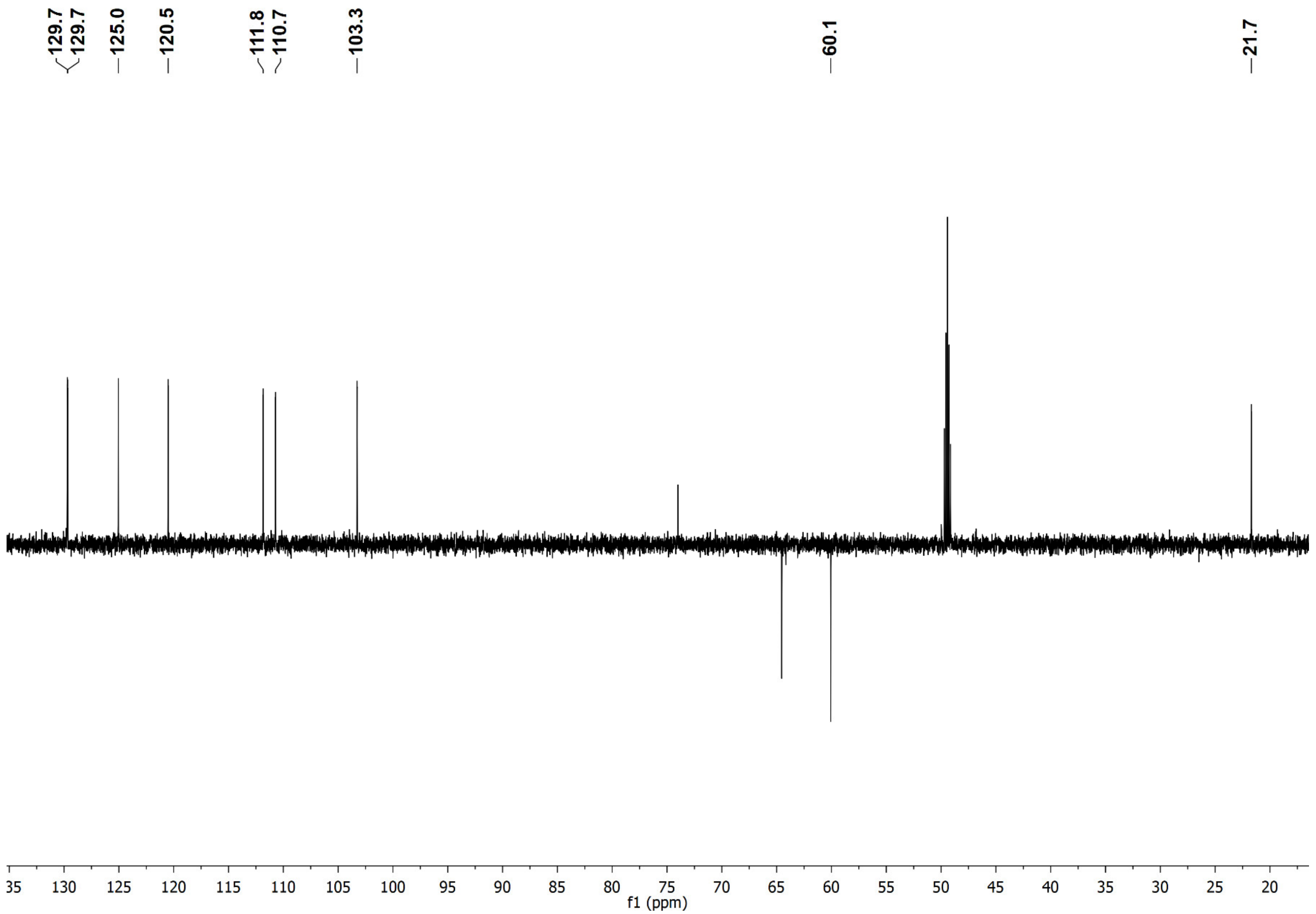

Figure S17.51. DEPT 135 spectrum of **11c** in methanol-d_4.

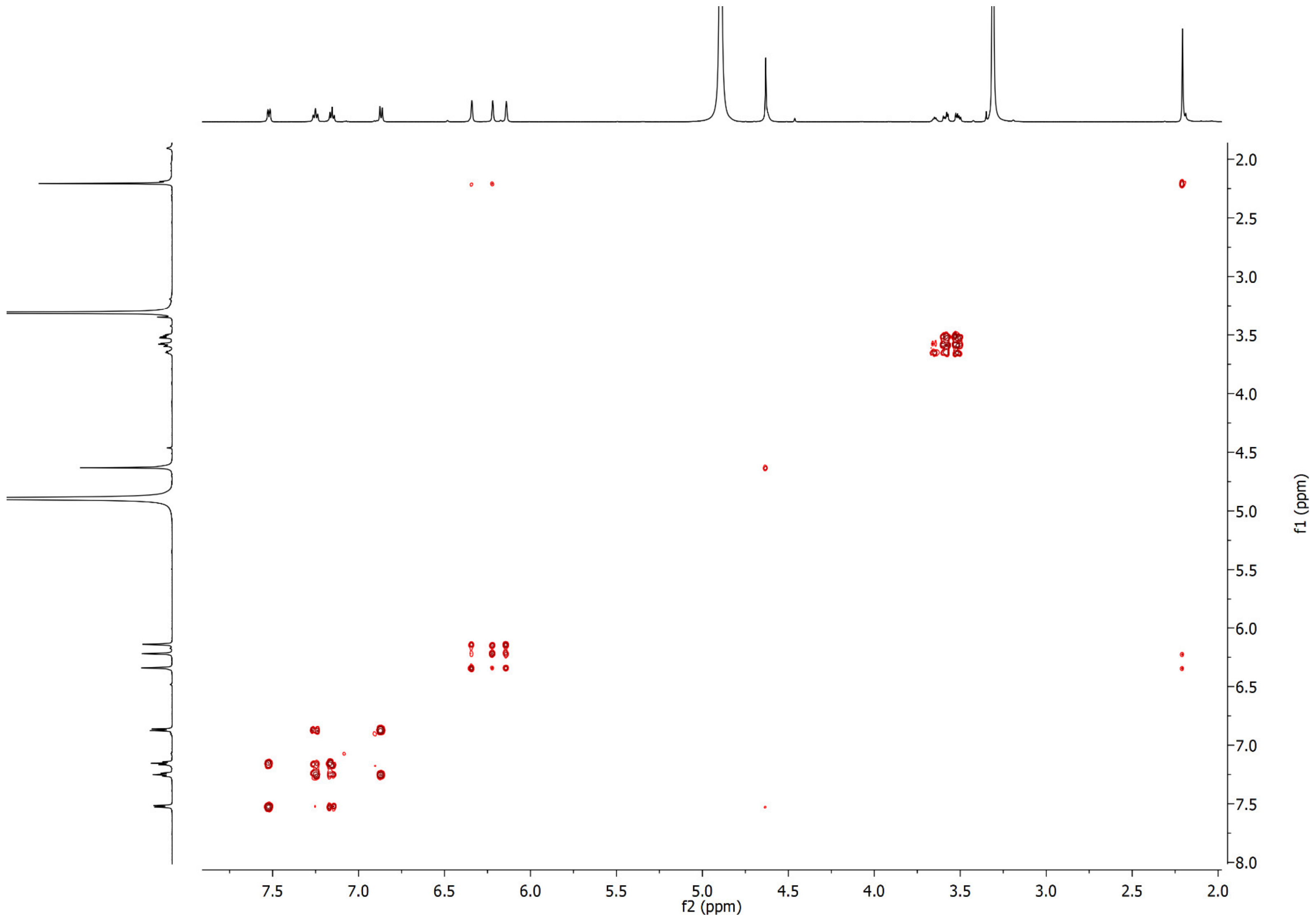

Figure S17.52. ^{1}H-^{1}H COSY spectrum of **11c** in methanol-*d*$_4$.

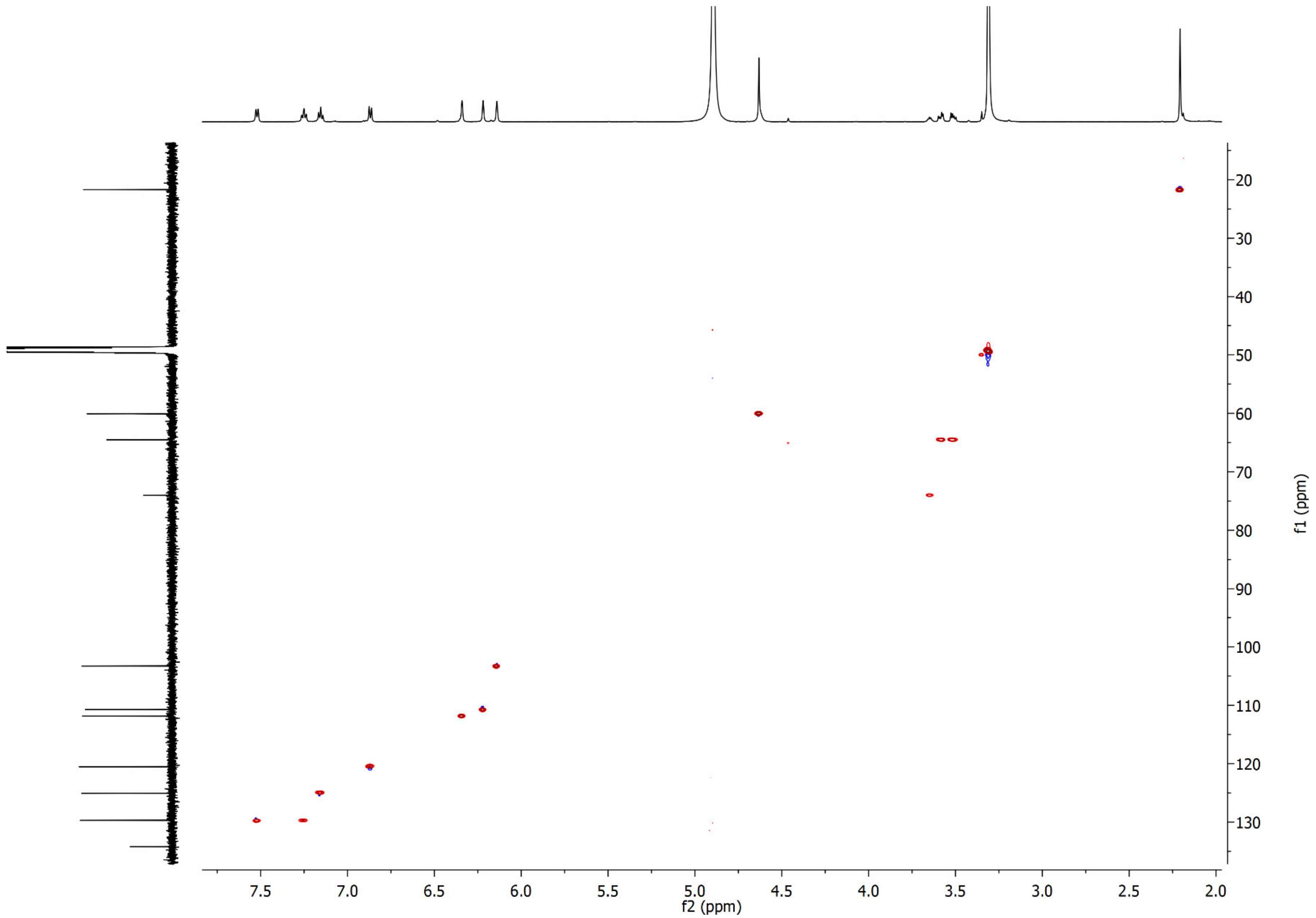

Figure S17.53. HSQC spectrum of **11c** in methanol-d_4.

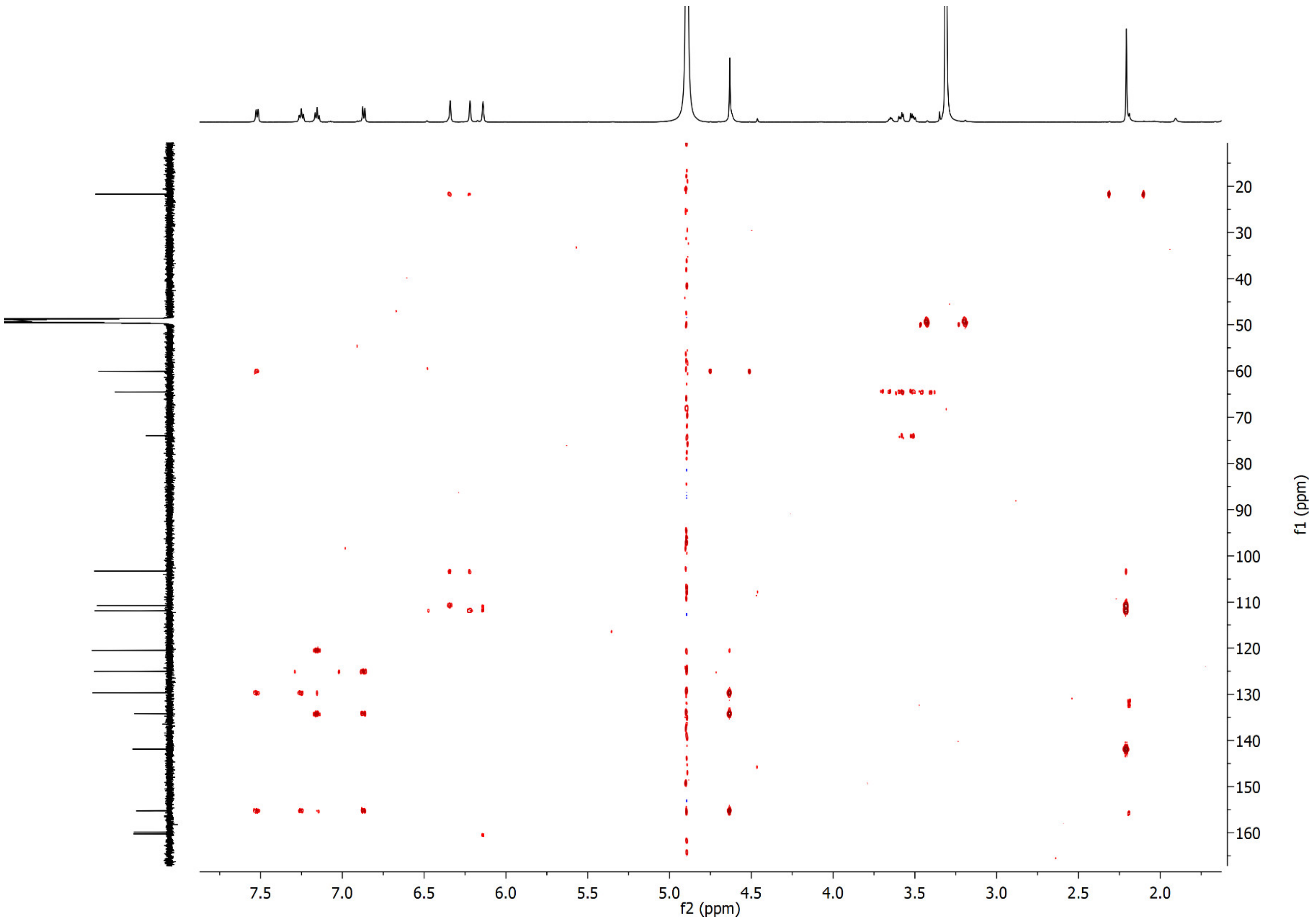

Figure S17.54. HMBC spectrum of **11c** in methanol-d_4.

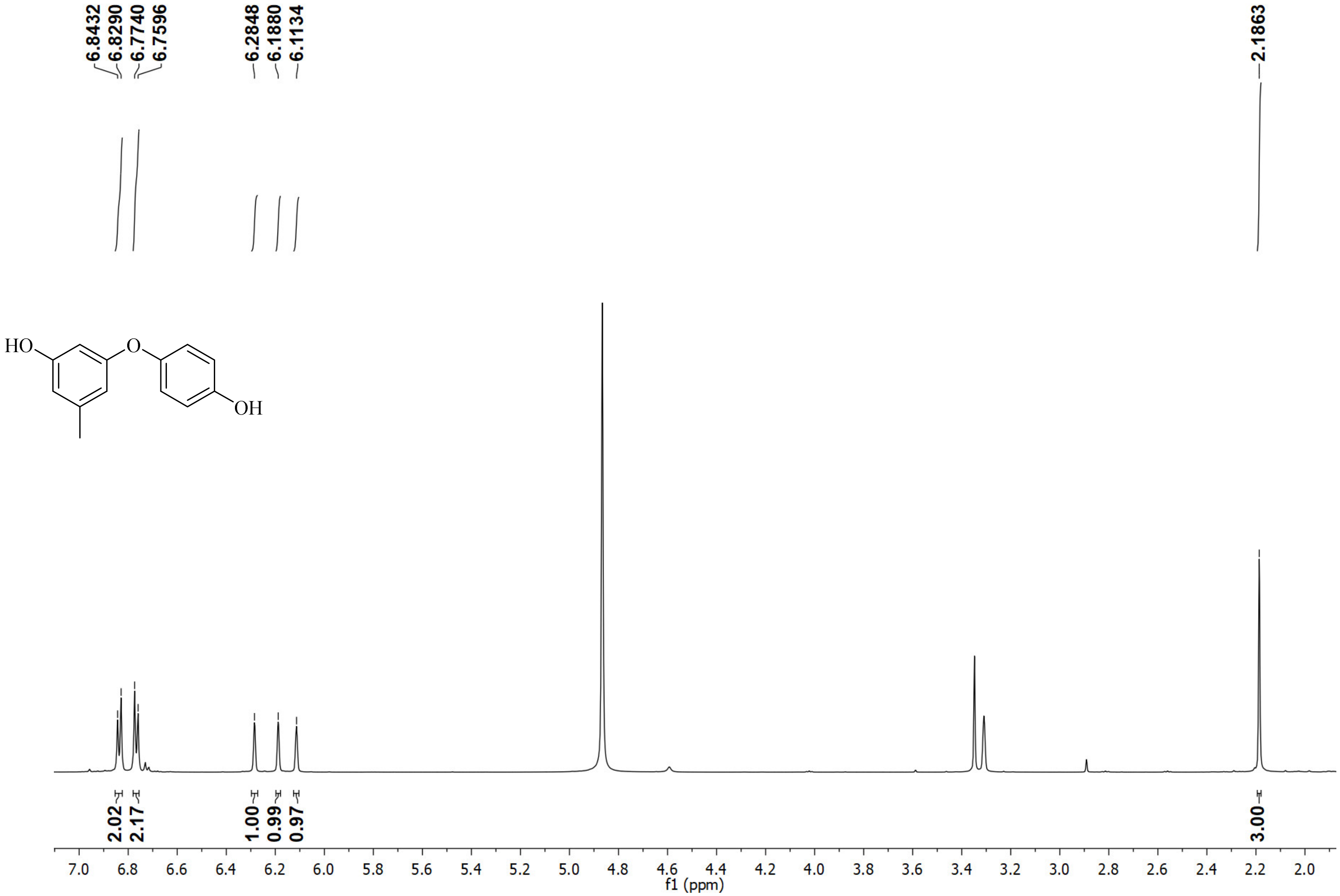

Figure S17.55. ^{1}H NMR spectrum of **12** in methanol-d_4.

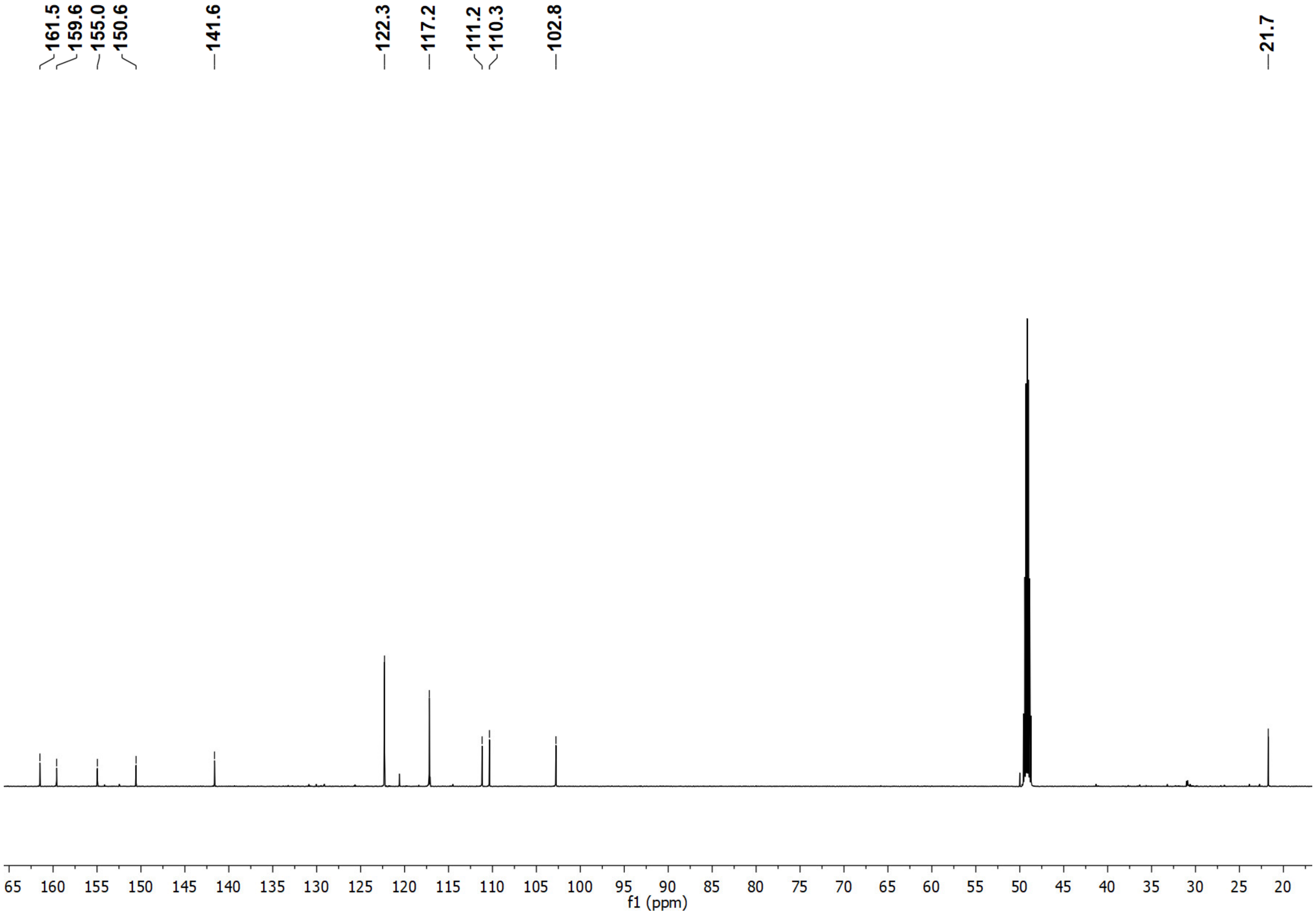

Figure S17.56. ^{13}C NMR spectrum of **12** in methanol-d_4.

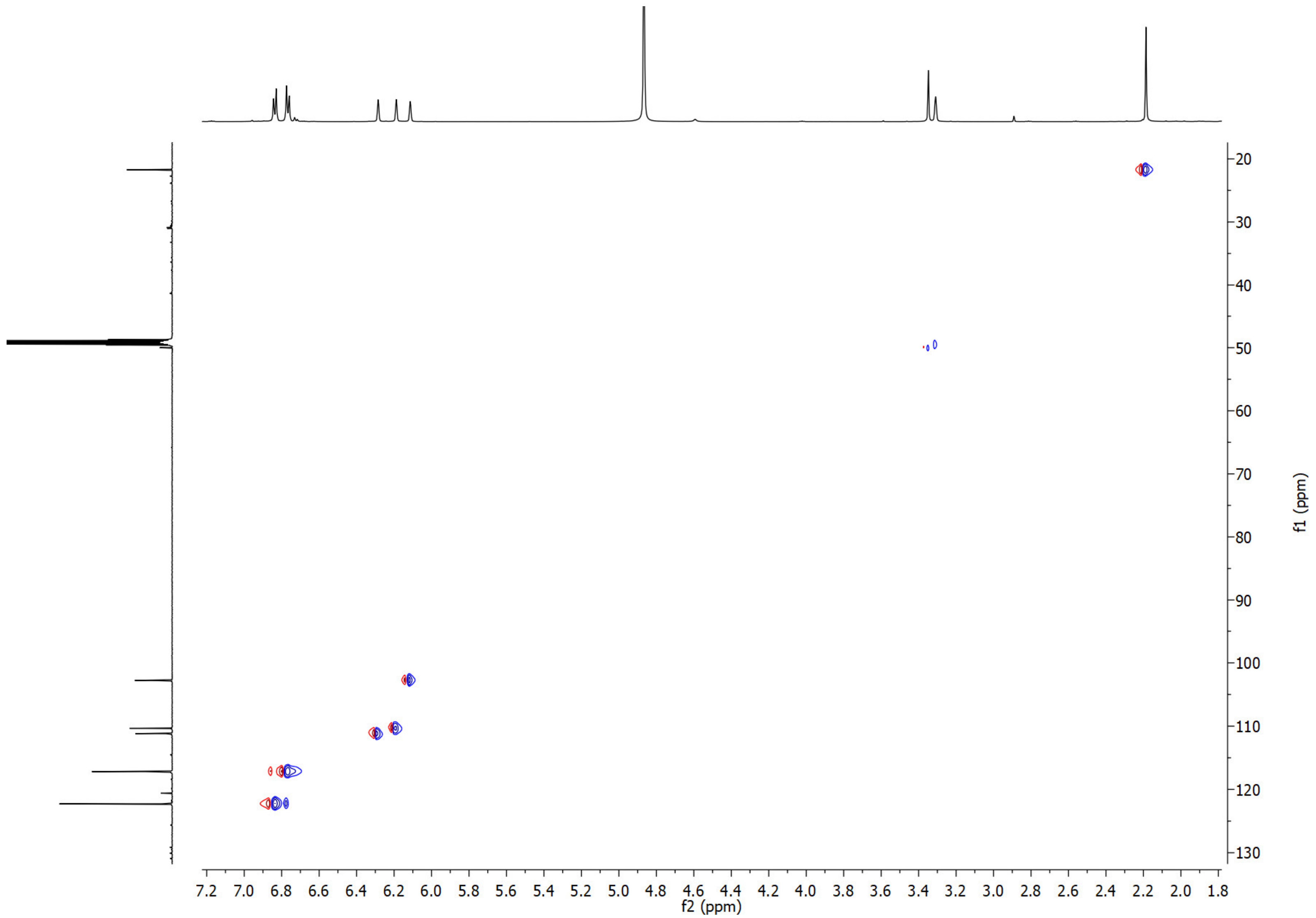

Figure S17.57. HSQC spectrum of **12** in methanol-d_4.

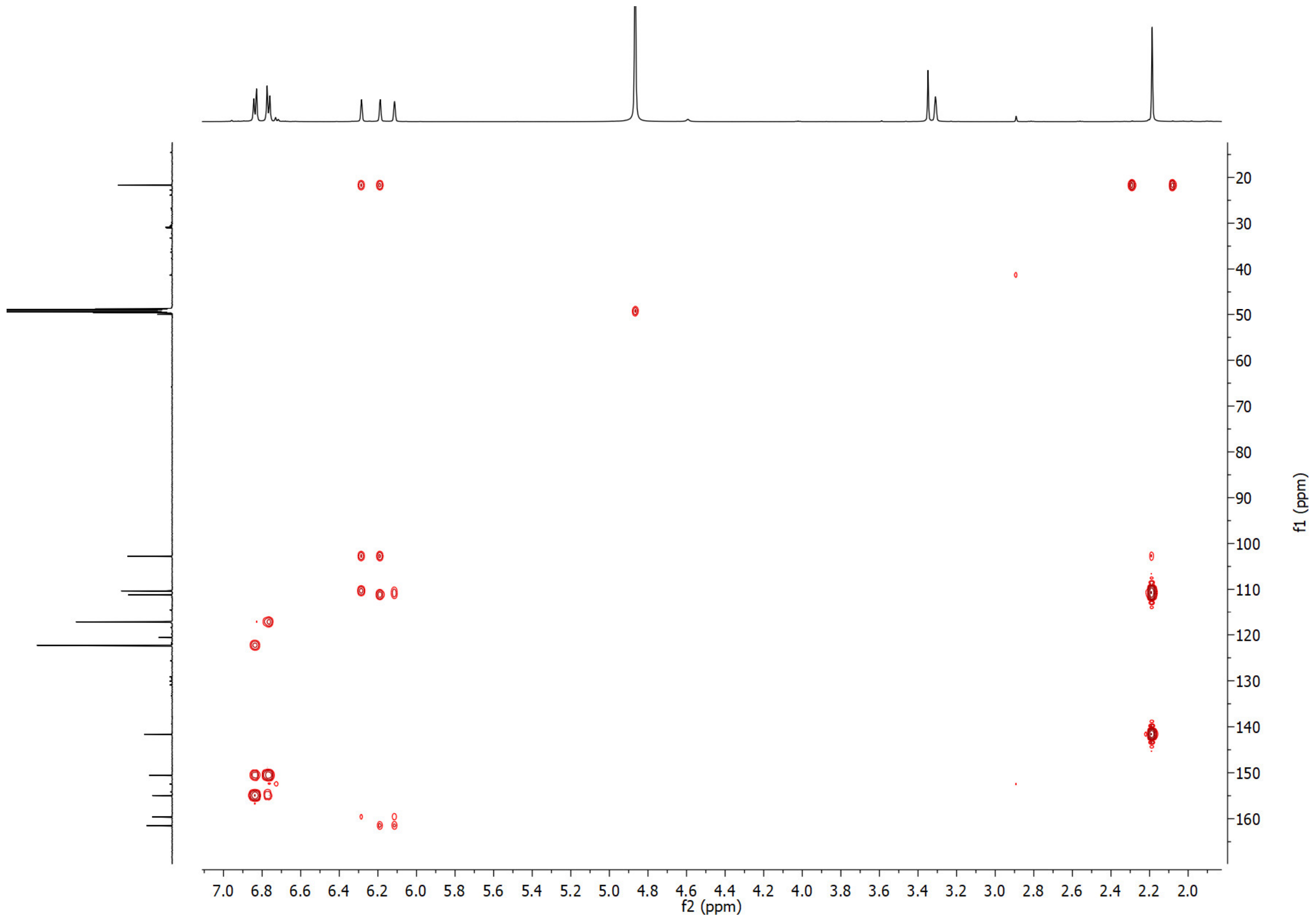

Figure S17.58. HMBC spectrum of **12** in methanol-d_4.

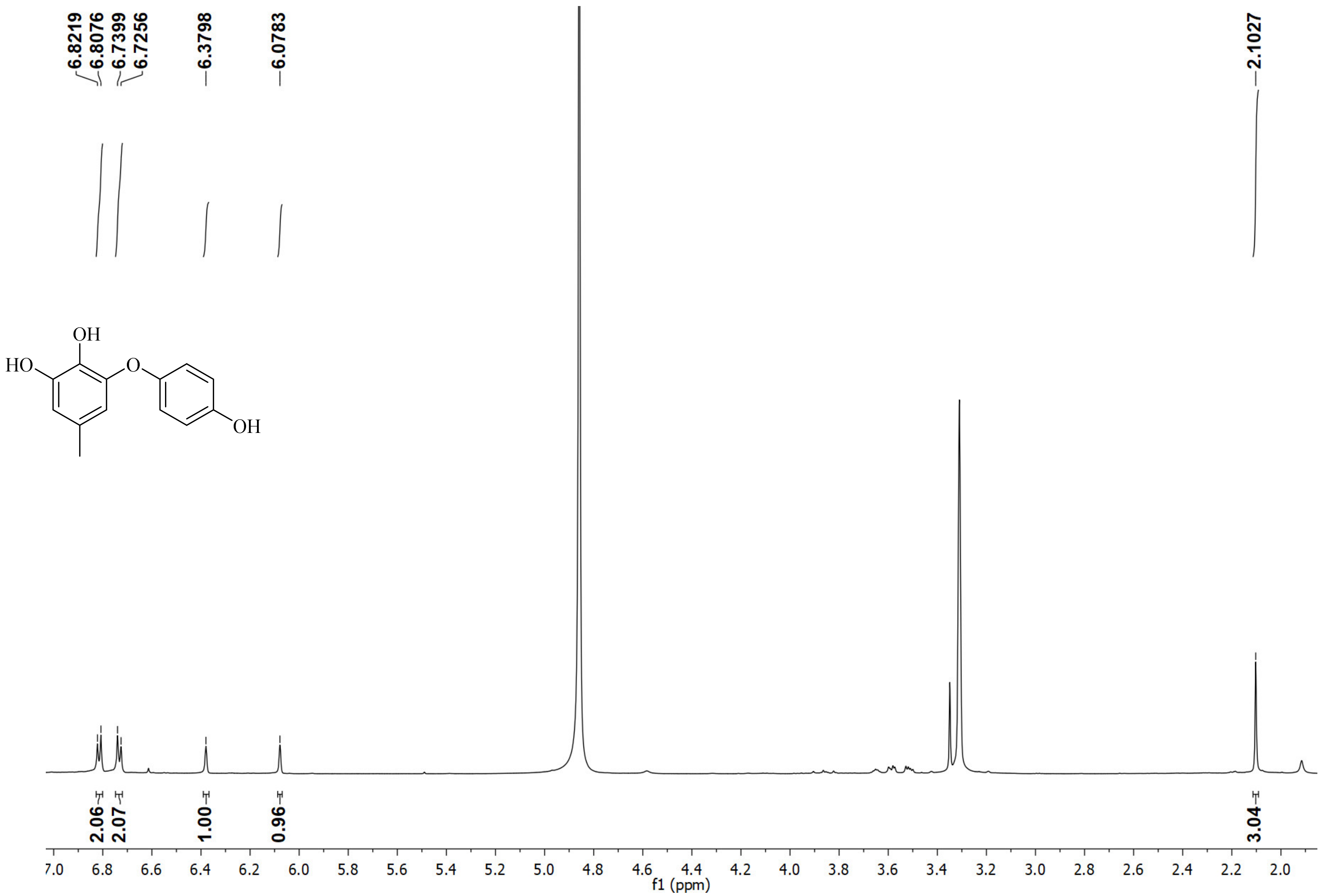

Figure S17.59. ^{1}H NMR spectrum of **12a** in methanol-d_4.

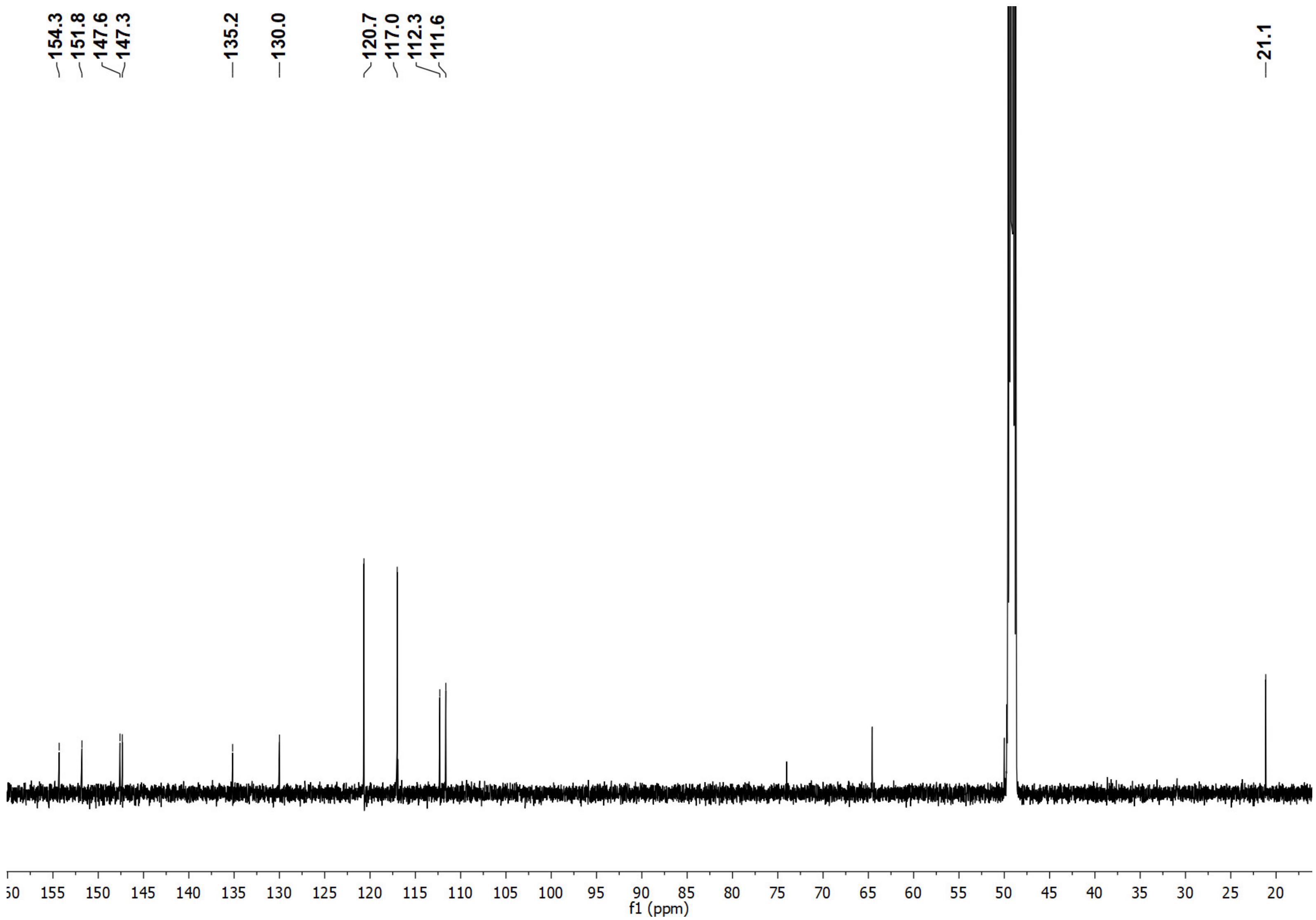

Figure S17.60. ^{13}C NMR spectrum of **12a** in methanol-d_4.

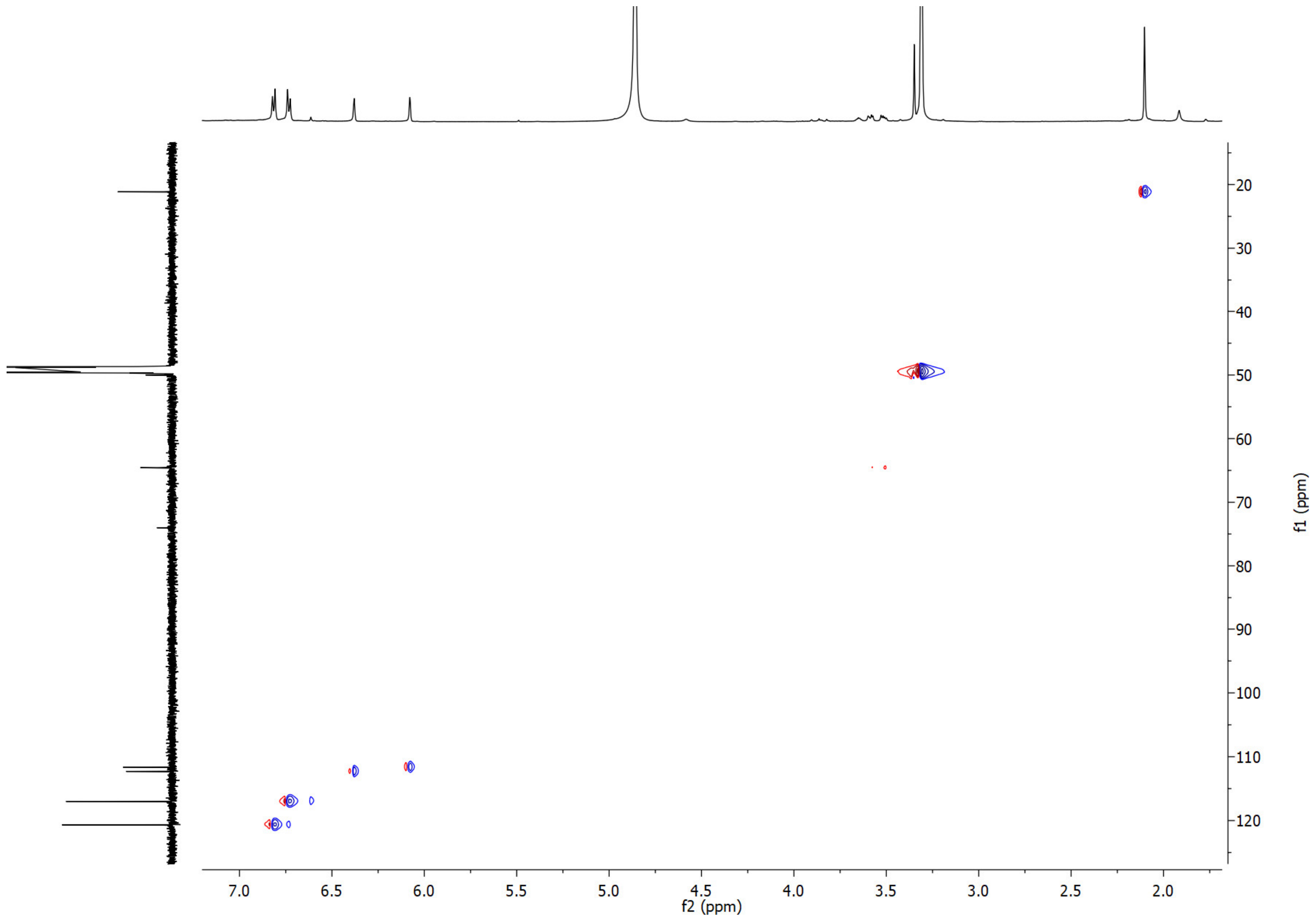

Figure S17.61. HSQC spectrum of **12a** in methanol-d_4.

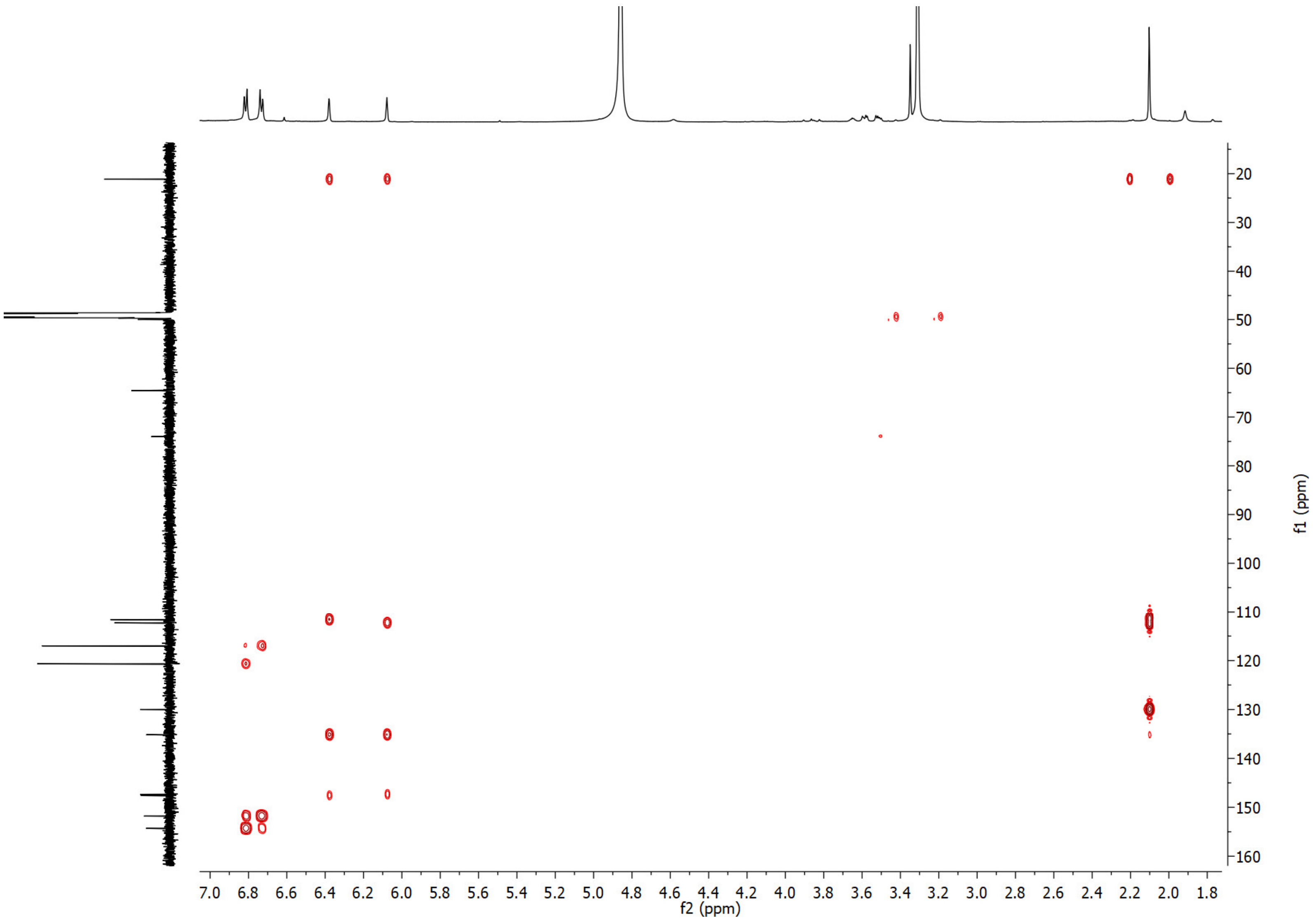

Figure S17.62. HMBC spectrum of **12a** in methanol-d_4.

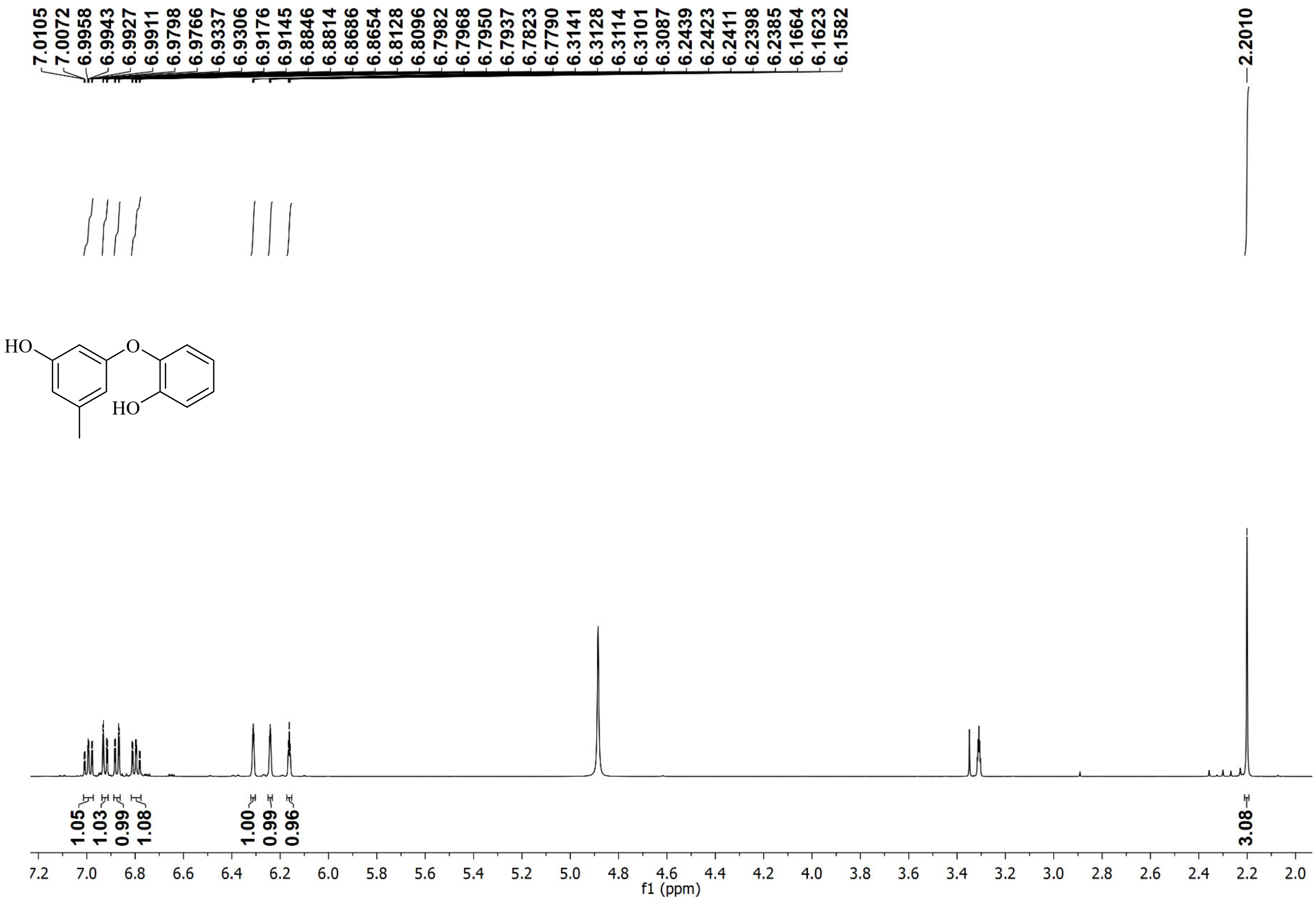

Figure S17.63. ^{1}H NMR spectrum of **13** in methanol-d_4.

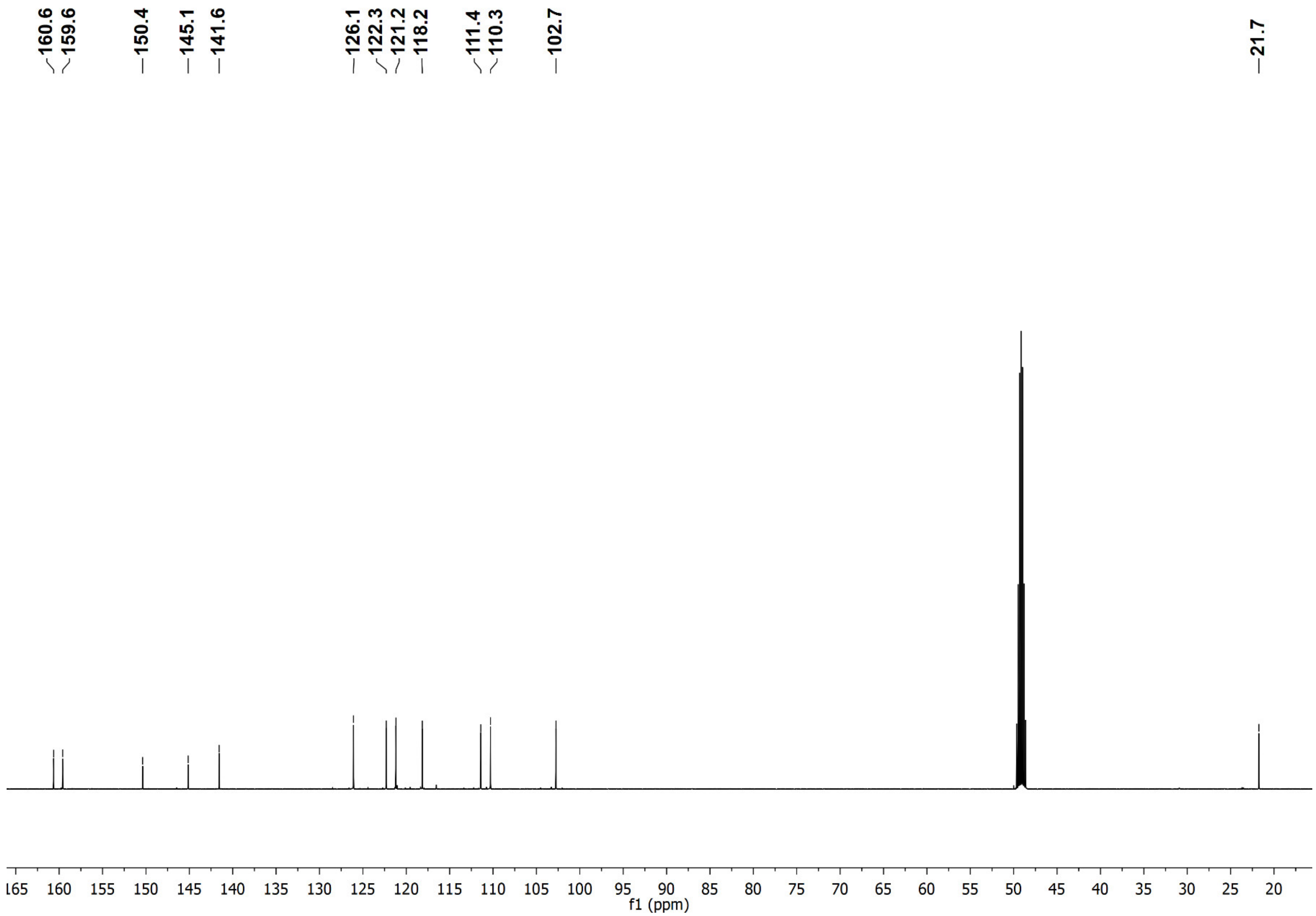

Figure S17.64. ^{13}C NMR spectrum of **13** in methanol-d_4.

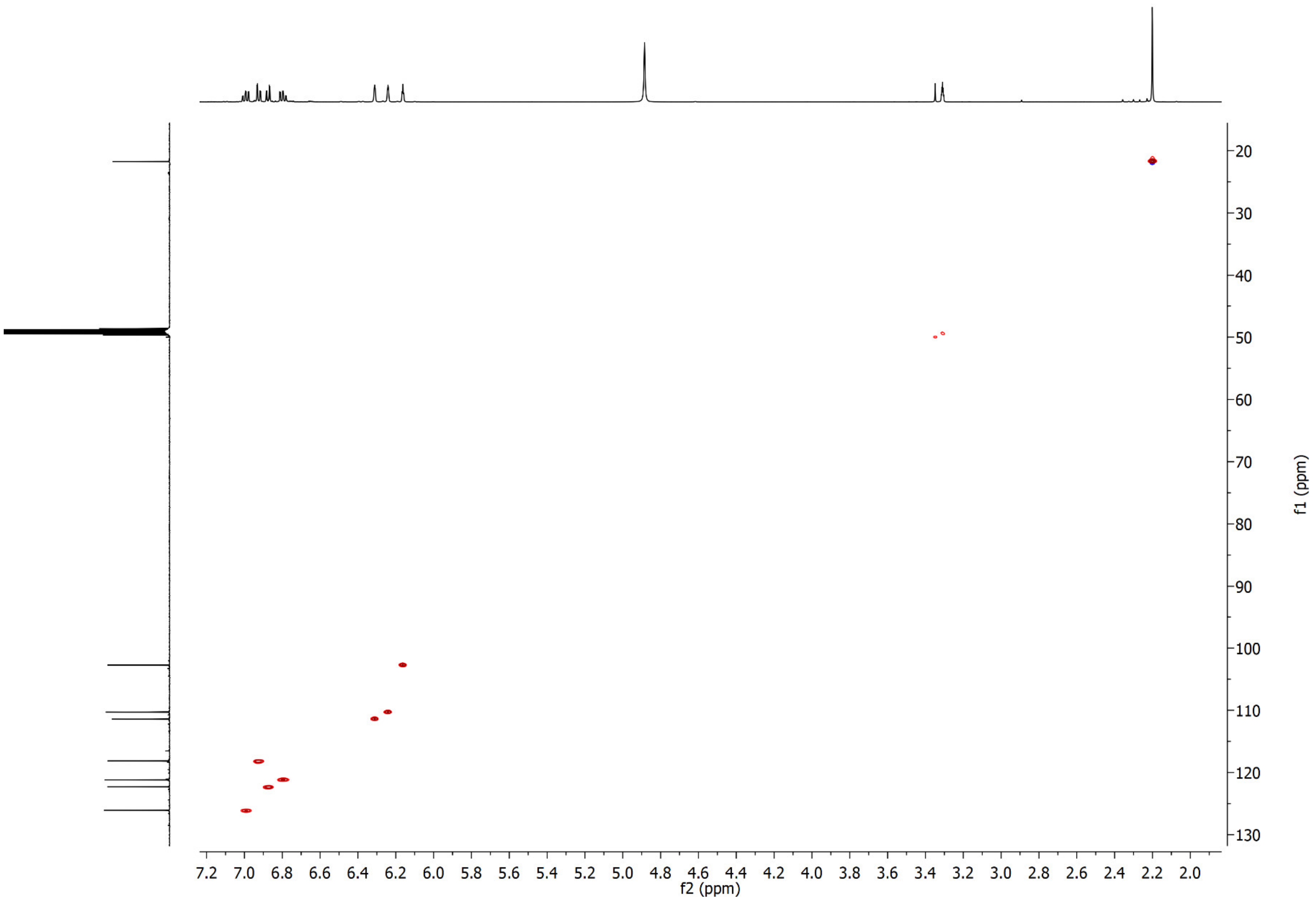

Figure S17.65. HSQC spectrum of **13** in methanol-*d*4.

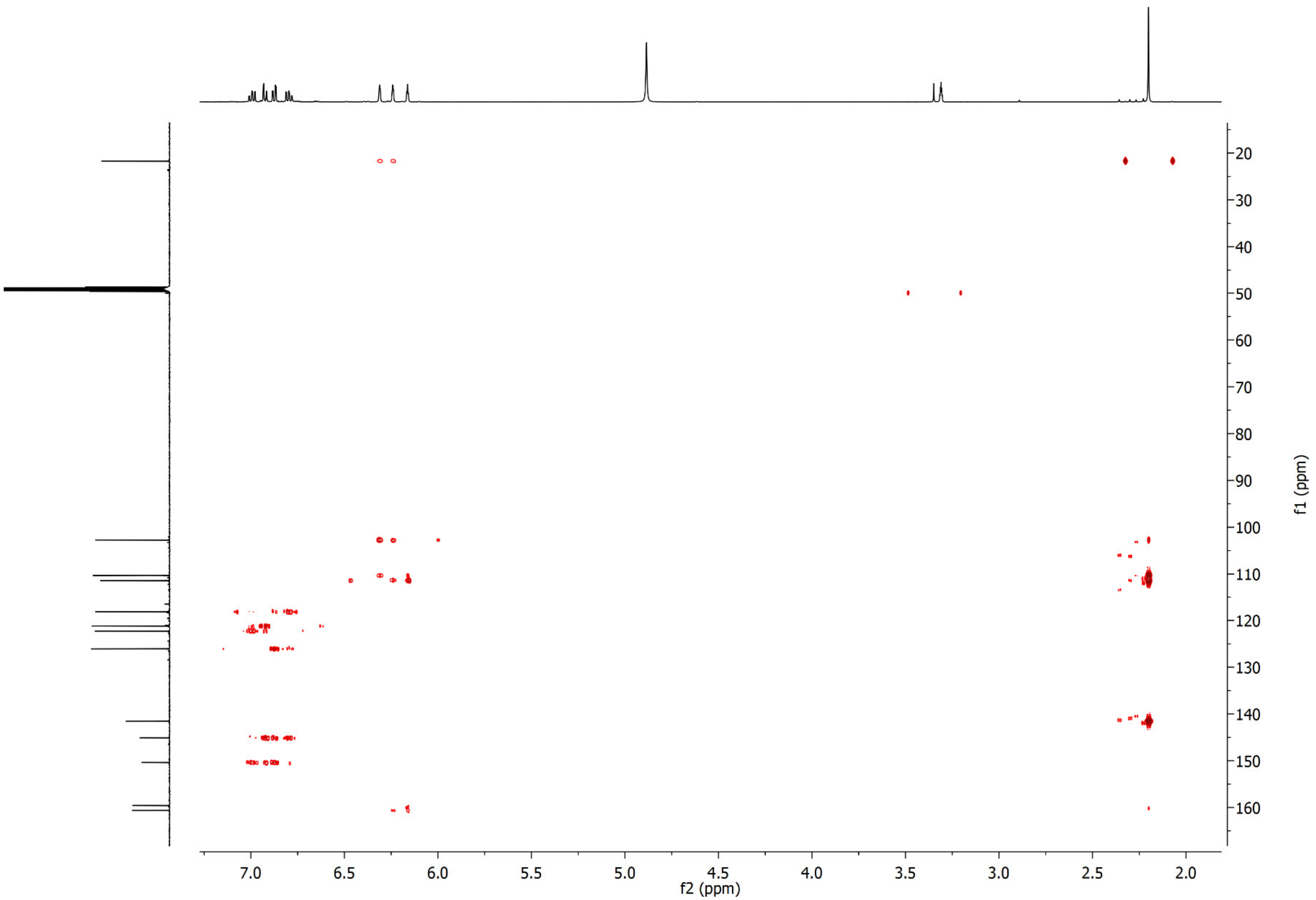

Figure S17.66. HMBC spectrum of **13** in methanol-d_4.

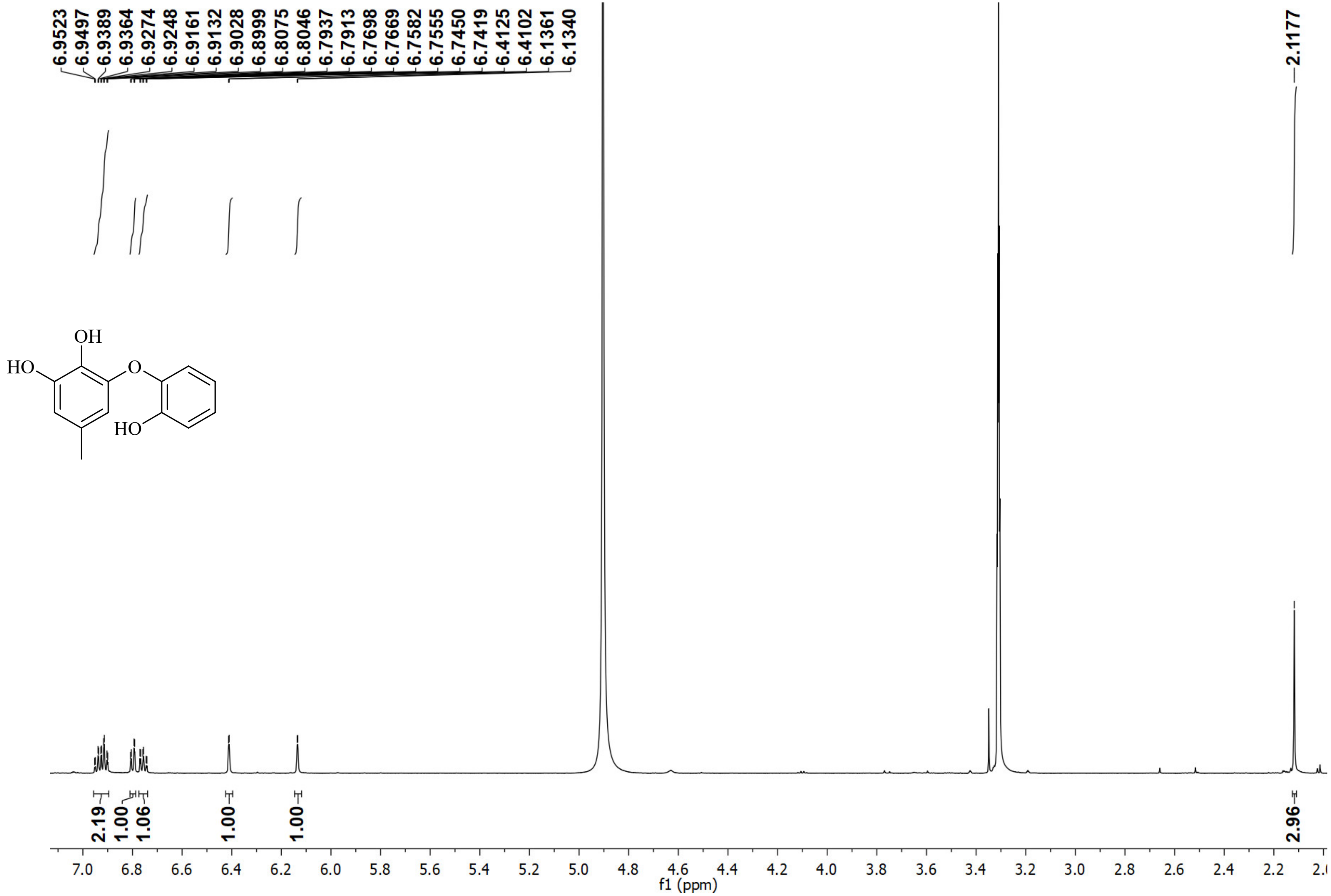

Figure S17.67. ^{1}H NMR spectrum of **13a** in methanol-d_4.

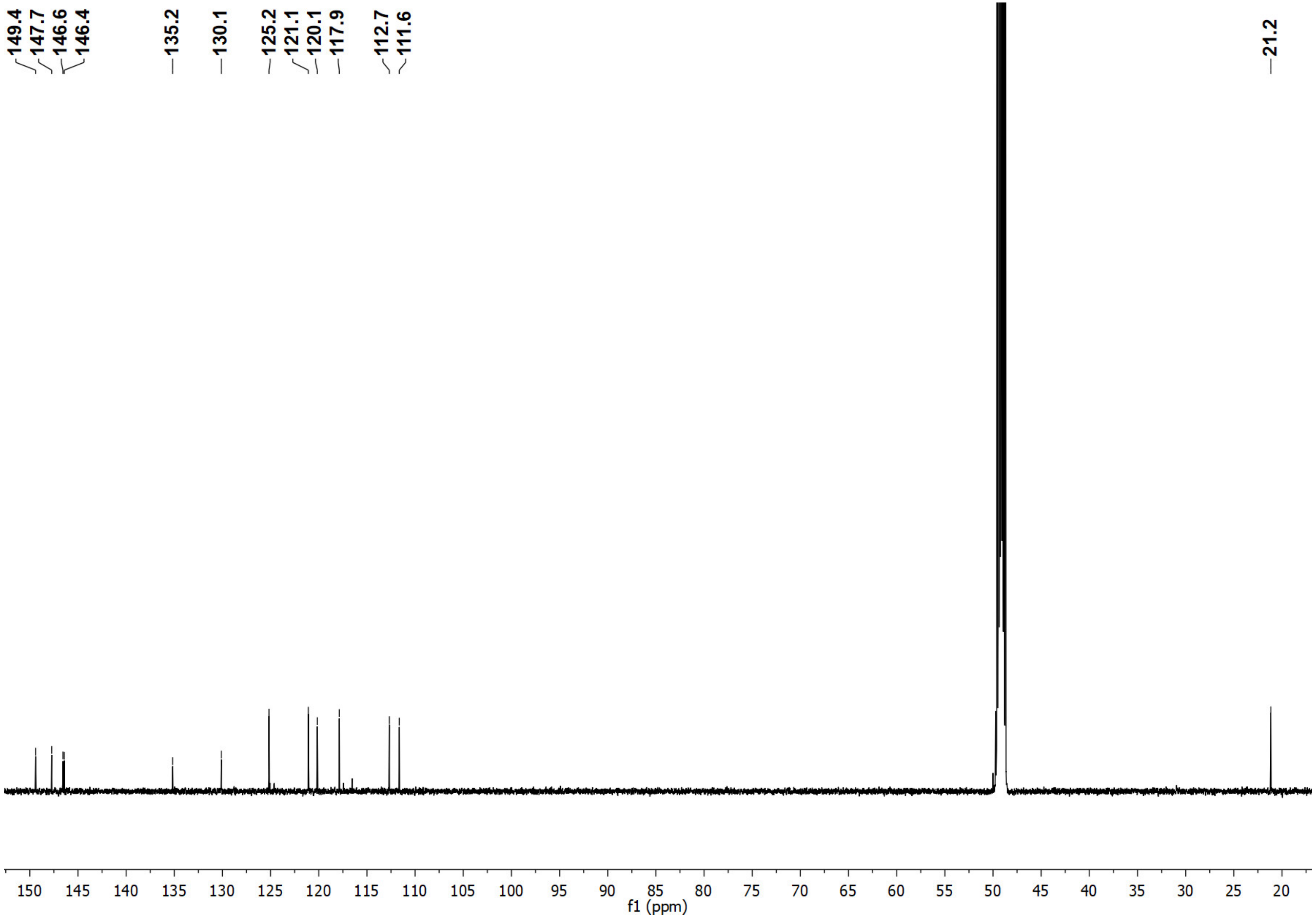

Figure S17.68. ^{13}C NMR spectrum of **13a** in methanol-d_4.

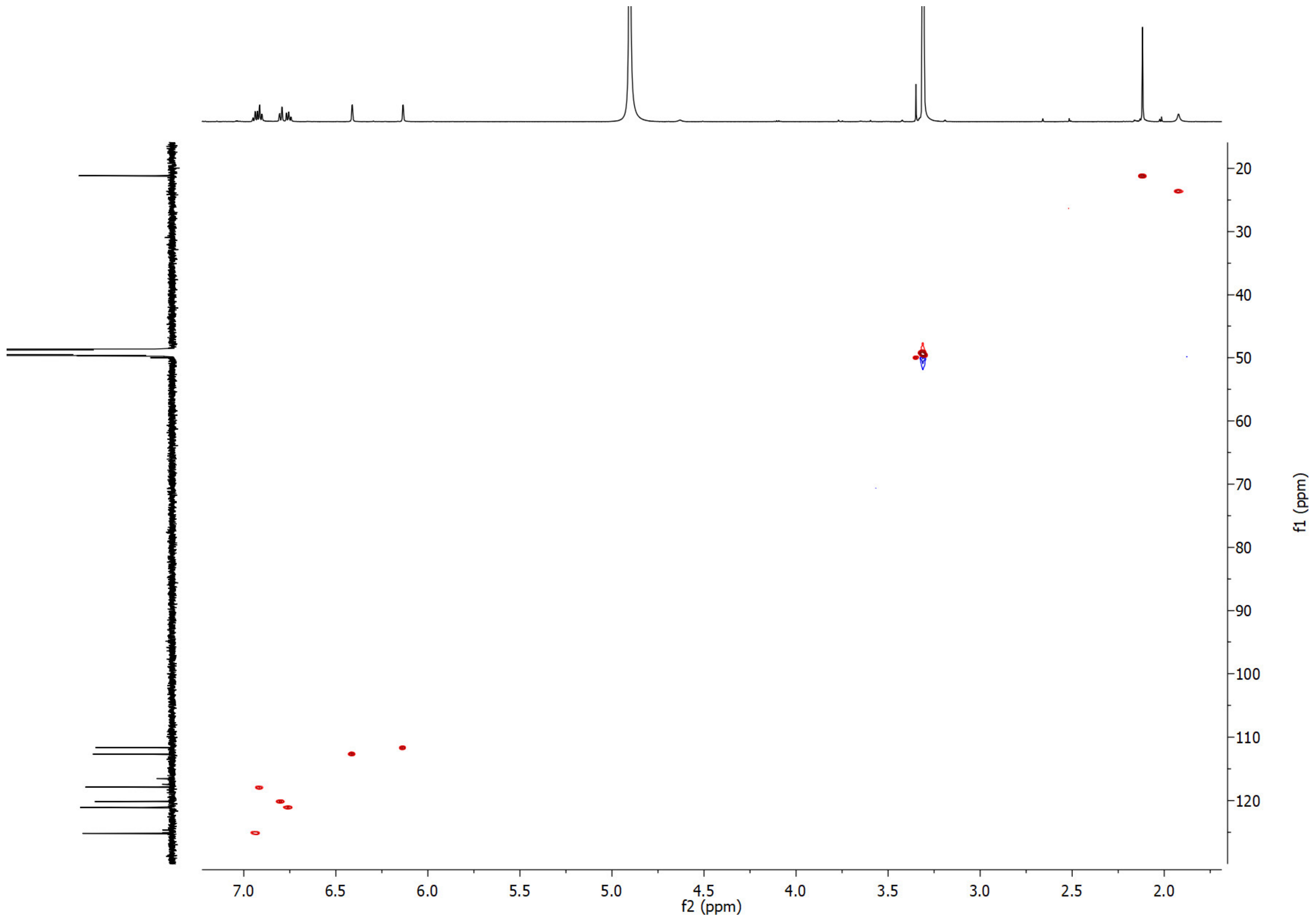

Figure S17.69. HSQC spectrum of **13a** in methanol-d_4.

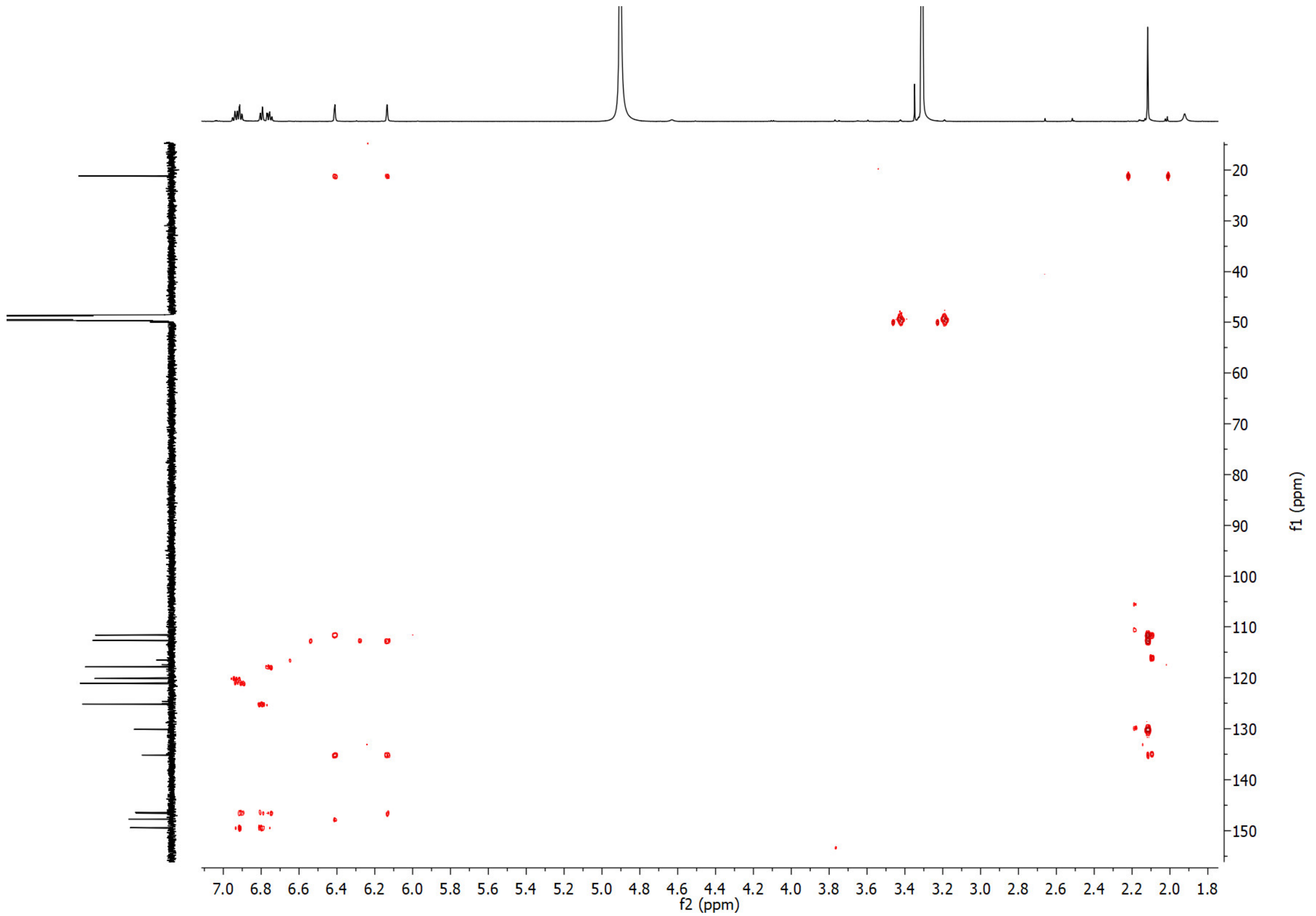

Figure S17.70. HMBC spectrum of **13a** in methanol-d_4.

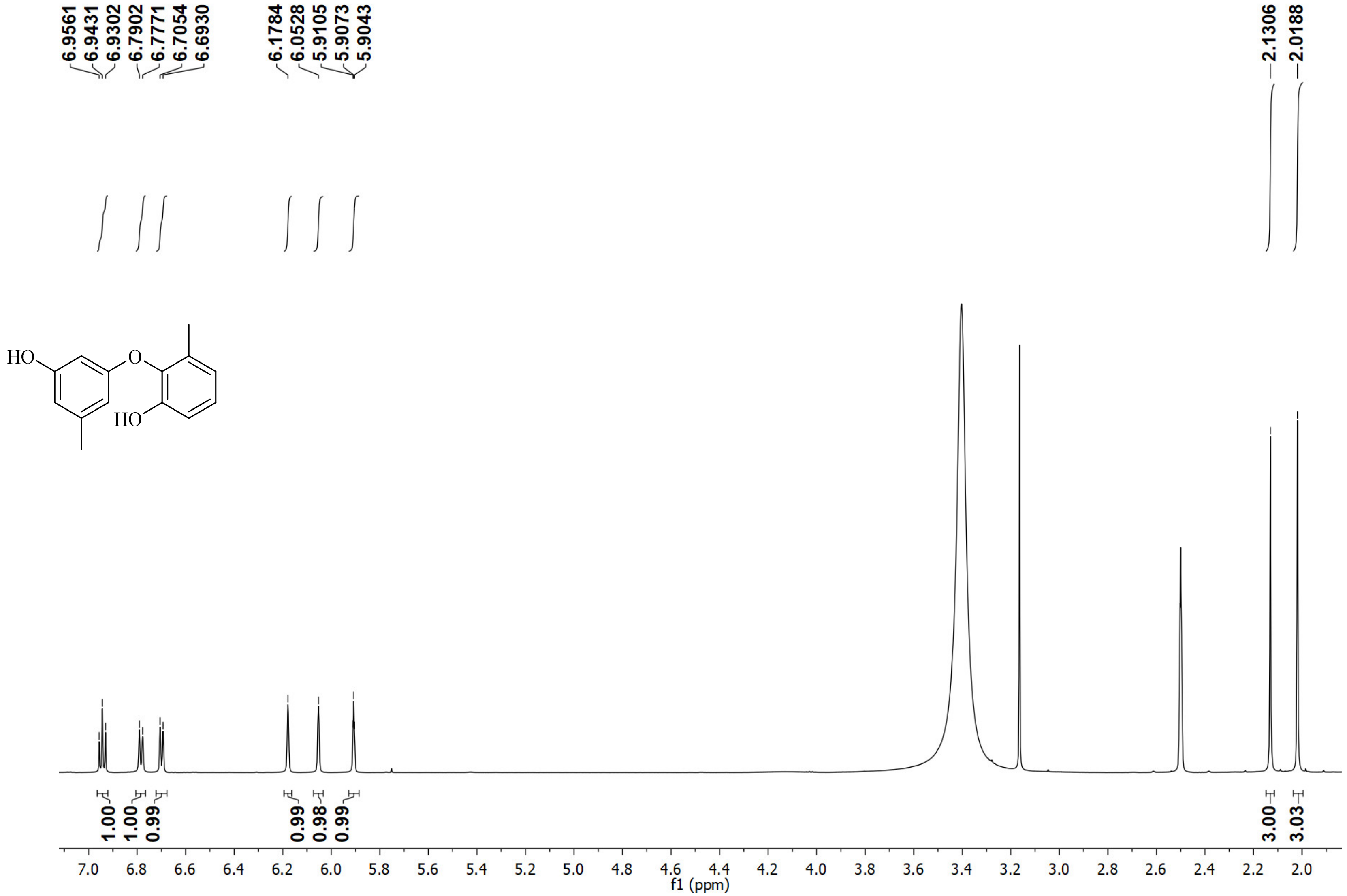

Figure S17.71. ^{1}H NMR spectrum of **14** in DMSO-d_6.

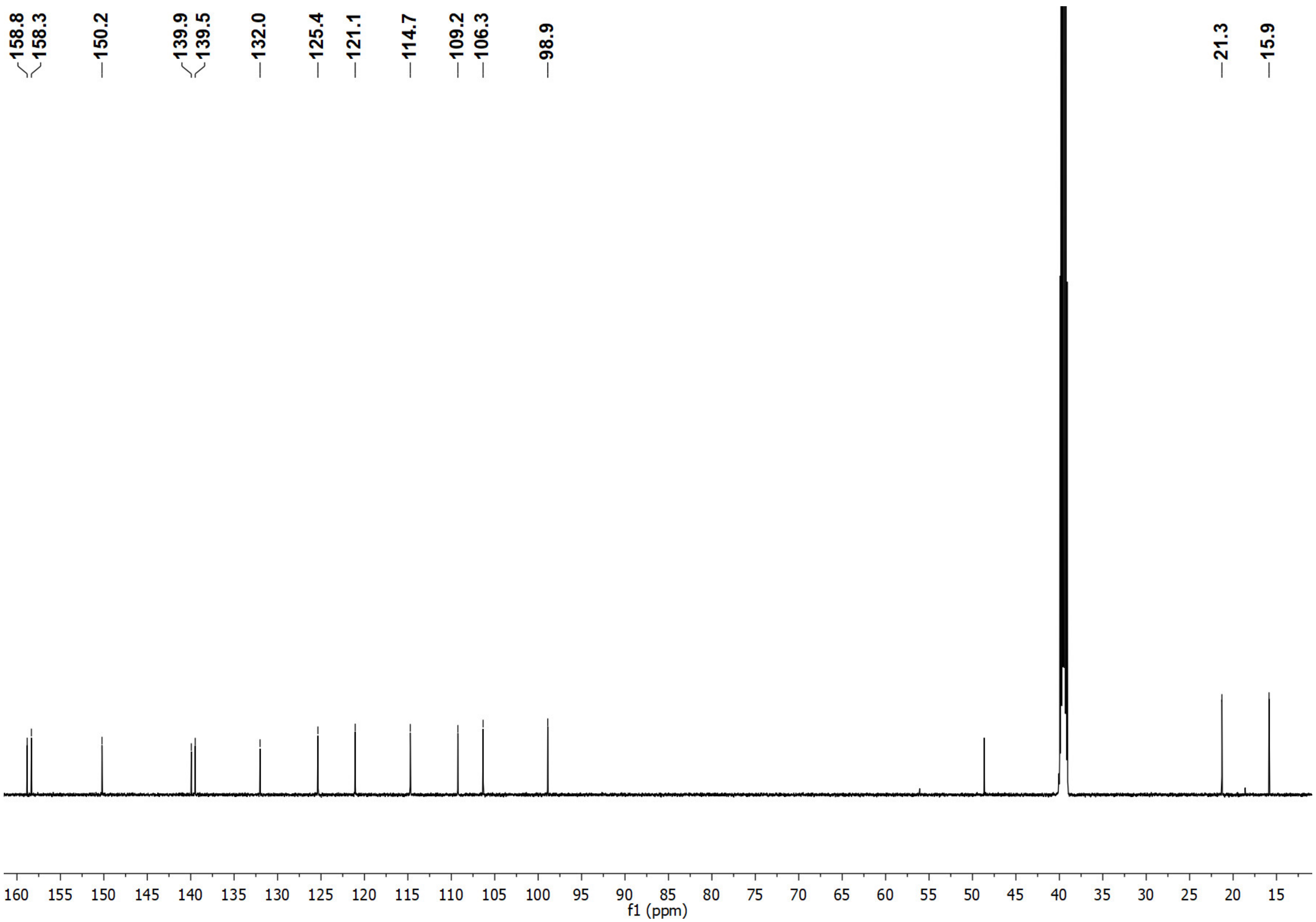

Figure S17.72. ^{13}C NMR spectrum of **14** in DMSO-d_6.

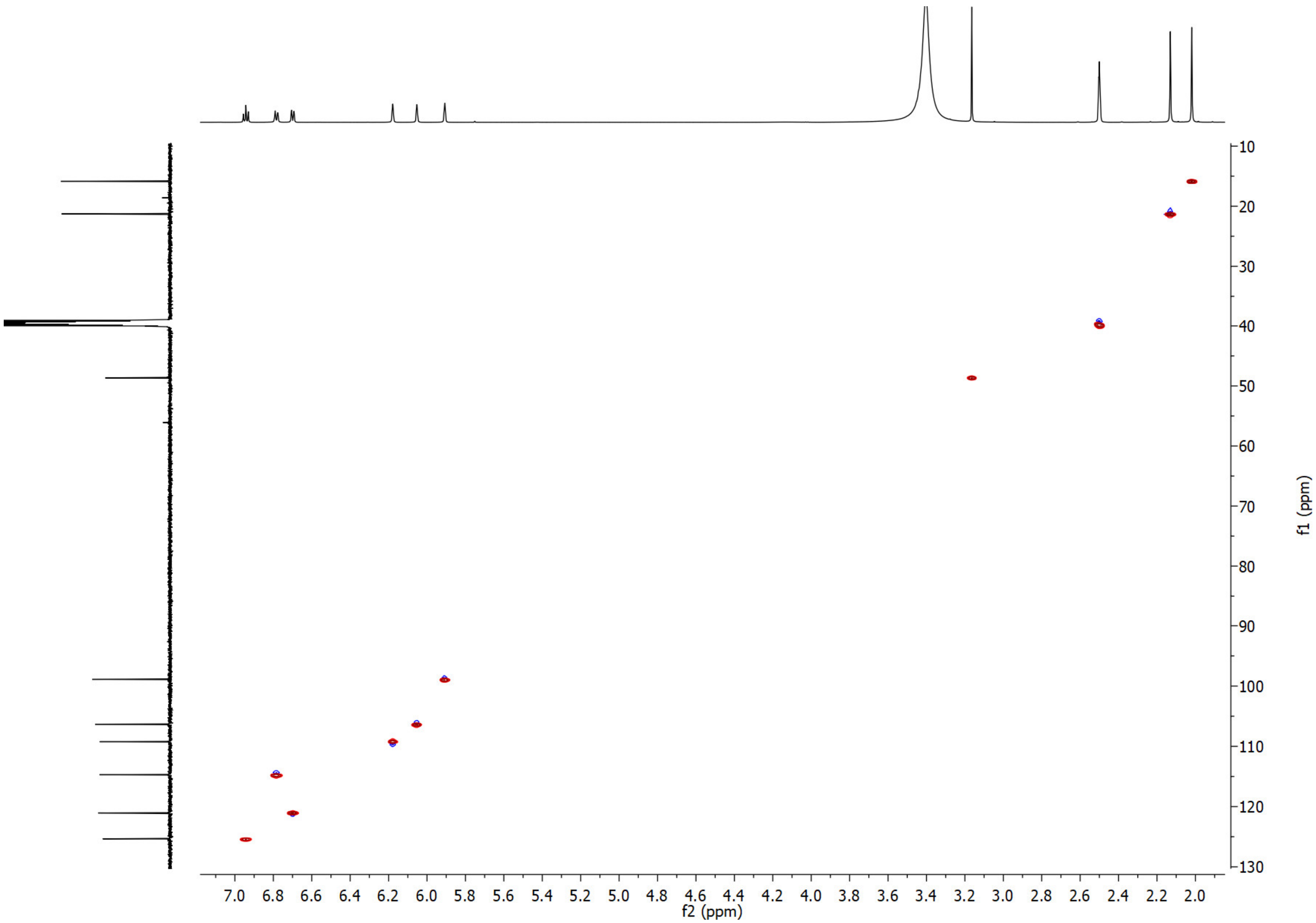

Figure S17.73. HSQC spectrum of **14** in DMSO-d_6.

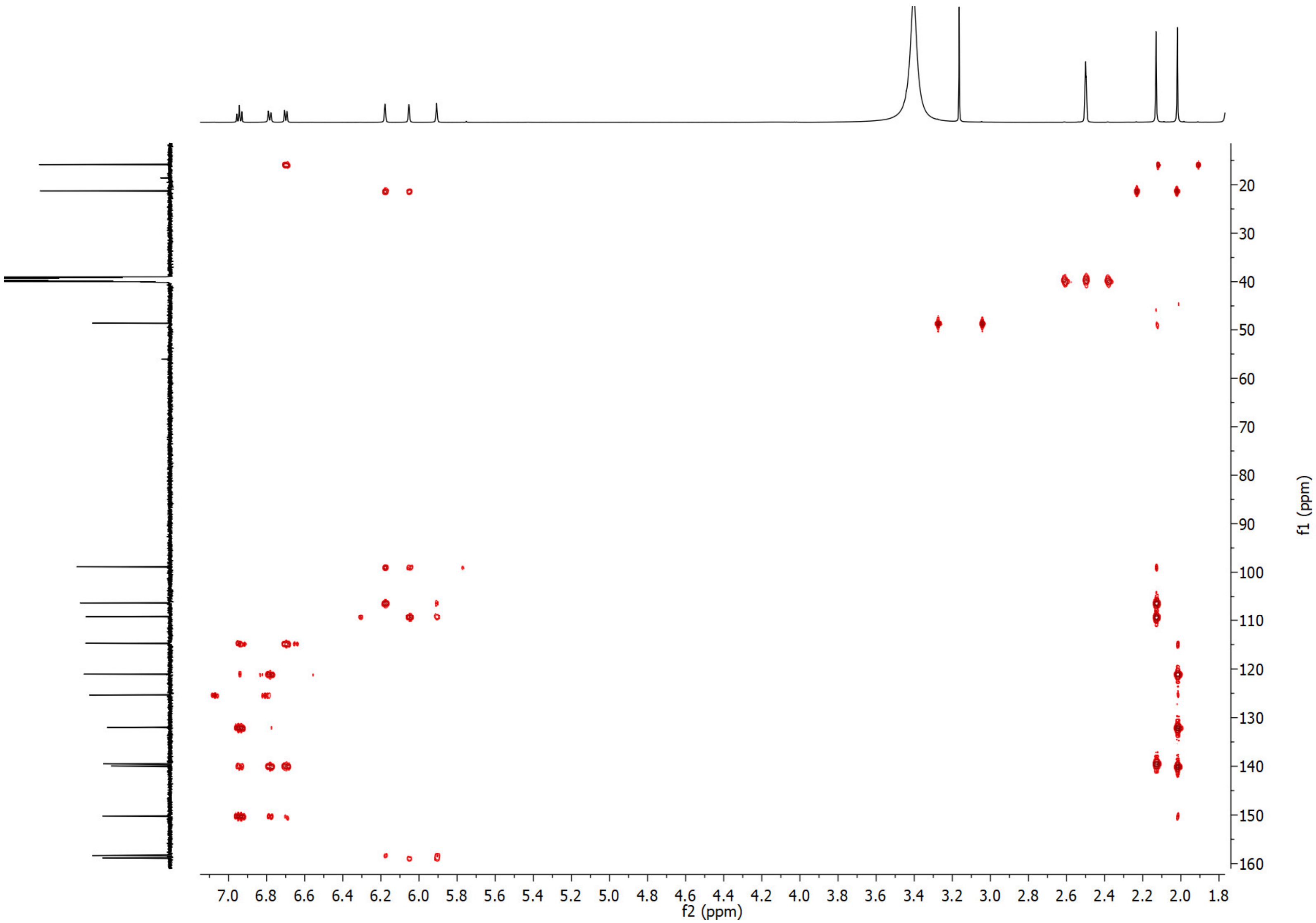

Figure S17.74. HMBC spectrum of **14** in DMSO-d_6.

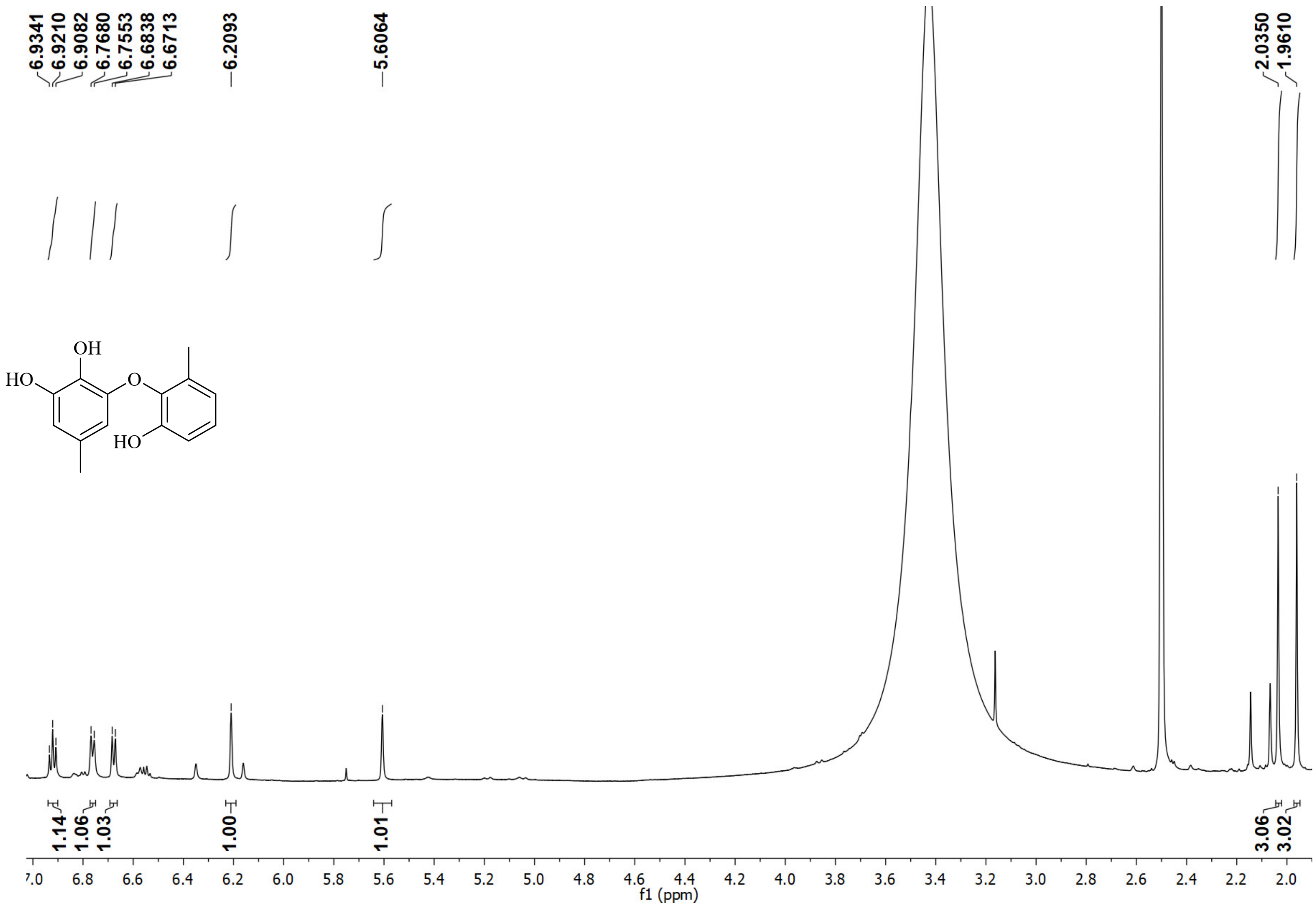

Figure S17.75. ^{1}H NMR spectrum of **14a** in DMSO-d_6.

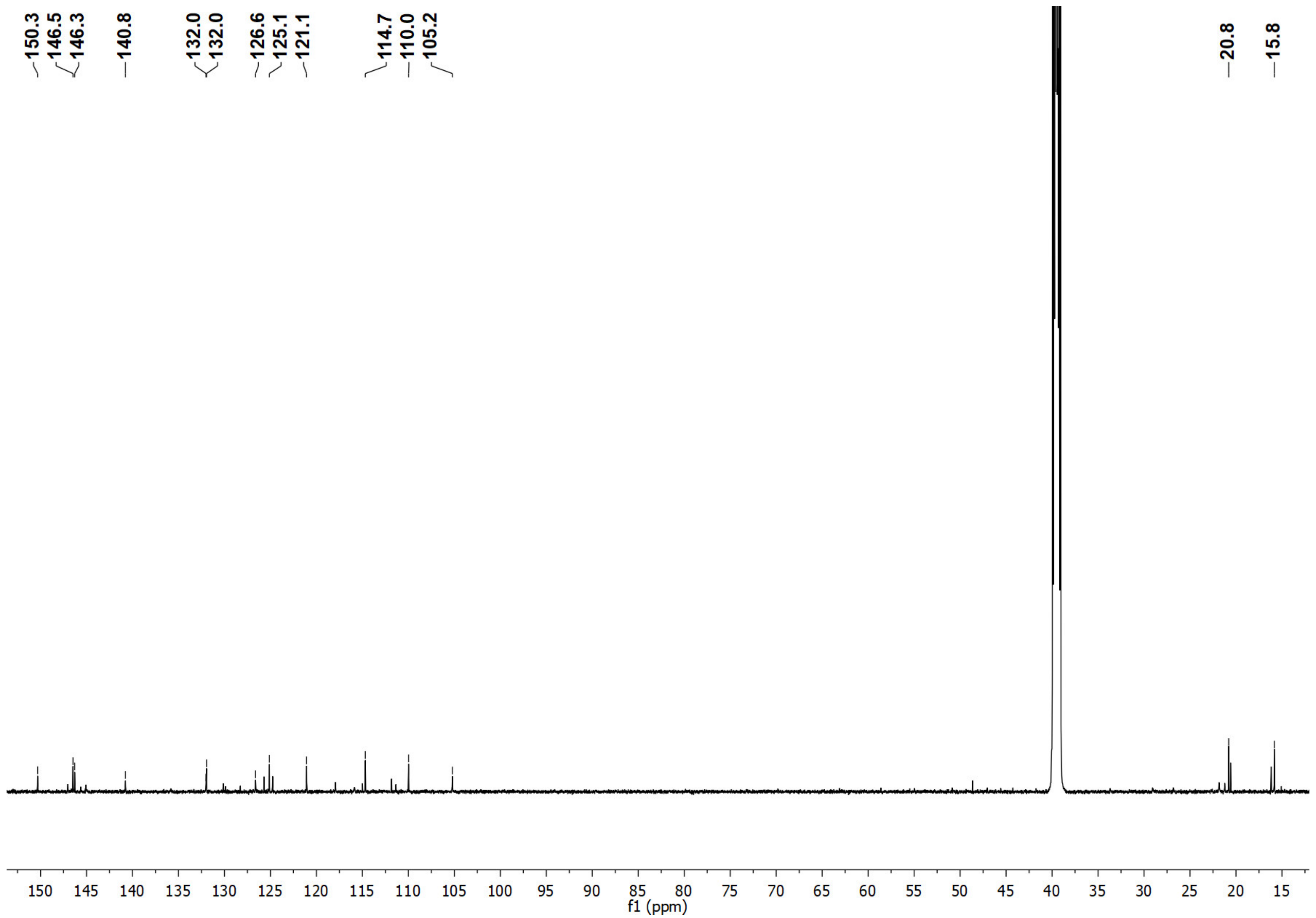

Figure S17.76. ^{13}C NMR spectrum of **14a** in DMSO-d_6.

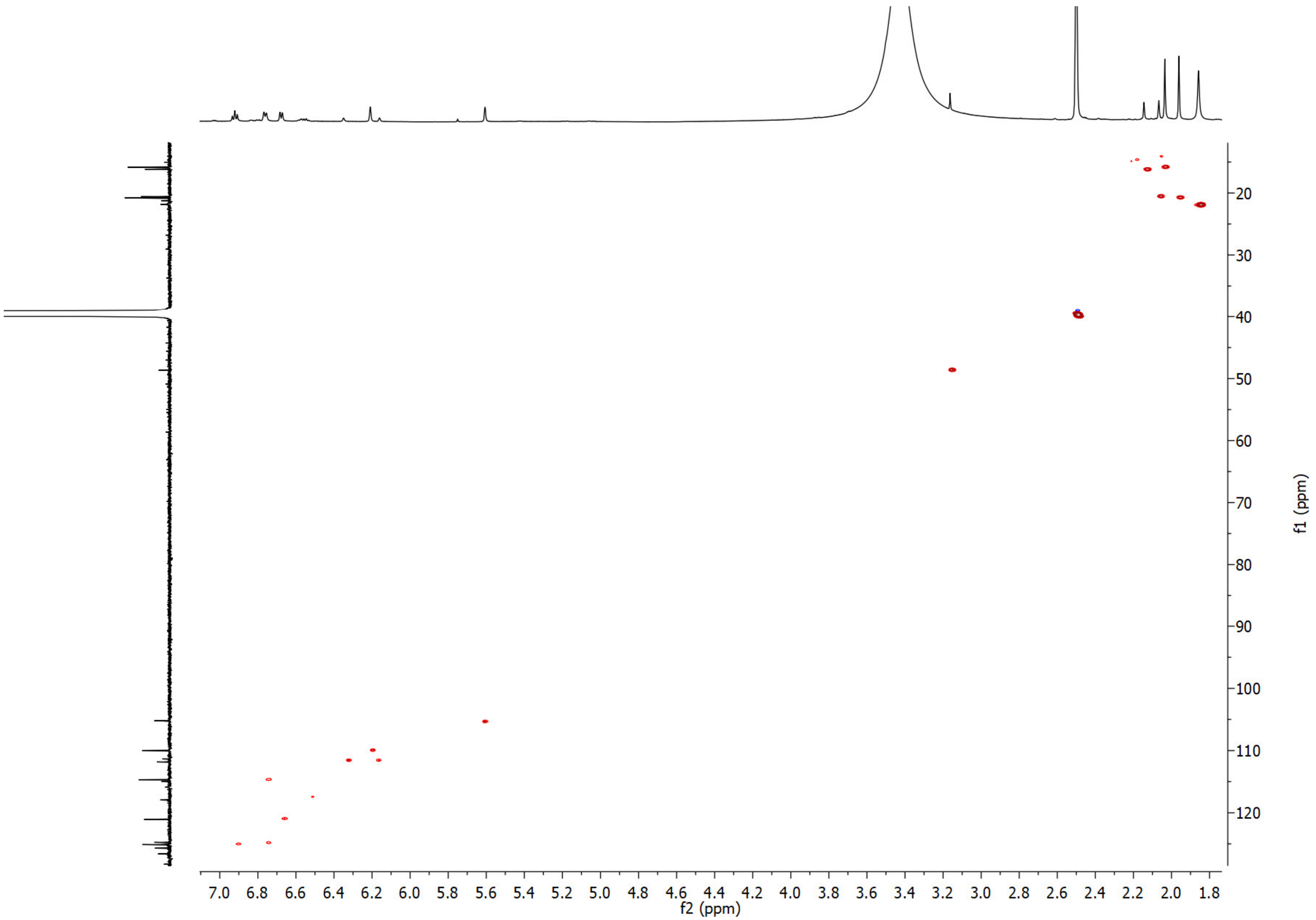

Figure S17.77. HSQC spectrum of **14a** in DMSO-d_6.

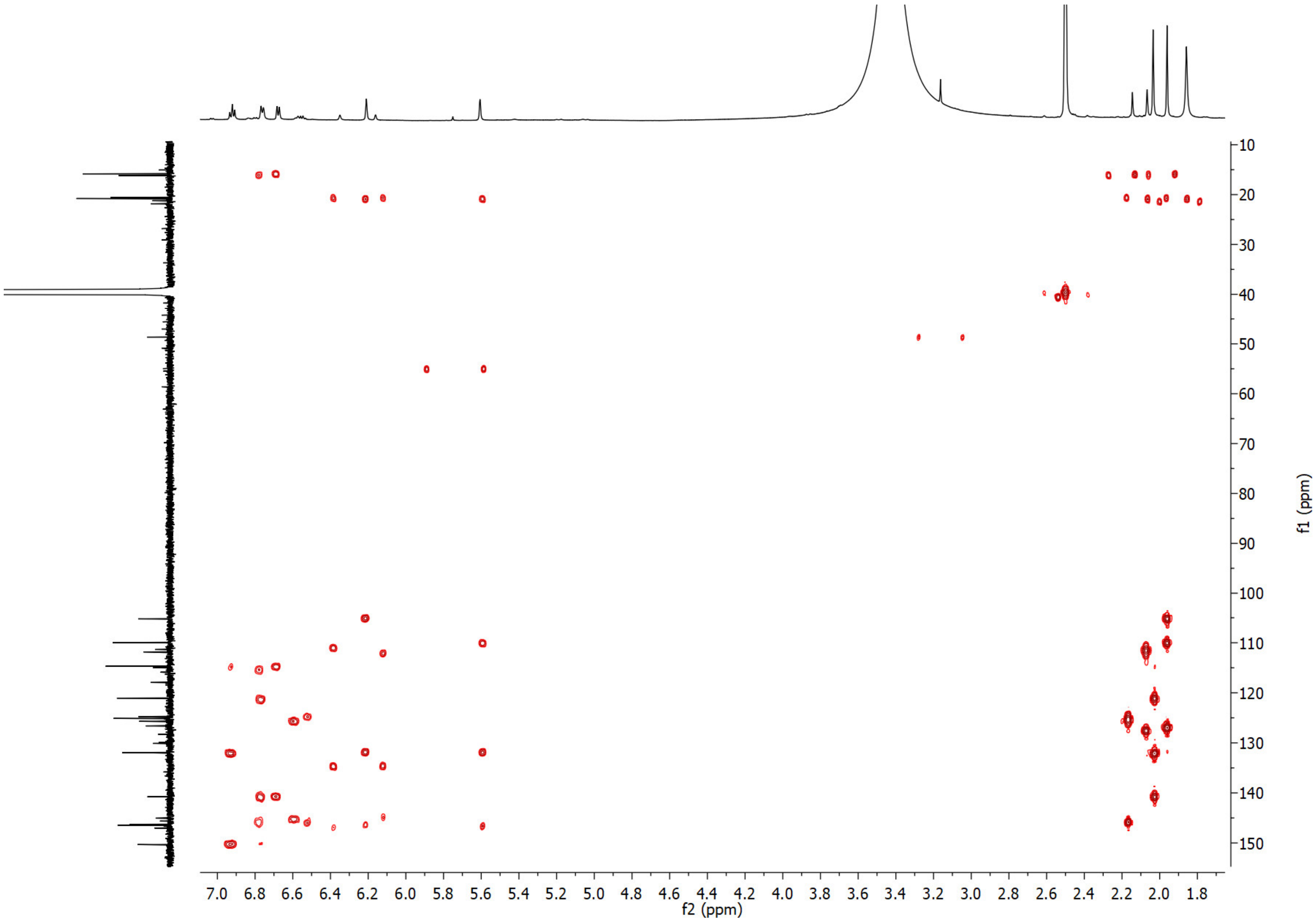

Figure S17.78. HMBC spectrum of **14a** in DMSO-d_6.

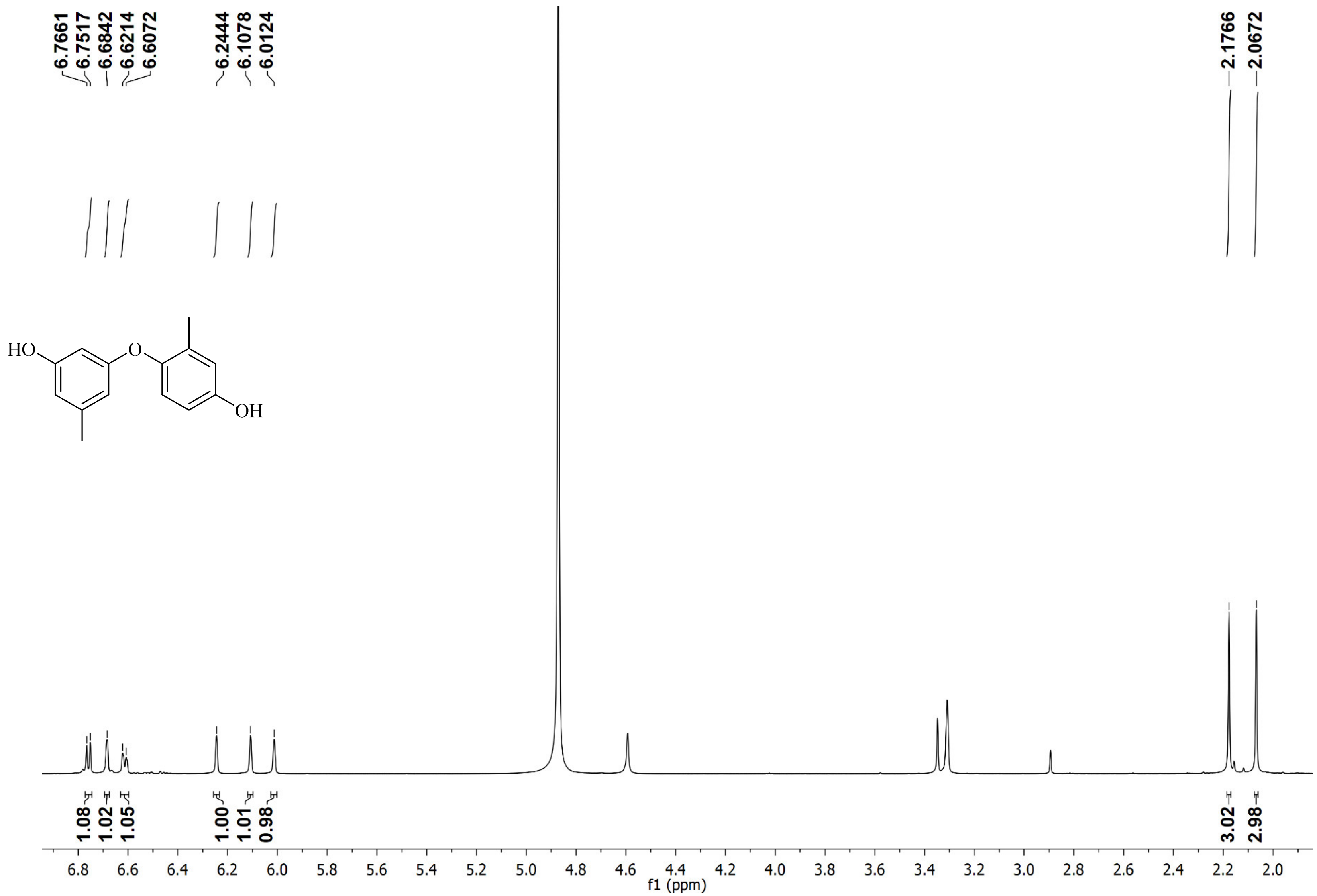

Figure S17.79. ^{1}H NMR spectrum of **15** in methanol-d_4.

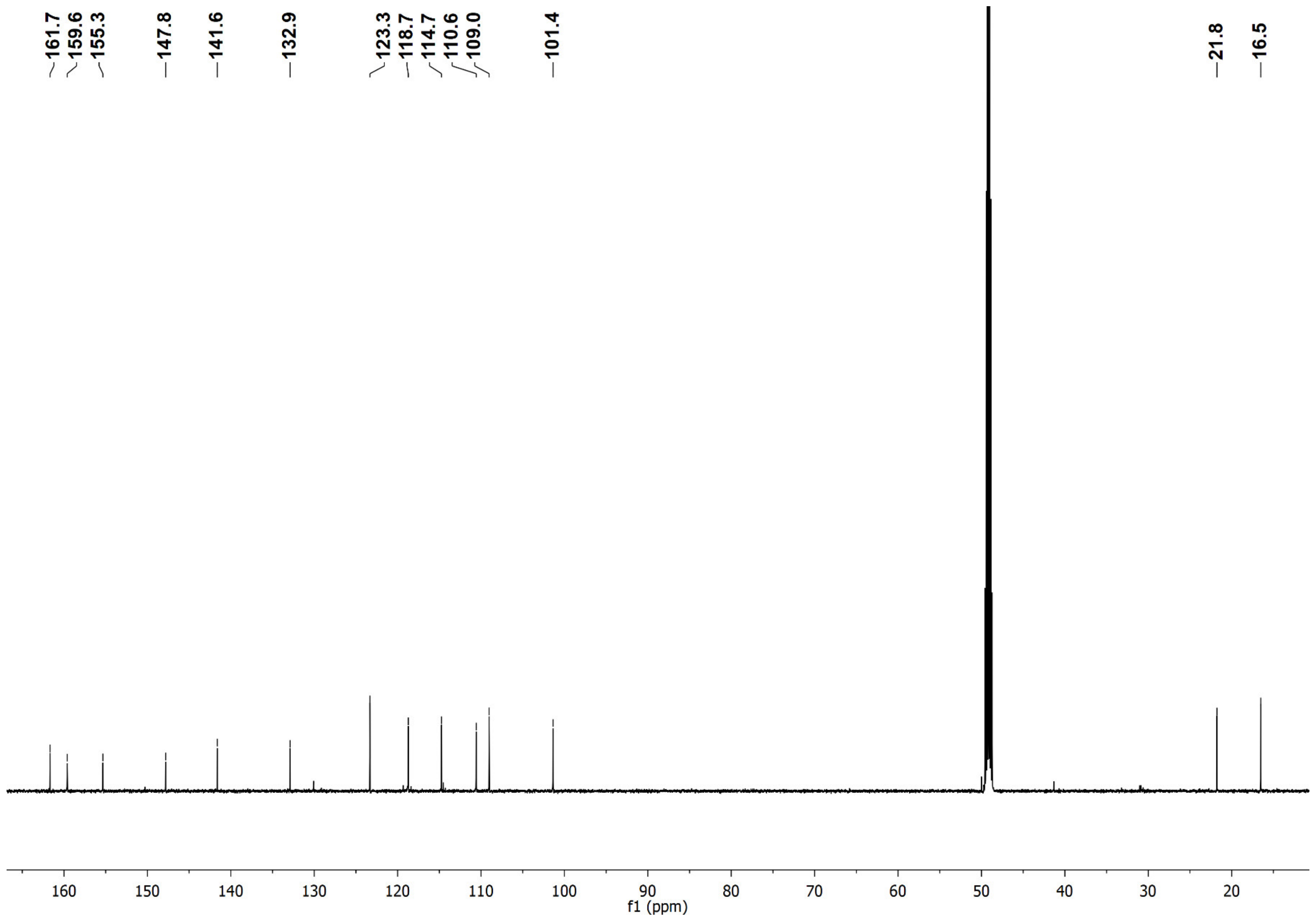

Figure S17.80. ^{13}C NMR spectrum of **15** in methanol-d_4.

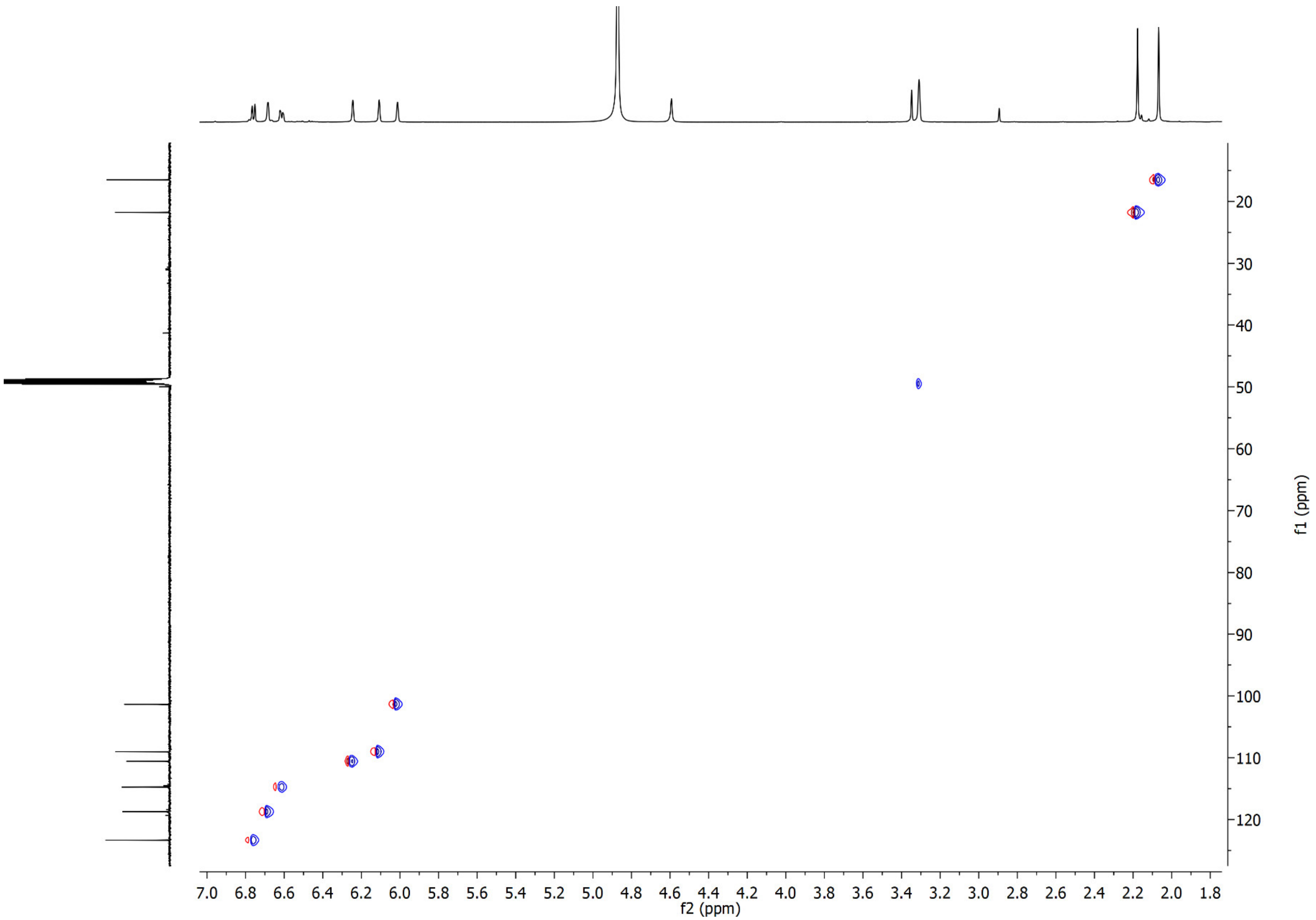

Figure S17.81. HSQC spectrum of **15** in methanol-d_4.

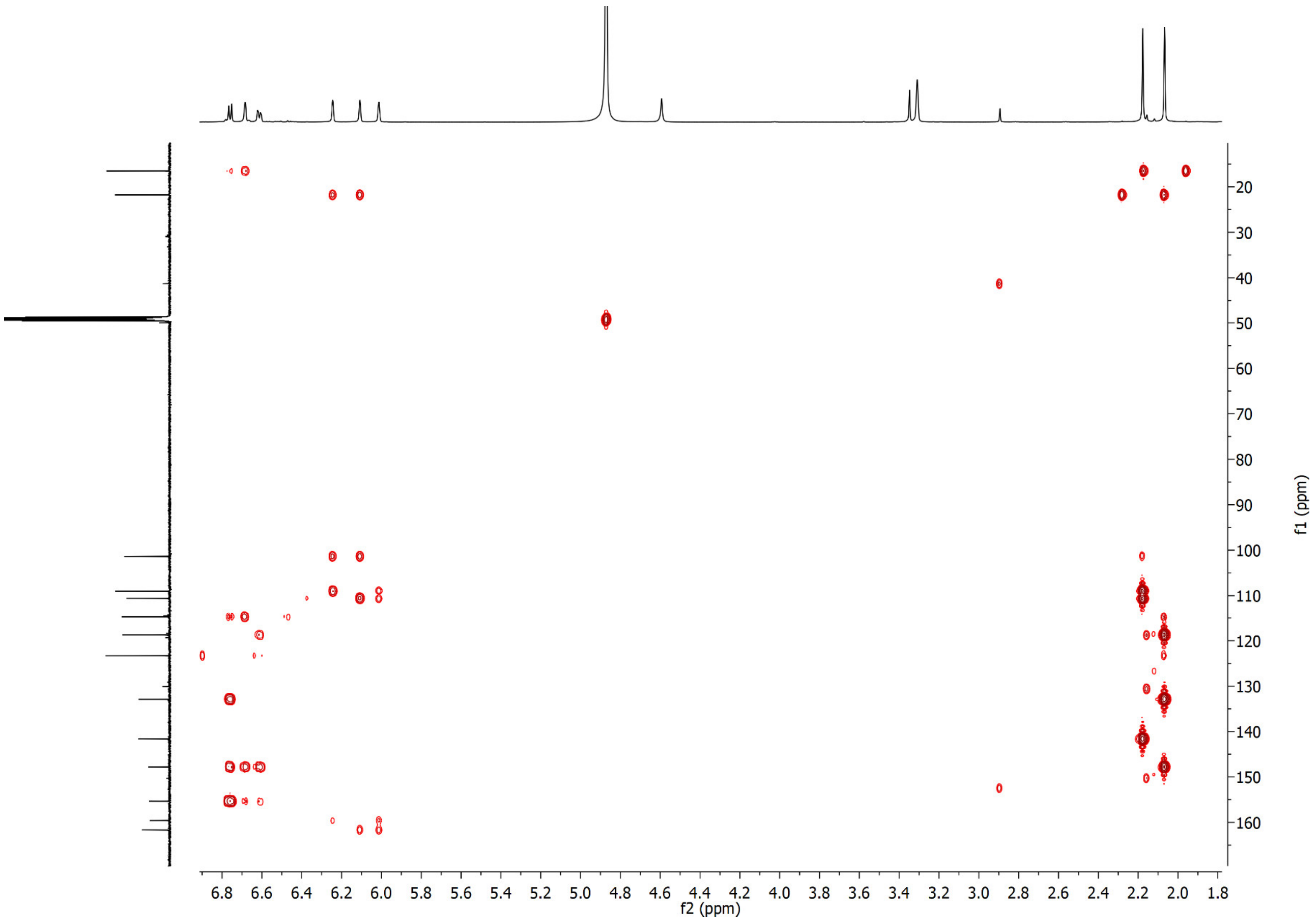

Figure S17.82. HMBC spectrum of **15** in methanol-d_4.

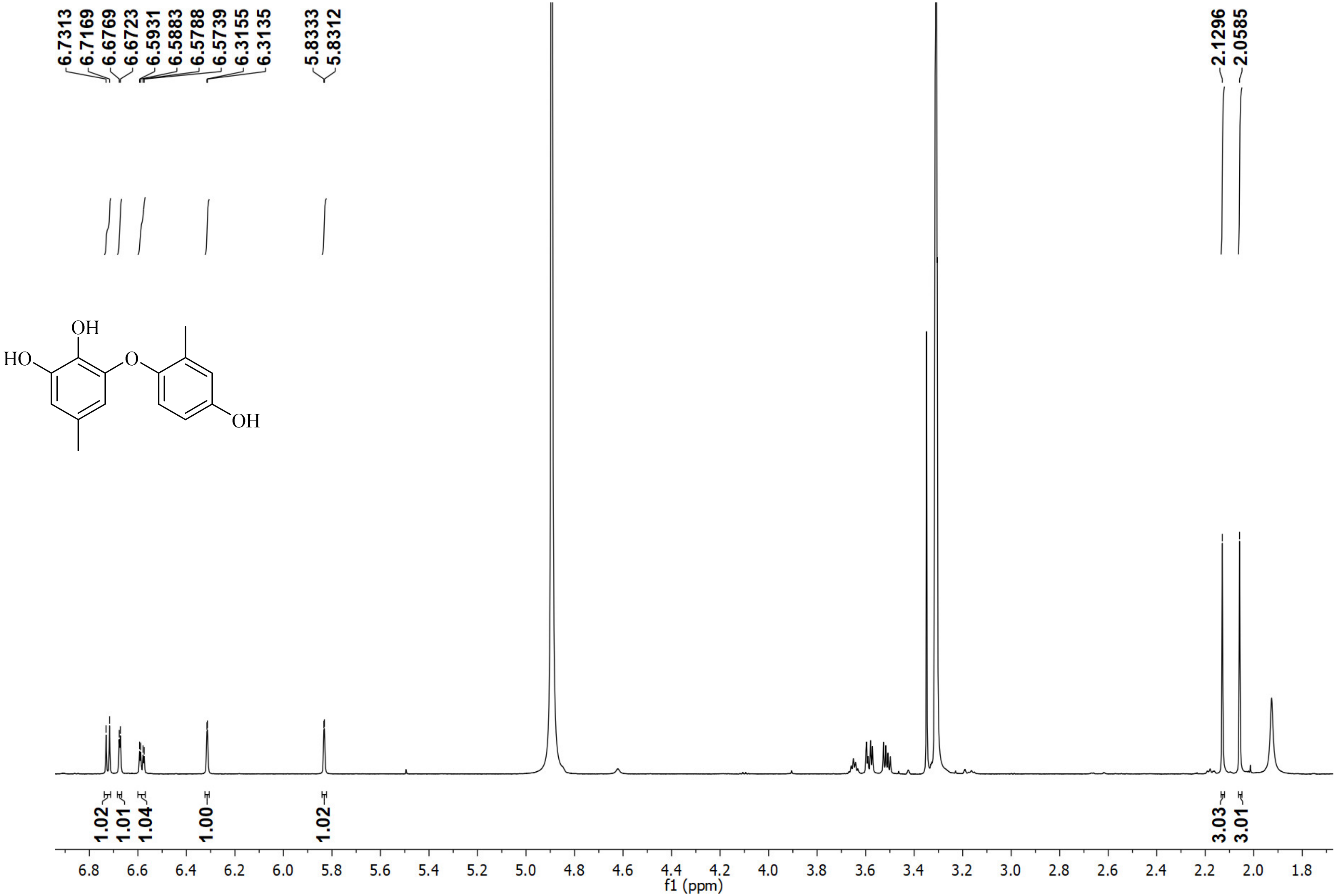

Figure S17.83. ^{1}H NMR spectrum of **15a** in methanol-d_4.

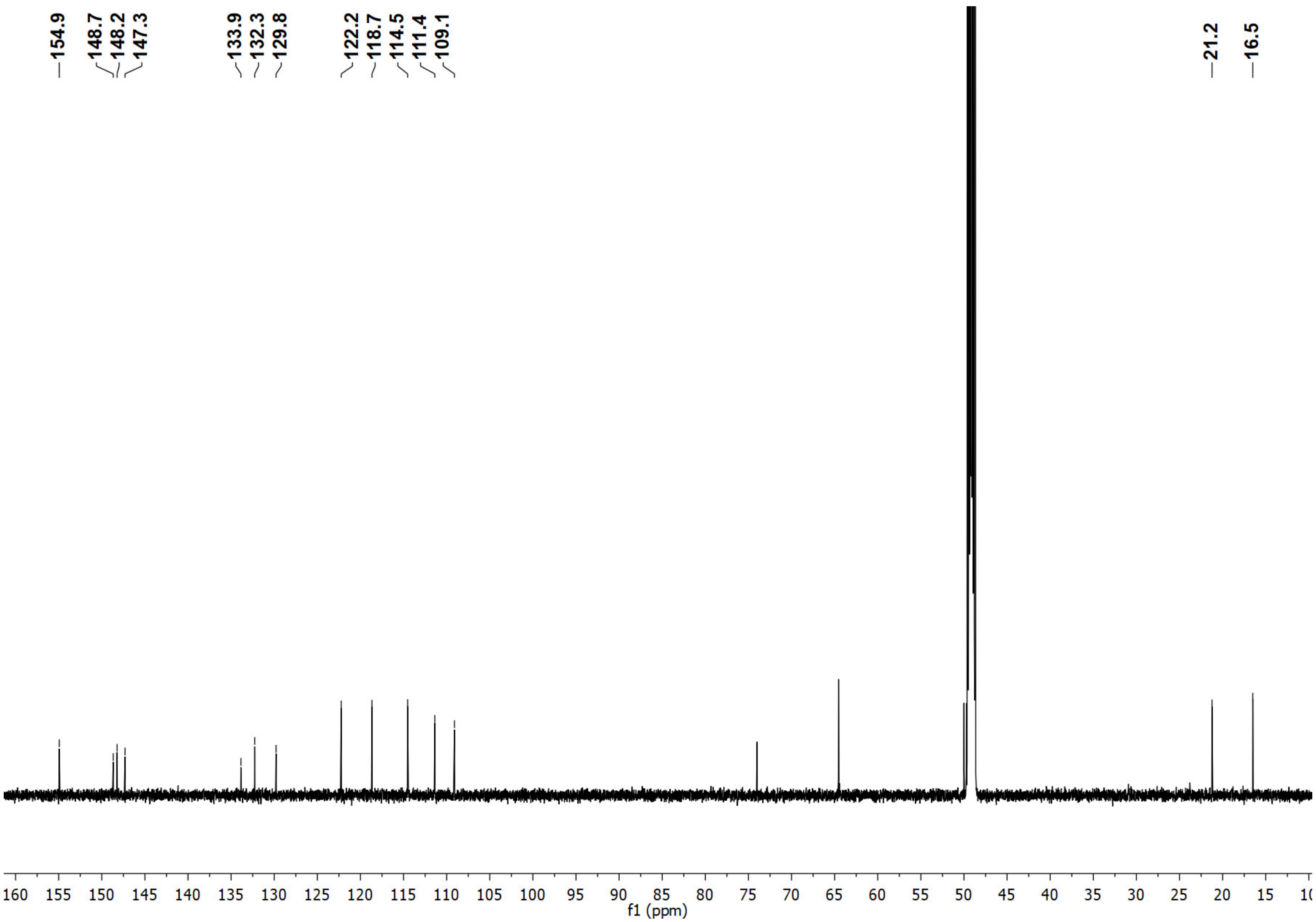

Figure S17.84. ^{13}C NMR spectrum of **15a** in methanol-d_4.

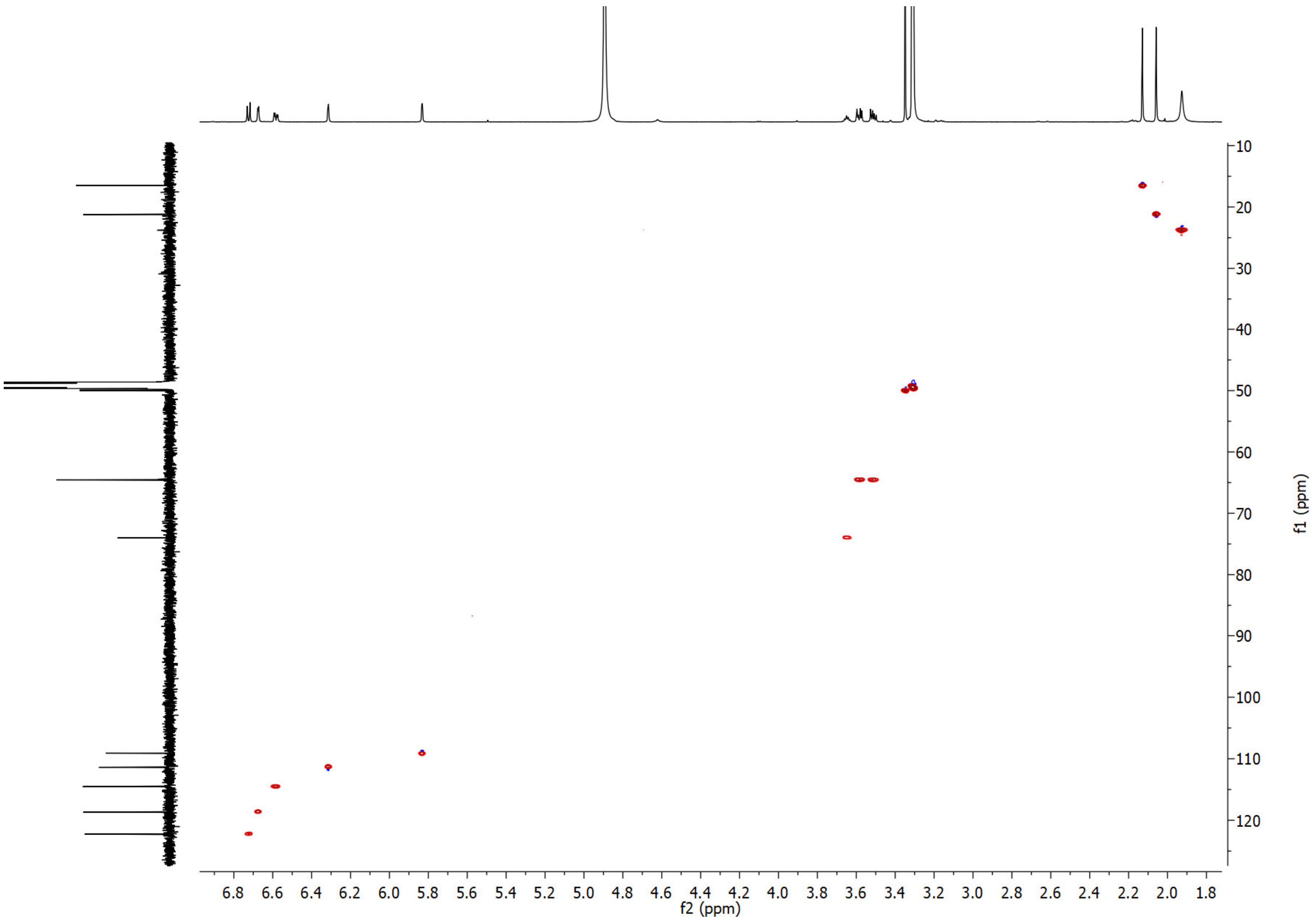

Figure S17.85. HSQC spectrum of **15a** in methanol-d_4.

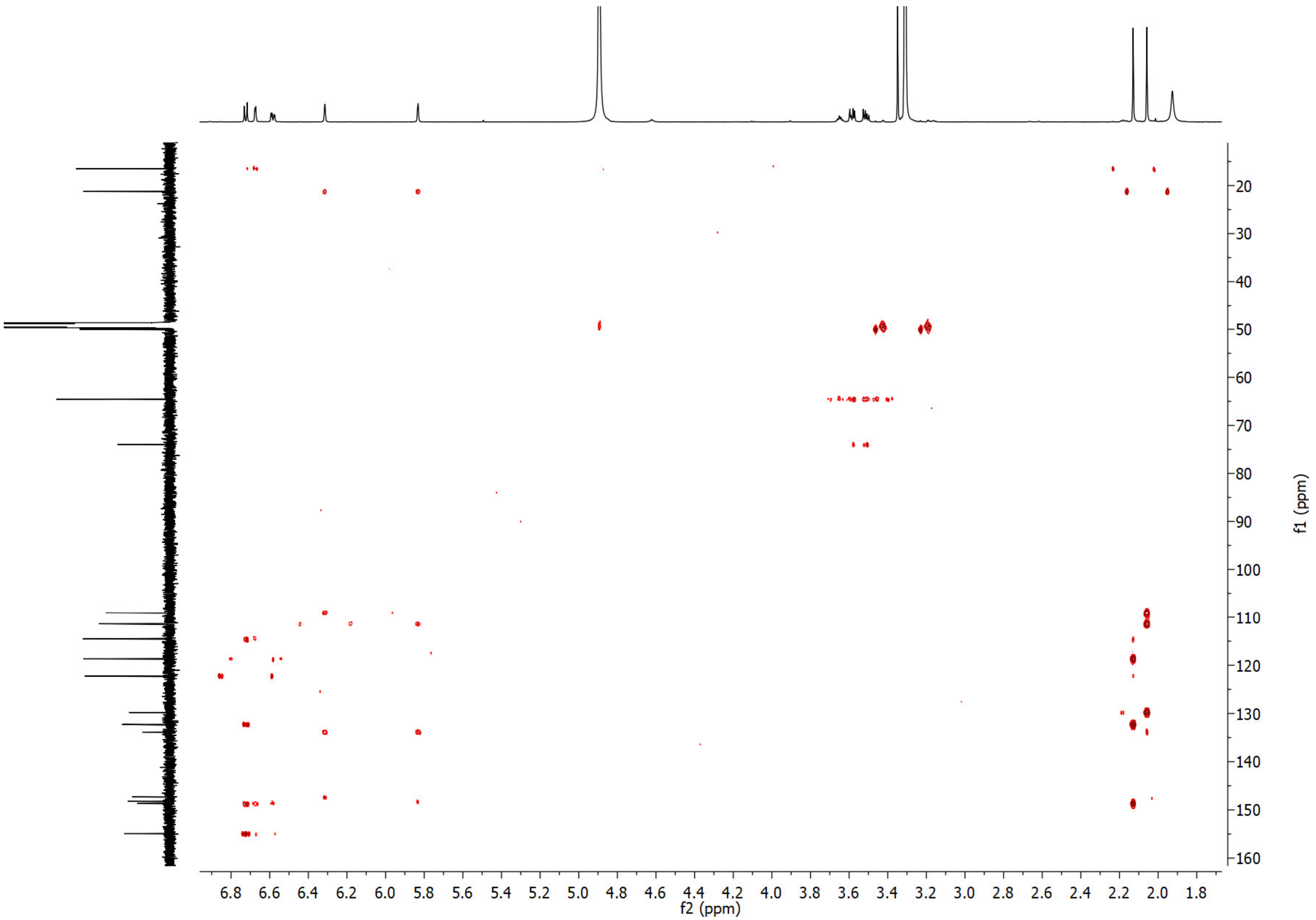

Figure S17.86. HMBC spectrum of **15a** in methanol-d_4.

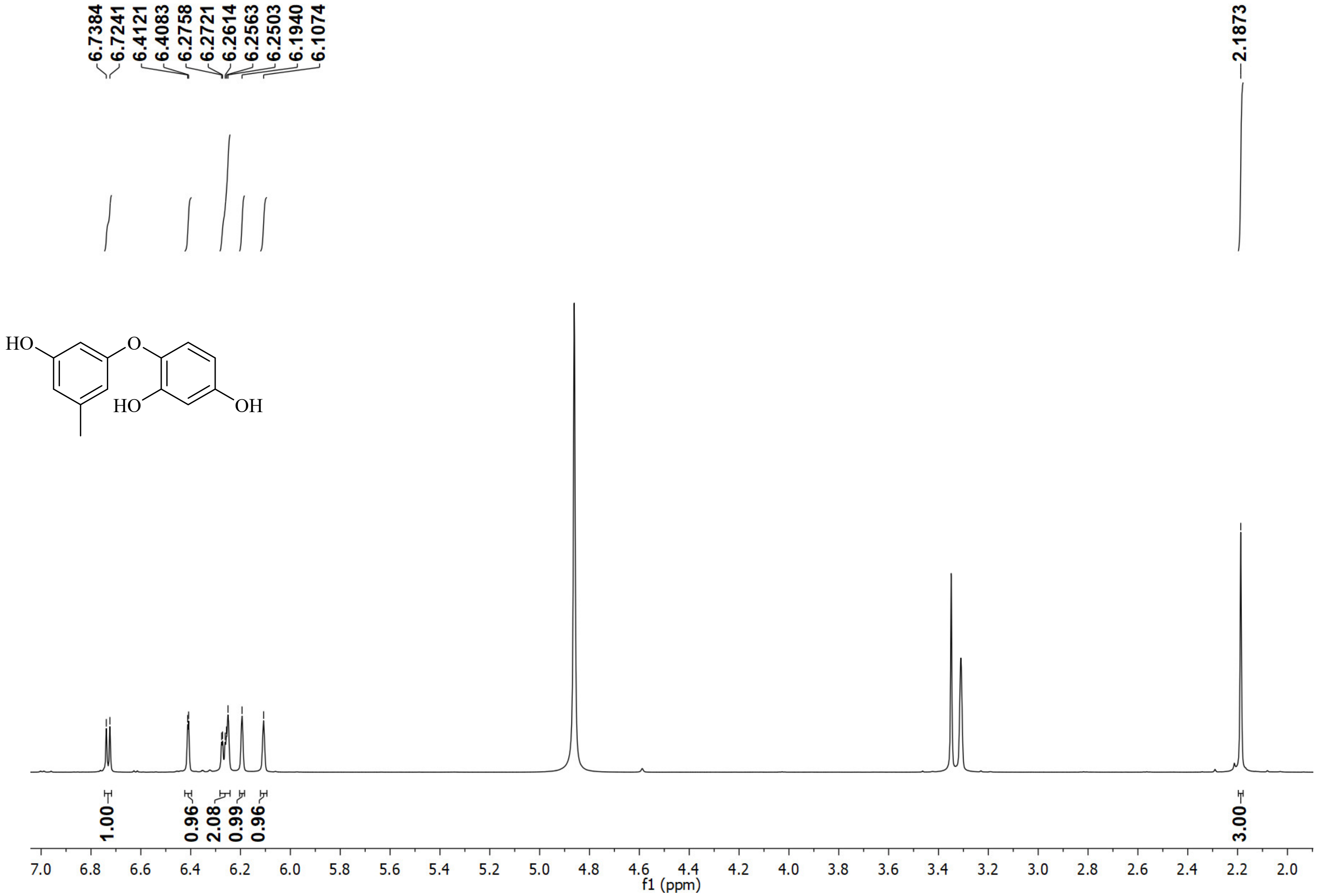

Figure S17.87. ^{1}H NMR spectrum of **16** in methanol-d_4.

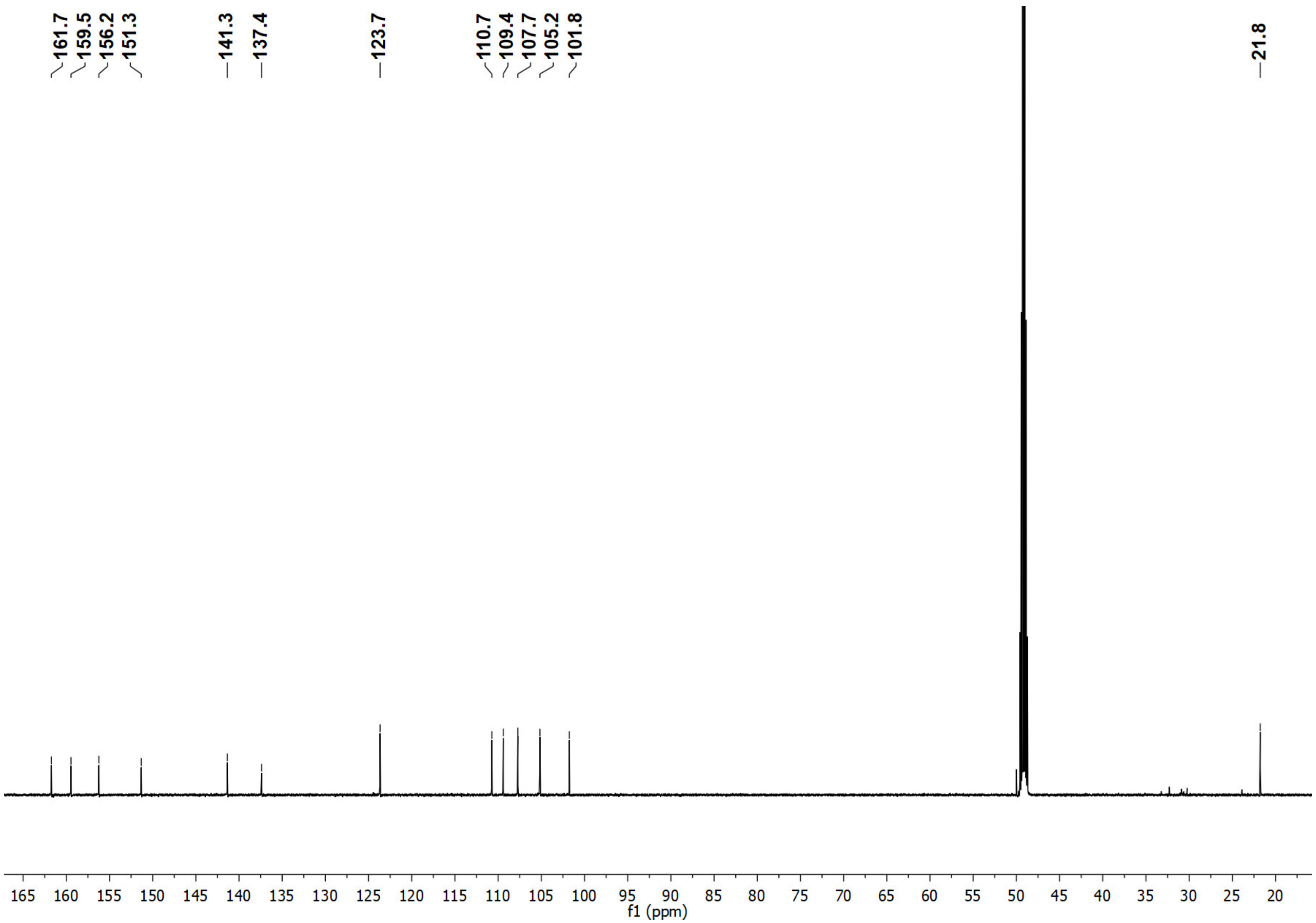

Figure S17.88. ^{13}C NMR spectrum of **16** in methanol-d_4.

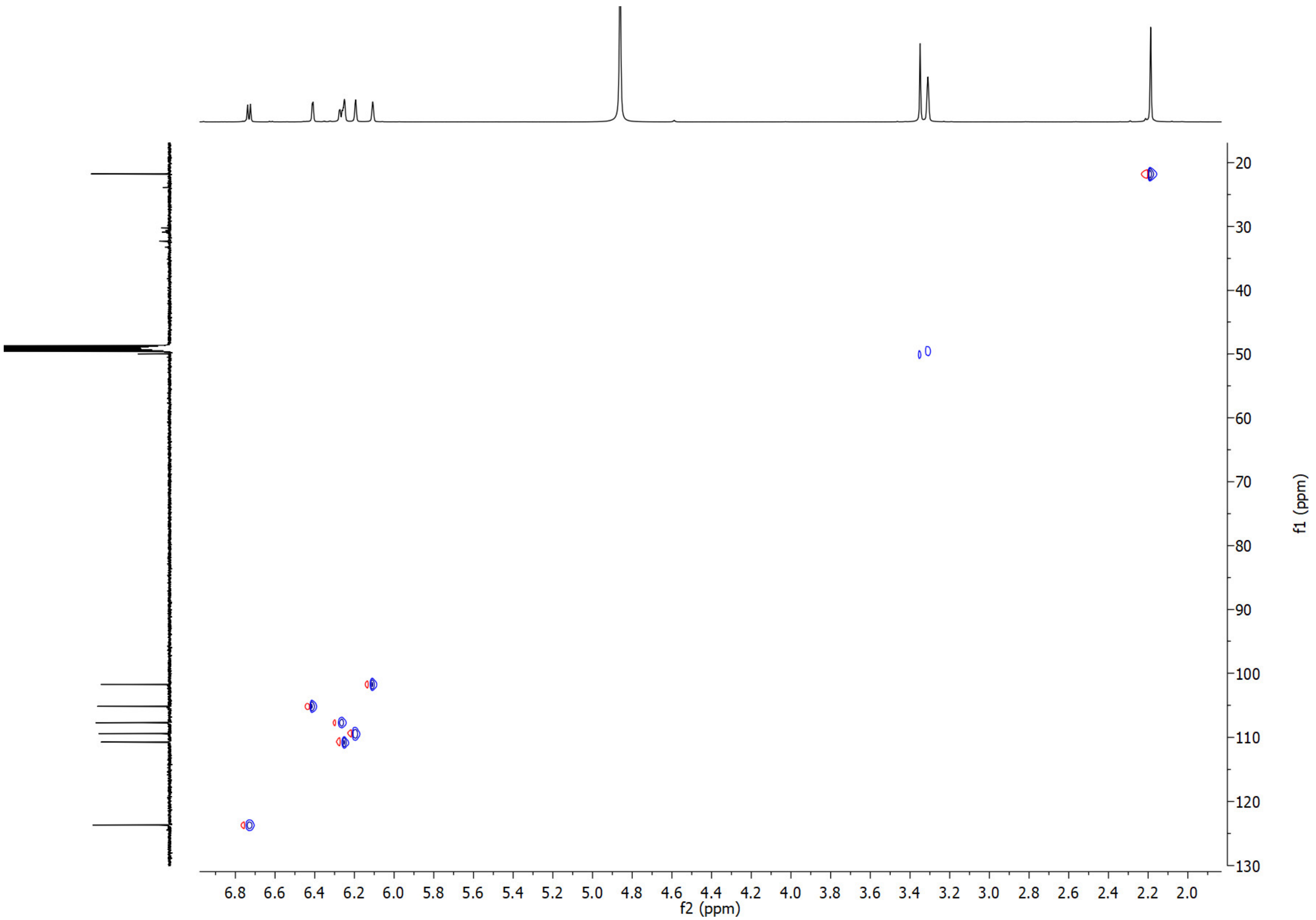

Figure S17.89. HSQC spectrum of **16** in methanol-d_4.

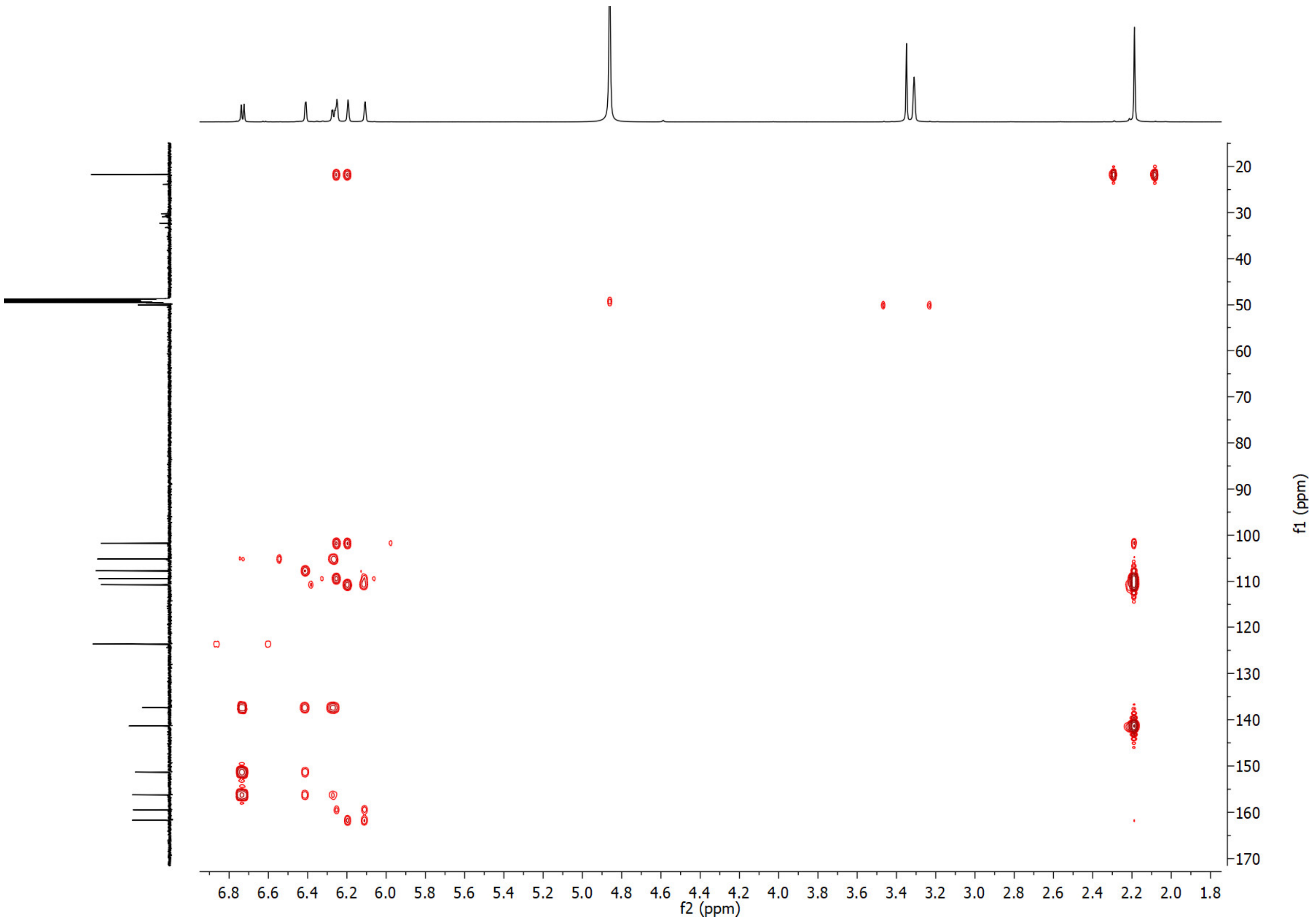

Figure S17.90. HMBC spectrum of **16** in methanol-d_4.

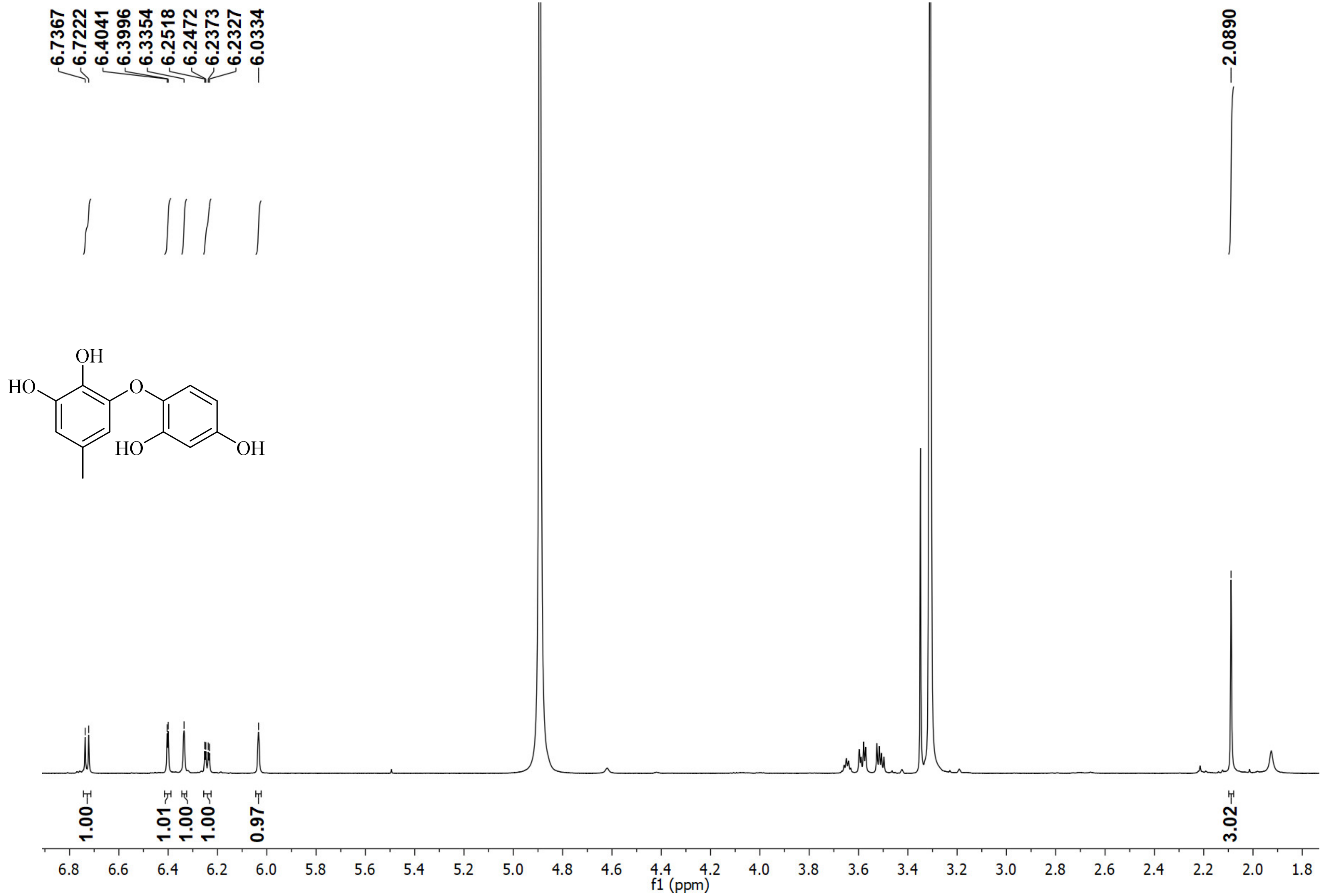

Figure S17.91. ^{1}H NMR spectrum of **16a** in methanol-d_4.

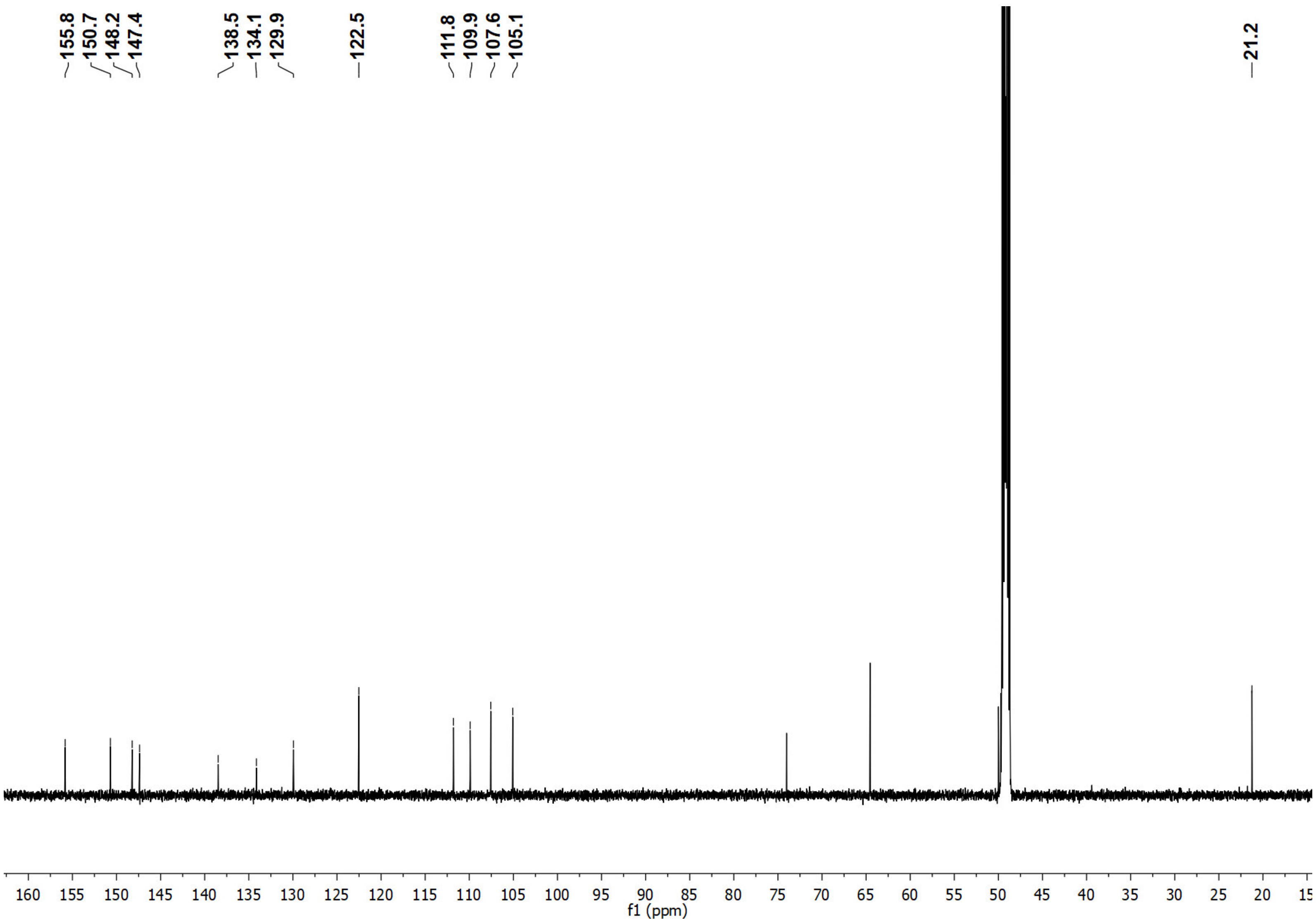

Figure S17.92. ^{13}C NMR spectrum of **16a** in methanol-d_4.

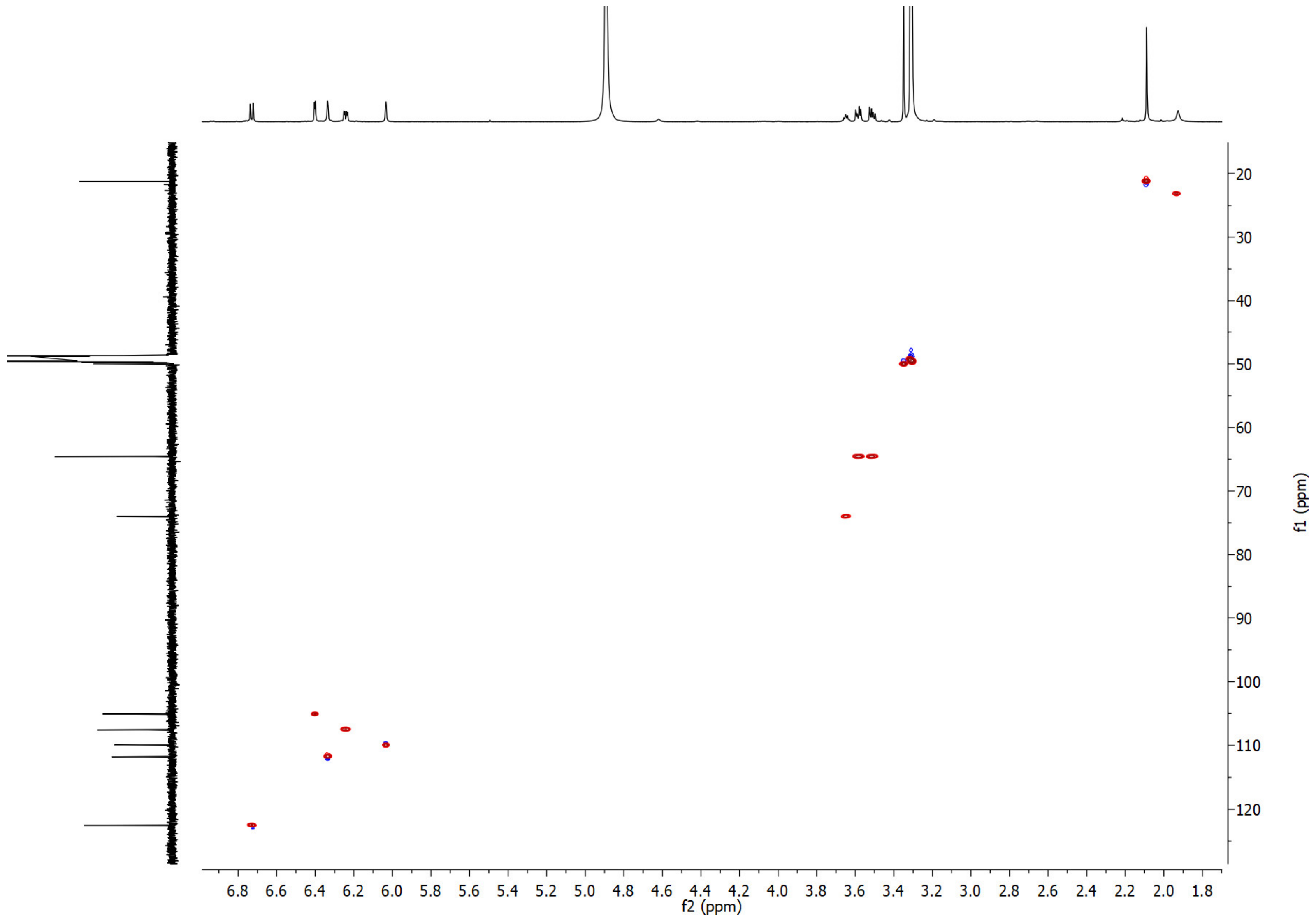

Figure S17.93. HSQC spectrum of **16a** in methanol-d_4.

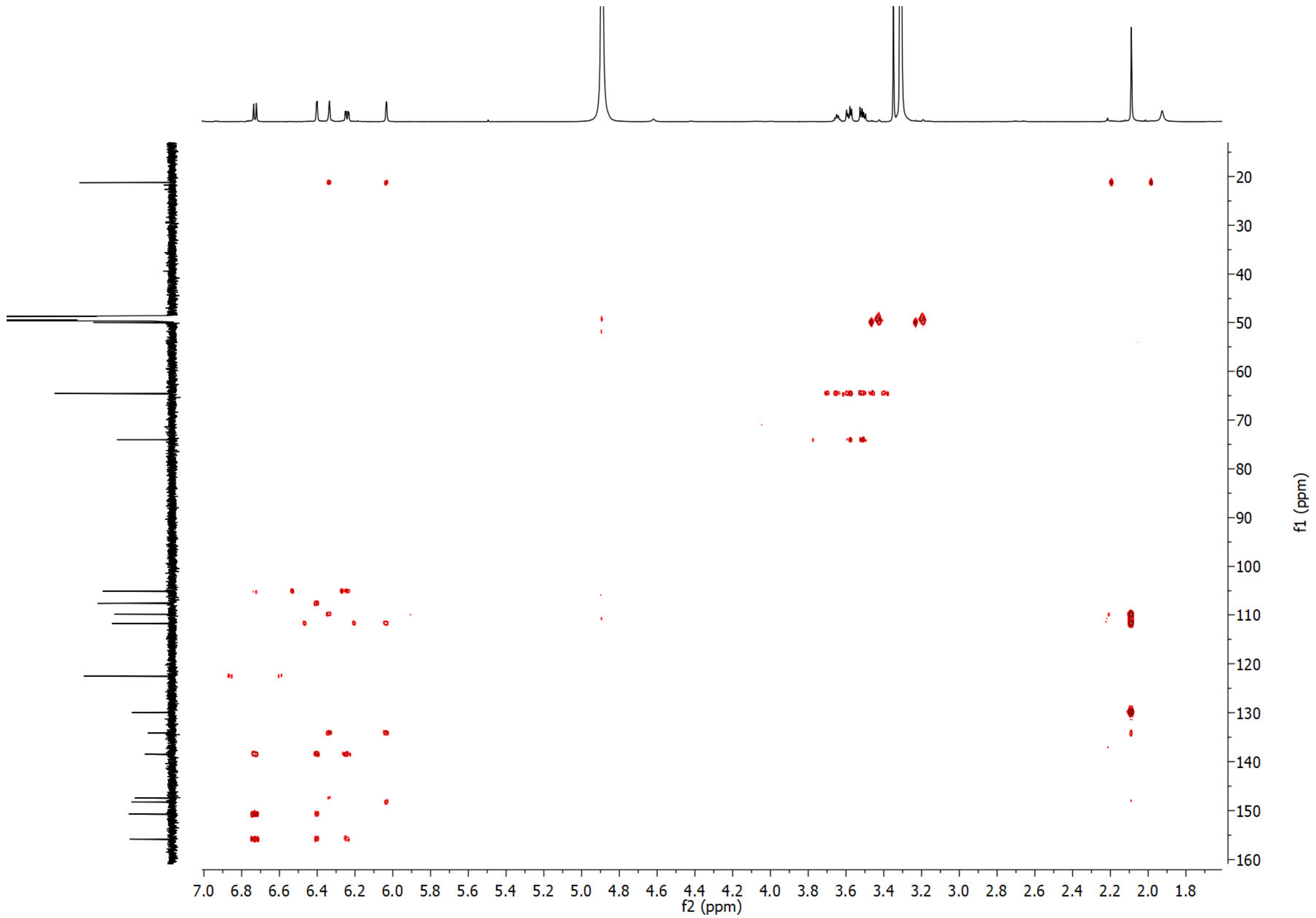

Figure S17.94. HMBC spectrum of **16a** in methanol-d_4.

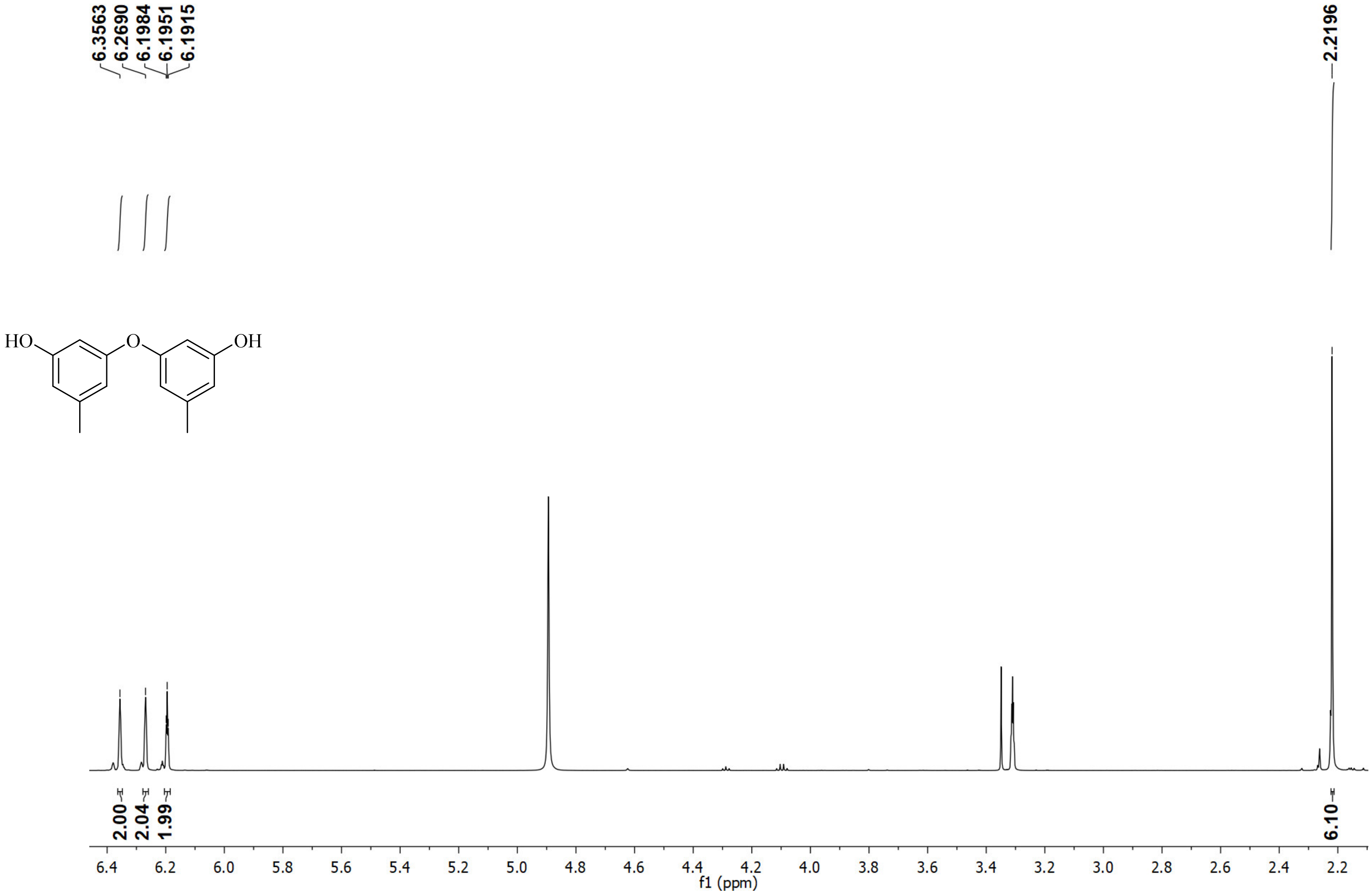

Figure S17.95. ^{1}H NMR spectrum of **17** in methanol-d_4.

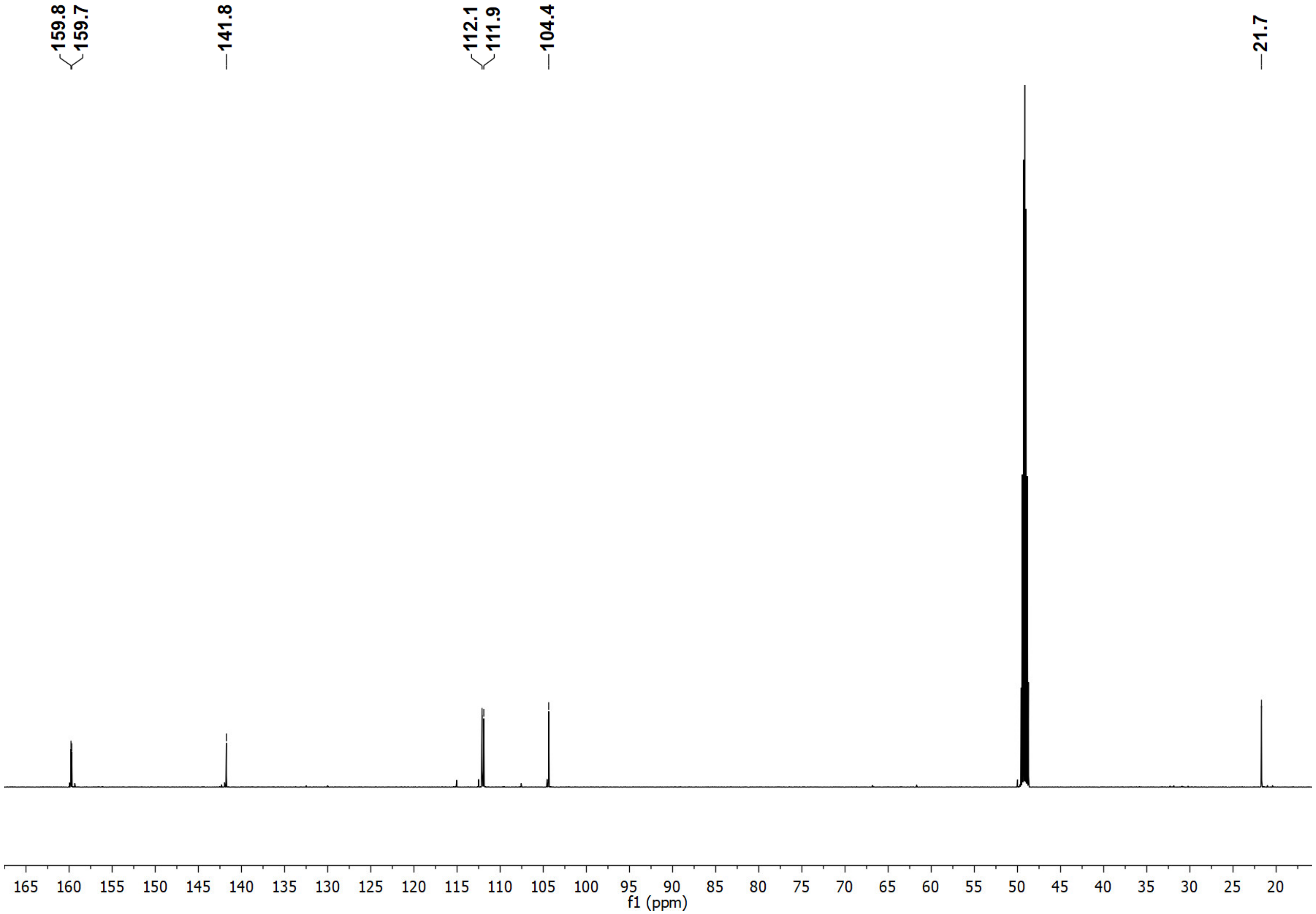

Figure S17.96. ^{13}C NMR spectrum of **17** in methanol-d_4.

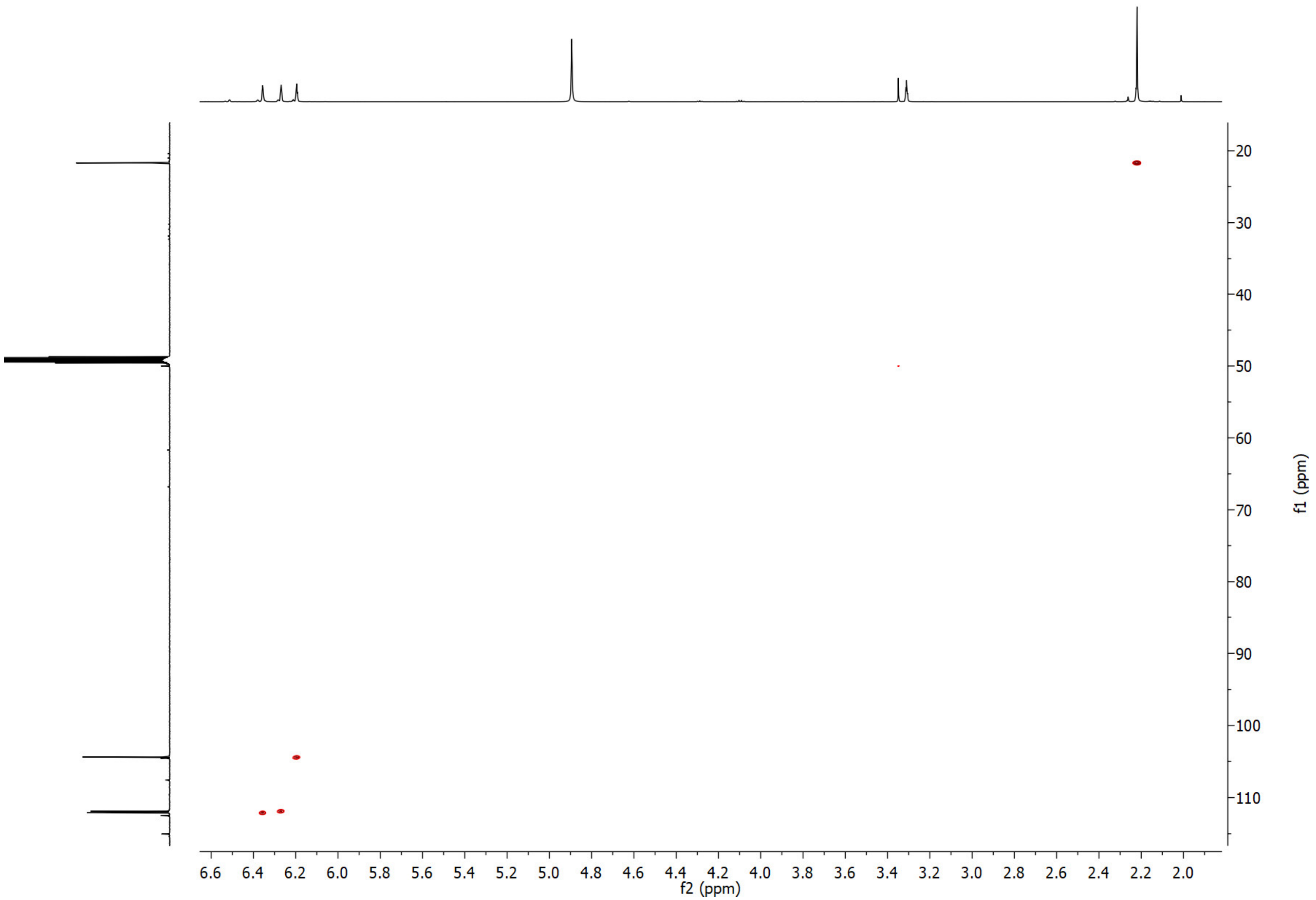

Figure S17.97. HSQC spectrum of **17** in methanol-d_4.

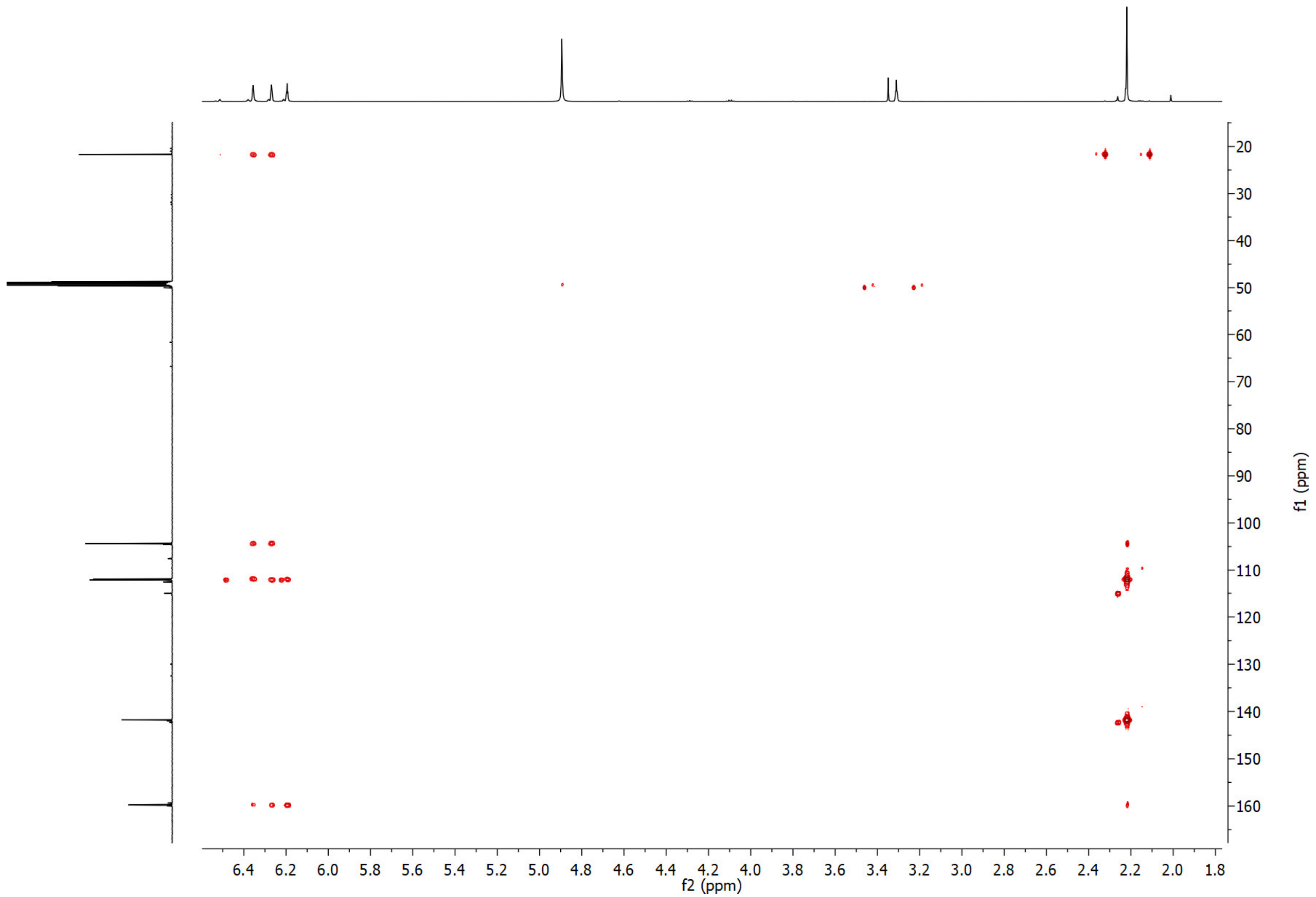

Figure S17.98. HMBC spectrum of **17** in methanol-*d*$_4$.

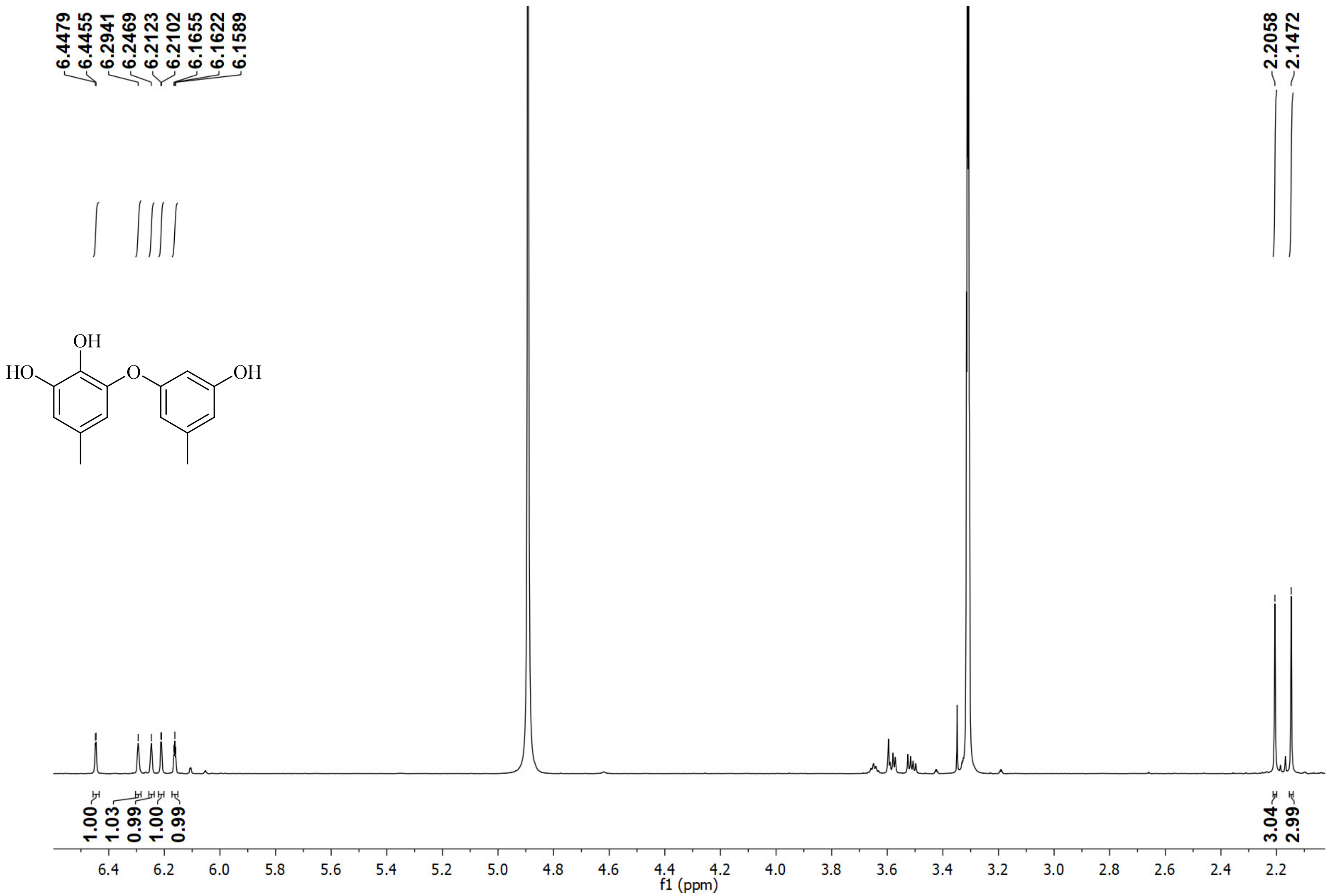

Figure S17.99. ^{1}H NMR spectrum of **17a** in methanol-d_4.

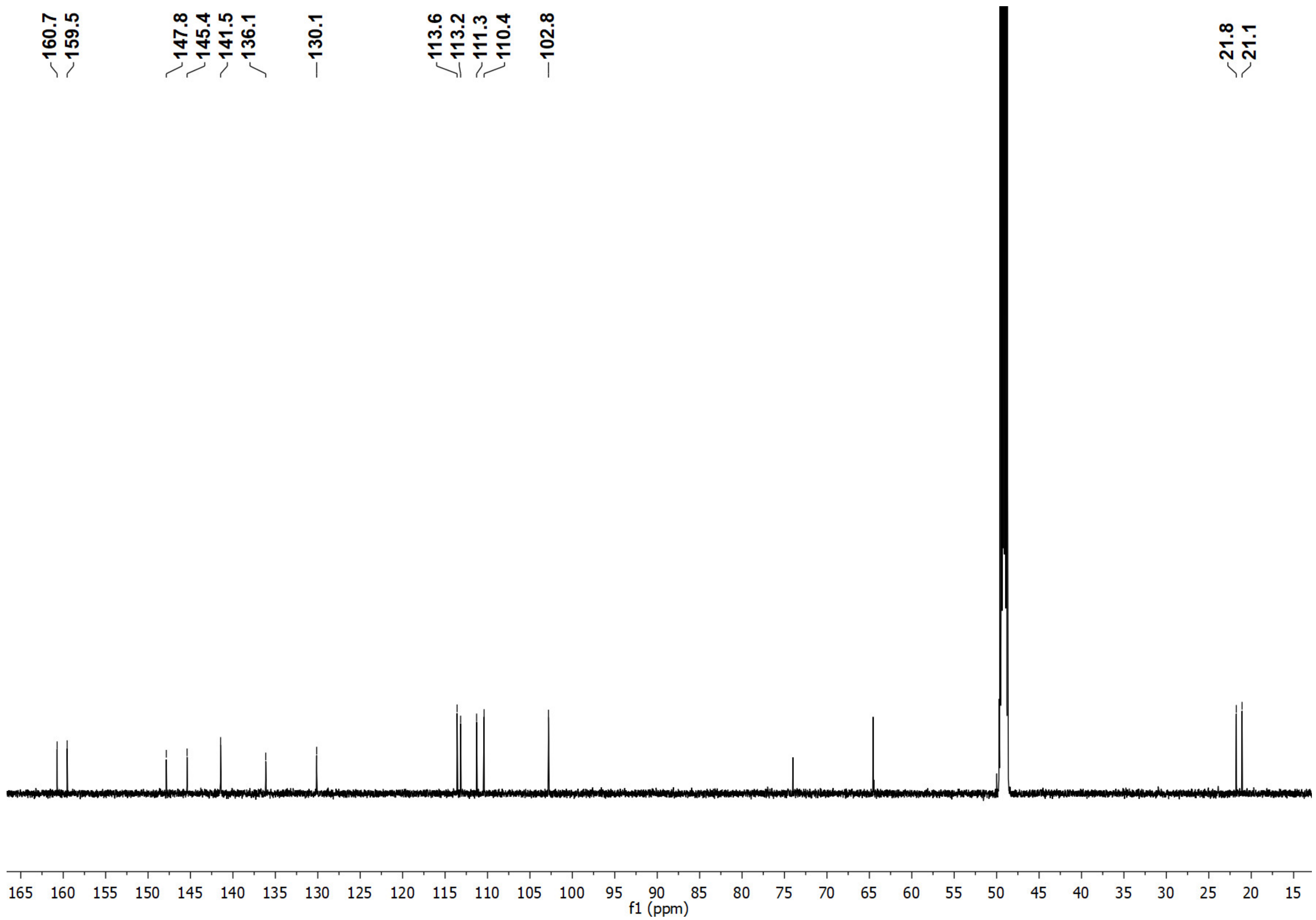

Figure S17.100. ^{13}C NMR spectrum of **17a** in methanol-d_4.

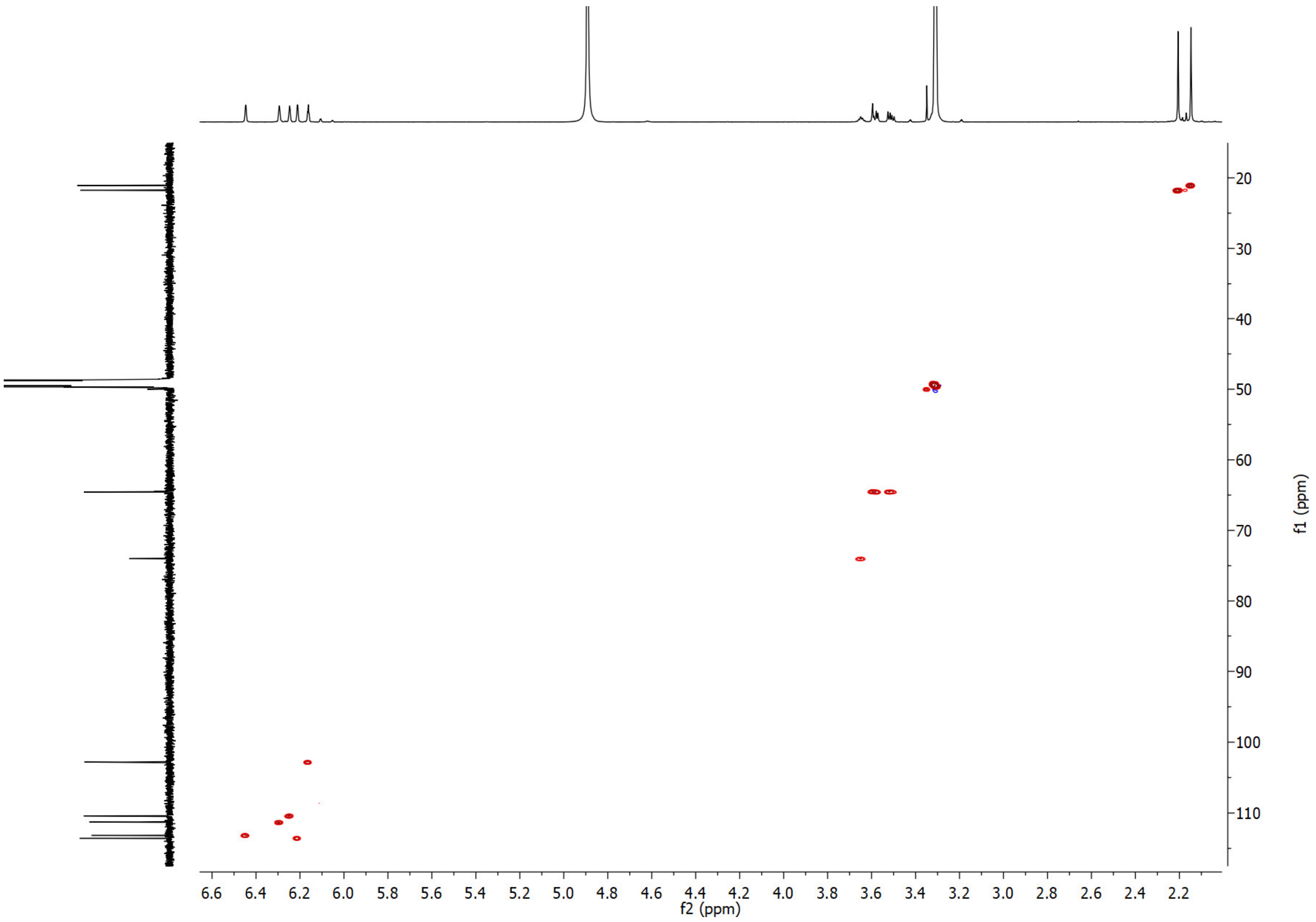

Figure S17.101. HSQC spectrum of **17a** in methanol-d_4.

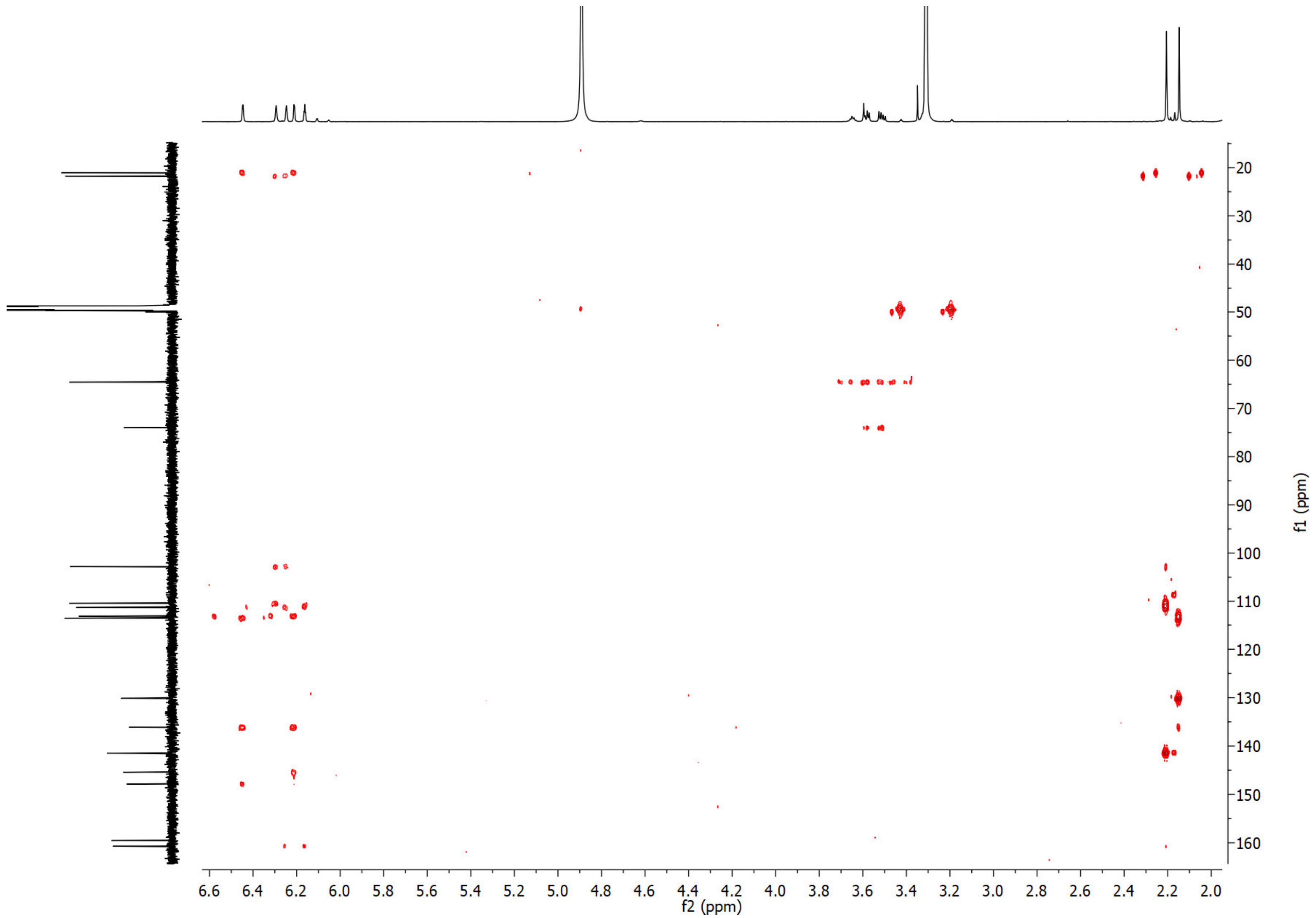

Figure S17.102. HMBC spectrum of **17a** in methanol-d_4.

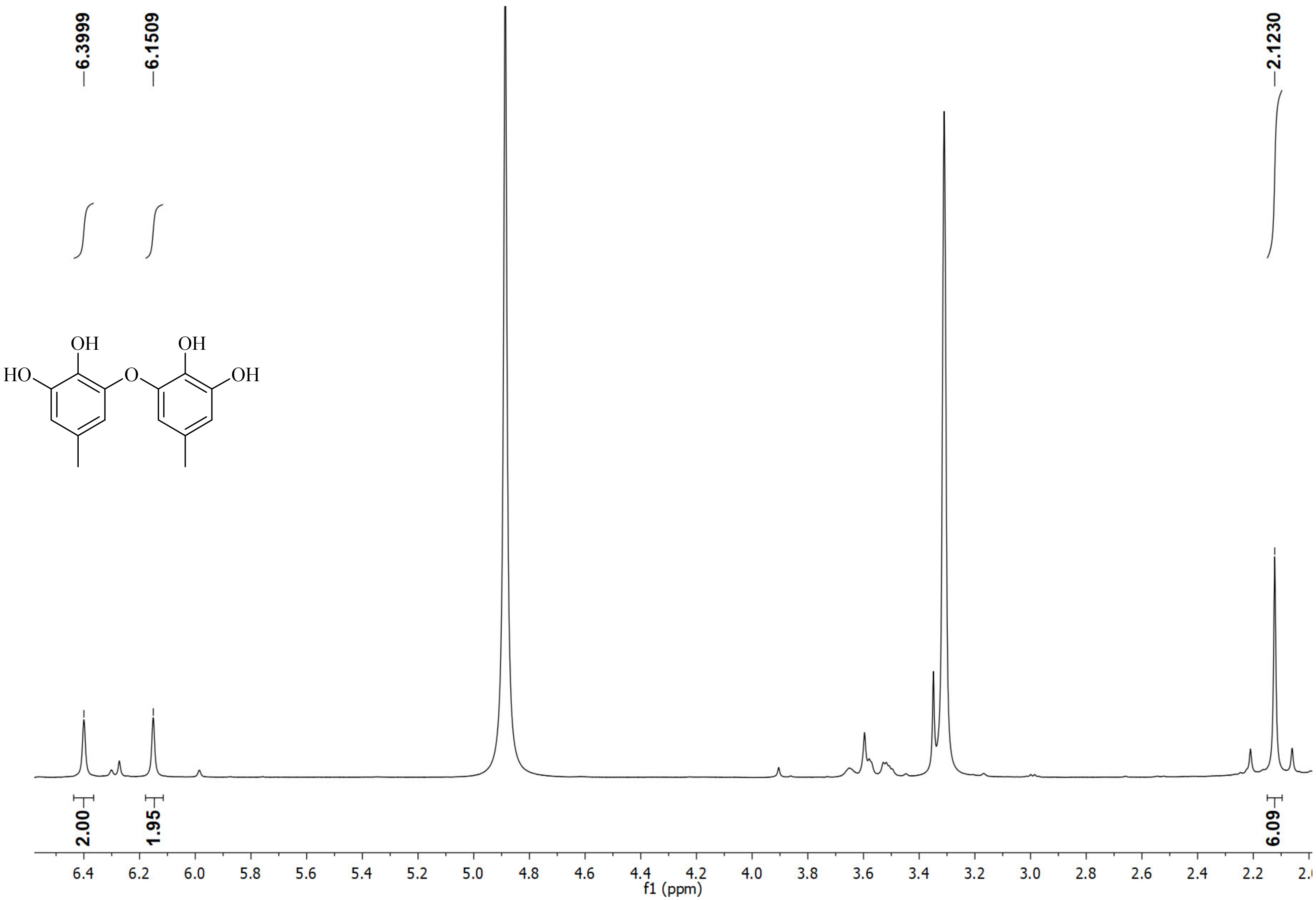

Figure S17.103. ^{1}H NMR spectrum of **17b** in methanol-d_4.

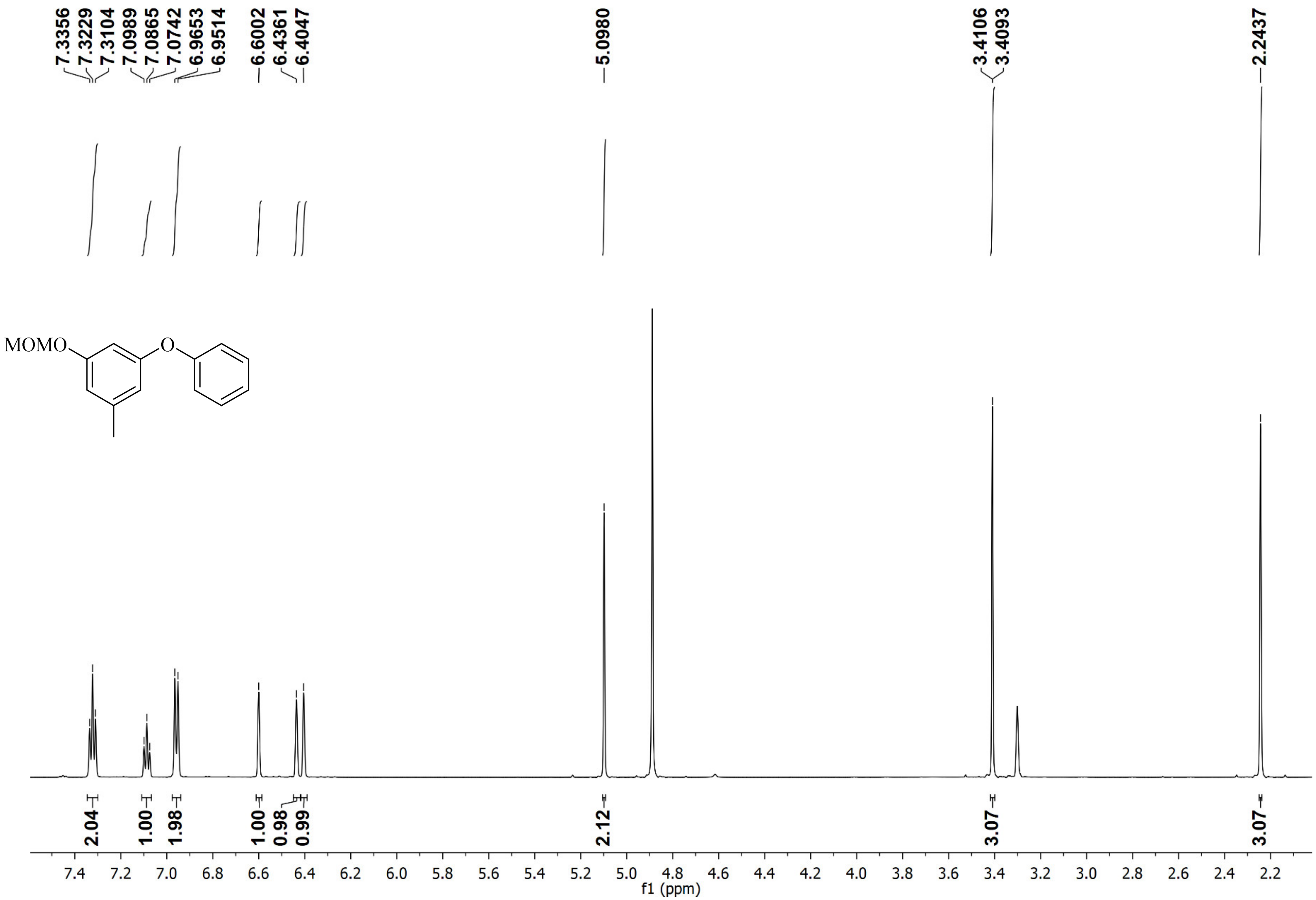

Figure S17.104. ^{1}H NMR spectrum of **18** in methanol-d_4.

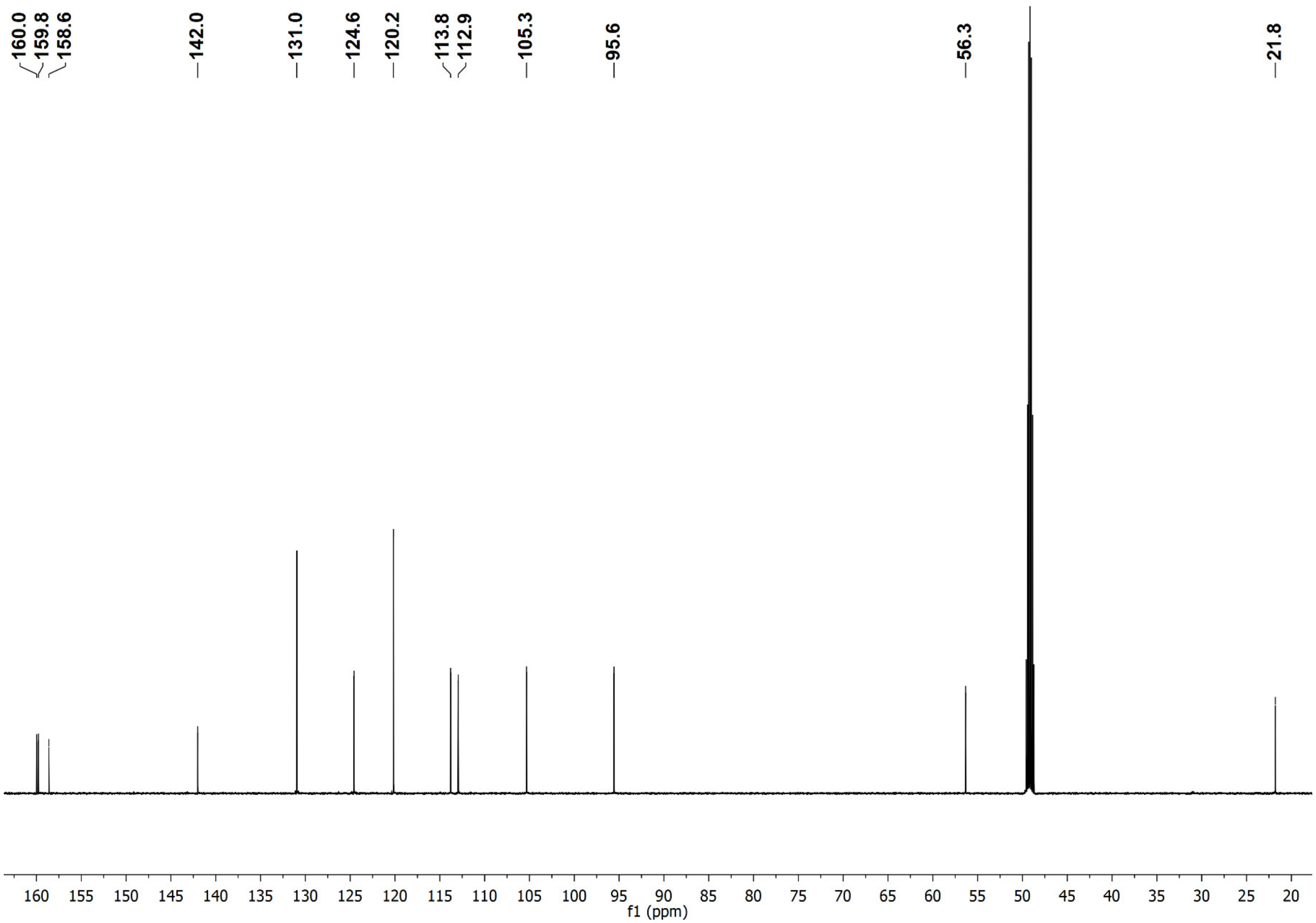

Figure S17.105. ^{13}C NMR spectrum of **18** in methanol-d_4.

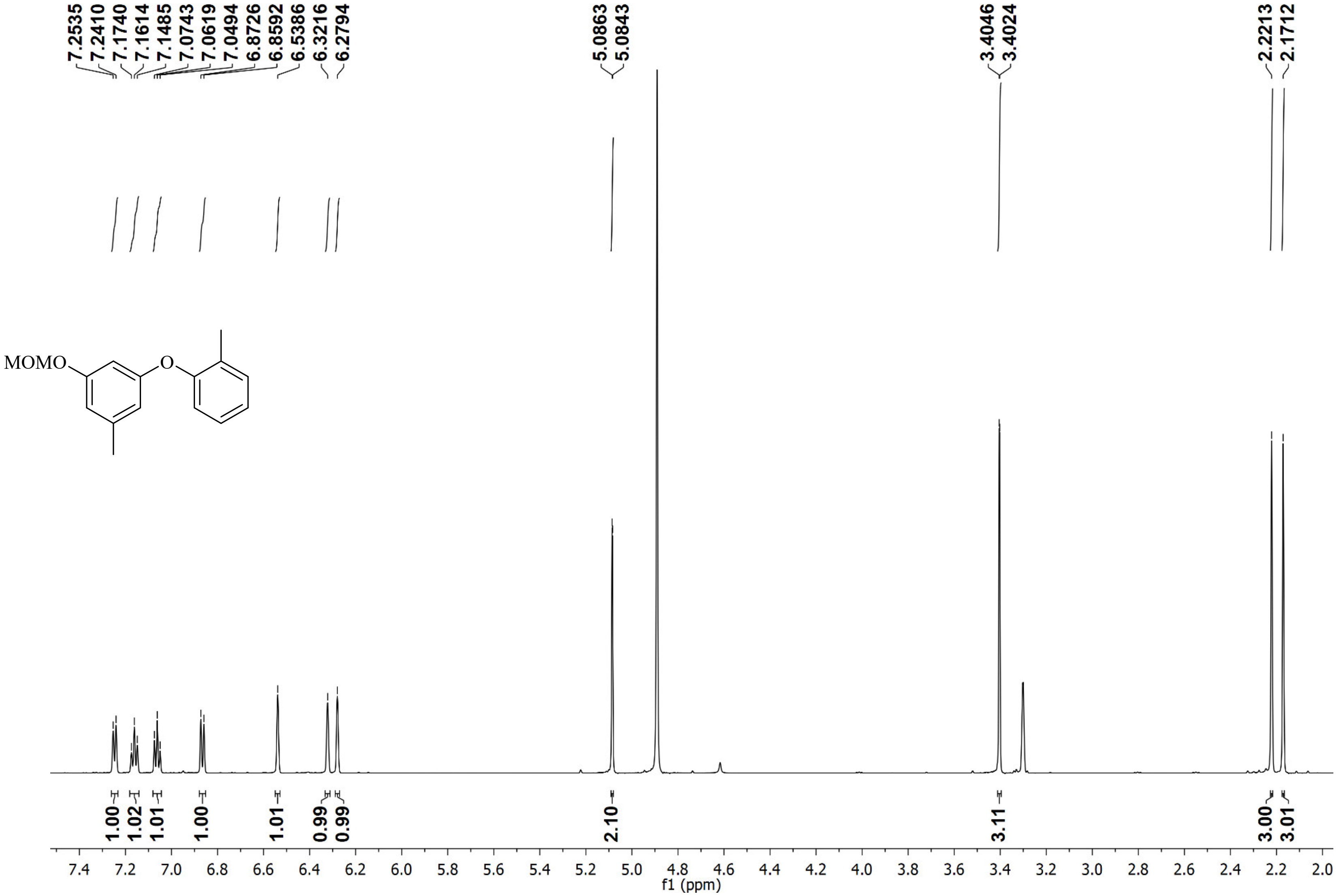

Figure S17.106. ^{1}H NMR spectrum of **19** in methanol-d_4.

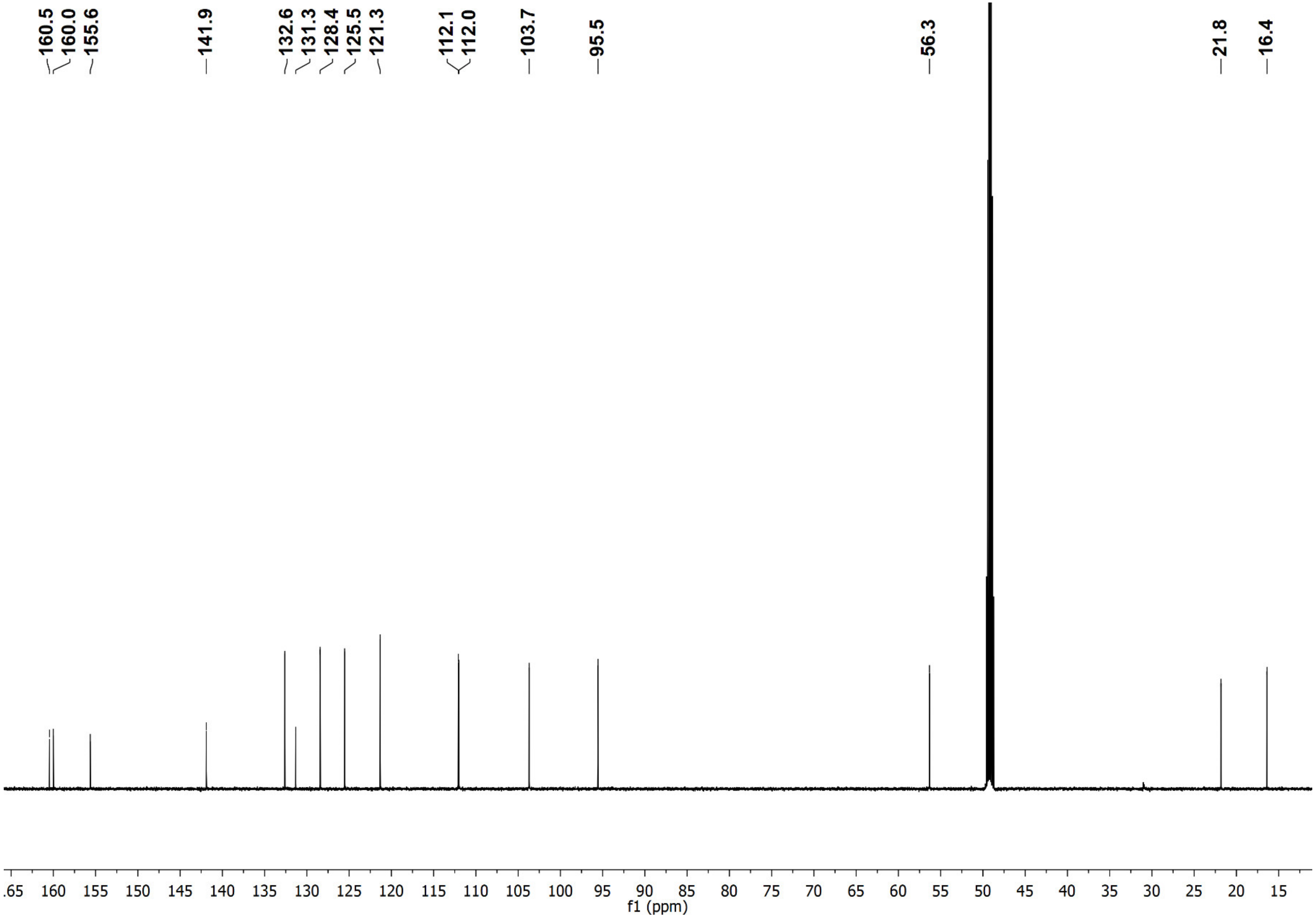

Figure S17.107. ^{13}C NMR spectrum of **19** in methanol-d_4.

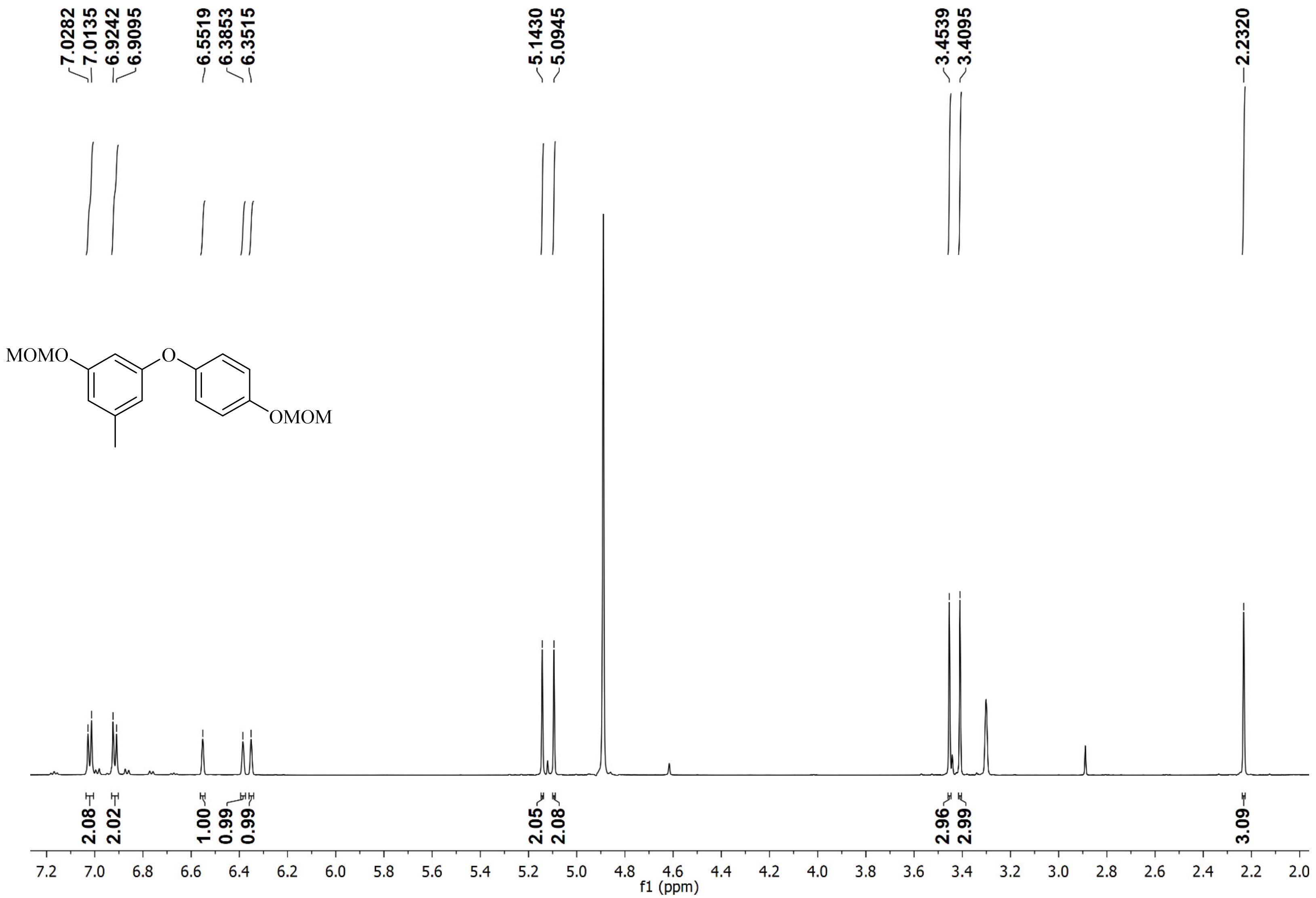

Figure S17.108. ^{1}H NMR spectrum of **20** in methanol-d_4.

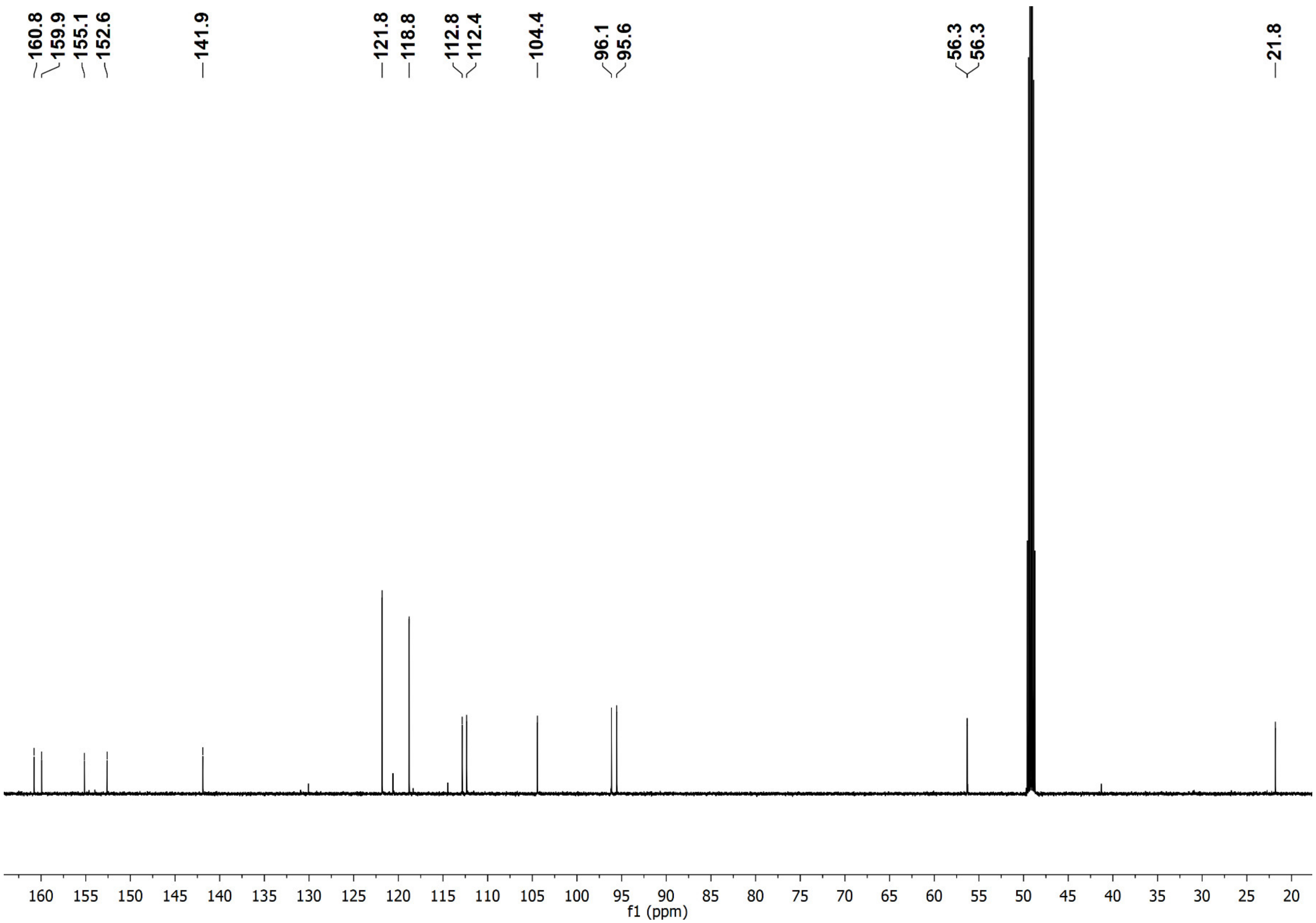

Figure S17.109. ^{13}C NMR spectrum of **20** in methanol-d_4.

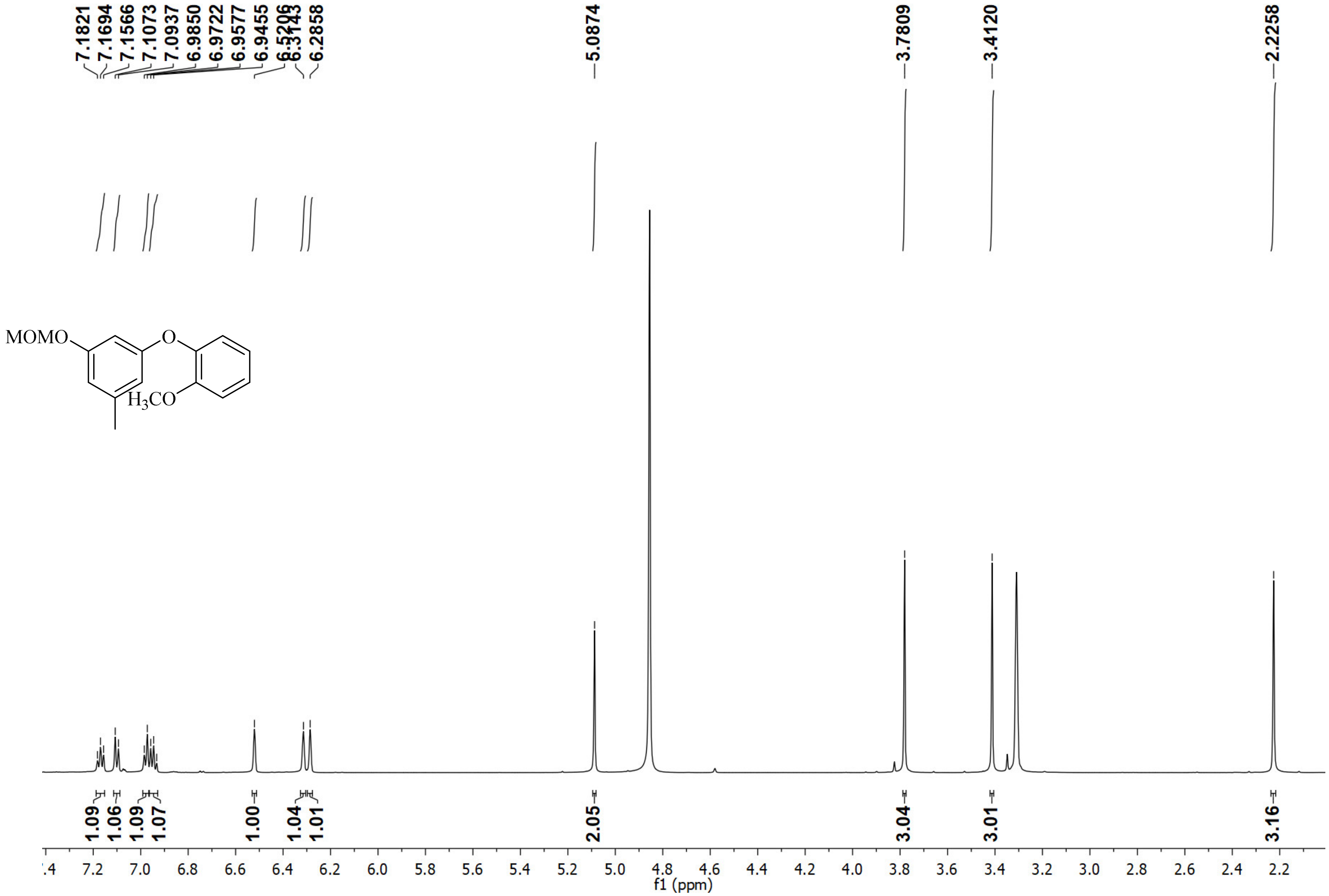

Figure S17.110. ^{1}H NMR spectrum of **21** in methanol-d_4.

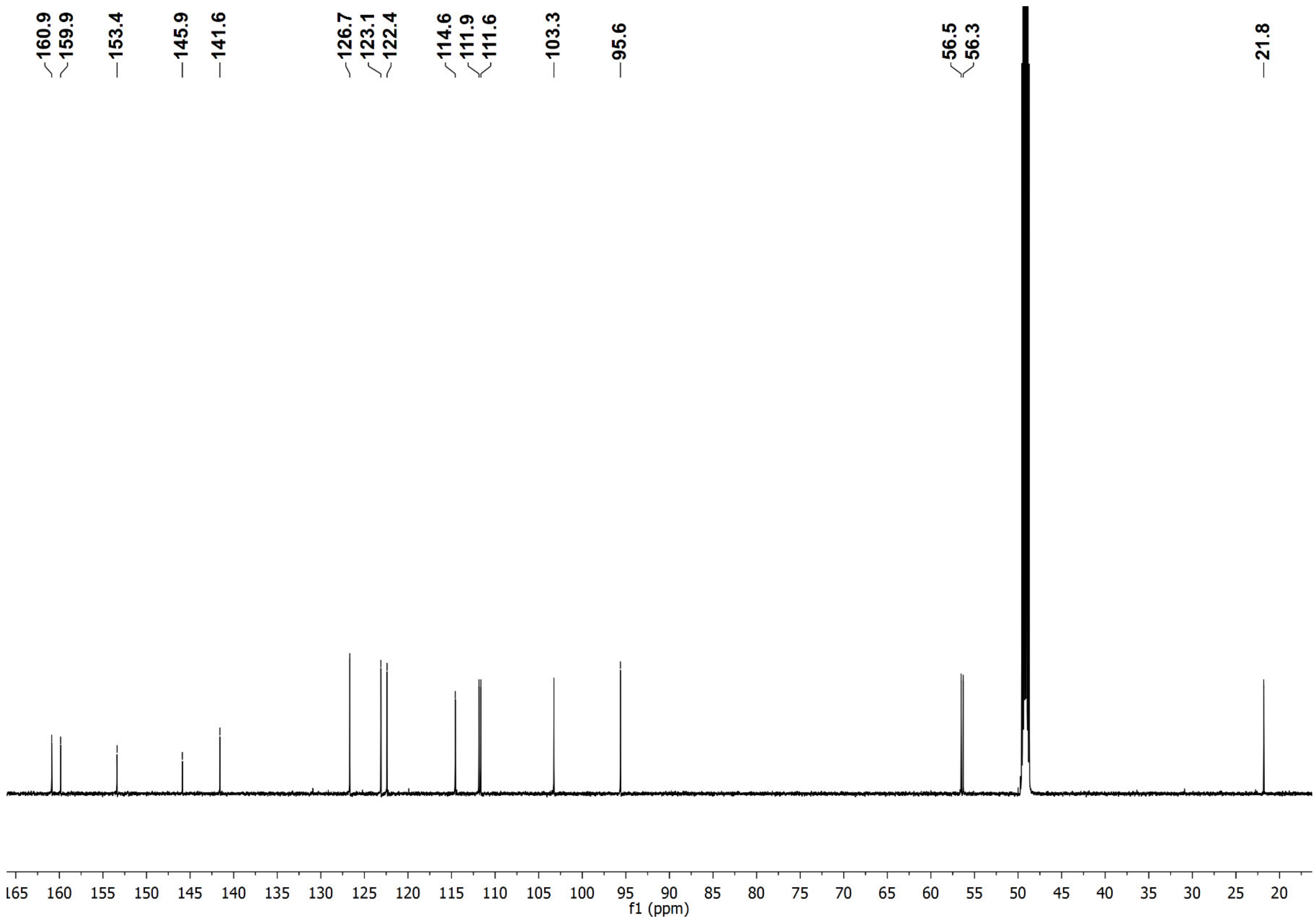

Figure S17.111. ^{13}C NMR spectrum of **21** in methanol-d_4.

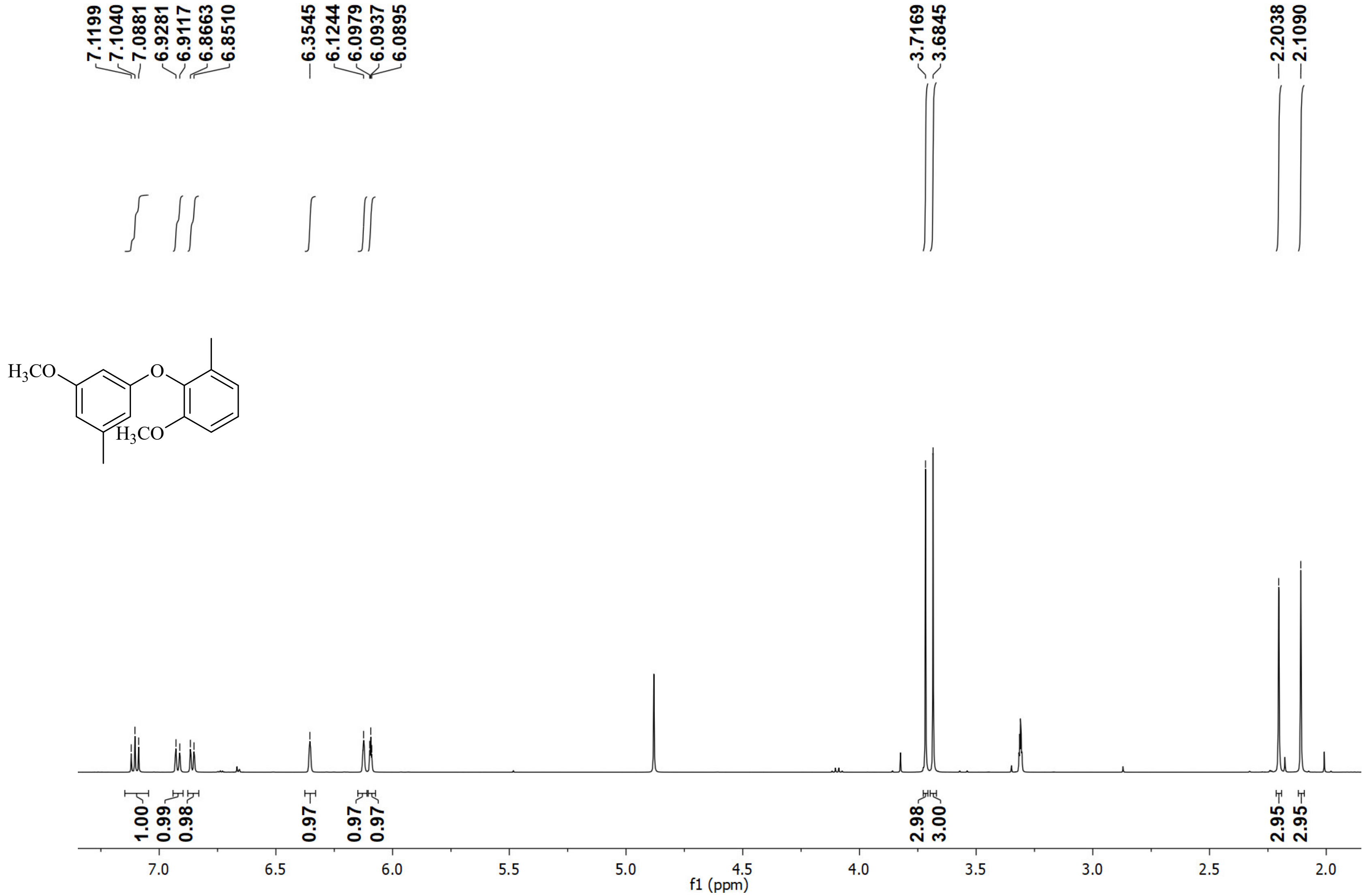

Figure S17.112. ^{1}H NMR spectrum of **22** in methanol-d_4.

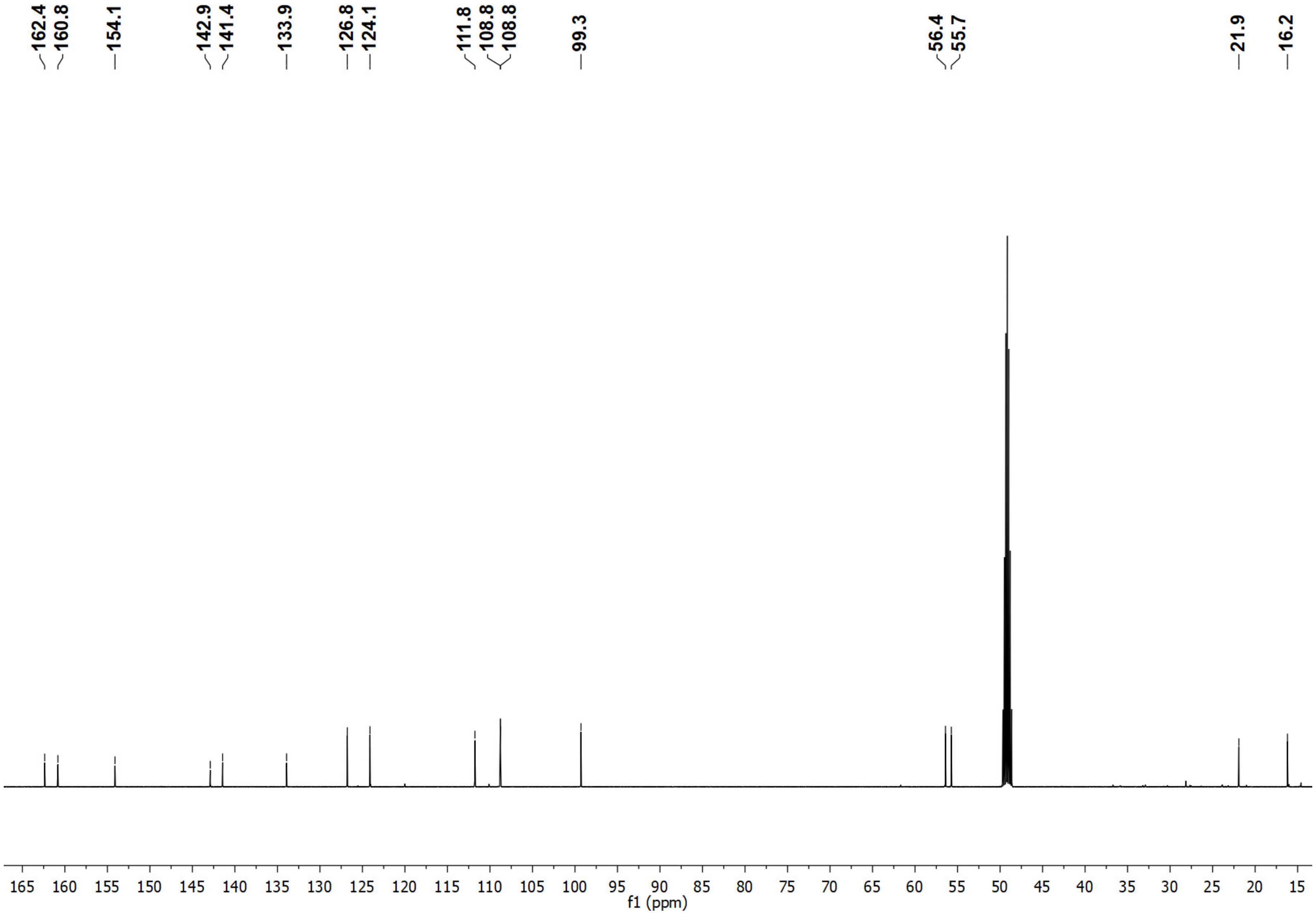

Figure S17.113. ^{13}C NMR spectrum of **22** in methanol-d_4.

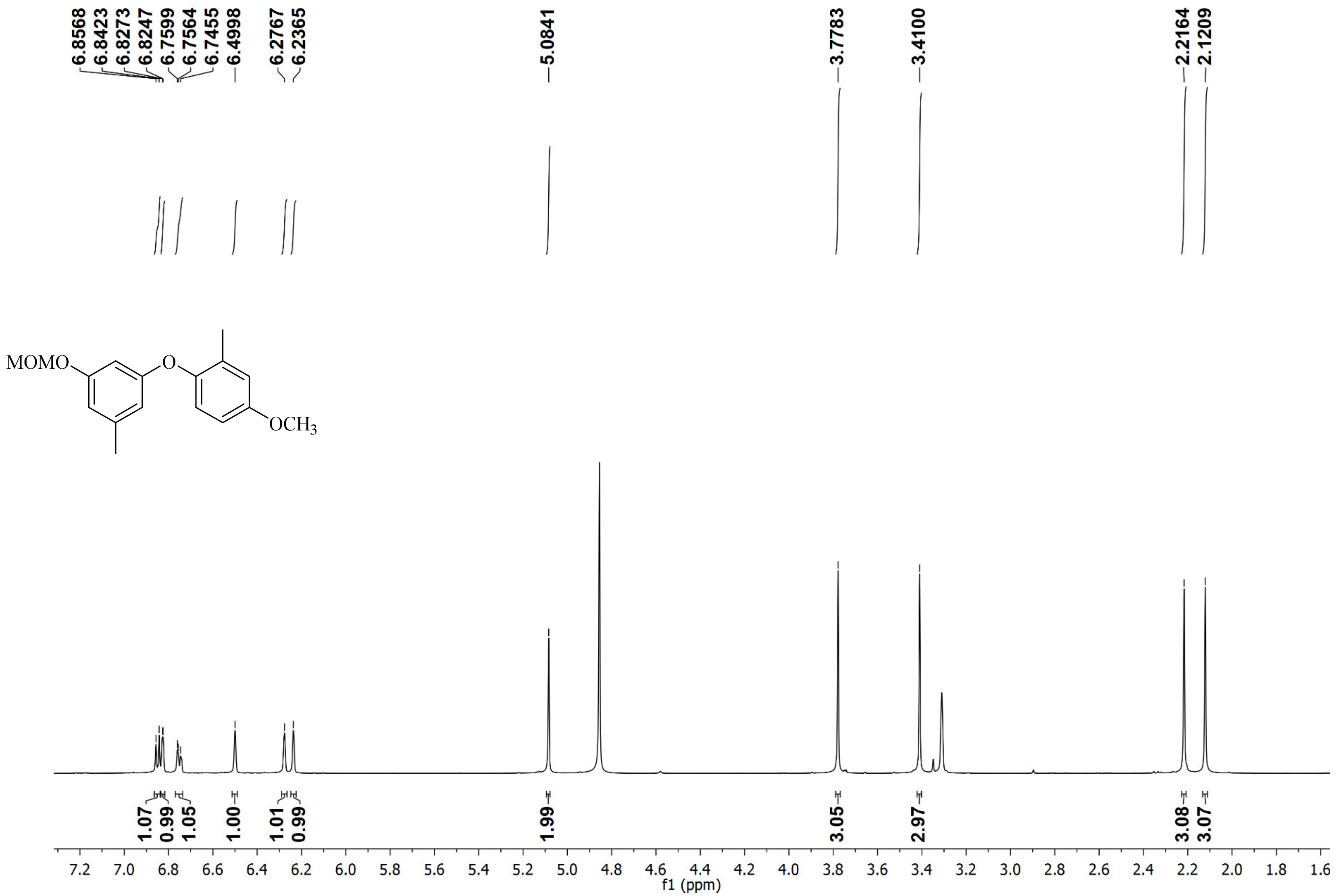

Figure S17.114. ^{1}H NMR spectrum of **23** in methanol-d_4.

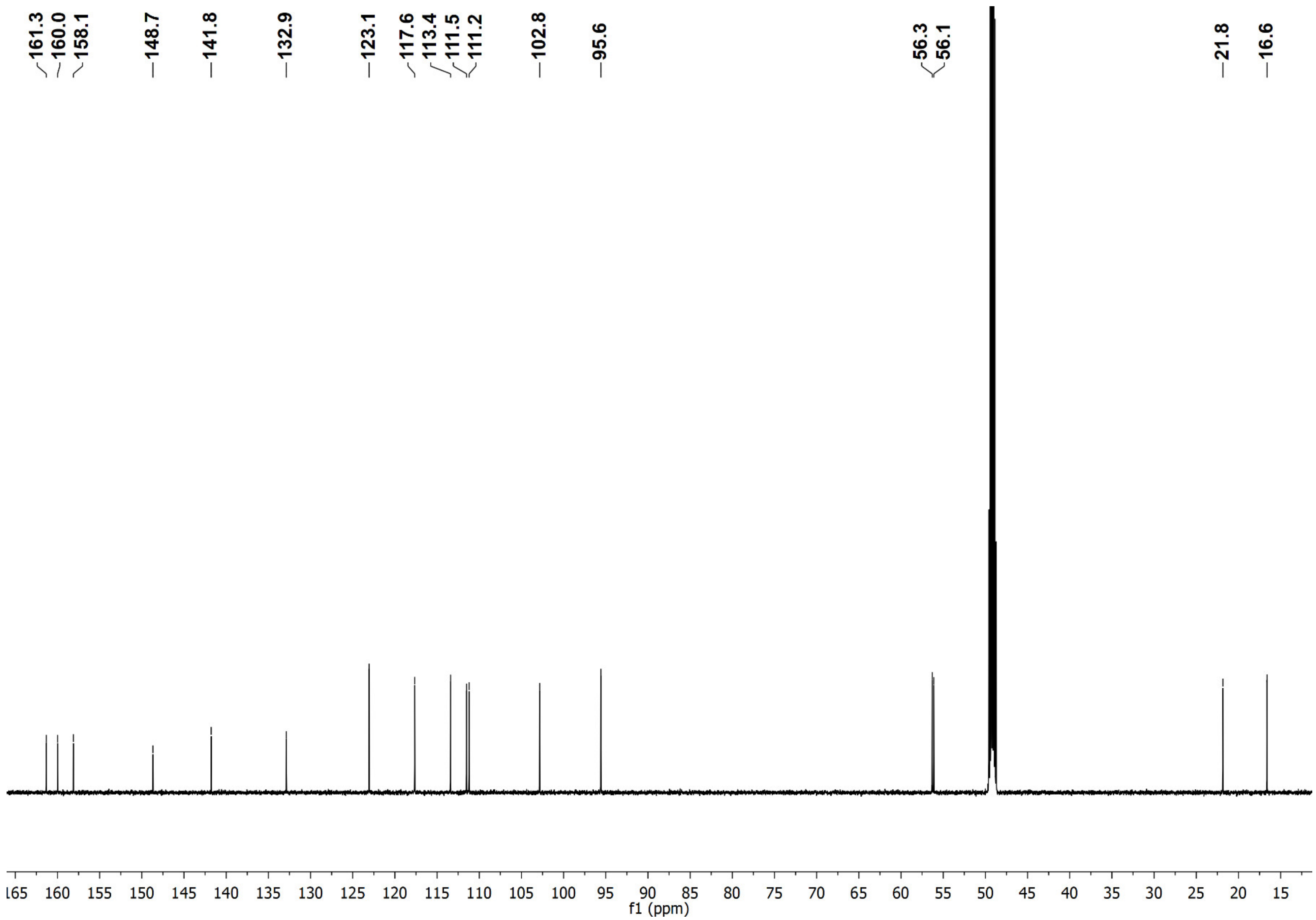

Figure S17.115. ^{13}C NMR spectrum of **23** in methanol-d_4.

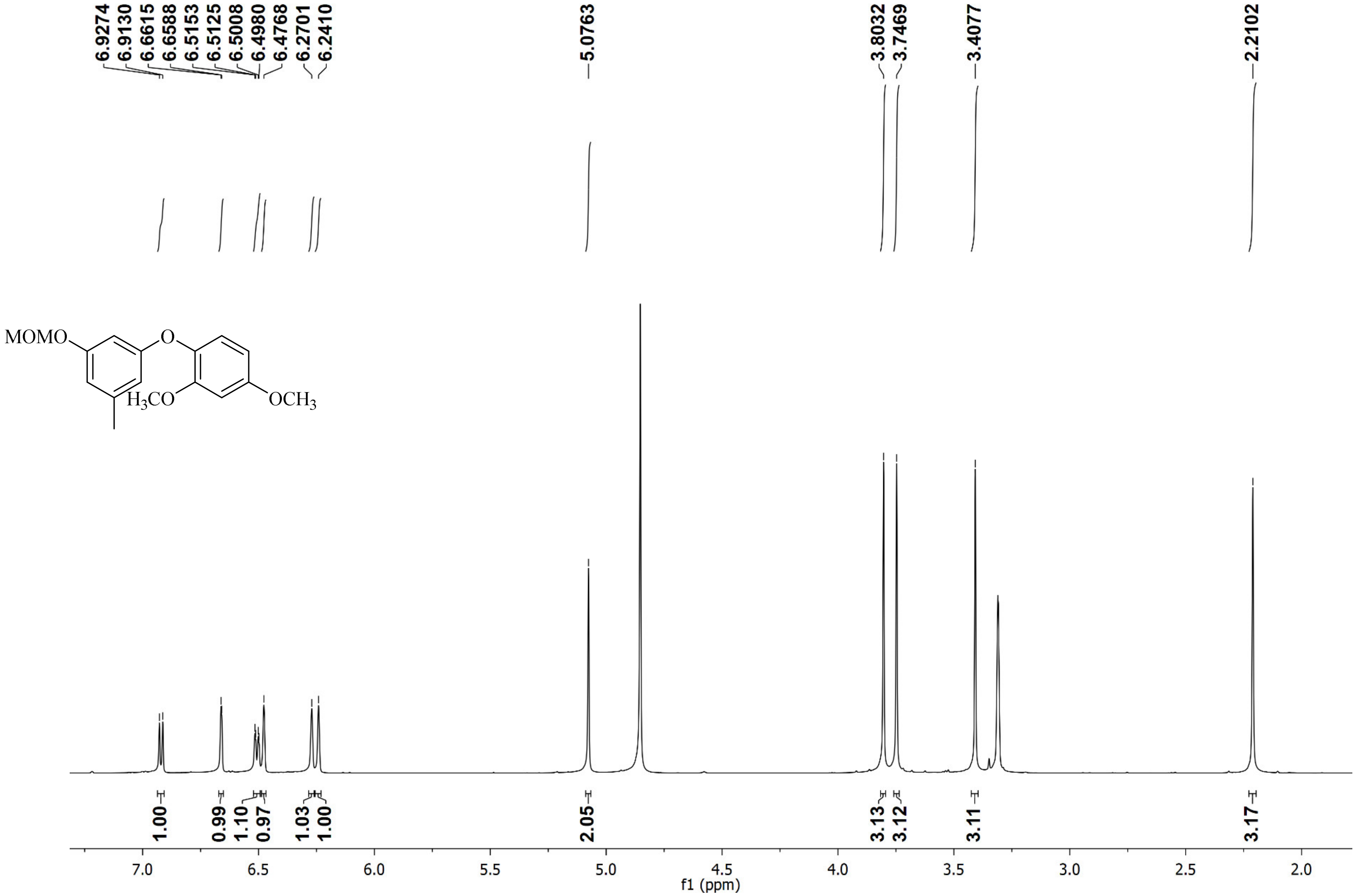

Figure S17.116. ^{1}H NMR spectrum of **24** in methanol-d_4.

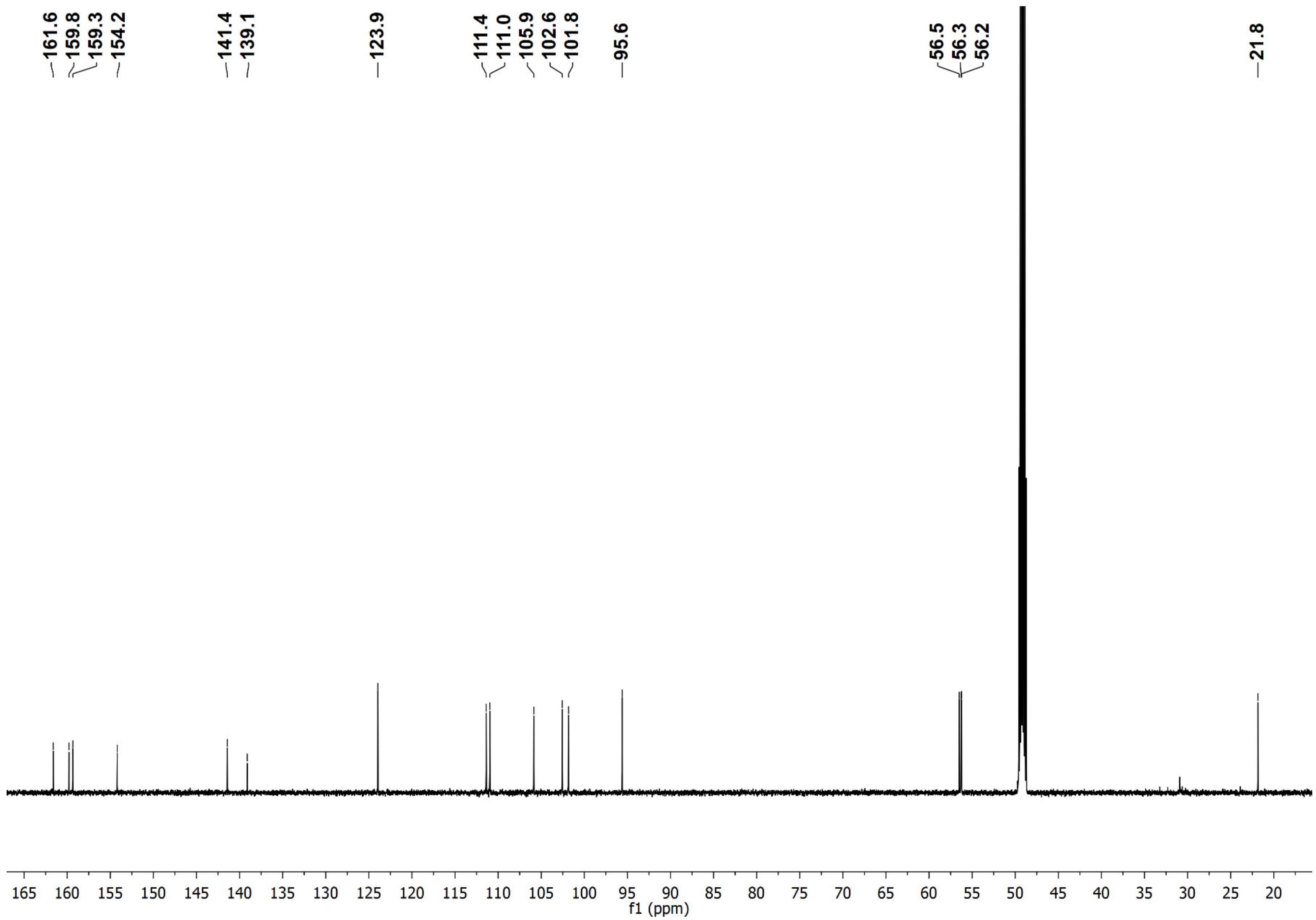

Figure S17.117. ^{13}C NMR spectrum of **24** in methanol-d_4.

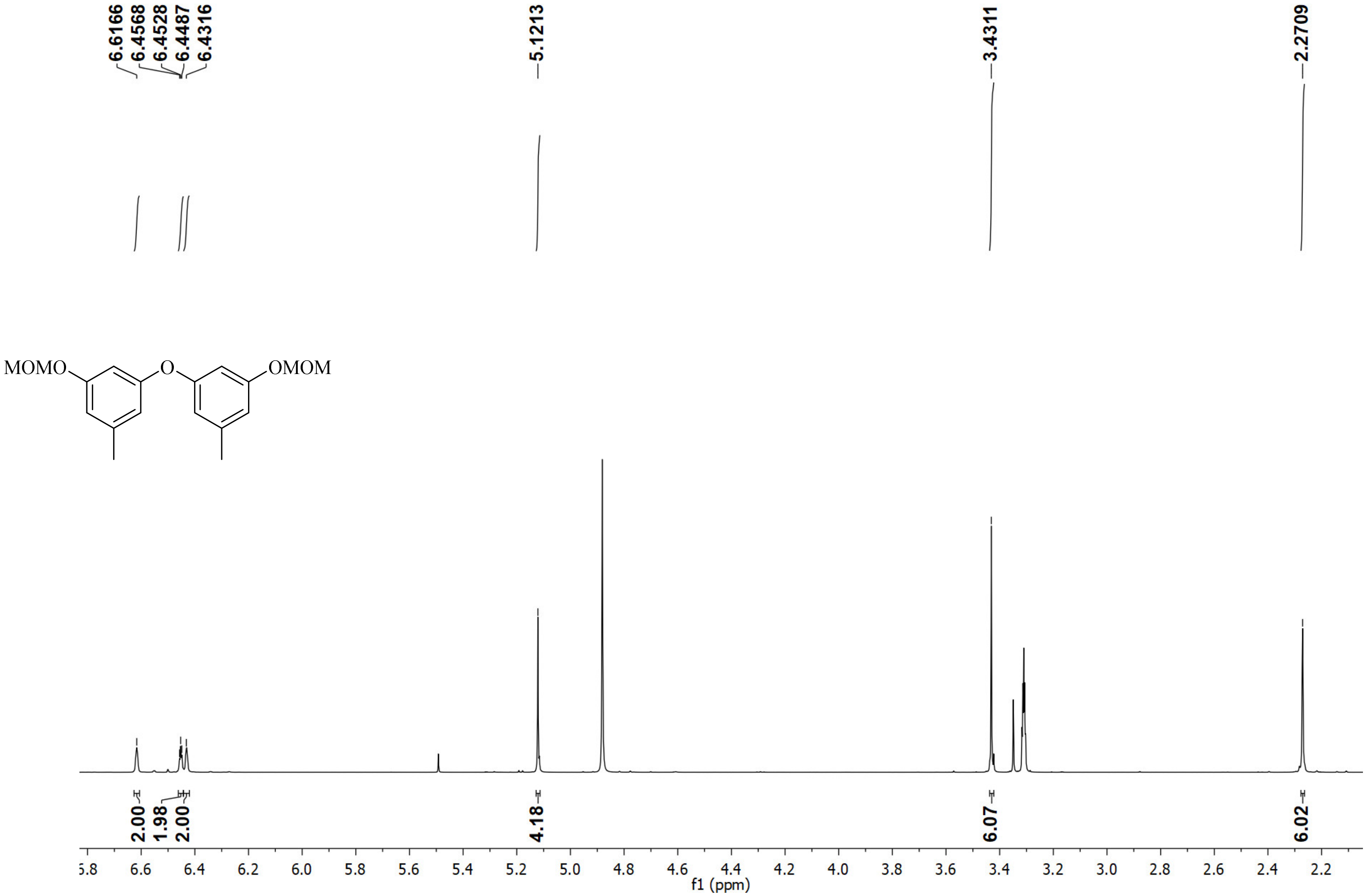

Figure S17.118. ^{1}H NMR spectrum of **25** in methanol-d_4.

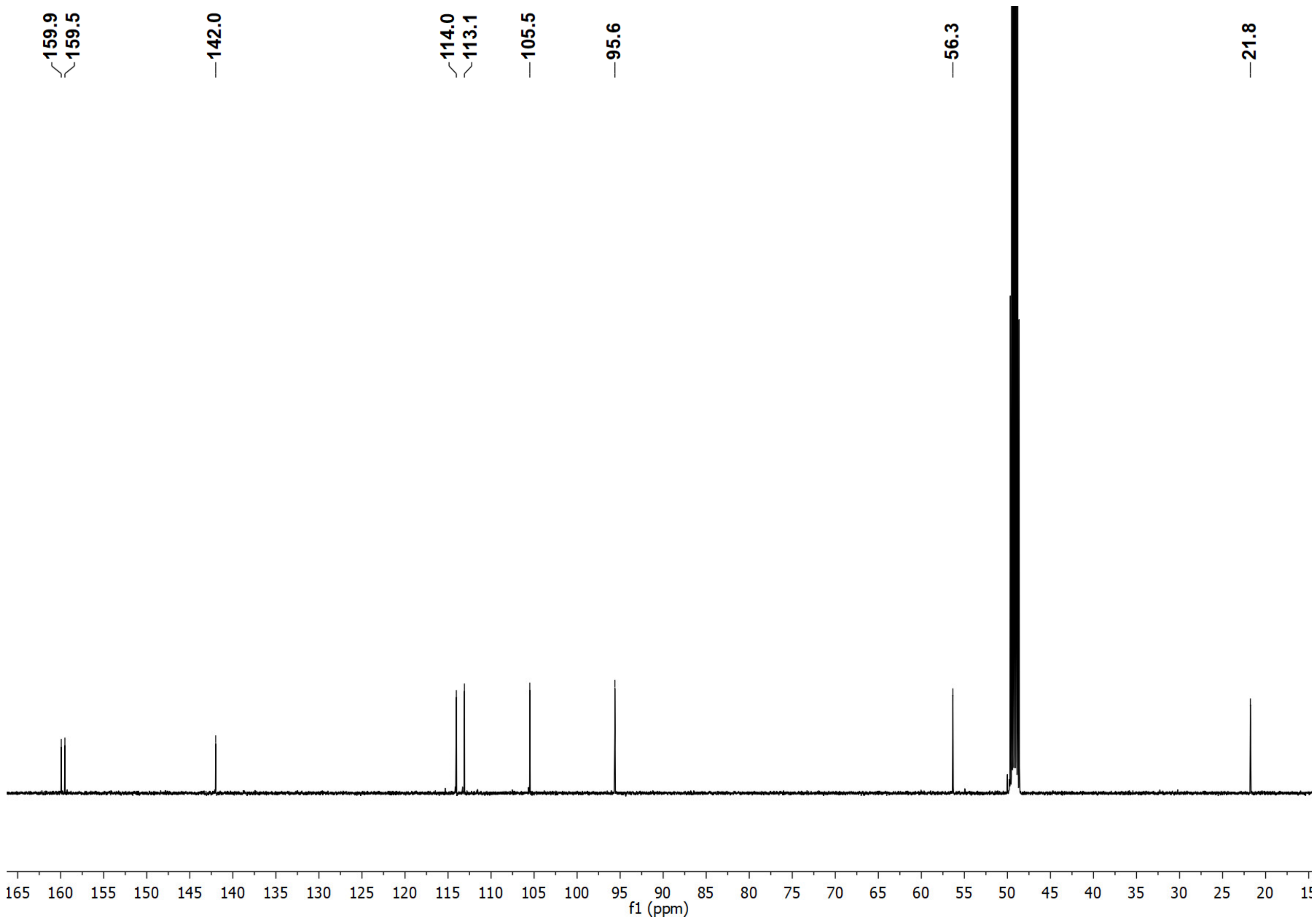

Figure S17.119. ^{13}C NMR spectrum of **25** in methanol-d_4.

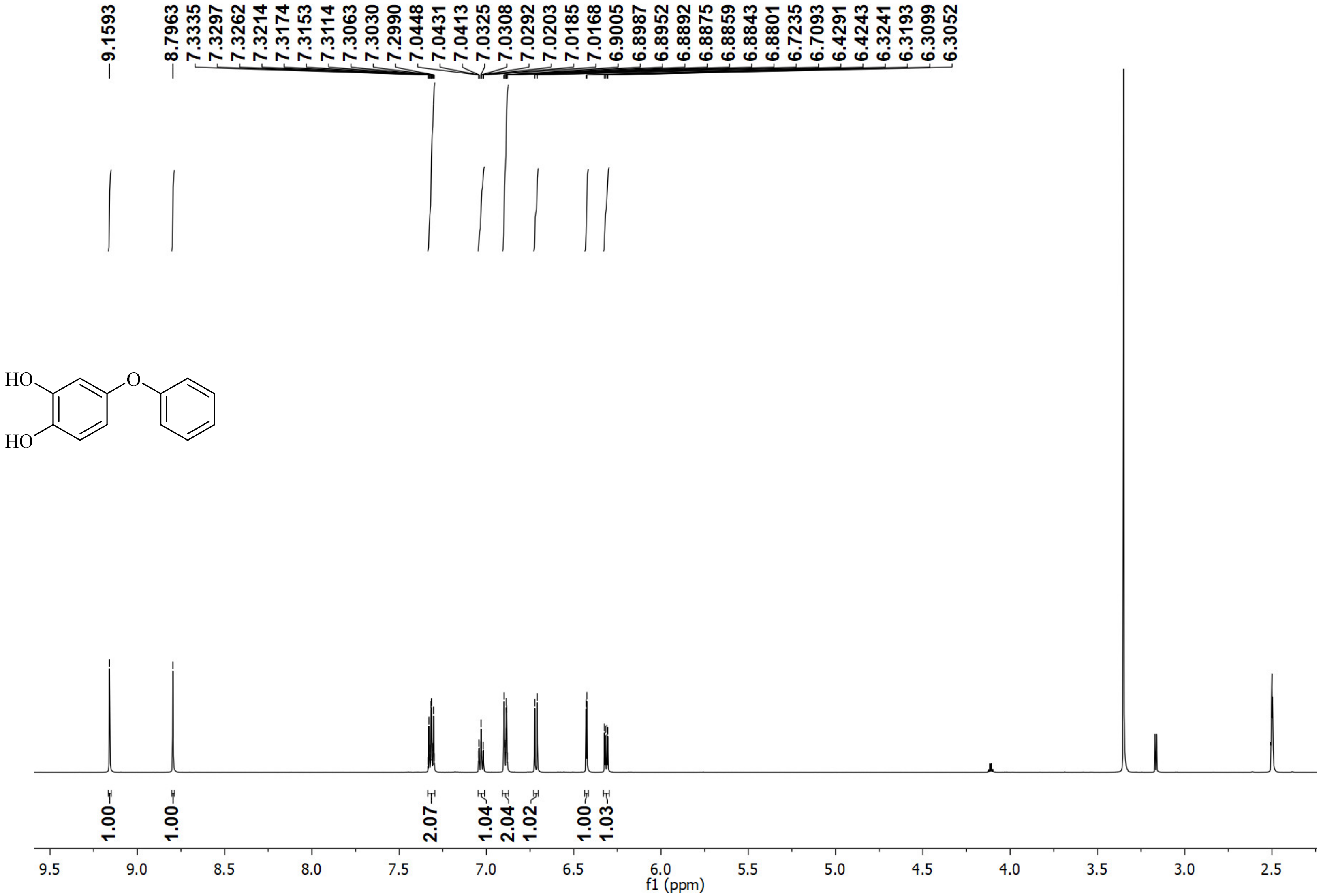

Figure S17.120. ^{1}H NMR spectrum of **26** in DMSO-d_6.

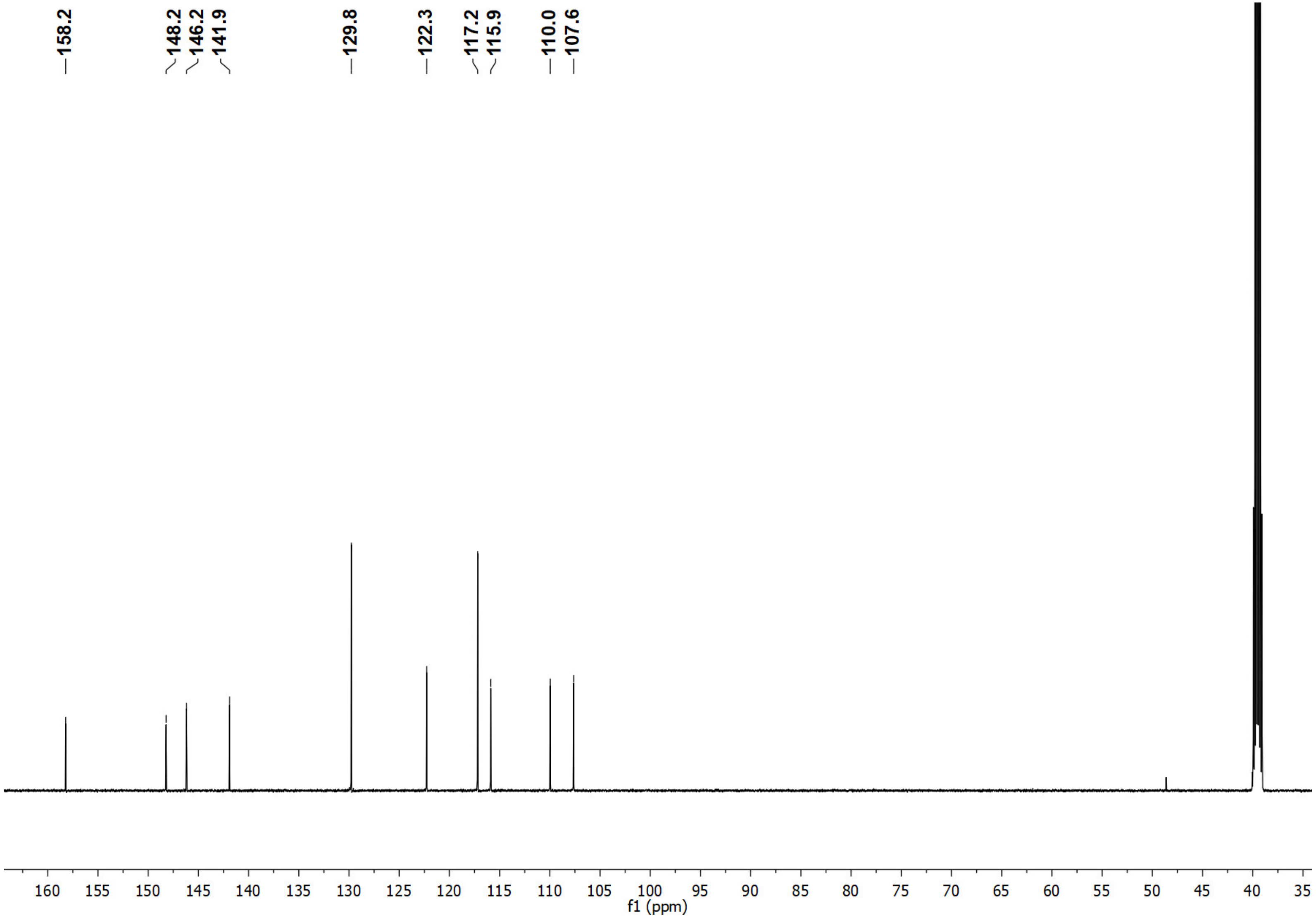

Figure S17.121. ^{13}C NMR spectrum of **26** in DMSO-d_6.

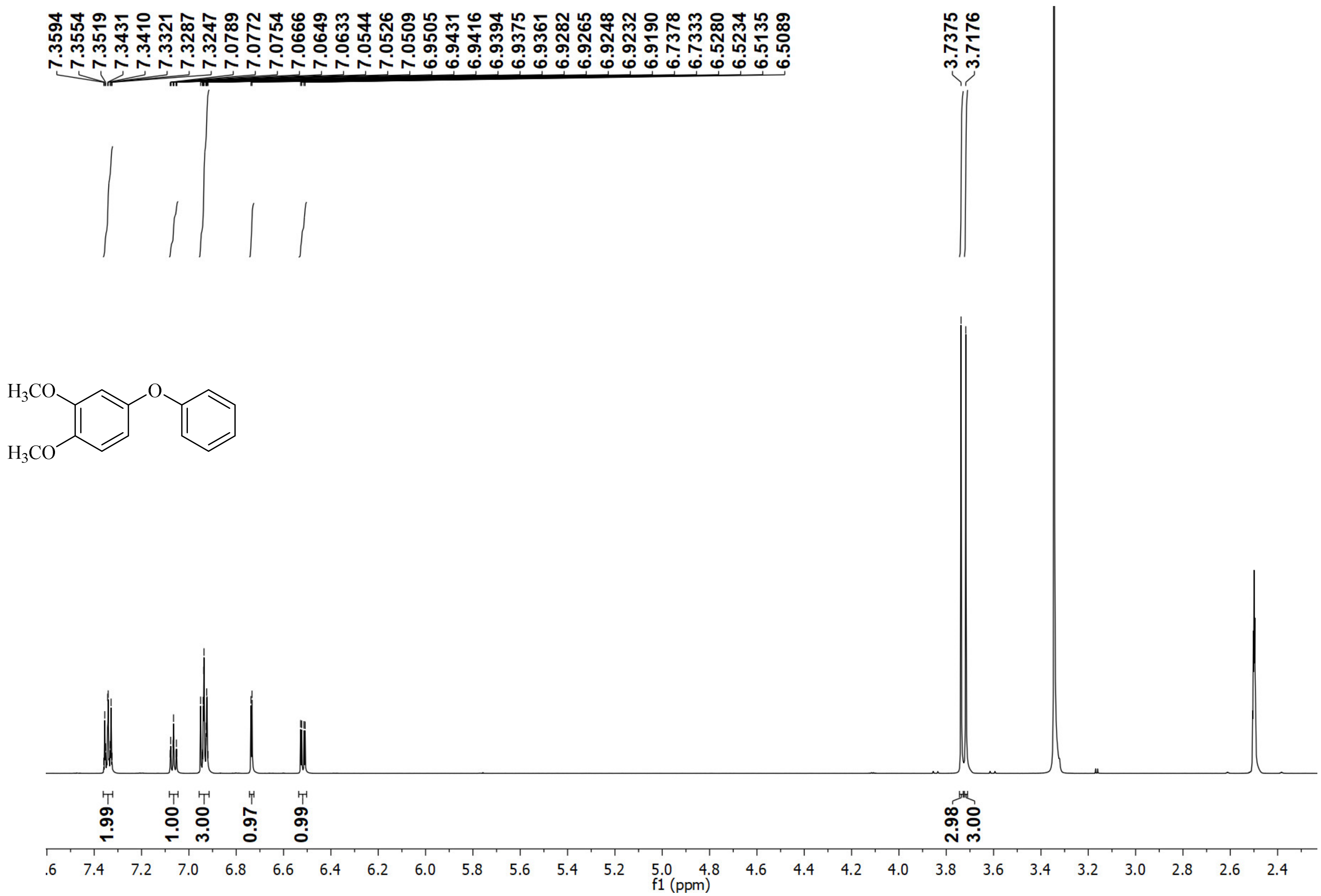

Figure S17.122. ^{1}H NMR spectrum of **27** in DMSO-d_6.

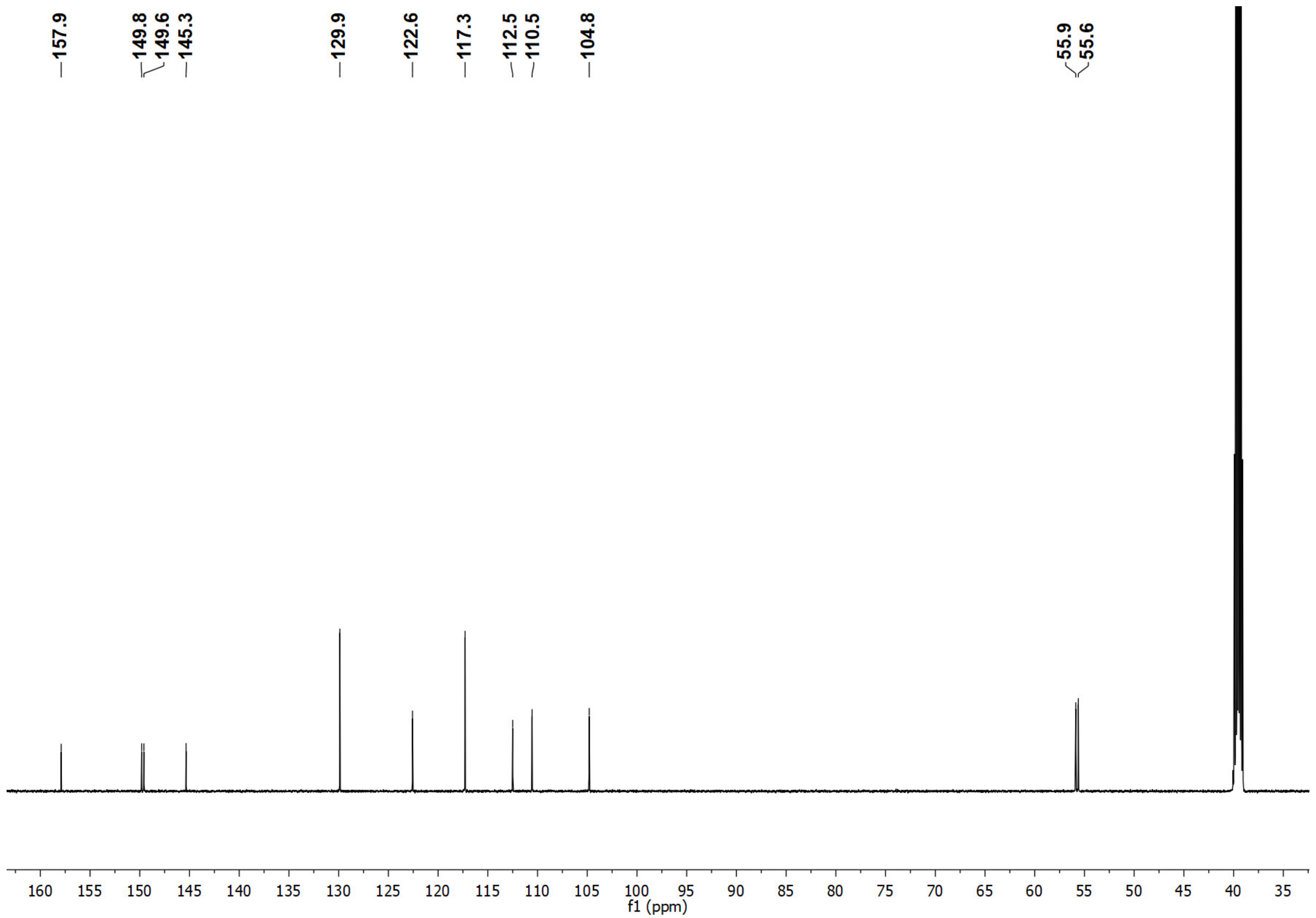

Figure S17.123. ^{13}C NMR spectrum of **27** in DMSO-d_6.

References

1 X. Wang, C. Wang, L. Duan, L. Zhang, H. Liu, Y. Xu, Q. Liu, T. Mao, W. Zhang, M. Chen, M. Lin, A. A. L. Gunatilaka, Y. Xu and I. Molnár, *J. Am. Chem. Soc.*, 2019, **141***,* 4355.

2 Q. Liu, D. Zhang, S. Gao, X. Cai, M. Yao, Y. Xu, Y. Gong, K. Zheng, Y. Mao, L. Yang, D. Yang, I. Molnár and X. Yang, *Angew. Chem. Int. Ed. Engl.*, 2023, **62**, e202214379.

3 Y. Matsuda, T. Wakimoto, T. Mori, T. Awakawa and I. Abe, *J. Am. Chem. Soc.*, 2014, **136**, 15326.

4 S. M. Ma, J. W. Li, J. W. Choi, H. Zhou, K. K. Lee, V. A. Moorthie, X. Xie, J. T. Kealey, N. A. Da Silva, J. C. Vederas and Y. Tang, *Science*, 2009, **326***,* 589.

5 R. D. Gietz and R. H. Schiestl, *Nat. Protoc*., 2007, **2**, 35.

6 K. Gmi, Y. Iimura and S. Hara, *Agric. Biol. Chem*., 1987, **51**, 2549.

7 C. Feng, Q. Wei, C. Hu and Y. Zou, *Org. Lett.*, 2019, **21**, 3114.

8 F. B. C. Okoye, S. Lu, C. S. Nworu, C. O. Esimone, P. Proksch, A. Chadli and A. Debbab, *Tetrahedron Lett*., 2013, **54**, 4210.

9 C. Yuan, Y. Guo, Y. Zhang, H. Wang, X. Hu, D. Wang, L. Guan, F. Yu and G. Li, *Acta Pharmacol. Sin.*, 2019, **54**, 892.

10 P. Chomcheon, S. Wiyakrutta, N. Sriubolmas, N. Ngamrojanavanich, S. Kengtong, C. Mahidol, S. Ruchirawat and P. Kittakoop, *Phytochemistry*, 2009, **70**, 407.

11 H. A. Weber and J. B. Gloer, *J. Nat. Prod*., 1988, **51**, 879.

12 A. Stierle, R. Upadhyay and G. Strobel, *Phytochemistry*, 1991, **30**, 2191.

13 X. Wu, L. Fang, F. Liu, X. Pang, H. Qin, T. Zhao, L. Xu, D. Yang and X. Yang, *RSC Adv.*, 2017, **7**, 31115.

14 A. Yajima, I. Shirakawa, N. Shiotani, K. Ueda, H. Murakawa, T. Saito, R. Katsuta and K. Ishigami, *Tetrahedron*, 2021, **92**, 132253.

15 D. Ma and Q. Cai, *Org. Lett.*, 2003, **5**, 3799.

16 A. Ninomiya, S. Urayama and D. Hagiwara, *Appl. Microbiol. Biotechnol.*, 2022, **106**, 4169.

17 C. Zhu, A. Yoshimura, L. Ji, Y. Wei, V. N. Nemykin and V. V. Zhdankin, *Org. Lett.*, 2012, **14**, 3170.

18 Z. Ghasemi, N. S. Shahrak, B. J. Roomi and Z. Zakeri, *J. Chem. Res.*, 2015, **39**, 73.

19 P. Zhang, X. Li, X. L. Yuan, Y. M. Du, B. G. Wang and Z. F. Zhang, *Molecules*, 2018, **23**, 3179.

20 L. L. Xu, C. C. Zhang, X. Y. Zhu, F. Cao and H. J. Zhu, *Nat. Prod. Res.*, 2017, **31**, 1875.

21 D. H. El-Kashef, F. S. Youssef, I. Reimche, N. Teusch, W. E. G. Müller, W. Lin, M. Frank, Z. Liu and P. Proksch, *Bioorg. Med. Chem.*, 2021, **29**, 115883.

22 Y. B. Ji, W. J. Chen, T. Z. Shan, B. Y. Sun, P. C. Yan and W. Jiang, *Chem. Biodivers.*, 2020, **17**, e1900640.

23 Y. Wu, Y. Chen, X. Huang, Y. Pan, Z. Liu, T. Yan, W. Cao and Z. She, *Mar. Drugs*, 2018, **16**, 307.

24 K. Takahashi, K. Sakai, W. Fukasawa, Y. Nagano, S. O. Sakaguchi, A. O. Lima, V. H. Pellizari, M. Iwatsuki, K. Takishita, T. Yoshida, K. Nonaka, K. Fujikura and S. Ōmura, *J. Antibiot.*, 2018, **71**, 741.

25 J. F. Sanchez, Y. M. Chiang, E. Szewczyk, A. D. Davidson, M. Ahuja, C. Elizabeth Oakley, J. Woo Bok, N. Keller, B. R. Oakley and C. C. Wang, *Mol. Biosyst.*, 2010, **6**, 587.

26 O. I. Zhuravleva, S. S. Afiyatullov, O. S. Vishchuk, V. A. Denisenko, N. N. Slinkina and O. F. Smetanina, *Arch. Pharm. Res.*, 2012, **35**, 1757.

27 O. I. Zhuravleva, N. N. Kirichuk and V. A. Denisenko, *Chem. Nat. Compd.*, 2016, **52**, 227.

28 J. F. Wang, X. P. Lin, C. Qin, S. R. Liao, J. T. Wan, T. Y. Zhang, J. Liu, M. Fredimoses, H. Chen, B. Yang, X. F. Zhou, X. W. Yang, Z. C. Tu and Y. H. Liu, *J. Antibiot.*, 2014, **67**, 581.

29 Y. N. Wang, Y. H. Mou, Y. Dong, Y. Wu, B. Y. Liu, J. Bai, D. J. Yan, L. Zhang, D. Q. Feng, Y. H. Pei and Y. C. Hu, *Mar. Drugs*, 2018, **16**, 451.

30 M. V. Nguyen, J. W. Han, H. Kim and G. J. Choi, *ACS omega*, 2022, **7**, 33273.

31 Z. X. Li, X. F. Wang, G. W. Ren, X. L. Yuan, N. Deng, G. X. Ji, W. Li and P. Zhang, *Molecules*, 2018, **23**, 2368.

32 R. F. Curtis, C. H. Hassall, D. W. Jones and T. W. Williams, *J. Am. Chem. Soc.*, 1960, 4838.

33 S. H. Wu, Y. W. Chen, S. Qin and R. Huang, *J. Basic Microbiol.*, 2008, **48**, 140.

34 S. Sureram, S. Wiyakrutta, N. Ngamrojanavanich, C. Mahidol, S. Ruchirawat and P. Kittakoop, *Planta Med.*, 2012, **78**, 582.

35 P. Aetang, V. Rukachaisirikul, S. Phongpaichit, S. Preedanon, J. Sakayaroj, S. Hadsadee and S. Jungsuttiwong, *J. Nat. Prod.*, 2021, **84**, 1498.

36 Z. H. Wu, Y. R. Wang, D. Liu, P. Proksch, S. W. Yu and W. H. Lin, *Tetrahedron*, 2016, **72**, 50.

37 H. Gao, L. Zhou, S. Cai, G. Zhang, T. Zhu, Q. Gu and D. Li, *J. Antibiot.*, 2013, **66**, 539.

38 S. S. Hu, N. Jiang, X. L. Wang, C. J. Chen, J. Y. Fan, G. Wurin, H. M. Ge, R. X. Tan and R. H. Jiao, *Tetrahedron Lett.*, 2015, **56**, 3894.

39 X. B. Li, Y. H. Zhou, R. X. Zhu, W. Q. Chang, H. Q. Yuan, W. Gao, L. L. Zhang, Z. T. Zhao and H. X. Lou, *Chem. Biodivers.*, 2015, **12**, 575.

40 H. Z. Weng, J. Y. Zhu, F. Y. Yuan, Z. Y. Tang, X. Q. Tian, Y. Chen, C. Q. Fan, G. H. Tang and S. Yin, *Mar. Drugs*, 2022, **20**, 322.

41 W. Liu, L. Wang, B. Wang, Y. Xu, G. Zhu, M. Lan, W. Zhu and K. Sun, *Mar. Drugs*, 2018, **17**, 6.

42 X. Wang, Y. Mou, J. Hu, N. Wang, L. Zhao, L. Liu, S. Wang and D. Meng, *Chem. Biodivers.*, 2014, **11**, 133.

43 Y. Chen, L. T. Sun, H. X. Yang, Z. H. Li, J. K. Liu, H. L. Ai, G. K. Wang and T. Feng, *Fitoterapia*, 2020, **141**, 104483.

44 F. Lünne, E. M. Niehaus, S. Lipinski, J. Kunigkeit, S. A. Kalinina and H. U. Humpf, *Fungal Genet. Biol.*, 2020, **145**, 103481.

45 H. Hussain, I. Kock, A. Al-Harrasi, G. Abbas, N. U. Rehman, A. Shah, A. Badshah, U. A. Rana, Z. Noureen, I. R. Green, S. Draeger, B. Schulz and K. Krohn, *J. Asian Nat. Prod. Res.*, 2014, **16**, 1094.

46 T. Bunyapaiboonsri, S. Yoiprommarat, K. Intereya and K. Kocharin, *Chem. Pharm. Bull.*, 2007, **55**, 304.

47 F. B. Okoye, C. S. Nworu, P. A. Akah, C. O. Esimone, A. Debbab and P. Proksch, *Immunopharmacol. Immunotoxicol.*, 2013, **35**, 662.

48 D. L. Zhao, C. L. Shao, C. Y. Wang, M. Wang, L. J. Yang and C. Y. Wang, *Molecules*, 2016, **21**, 160.

49 H. Oh, T. O. Kwon, J. B. Gloer, L. Marvanová and C. A. Shearer, *J. Nat. Prod.*, 1990, **62**, 580.

50 A. Cimmino, L. Maddau, M. Masi, M. Evidente, B. T. Linaldeddu and A. Evidente, *Tetrahedron*, 2016, **72**, 6788.

51 M. M. Salvatore, M. T. Russo, R. Nicoletti, A. E. Mahamedi, A. Berraf-Tebbal, A. Cimmino, M. Masi and A. Andolfi, *Molecules*, 2023, **28**, 6302.

52 M. Millot, S. Tomasi, E. Studzinska, I. Rouaud and J. Boustie, *J. Nat. Prod.*, 2009, **72**, 2177.

53 Q. Y. Song, Z. B. Nan, K. Gao, H. Song, P. Tian, X. X. Zhang, C. J. Li, W. B. Xu and X. Z. Li, *J. Agric. Food Chem.*, 2015, **63**, 8787.

54 J. Zhu, Z. Li, H. Lu, S. Liu, W. Ding, J. Li, Y. Xiong and C. Li, *Bioorg. Chem.*, 2021, **115**, 105232.

55 Y. Li, B. Sun, S. Liu, L. Jiang, X. Liu, H. Zhang and Y. Che, *J. Nat. Prod.*, 2008, **71**, 1643.

56 D. M. Nguyen, L. M. Do, V. T. Nguyen, W. Chavasiri, J. Mortier and P. P. Nguyen, *J. Nat. Prod.*, 2017, **80**, 261.

57 X. Kong, X. Ma, Y. Xie, S. Cai, T. Zhu, Q. Gu and D. Li, *Arch. Pharm. Res.*, 2013, **36**, 739.

58 J. D. M. de Sá, J. A. Pereira, T. Dethoup, H. Cidade, M. E. Sousa, I. C. Rodrigues, P. M. Costa, S. Mistry, A. M. S. Silva and A. Kijjoa, *Mar. Drugs*, 2021, **19**, 457.

59 T. H. Do, T. T. Nguyen, T. B. Dao, H. C. Vo, B. L. Huynh, T. A. Nguyen, D. T. Mai, T. P. Vo, J. Sichaem, N. H. Nguyen and T. H. Duong, *Nat. Prod. Res.*, 2022, **36**, 4879.

60 B. L. C. Huynh, V. M. Bui, K. P. P. Nguyen, N. K. T. Pham and T. P. Nguyen, *Nat. Prod. Res.*, 2022, **36**, 1934.

61 J. F. Wang, L. M. Zhou, S. T. Chen, B. Yang, S. R. Liao, F. D. Kong, X. P. Lin, F. Z. Wang, X. F. Zhou and Y. H. Liu, *Fitoterapia*, 2018, **125**, 49.

62 F. Zhan, T. Yang, Y. P. Han and G. Y. Li, *Nat. Prod. Res.*, 2013, **27**, 1393.

63 T. Ogawa, K. Ando, Y. Aotani, K. Shinoda, T. Tanaka, E. Tsukuda, M. Yoshida and Y. Matsuda, *J. Antibiot.*, 1995, **48**, 1401.

64 X. Xu, L. Liu, F. Zhang, W. Wang, J. Li, L. Guo, Y. Che and G. Liu, *Chembiochem*, 2014, **15**, 284.

65 H. Jayasuriya, R. G. Ball, D. L. Zink, J. L. Smith, M. A. Goetz, R. G. Jenkins, M. Nallin-Omstead, K. C. Silverman, G. F. Bills and R. B. Lingham, *J. Nat. Prod.*, 1995, **58**, 986.

66 Z. J. Li, H. Y. Yang, J. Li, X. Liu, L. Ye, W. S. Kong, S. Y. Tang, G. Du, Z. H. Liu, M. Zhou, G. Y. Yang, Q. F. Hu and X. M. Li, *J. Antibiot.*, 2018, **71**, 359.

67 X. Han, H. Gao, H. Lai, W. Zhu and Y. Wang, *J. Nat. Prod.*, 2023, **86**, 882.

68 B. N. S. Ningsih, V. Rukachaisirikul, S. Pansrinun, S. Phongpaichit, S. Preedanon and J. Sakayaroj, *Nat. Prod. Res.*, 2022, **36**, 4982.

69 P. Saetang, V. Rukachaisirikul, S. Phongpaichit, S. Preedanon, J. Sakayaroj, S. Borwornpinyo, S. Seemakhan and C. Muanprasat, *Phytochemistry*, 2017, **143**, 115.

70 Z. K. Guo, W. Y. Zhu, L. X. Zhao, Y. C. Chen, S. J. Li, P. Cheng, H. M. Ge, R. X. Tan and R. H. Jiao, *Chin. J. Nat. Med.*, 2022, **20**, 627.

71 D. Schreiber, M. Jung, L. P. Sandjo, J. C. Liermann, T. Opatz and G. Erkel, *J. Antibiot.*, 2012, **65**, 473.

72 F. Ismed, F. Lohézic-Le Dévéhat, O. Delalande, S. Sinbandhit, A. Bakhtiar and J. Boustie, *Fitoterapia*, 2012, **83**, 1693.

73 B. N. S. Ningsih, V. Rukachaisirikul, S. Phongpaichit, C. Muanprasat, S. Preedanon, J. Sakayaroj, R. Intayot and S. Jungsuttiwong, *Nat. Prod. Res.*, 2023, **15**, 1.

74 V. Ivanova, M. Kolarova, K. Aleksieva, U. Graefe and B. Schlegel, *Prep. Biochem. Biotechnol.*, 2007, **37**, 39.

75 W. Peng, F. You, X. L. Li, M. Jia, C. J. Zheng, T. Han and L. P. Qin, *Chin. J. Nat. Med.*, 2013, **11**, 673.

76 L. Du, J. B. King, B. H. Morrow, J. K. Shen, A. N. Miller and R. H. Cichewicz, *J. Nat. Prod*., 2012, **75**, 1819.
